# Late-Stage Outcomes as Surrogates for Mortality in Cancer Screening Trials: A Systematic Review and Meta-analysis

**DOI:** 10.1158/1055-9965.EPI-25-0201

**Published:** 2025-07-22

**Authors:** Matejka Rebolj, Adam R. Brentnall, Julia Geppert, Nefeli Kouppa, Bethany Shinkins, Karoline Freeman, Chris Stinton, Matthew J. Randell, Samantha Johnson, Robert A. Smith, Peter Sasieni, Sam M. Janes, Ruth Etzioni, Stephen W. Duffy, Sian Taylor-Phillips

**Affiliations:** 1 Centre for Cancer Screening, Prevention, and Early Detection, Wolfson Institute of Population Health, Queen Mary University of London, London, United Kingdom.; 2 Centre for Evaluation and Methods, Wolfson Institute of Population Health, Queen Mary University of London, London, United Kingdom.; 3 Warwick Screening, Warwick Applied Health, Warwick Medical School, University of Warwick, Coventry, United Kingdom.; 4 University of Warwick Library, University of Warwick, Coventry, United Kingdom.; 5 American Cancer Society Center for Early Cancer Detection Science, American Cancer Society, Atlanta, Georgia.; 6 Division of Medicine, Lungs for Living Research Centre, University College London, London, United Kingdom.; 7 Division of Public Health Sciences, Biostatistics Program, Fred Hutchinson Cancer, Seattle, Washington.

## Abstract

Late-stage cancer incidence has been proposed as a surrogate outcome for cancer-specific mortality in future screening trials. Two previous meta-analyses with 33 and 39 trials assessed trial-level surrogacy but provided inconsistent conclusions about the suitability of late-stage cancer endpoints replacing mortality. Our systematic review and meta-analysis (PROSPERO ID, CRD42023369320) investigated the association between the effect of cancer screening on the incidence of late-stage cancer and cancer-specific mortality. From 57 trials with 61 trial arm comparisons, correlation between late-stage incidence and mortality outcomes was 0.69 [95% confidence interval (CI), 0.47–0.84] for all cancers combined. Specifically, correlations were 0.58 (95% CI, 0.27–0.93) for bowel (*N* = 11 trials), 0.79 (95% CI, 0.49–0.94) for breast (*N* = 13), and 0.91 (95% CI, 0.84–0.96) for lung cancer (*N* = 14). Trial point estimates of the screening effect on mortality were within each trial’s 95% CI late-stage incidence estimates in 56 of 61 (92%) trial arm comparisons and in 16 of 19 (84%) trial arm comparisons in which the entire 95% CI for screening effect on late-stage incidence was below one. Evidence suggests potential for late-stage cancer incidence as a key outcome in screening trials, but further research is needed to clarify when to measure late-stage outcomes, extrapolation for cancer types without trials, and the conditions when late-stage cancer does not accurately predict mortality.

## Introduction

Guideline development methodology prioritizes randomized controlled trial (“trial”) evidence on reduced cancer-specific mortality before recommending adoption of a new cancer screening program (1). Although a long-standing criterion for a high degree of confidence in the efficacy of a screening test, this can be a practical barrier to timely implementation of effective screening interventions because evaluating mortality in apparently healthy, asymptomatic screening cohorts is challenging, slow, and expensive. Randomized cancer screening trials powered for cancer-specific mortality need to be large, commonly in the tens (if not hundreds) of thousands of individuals, and are typically conducted with a follow-up duration of one to two decades. This also poses a risk that by the time the results can be reported, the trialed screening technology may have become obsolete (2). Thus, there is interest in the possibility of using surrogate primary outcomes instead of cancer-specific mortality to facilitate faster and more efficient trials that ultimately support an earlier (planning of the) implementation of effective screening tests before direct evidence on mortality is available (3). Although some cancer screening trials designed to report surrogate primary outcomes are underway (4), there is no consensus on how or whether to utilize these endpoints as predictors of mortality, especially for the evaluation of a new screening test. Consequently, surrogate endpoints have largely not been adopted into decision-making by expert advisory groups responsible for national policy or guidelines.

The relationship between a reduction in late-stage diagnoses and mortality in cancer screening is complex and can differ across the spectrum of cancer locations and subtypes (5–7). For most cancer types, stage at diagnosis is prognostic of both morbidity and short- and long-term survival (8–11). Here, it is reasonable to expect that a stage shift from a late-stage to an early-stage diagnosis would be followed by a substantial reduction in cancer-specific mortality. Although nearly all cancers have better 5-year survival for earlier- versus late-stage disease, there is variation in the difference in prognosis across cancer types (12–15). In settings in which there is little difference in prognosis between earlier- versus late-stage disease groups, it is less likely that a stage shift would be followed by a substantial reduction in cancer-specific mortality. Conversely, earlier detection through screening might permit more successful treatment even though there was no stage shift. This might lead to a reduction in mortality but no effect would be predicted based simply on stage at diagnosis, whereas more granular evaluation of tumor characteristics within and across groups could be more informative. Whether late-stage incidence is a reasonable surrogate in the light of these effects requires in-depth study (16).

Previous studies on this topic include meta-analytic evaluations of early indicators of a mortality reduction in breast screening, which found high trial-level correlations between the screening effect on late-stage breast cancer and breast cancer mortality in mammography trials (17, 18). Two recent meta-analytic evaluations included more cancer types but reached different conclusions about the suitability of late-stage outcomes as surrogates for mortality across multiple cancer types (Supplementary Methods and Tables, Section 1, Supplementary Tables S1 and S2; refs. 19, 20). Following their search strategies and inclusion criteria, these meta-analyses included 33 and 39 trials, respectively, for which one concluded that there was compelling evidence for the use of late-stage alternative endpoints in future cancer screening trials and the other concluded that late stage is not a suitable alternative endpoint for some cancer types.

In this article, we report a systematic literature review of cancer screening trials that reported both cancer-specific late-stage diagnoses and mortality outcomes and included any testing technology, cancer type, or trial size, following the inclusion criteria and other decisions made available in a pre-published protocol [PROSPERO ID: CRD42023369320; Supplementary Methods (Protocol)]. Many different criteria for surrogate endpoints have been developed. Here, we evaluate surrogacy by following the definition used to support the development of extensions to the CONSORT and SPIRIT checklists for studies reporting surrogate endpoints, that is, “An outcome that is used in clinical trials as a substitute for a direct measure of how a patient feels; functions; or survives. A surrogate outcome does not measure the clinical benefit of primary interest in and of itself; but rather is expected to predict that clinical benefit or harm based on epidemiologic; therapeutic; pathophysiologic; or other scientific evidence.” (21). Through this broad lens, we consider how well detection at an earlier stage at diagnosis through screening, measured through a reduction in the rate of late-stage cancer, can be seen to predict the clinical benefit of reduced cancer-specific mortality. We use meta-analysis to evaluate the association between a change in the relative incidence of late-stage cancer and a change in cancer-specific mortality at a trial level or the level of the class of screening test. We further consider whether one would be able to substitute evaluation of a screening test using a reduction in cancer-specific mortality with analysis using a reduction in a late-stage surrogate.

## Materials and Methods

### Literature search

The systematic search of the literature was undertaken in two stages. Both stages are explained in detail in Supplementary Methods and Tables, Section 2, Supplementary Tables S3–S11. Briefly, the aim of the first stage (“Search 1”) was to identify trials of primary screening for any human cancer, using any screening test, that reported the mortality outcomes, without size, time, or other restrictions except English language. These searches were undertaken in the MEDLINE, Embase, and Web of Science databases and included publications registered therein by the 21st of September 2022. The second stage (“Search 2”) was undertaken in the MEDLINE and Embase databases for publications registered until the period spanning from the 11th of January 2023 to the 28th of April 2023 (the exact date depended on the trial) and aimed to identify, for each trial, all relevant publications reporting outcomes that might be considered as surrogates for mortality (see below for the definitions) or any additional mortality outcomes not identified by “Search 1”. If additional trials were identified at this stage, they were included in the review. “Search 1” was supplemented by contacting cancer screening experts, checking the lists of trials included in the most recent cancer screening reviews by independent bodies such as the International Agency for Research on Cancer and the U.S. Preventive Services Task Force, and by publications identified via “Search 2”. To supplement “Search 2,” we checked the introduction and discussion sections of articles included in the mapping and data extraction phases for any previously unidentified references. Because of funder deadlines, no additional data were requested from the trialists.

### Study selection

Titles and abstracts of the publications found by the searches were screened independently by the same two reviewers in both stages (J. Geppert and M. Rebolj), consulting all full-text publications considered potentially relevant by either reviewer. Full-text publications were assessed against the inclusion and exclusion criteria (Supplementary Methods and Tables, Section 2, Supplementary Tables S3 and S6) independently by five reviewers (J. Geppert, M. Rebolj, K. Freeman, N. Kouppa, and C. Stinton), with disagreements resolved by a third reviewer (A.R. Brentnall, M. Rebolj, and S. Taylor-Phillips).

In “Search 1,” individual or cluster randomized controlled trials were eligible for inclusion if they reported cancer-specific or all-cause mortality outcomes for all arms. In “Search 2,” publications were eligible if they reported outcomes that might be considered as surrogates (see below for definitions) or further mortality outcomes for trials identified in “Search 1.” In both stages, we excluded non-randomized studies; nonhuman studies, letters, reviews, editorials, and communications with insufficient information on methods and/or no numerical outcomes data; gray literature and conference abstracts; and articles not available in the English language. Reasons for exclusions at the full-text level are reported in Supplementary Methods and Tables, Section 2, Supplementary Tables S5 (“Search 1”) and S9 (“Search 2”).

**Figure 1.**
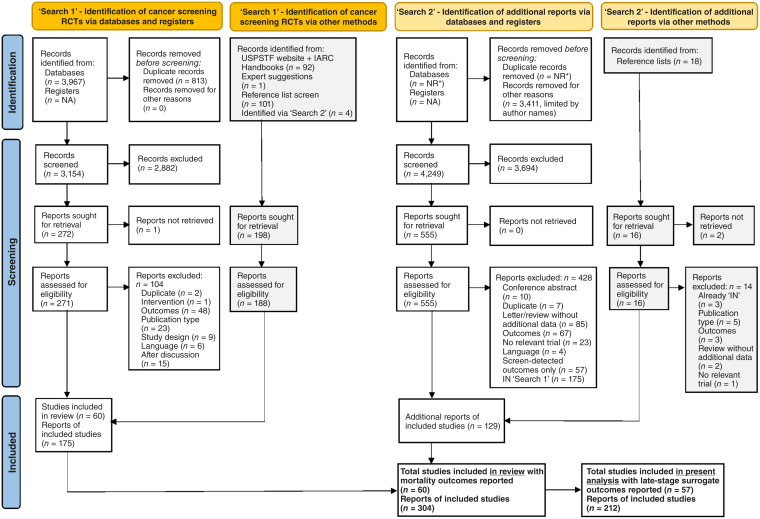
PRISMA flowchart for the systematic search of the literature. The figure describes the process of identifying, screening, and selecting the studies for the systematic review. * As half of the original MEDLINE search results (prior deduplication) were inadvertently overwritten, the original number retrieved is not available for “Search 2”. Records: titles with abstracts. Reports: full-text articles. List of reasons for exclusion with explanation. Duplicate: the same abstract was included twice for full-text assessment i.e., it was identified from different databases and not picked up as a duplicate at the de-duplication stage. Intervention: the intervention was not invitation or no invitation to screening with a well-defined test, but study group subjects were urged to have annual multiphasic health checkups, with screening tests not being defined. Outcomes: Search 1: no relevant mortality outcomes reported for all arms (all cause or cancer specific). Search 2: no relevant comparative surrogate outcomes specified in our protocol were reported. Publication type: included letters, reviews, editorials, and communications with insufficient information on methods and/or no numerical outcomes data; gray literature and conference abstracts. Study design: not a randomized controlled trial. Language: the report was not published in English. After discussion: these are full texts for which the two reviewers (MR/JG) initially disagreed on the inclusion but agreed on the exclusion after a discussion. Conference abstract: only an abstract was reported. Letter/review without additional data: these are letters or reviews that reported sufficient information on methods but did not report any additional outcomes data that we have not already identified from other reports. No relevant trial: this report was not on a trial that could be included in the study. Screen-detected outcomes only: these reports only included outcomes from the intervention arm e.g., proportion of screen-detected cancers. IN “Search 1”: reports identified in “Search 2” that were already included after “Search 1”. Already “IN”: reports that were already identified and included via “Search 1” or “Search 2” database searches. Review without additional data: previous reviews that reported sufficient information on methods but did not report any additional outcome data that we have not identified already from other reports. IARC, International Agency for Research on Cancer; NA, not applicable; NR, not reported; USPSTF, US Preventive Services Task Force.

For each trial, all identified publications were mapped by the reported outcomes and timepoints, and publications that did not provide additional data were excluded from the data extraction. Figure 1 shows the PRISMA flowchart for the two search stages combined. The designs and other characteristics of the included trials are summarized in Table 1; more details can be found in Supplementary Methods and Tables, Section 3, Supplementary Tables S12 and S13.

**Table 1.** Summary of the design and other characteristics of the 57 trials included in the systematic review and meta-analysis.

Cancer type	Trial acronym or name	Country	Calendar years of enrollment	Population type	Population size	Intervention arm(s)	Control arm
Bowel	Finnish	Finland	2004–2012^a^	General	362,165	FOBT	Usual care
	Funen	Denmark	1985	General	61,933	FOBT	Usual care
	Gothenburg	Sweden	1982–1990	General	Cohorts 1–3: 68,308	FOBT	Usual care
	Minnesota	United States	1975–1978	General	46,551	(1) FOBT (annual) (2) FOBT (biennial)	Usual care
	NORCCAP	Norway	1999–2000 (55–64 y); 2001 (50–54 y)	General	100,210 (55,736 aged 55–64 years)	FS (±FOBT; 1:1)	Usual care
	NordICC	Poland, Norway, and Sweden (Netherlands)	2009–2014	General	Poland, Norway, and Sweden: 85,179	Colonoscopy	Usual care
	Nottingham	United Kingdom	Pilot: 1981–1983 Main: 1985–1991	General	152,850	FOBT	Usual care
	PLCO (bowel)	United States	1993–2001	General	154,887	FS	Usual care
	SCORE	Italy	1995–1999	General	34,292	FS	Usual care
	Telemark	Norway	1983	General	799	FS (±colonoscopy after 13 years)	Usual care
	UKFSST	United Kingdom	1994–1999	General	170,432	FS	Usual care
Breast	CNBSS-1	Canada	1980–1985	General	50,430	Mammography + CBE	CBE (1 round)
	CNBSS-2	Canada	1980–1985	General	39,405	Mammography + CBE	CBE
	Edinburgh	United Kingdom	1978–1985	General	54,671 (cohort 1: 44,288)	Mammography + CBE	Usual care
	Gothenburg	Sweden	1982–1984	General	52,833	Mammography	Usual care
	HIP NY	United States	1963–1964	General	∼62,000	Mammography + CBE	Usual care
	Malmo	Sweden	1976–1978	General	42,283	Mammography	Usual care
	Mumbai	India	1998–2002	General	151,538	CBE	Usual care
	Russia/WHO	Russia	1985–1989	General	Leningrad: 122,471	BSE	Usual care
	Shanghai	China	1989–1991	General	267,400	BSE	Usual care
	Stockholm	Sweden	1981–1983	General	60,261	Mammography	Usual care
	Trivandrum	India	Initiated 2006	General	115,290	CBE	Usual care
	Two-County	Sweden	1977–1980 (Kopparberg); 1978–1981 (Ostergotland)	General	162,981 (134,867 aged 40–74 years)	Mammography	Usual care
	UK Age	United Kingdom	1990–1997	General	160,921	Mammography	Usual care
Cervical	Finnish	Finland	1999–2003	General, high risk	520,312	Papnet cytology	Conventional cytology
	Mumbai	India	1998–2002	General	151,538	VIA	Usual care
	Osmanabad	India	Initiated 2000	General	131,806	(1) VIA (2) Conventional cytology (3) HPV testing	Usual care
	Tamil Nadu	India	Initiated 1999	General	80,282	VIA	Usual care
Liver	Qidong	China	1989–1992	High risk	5,581	AFP + ALT (3–6 rounds)	AFP + ALT (1 round)
	Shanghai	China	1993–1995	High risk	19,200	AFP + US	Usual care
Lung	Czech Study	Czech	1976–1977	High risk	6,345	CXR + sputum cytology	CXR + sputum cytology (less intensive schedule)
	DANTE	Italy	2001–2006	High risk	2,811	LDCT + clinical review (baseline: + CXR + sputum cytology)	Clinical review (baseline: + CXR + sputum cytology)
	DLCST	Denmark	2004–2006	High risk	4,104	LDCT	Usual care
	ITALUNG	Italy	2004–2006	High risk	3,206	LDCT	Usual care
	Johns Hopkins	United States	1973–1978	High risk	10,387	CXR + sputum cytology	CXR
	LSS	United States	2000	High risk	3,318	LDCT	CXR
	LUSI	Germany	2007–2011	High risk	4,052	LDCT	Usual care
	MLP	United States	1971–1976	High risk	9,211	CXR + sputum cytology	Usual care
	MSK	United States	1974–1978	High risk	10,040	CXR + sputum cytology	CXR
	MILD	Italy	2005–2011	High risk	4,099	(1) LDCT (annual) (2) LDCT (biennial)	Usual care
	NELSON	Belgium and Netherlands	2003–2006	High risk	15,5792 (13,195 men)	LDCT	Usual care
	NLST	United States	2002–2004	High risk	53,454	LDCT	CXR
	PLCO (lung)	United States	1993–2001	General	154,887	CXR	Usual care
	UKLS	United Kingdom	2011–2013	High risk	4,055	LDCT	Usual care
NPC	China NPC	China	2009–2014^b^	General	122,074	EBV serology + indirect mirror + lymphatic palpation	Usual care
Oral	Trivandrum	India	1995–2004	General	191,873	Visual inspection	Usual care
Ovarian	PLCO (ovarian)	United States	1993–2001	General	78,215	CA125 ± TVU	Usual care
	UK Pilot	United Kingdom	1989	General - volunteered for a previous prevalence screen	21,935	CA125 (+ TVU as 2nd tier)	Usual care
	UKCTOCS	United Kingdom	2001–2005	General	202,638	(1) CA125 (+ TVU as 2nd tier) (2) TVU	Usual care
Prostate	CAP	United Kingdom	2001–2009	General	415,537	Blood PSA	Usual care
	ERSPC	Belgium, Finland, France, Italy, Netherlands, Portugal, Spain, Sweden, and Switzerland	1991–2005, depending on country	General	266,512 (162,243 aged 55–69 years, excluding France and Portugal)	Blood PSA ± DRE ± TRUS (depending on country)	Usual care
	ERSPC Pilot 1	Netherlands	1991–1992	Low risk (PSA <10.0 ng/mL at baseline)	1,134	Blood PSA	Usual care
	Norrkoping	Sweden	1987	General	9,026	DRE only first, later DRE + blood PSA	Usual care
	PLCO (prostate)	United States	1993–2001	General	76,683	Blood PSA + DRE	Usual care
Multiple^c^	D’Aquapendente	Italy	NR (likely 2010s)	High risk	195	Torso CT + FOBT	Usual care
	MVTEP	France	2009–2012	High risk	399	Limited screening + PET-CT	Usual care
	SOMIT	Italy	1993–1997	High risk	201	Extensive screening including torso CT	Usual care

Abbreviations: AFP, α-fetoprotein; ALT, alanine aminotransferase; BSE, breast self-examination; CA125, cancer antigen 125; CBE, clinical breast examination; CXR; chest X-ray; DRE, digital rectal examination; EBV, Epstein–Barr virus; FOBT, fecal occult blood test; FS, flexible sigmoidoscopy; HPV, human papillomavirus; LDCT, low-dose CT; NPC, nasopharyngeal cancer; NR, not reported; TRUS, transrectal ultrasound; TVU, transvaginal ultrasound; US, ultrasound; VIA, visual inspection with acetic acid; WHO, World Health Organization.

Trial name abbreviations: CAP, Cluster Randomized Trial of PSA Testing for Prostate Cancer; CNBSS, Canadian National Breast Screening Study; DANTE, Detection and Screening of Early Lung Cancer by Novel Imaging Technology and Molecular Essays Trial; DLCST, Danish Lung Cancer Screening Trial; ERSPC, European Randomized Study of Screening for Prostate Cancer; HIP NY, Health Insurance Plan of Greater New York; ITALUNG, Italian Lung Cancer Screening Trial; LSS, Lung Screening Study; LUSI, German Lung Cancer Screening Intervention Trial; MILD, Multicentric Italian Lung Detection; MLP, Mayo Lung Project; MSK; Memorial Sloan Kettering Lung Study; MVTEP, Standard Diagnostic Procedures With or Without Fludeoxyglucose F 18 Positron Emission Tomography in Finding Cancer in Patients With a Blood Clot in a Vein; NELSON, Nederlands–Leuvens Longkanker Screenings Onderzoek; NLST, National Lung Screening Trial; NORCCAP, Norwegian colorectal cancer prevention; NordICC, Nordic-European Initiative on Colorectal Cancer; PLCO, Prostate, Lung, Colorectal, and Ovarian Cancer Screening Trial; SCORE, Screening for COlon Rectum trial; SOMIT, Subsequent diagnosis Of Malignancy in patients presenting with Idiopathic venous Thromboembolism; UKCTOS, UK Collaborative Trial of Ovarian Cancer Screening; UKFSST, UK Flexible Sigmoidoscopy Screening Trial; UKLS, UK Lung Cancer Screening Trial.

a Later extended to 2014.

b Interim analysis of the Zhongshan cohort recruited from 2009 to 2014. The whole trial included three towns in Zhongshan City and 13 towns in Sihui City, recruiting people from 2008 to 2015.

c Different target cancers screened for in each trial. High-risk population was defined as patients with a recent diagnosis of venous thrombosis ± pulmonary embolism.

### Data extraction

The relevant data were extracted into a piloted electronic data collection form by one researcher and checked by a second (J. Geppert, M. Rebolj, N. Kouppa, C. Stinton, M.J. Randell, K. Freeman, and B. Shinkins). For consistency, the data for most trials were extracted by the same researcher (J. Geppert); for the remaining trials, J. Geppert performed the checks. Disagreements were resolved by a third reviewer (A.R. Brentnall, M. Rebolj, and S. Taylor-Phillips). The extracted information included general information on the trial design and methods such as the eligibility criteria, study flow, population, intervention, and comparator and publication-specific statistical methods and results on the reported mortality and surrogate outcomes.

### Outcomes

Our protocol included an extraction of the following outcomes: (i) cancer-specific mortality, (ii) the incidence of late-stage cancer, and (iii) the proportion of cancers diagnosed at a late stage. Numerators of (ii) and (iii) included the cumulative numbers of late-stage target cancer diagnoses since randomization, by arm, up to the timepoint of the reporting by trialists. For the incidence of late-stage cancer, the denominator included person-years of follow-up in randomized individuals, by arm, up to the time of the reporting; if this was not available, we substituted it with the number of randomized individuals by arm. For the proportion of cancers diagnosed at a late stage, the denominator included the cumulative number of target cancers diagnosed at any stage, including cancers with unknown stage, up to the same timepoint since randomization.

For (i) and (ii), the endpoints extracted were the unadjusted rate ratio, relative risk (RR), or hazard ratio estimates comparing the intervention versus the control arm and their 95% confidence intervals (CI) as reported in trial publications if available; otherwise, we used the extracted aggregated data and calculated rate ratios where person-years were available, or risk ratios if only numbers randomized were available, with Wald-type 95% CI. For (iii), we used the extracted aggregate data to estimate the relative proportions.

### Definition of late-stage cancer

There is no standardized definition of late-stage cancer across cancer types or across the periods of time in which screening trials have been run. Therefore, we defined it using a variety of nomenclatures. The definition chosen for each trial was based on what was reported and (subjective) judgment on the most appropriate definition based on the difference in expected prognosis and frequency between early and late stages. Late-stage definitions of cancer included (i) stage II+, stage IIB+, stage III+, or stage IV using a variety of staging systems; (ii) T4 (tumor invades adjacent structures) and/or N ≥ 1 (describing the number of nodes that contain cancer) and/or M1 (cancer has spread to other parts of the body) in the tumor–node–metastasis classification of malignant tumors; (iii) cancer-specific alternatives such as Dukes stage C or D for bowel cancer; or (iv) close cancer-specific approximations such as node positivity for breast cancer or commonly used alternative prognostic factors such as Gleason score 8+ for prostate cancer. Where the trialists did not categorize cancers into late and early stages, we attempted to follow a consistent definition across trials and cancer types, but this was not always possible. In particular, for one bowel and one cervical screening trial, data on cancer diagnoses were not provided by stage or another prognostic factor. However, screening tests in both trials targeted (early) preinvasive lesions with the aim of preventing invasive cancer. Therefore, we defined late-stage cancer as any invasive (bowel or cervical, respectively) cancer as screening with these tests is expected to reduce the cancer-specific incidence. The final definitions of late-stage cancer by trial, as used in our primary analyses, are given in Table 2.

**Table 2.** Summary of the “primary” late-stage and “main” mortality outcome estimates in the 57 trials.

Cancer type	Trial acronym or name	Definition of late stage	Timing of surrogate (years since entry)	Timing of mortality (years since entry)	Relative late-stage cancer incidence, RR (95% CI)	Relative proportion of late-stage cancer, RR (95% CI)	Cancer-specific mortality, RR (95% CI)	Weights in the main analysis
Bowel	Finnish	N ≥ 1	Range, 0–7	Md 4.5, range 0.0–8.3	1.07 (0.90–1.27)^a^	0.89 (0.78–1.01)^a^	1.04 (0.84–1.28)^b^	1.99
	Funen	Dukes C or distant spread^c^	Me 9.1	Me 9.1	0.84 (0.70–1.01)	0.84 (0.74–0.96)	0.82 (0.68–0.99)	2.41
	Gothenburg	Dukes D	Me 15.5	Me 15.5	0.95 (0.76–1.18)	0.99 (0.81–1.20)	0.84 (0.71–0.99)	3.17
	Minnesota	Dukes D	Me 11.8, max 13	Me 11.8, max 13	0.50 (0.33–0.76) (annual vs. UC)	0.57 (0.38–0.84) (annual vs. UC)	0.67 (0.51–0.89) (annual vs. UC)	Annual: 1.09 Biennial: 1.33
					0.62 (0.42–0.92) (biennial vs. UC)	0.68 (0.47–0.97) (biennial vs. UC)	0.96 (0.74–1.23) (biennial vs. UC)	
	NORCCAP	Dukes C or distant spread	Md 7, max 8	Md 15	0.90 (0.70–1.15)^d^	0.88 (0.76–1.02)^d^	0.82 (0.66–1.02)^d^	1.80
	NordICC	Dukes C or D	Md 10.0	Md 10.0	0.80 (0.65–1.00)	0.97 (0.82–1.14)	0.91 (0.64–1.16)	1.33
	Nottingham	Dukes C or D	Md 7.8	Md 7.8	0.91 (0.80–1.04)	0.88 (0.80–0.97)	0.85 (0.74–0.98)	4.23
	PLCO (bowel)	Stage III+	Md 11.9, max 13.0	Md 12.1, max 13.0	0.71 (0.62–0.81)	0.90 (0.81–1.00)	0.74 (0.63–0.87)	3.27
	SCORE	Stage III+	Md 10.5	Md 11.4	0.73 (0.57–0.94)	0.90 (0.75–1.07)	0.78 (0.56–1.08)	0.81
	Telemark	Dukes C or D	Up to 11	Up to 13	0.50 (0.05–5.48)	1.00 (0.18–5.46)	0.33 (0.03–3.18)	0.02
	UKFSST	Invasive cancer^e^	Me 10.8	Md 11.2	0.77 (0.70–0.84)	No data on stage	0.68 (0.59–0.80)	3.24
Breast	CNBSS-1	N ≥ 1	Max 7	Max 7	1.55 (1.13–2.11)	1.27 (0.97–1.66)	1.36 (0.84–2.21)	0.36
	CNBSS-2	N ≥ 1	Max 7	Max 7	1.09 (0.82–1.45)	0.94 (0.74–1.20)	0.97 (0.62–1.52)	0.42
	Edinburgh	Stage III+	Me 6.8	Me 6.8	0.63 (0.46–0.87)^f^	0.46 (0.35–0.61)^f^	0.84 (0.60–1.16)^f^	0.81
	Gothenburg	N ≥ 1	Me 6 (screening period)	Max 14; cancers diagnosed in screening period	0.80 (0.61–1.05)	0.89 (0.71–1.10)	0.78 (0.57–1.06)	0.90
	HIP NY	N ≥ 1	5	10	0.84 (0.65–1.10)	0.80 (0.65–0.99)	0.78 (0.63–0.96)	1.86
	Malmö	Stage II+	Me 8.8	Me 8.8	0.83 (0.68–1.00)	0.67 (0.58–0.76)	0.96 (0.68–1.35)	0.74
	Mumbai	Stage III+	Md 18	Md 18	0.81 (0.68–0.97)	0.83 (0.72–0.96)	0.85 (0.70–1.02)	2.57
	Russia/WHO	N ≥ 1	Up to 10	Up to 13; cancers diagnosed up to 1994	1.20 (1.01–1.43)	0.97 (0.86–1.09)	1.07 (0.86–1.34)	1.79
	Shanghai	N ≥ 1	Me 10	Me 10	0.92 (0.79–1.07)	0.95 (0.84–1.08)	1.04 (0.82–1.33)	1.42
	Stockholm	Stage II+	Up to 6	Me 12, cancers diagnosed 1981–1986	0.88 (0.68–1.12)	0.93 (0.78–1.12)	0.74 (0.50–1.10)	0.54
	Trivandrum	Stage III+	Me 12	Me 12	1.18 (0.92–1.52)	0.90 (0.74–1.10)	1.03 (0.75–1.41)	0.85
	Two-County	Stage II+	Me 6	Me 6	0.79 (0.69–0.91)^g^	0.61 (0.55–0.67)^g^	0.72 (0.54–0.98)^g^	0.96
	UK Age	N ≥ 1	Me 10.6	Md 17.7, cancers diagnosed in screening period	0.90 (0.78–1.05)	0.89 (0.79–1.00)	0.88 (0.74–1.04)	3.07
Cervical	Finnish	Invasive cancer^e^	Me 6.3	Me 6.3	1.00 (0.76–1.29)	No data on stage	1.11 (0.62–1.92)	0.29
	Mumbai	Stage ≥IIB+	Me 8.0, max 12	Me 8.0, max 12	0.84 (0.63–1.12)	0.86 (0.72–1.04)	0.69 (0.50–0.94)	0.89
	Osmanabad	Stage II+	Me 8	Me 8	0.97 (0.72–1.32) (VIA vs. UC) 0.70 (0.50–0.98) (cytology vs. UC) 0.44 (0.30–0.64) (HPV vs. UC)	0.79 (0.65–0.95) (VIA vs. UC) 0.55 (0.43–0.69) (cytology vs. UC) 0.44 (0.33–0.59) (HPV vs. UC)	0.81 (0.57–1.16) (VIA vs. UC) 0.83 (0.58–1.20) (cytology vs. UC) 0.49 (0.32–0.74) (HPV vs. UC)	VIA: 0.67 cytology: 0.65 HPV: 0.50
	Tamil Nadu	Stage II+	Me 6, max 7	Me 6, max 7	0.70 (0.53–0.92)	1.01 (0.86–1.20)	0.59 (0.44–0.79)	0.97
Liver	Qidong	Stage III^h^	Me 5.2	Me 5.2	0.54 (0.37–0.81)	0.48 (0.35–0.67)	1.01 (0.81–1.29)	1.62
	Shanghai	Stage III^h^	Me 4.1–4.3	Me 4.1–4.3	0.53 (0.32–0.88)	0.41 (0.27–0.61)	0.63 (0.41–0.98)	0.44
Lung	Czech Study	Stage III^i^	6	15	1.15 (0.78–1.71)	0.87 (0.67–1.15)	1.14 (0.96–1.36)	2.77
	DANTE	Stage II+	Md 8.35	Md 8.35	0.94 (0.64–1.38)	0.69 (0.54–0.89)	1.00 (0.69–1.44)	0.63
	DLCST	Stage III+	Md 9.8	Md 9.8	1.12 (0.74–1.70)	0.59 (0.46–0.77)	1.03 (0.66–1.61)	0.43
	ITALUNG	Stage III+	Md 8.5	Md 9.3	0.76 (0.48–1.19)	0.81 (0.60–1.11)	0.70 (0.47–1.03)	0.57
	Johns Hopkins	Stage II+	Me 7.2, max 9	Me 7.2, max 9	0.86 (0.68–1.09)	0.91 (0.77–1.06)	0.83 (0.67–1.04)	1.68
	LSS	Stage III+	1	Md 5.2	1.78 (0.79–4.01)	0.89 (0.48–1.64)	1.24 (0.74–2.08)	0.32
	LUSI	Stage II+	7	Md 8.9, max 11	0.55 (0.34–0.88)	0.39 (0.29–0.54)	0.72 (0.45–1.16)	0.38
	MLP	Stage III+	Me 9 (range, 7.5–11.0)	Me 9 (range, 7.5–11.0)	0.98 (0.75–1.27)	0.76 (0.64–0.90)	1.06 (0.82–1.36)	1.35
	MSK	Stage II+	Me 7.2, max 9	Me 7.2, max 9	1.01 (0.77–1.33)	0.98 (0.82–1.18)	0.95 (0.73–1.25)	1.14
	MILD	Stage II+	Me 9.4–9.6	Me 9.7	0.74 (0.50–1.10) (LDCT combined vs. UC)	0.64 (0.50–0.81) (LDCT combined vs. UC)	0.70 (0.45–1.09) (LDCT combined vs. UC)	0.45
	NELSON	Stage III+	Me 9.5, max 10	Me 9.5, max 10	0.71 (0.58–0.87)^j^	0.63 (0.55–0.72)^j^	0.76 (0.61–0.94)^j^	1.90
	NLST	Stage III+	Md 6.5	Md 6.5	0.79 (0.70–0.89)	0.70 (0.64–0.77)	0.84 (0.75–0.95)	5.66
	PLCO (lung)	Stage III+	Incidence: 7 (Me 6.7) Proportion: Md 11.9, max 13	Md 11.9, max 13	0.94 (0.84–1.05)	0.92 (0.87–0.98)	0.99 (0.91–1.07)	15.47
	UKLS	Stage III+	4	Md 7.3	0.44 (0.23–0.87)	0.33 (0.19–0.57)	0.65 (0.41–1.02)	0.42
NPC	China NPC	Stage III+	Md 6.0	Md 6.0	0.78 (0.57–1.07)	0.70 (0.58–0.85)	0.81 (0.45–1.45)	0.25
Oral	Trivandrum	Stage III+	Me 4.4–4.9, max 9	Me 4.4–4.9, max 9	0.89 (0.68–1.16)	0.76 (0.64–0.91)	0.79 (0.51–1.22)	0.45
Ovarian	PLCO (ovarian)	Stage III+	Md 12.4, max 13	Md 12.4, max 13	1.20 (0.96–1.51)	0.99 (0.89–1.10)	1.18 (0.91–1.54)	1.21
	UK Pilot	Stage III+	Max 8 (1990–1997)	Max 8 (1990–1997)	0.61 (0.29–1.30)	0.76 (0.53–1.10)	0.50 (0.19–1.28)	0.10
	UKCTOCS	Stage III+	Me 10.8	Md 11.1	0.83 (0.70–0.99) (MMS vs. UC) 0.91 (0.77–1.08) (TVU vs. UC)	0.77 (0.69–0.87) (MMS vs. UC) 0.91 (0.82–1.10) TVU vs. UC)	0.85 (0.70–1.03) (MMS vs. UC) 0.89 (0.73–1.07) (TVU vs. UC)	MMS: 2.31 TVU: 2.38
Prostate	CAP	Gleason 8–10	Max 6	Md 10, max 15	1.00 (0.90–1.11)	0.63 (0.57–0.70)	0.96 (0.86–1.08)	6.62
	ERSPC	Gleason 8–10	Md 11.0	Me 10.5	0.82 (0.74–0.91)^k^	0.52 (0.47–0.57)^k^	0.79 (0.68–0.91)^k^	4.05
	ERSPC Pilot 1	Gleason ≥3 +4	Md 19	Md 19	0.86 (0.50–1.48)	0.66 (0.43–1.01)	0.48 (0.17–1.36)	0.08
	Norrköping	T3-4, N1, or MX/M1	Max 13	Max 22, cancers diagnosed up to 1999	0.87 (0.62–1.23)	0.59 (0.46–0.76)	1.16 (0.78–1.73)	0.54
	PLCO (prostate)	Gleason 8–10	Max 10	Me 11.1, max 13	0.85 (0.73–0.99)	0.73 (0.63–0.85)	1.09 (0.87–1.36)	1.75
Multiple^l^	D’Aquapendente	Stage IV	2	2	0.74 (0.17–3.23)	0.62 (0.18–2.16)	0.49 (0.09–2.64)	0.03
	MVTEP	“Advanced”	2	2	0.71 (0.23–2.21)	0.77 (0.33–1.79)	0.40 (0.08–2.04)	0.03
	SOMIT	N1 and/or M1	2	2	0.64 (0.22–1.90)	0.45 (0.21–0.96)	0.52 (0.10–2.75)	0.03

Abbreviations: HPV, human papillomavirus; LDCT, low-dose CT; M1, distant metastasis present; Max, maximum follow-up time; Me, mean follow-up time; Md, median follow-up time; MMS, multimodal screening; MX, metastasis cannot be measured; N1, cancer has spread to nearby lymph nodes; N1, N2, N3, number of nearby lymph nodes that have cancer; NPC, nasopharyngeal cancer; RR, relative risk or rate ratio; T3, tumor size or area: cancer has broken through the capsule (covering) of the prostate gland; T4, tumor size or area: cancer has spread into other body organs nearby; TVU, transvaginal ultrasound; UC, usual care; VIA, visual inspection with acetic acid; WHO, World Health Organization.

a 321,311 individuals randomized from 2004 to 2011.

b 362,165 individuals randomized from 2004 to 2012.

c “Advanced” colorectal cancer was defined as stage C, distant spread, and no classification.

d 55,736 men and women aged 55–64 years.

e No stage data reported. For screening tests that can prevent cancer, the incidence of invasive cancer was used as late-stage surrogate.

f 44,288 women in cohort 1.

g 134,867 women aged 40 to 74 years.

h Chinese hepatocellular cancer staging system.

i Clinical-diagnostic staging system with occult, I, II, and III stages.

j 13,195 men.

k 162,243 men aged 55 to 69 years (excluding France and Portugal).

l Different target cancers screened for in each trial.

### Trial-specific timepoints for late-stage outcomes

The number of relevant publications varied substantially by trial, ranging from a single report on the mortality and a late-stage cancer outcome to multiple reports with assessments at various timepoints since randomization. Where late-stage cancer outcomes were reported at several timepoints, we defined the “primary” available late-stage outcome as (i) one that was measured the closest to the midway point between the end of the intervention period and the “main” mortality timepoint (defined below). This is because late-stage outcomes measured after the conclusion of the intervention in a trial and before the “main” mortality outcome could be informative for early decision-making about a population-based rollout. If no such report was identified, then an estimate reported (ii) at the same time as the “main” mortality outcome was considered as the “primary” available late-stage outcome or else (iii) at the latest timepoint when the screening intervention was still ongoing or soon thereafter. Thus, the defined “primary” late-stage outcomes for each trial are reported in Table 2 and in Supplementary Methods and Tables, Section 3, Supplementary Tables S14 (relative incidence of late-stage diagnoses) and S15 (relative proportion of cancers diagnosed at late stage).

### Trial-specific “main” timepoints for cancer-specific mortality outcomes

Where cancer-specific mortality was reported at more than one timepoint, we defined the “main” mortality timepoint as one at which primary results were to be calculated by trialists in the original publications, in the trial protocol, or in the statistical analysis plan and the power calculations or if necessary, the only or the latest available timepoint. The “main” mortality endpoints are reported by trial in Table 2 and in Supplementary Methods and Tables, Section 3, Supplementary Tables S14 and S15.

### Statistical analysis

We evaluated the association between the screening effect on late-stage incidence and proportion cancer outcomes and cancer-specific mortality using a fixed-effects linear model of log RR estimates weighted by the inverse variance of the screening effect on mortality [rather than late-stage incidence, because we felt that precision on cancer-specific mortality better standardizes information at a trial level across different cancers than either surrogate examined, which are also affected by differing late-/early-stage incidence (in control) across cancer types]. We made no assumption on the position of the intercept in this regression model; a value close to the origin, however, would be indicative of no screening effect on mortality when there is no screening effect on the late-stage outcome. We calculated a weighted Pearson correlation coefficient and associated coefficient of determination (*R*^2^) when at least three trials were available; 95% CIs used a non-parametric empirical bootstrap (50,000 resamples), with trial as the resampling unit.

We evaluated heterogeneity in multiple ways. We explored the relationship between late-stage cancer and cancer-specific mortality by the type of screening test for cancers for which sufficient data were available. To study the impact of the timing of the reporting on these associations, we plotted estimates of the relative incidence of late-stage cancer against the “main” relative mortality estimate when the former were reported for at least two timepoints from the same trial (Supplementary Methods and Tables, Section 4, Supplementary Table S16). Subgroup analyses focused on trials in which the late-stage outcomes were measured before the “main” mortality endpoint but after the screening period had ended. Sensitivity analysis used stage III+ across all cancers. Sensitivity of correlation coefficients to individual trial (as a unit of observation) was assessed by leave-one-out validation, in which each trial was left out once and the weighted correlation was performed on the other trials.

Exploratory analysis included the use of a single combined outcome for each combination of cancer type and class of screening test. The combined outcomes and the associated *z*-statistics were obtained from separate fixed-effect meta-analysis for late-stage and mortality outcomes. The aim of this analysis was to reduce noise from the trial-level analysis and help understand potential surrogacy across classes of screening tests. Another exploratory analysis evaluated the forecast calibration of late-stage incidence effects for cancer-specific mortality effects. A (naïve) density forecast treated 95% CIs for log RR as the 2.5% and 97.5% quantiles from a normal distribution. Calibration of probability integral transform values (the cumulative distribution function value of observed mortality given the forecast density) was evaluated through an Anderson–Darling goodness-of-fit test (22).

The systematic search of the literature was operationalized and documented using EndNote version 20 (Clarivate). The data were extracted into Excel files (Microsoft). All statistical analyses were undertaken with R version 4.2.1 [packages “weights” (23) to calculate the weighted correlations, “metafor” (24) to calculate effect measures and CIs for rate ratios and RRs from aggregate data when these were not reported by trialists, and “boot” (25) to calculate bootstrap CIs for summary statistics].

### Data availability

All data analyzed in this report are available on GitHub at https://github.com/brentnall/sums

## Results

### Literature review

#### Study selection

For “Search 1,” we screened 3,154 unique records identified by either of the three bibliographical databases (Fig. 1; Supplementary Methods and Tables, Section 2). Of those, we assessed 271 full texts for eligibility. Another 188 full texts were assessed after they had been identified from supplementary sources. In total, we identified 60 cancer screening trials that reported mortality outcomes for all arms. The trial-specific searches in bibliographical databases during “Search 2” identified 4,249 records that required screening; of these, we assessed 555 full texts for eligibility. Another 16 full texts were identified from supplementary sources and assessed for eligibility. Altogether, “Search 1” and “Search 2” combined identified 57 trials including 61 trial arm comparisons that reported both the mortality outcome and a late-stage outcome covered in 212 publications. All references for the included original trial publications are available in Supplementary Methods and Tables, Section 3.

#### Study characteristics

Fifty-four trials evaluated screening tests for nine individual cancer types (Table 1). An additional three trials offered screening for multiple cancer types combined to a well-defined high-risk patient group (unprovoked venous thrombosis with or without pulmonary embolism), using a variety of methods. Overall, trials undertaken in average-risk individuals were larger than trials recruiting from high-risk populations. We noted a wide variation between the trials in factors such as epochs and settings in which they were undertaken, inclusion criteria, design, or screening tests (Supplementary Methods and Tables, Section 3; Supplementary Tables S12 and S13). Likewise, the definition of late-stage cancer depended on the cancer type and trial as did the timing (counted since randomization) of the reporting of the outcomes (Table 2; Supplementary Methods and Tables, Section 3, Supplementary Tables S14 and S15). Almost all trials reported their data at multiple timepoints (for late-stage cancer, see Supplementary Methods and Tables, Section 4, Supplementary Table S16).

### Meta-analysis: primary analysis

#### Trial-level surrogacy meta-analysis: relative incidence of late-stage cancer between trial arms

**Figure 2.**
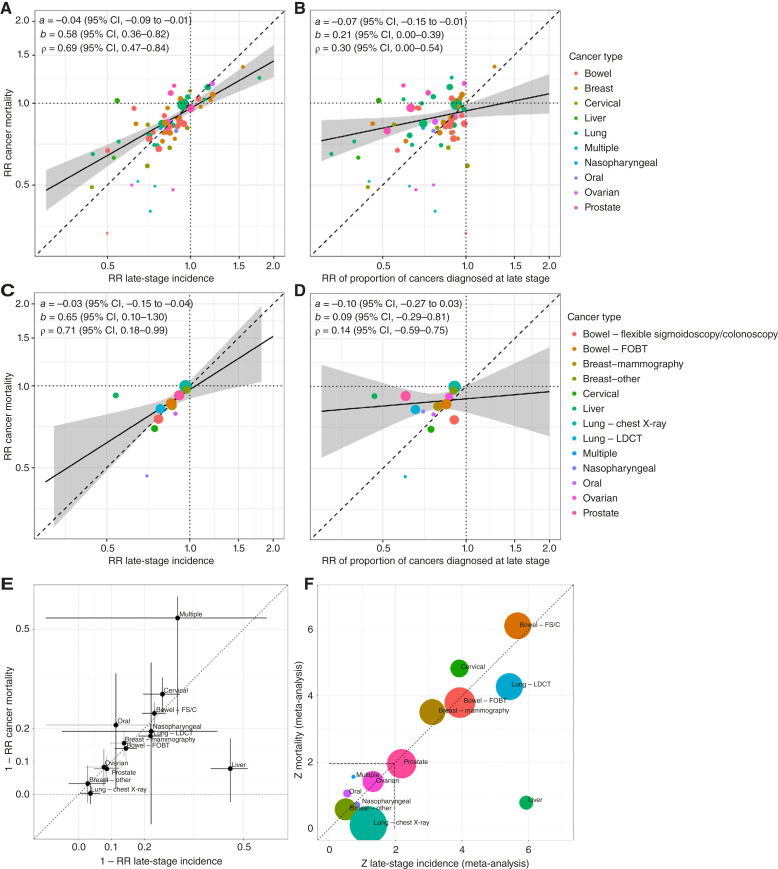
Bubble plots to evaluate the strength of association between the trial-level effect of screening on cancer mortality and the effect on either incidence of late-stage cancer or the proportion of cancers that are late stage across all trials. These are the primary results from the meta-analysis. **A,** Incidence of late-stage cancer. **B,** Proportion of cancers diagnosed at late stage. Each data point in **A** and **B** is a trial arm comparison. **C,** Incidence of late-stage cancer. **D,** Proportion of cancers diagnosed at late stage. Each data point in **C** and **D** is from a class of screening test. The same type of plot is used for **A–D**. **E,** Incidence of late-stage cancer. The scales (1-RR) from **C** are inverted and SEs are added to better show uncertainty. **F,** Associated *z*-statistics from **E**. Each data point in **E** and **F** is from a class of screening test. In all plots, the bubble sizes are proportional to the inverse variance of the screening effect on the mortality outcome. a, the intercept from the regression analysis; b, the regression coefficient from the regression analysis; ρ, the estimated correlation coefficients. Dotted lines: axes of no screening effect on late-stage and mortality outcomes. Dashed line: diagonal of equal screening effect on late-stage and mortality outcomes. FOBT, fecal occult blood test; FS/C, flexible sigmoidoscopy/colonoscopy; LDCT, low-dose CT.

**Figure 3.**
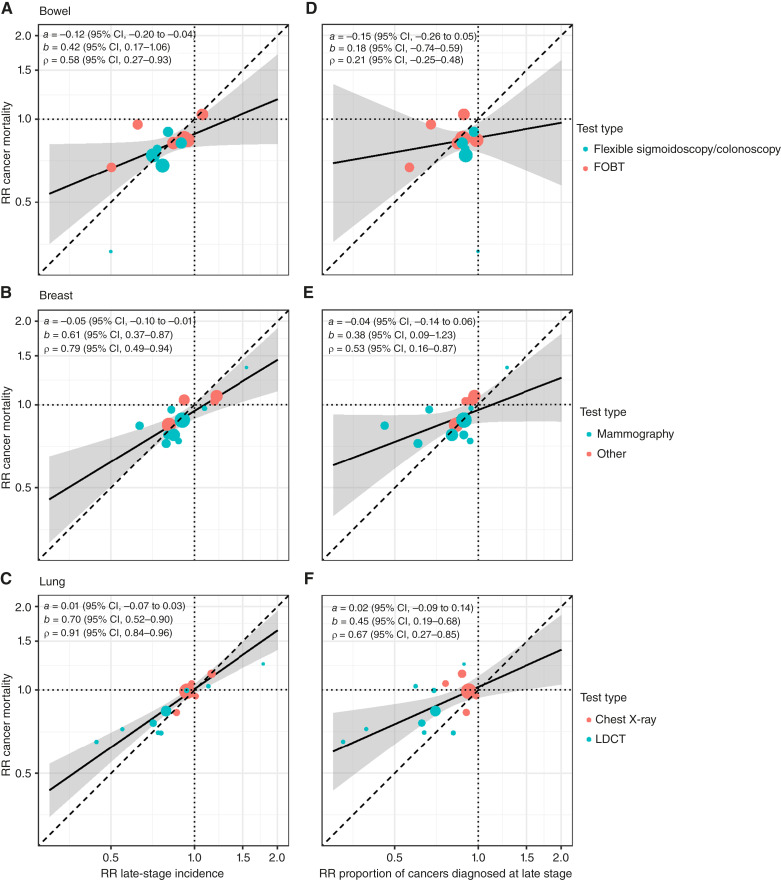
Bubble plots to evaluate the strength of association between the trial-level effect of screening on cancer mortality and the effect on either incidence of late-stage cancer or the proportion of cancers that are late stage, separately for bowel, breast, and lung cancer screening trials. The figure provides more details on the associations between the screening effect on mortality and the screening effect on the late-stage incidence for cancers with the largest numbers of trials and additionally stratifies by the type of the screening test within each cancer type. Plots **A–C** are for incidence of late-stage cancer and **D–F** for proportion of cancers that are late stage. a, the intercept from the regression analysis; b, the regression coefficient from the regression analysis; ρ, the estimated correlation coefficients. FOBT, fecal occult blood test; LDCT, low-dose CT.

Our primary analysis was of trial-level surrogacy using data from 57 trials. Only 14 of 57 trials reported 15 trial arm comparisons with late-stage incidence at an earlier timepoint than mortality but after the screening intervention had ended. Across the 57 trials, correlation between the relative incidence of late-stage cancer and relative mortality between trial arms was 0.69 (95% CI, 0.47–0.84; *R*^2^: 0.47; Fig. 2; Supplementary Methods and Tables, Section 4, Supplementary Table S17) for all cancers combined. The point estimate was unchanged in an unweighted analysis and when we excluded the two trials in which the late-stage outcome was defined as invasive cancer. The intercept was close to the origin (log RR: −0.04; 95% CI, −0.09 to −0.01). These values differed somewhat between cancer types. For cancer types with the largest number of trials, the correlation was 0.58 (95% CI, 0.27–0.93; *R*^2^: 0.34) for bowel cancer (*N* = 11), 0.79 (95% CI, 0.49–0.94; *R*^2^: 0.62) for breast cancer (*N* = 13), and 0.91 (95% CI, 0.84–0.96; *R*^2^: 0.83) for lung cancer (*N* = 14; Fig. 3A–C). For these three cancers, Fig. 3 also categorizes each trial depending on the type of the screening test, and clustering by test type can be observed. Detailed results for all cancer types with at least three trials are presented in Supplementary Methods and Tables, Section 4, Supplementary Table S17 and in Supplementary Figure S1.

**Figure 4.**
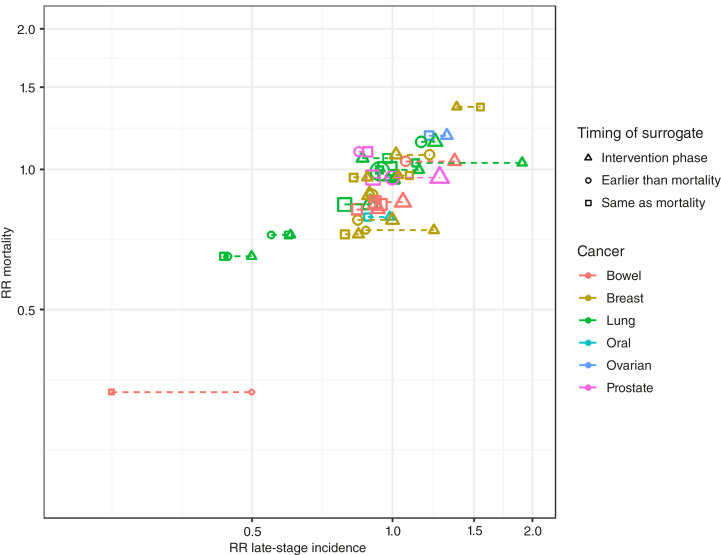
Exploratory analysis of the effect of the timing of the reporting of late-stage incidence on the association with the mortality outcome. The figure includes trials that reported their late-stage outcomes at least twice [at intervention phase (triangles), earlier than mortality (circles), and/or at mortality (squares)]. Each trial is then represented by two or up to three points connected by a straight line drawn at the main estimate of the screening effect on cancer-specific mortality; this line shows how the estimates of the screening effect on the late-stage incidence within a specific trial changed over time. Size of the points corresponds to precision of the mortality result (inverse variance of the estimate). Colors denote types of cancer (please see the legend).

The estimate of the screening effect on the late-stage incidence changed depending on the time of the reporting (Fig. 4; Supplementary Methods and Tables, Section 4, Supplementary Table S16; and Supplementary Figure S2). In most examples, the relative incidence of late-stage cancer (screening arm relative to comparator arm) decreased between the end of the intervention phase and the timepoint at which the mortality endpoint was measured.

#### Trial-level surrogacy meta-analysis: relative proportion of cancers diagnosed at late stage

Data for the relative proportion of cancers diagnosed at late stage were available from 55 trials. As expected, correlation between this late-stage outcome and the “main” relative mortality outcome was weaker than for the relative incidence of late-stage diagnoses. The estimate of the correlation coefficient for all cancers combined was 0.30 (95% CI, 0.00–0.54; *R*^2^: 0.09; Fig. 2B; Supplementary Methods and Tables, Section 4, Supplementary Table S18). Also here, the intercept was close to the origin (log RR: −0.07; 95% CI, −0.15 to −0.01). For bowel cancer (*N* = 10), the correlation was 0.21 (95% CI, −0.25 to 0.48; *R*^2^: 0.05), for breast cancer (*N* = 13) 0.53 (95% CI, 0.16–0.87; *R*^2^: 0.28), and for lung cancer (*N* = 14) 0.67 (95% CI, 0.27–0.85; *R*^2^: 0.44; Fig. 3D–F). Detailed results for all cancer types with at least three trials are presented in Supplementary Methods and Tables, Section 4, Supplementary Table S18 and in Supplementary Figure S3.

### Meta-analysis: sensitivity and subgroup analyses

Data were available for fewer trials for pre-planned sensitivity and subgroup analyses. However, for both late-stage outcomes, the observed patterns remained similar as in the primary analysis. For the relative incidence of late-stage cancer, the correlation was 0.74 (95% CI, 0.48–0.90; R^2^: 0.54), including only trial arm comparisons that reported late-stage outcomes before the main mortality outcome (*N* = 15), and 0.67 (95% CI, 0.33–0.89; R^2^: 0.45) when late-stage cancer was defined as stages III+ (*N* = 30; Supplementary Methods and Tables, Section 4, Supplementary Table S17). In leave-one-out analysis, the weighted correlation coefficient across all trials ranged between 0.66 and 0.78 with an IQR of 0.68 to 0.69 (results not tabulated). The estimate was very sensitive to the Qidong liver trial results (exclusion of this trial led to correlation 0.78, 95% CI, 0.69–0.85).

### Exploratory analyses

#### Association between the effect of screening on late-stage cancer incidence and on mortality at screening test level

Across all 57 included trials, there were on average 12% fewer target cancer deaths (95% CI, 9%–14%) in those randomized to a screening intervention compared with a control group. For comparison, there was a mean 13% reduction (95% CI, 11%–16%) in the late-stage cancer incidence (57 trials) and a mean 21% (95% CI, 19%–24%) reduction in the proportion of cancers diagnosed at late stage (55 trials).

Analysis shown in Fig. 2C identified that the association between the screening effect on late-stage cancer incidence and on cancer-specific mortality overall, which was dominated by breast, colorectal, and lung cancer screening trials, was also observed across different classes of cancer screening tests. These associations were, as expected, weaker for the relative proportions of cancers diagnosed at a late stage (Fig. 2D). However, liver cancer again emerged as an outlier. The two included liver cancer screening trials (combined) found only a small and nonsignificant effect on liver cancer mortality but a significant and substantial effect on late-stage liver cancer incidence (Fig. 2E). This was driven by the Qidong trial. A similar pattern emerged from the analysis of the *z*-statistics associated with each meta-analysis (Fig. 2F). The plot shows that screening tests that have been implemented in national programs have strong meta-analytic evidence of benefit from both late-stage incidence and cancer-specific mortality endpoints (e.g., mammography for breast cancer and low-dose CT for lung cancer screening). Some of those tests that have not been recommended have strong evidence of lack of substantial benefit on both cancer incidence and mortality endpoints (e.g., cancer antigen 125 for ovarian cancer and chest X-ray for lung cancer screening). Thus, late-stage cancer incidence would have provided similar inference across several classes of cancer screening test trialed to date, except for liver cancer screening.

#### Using the 95% CI of the effect on the late-stage incidence as a forecast to predict the effect on cancer-specific mortality in a trial

**Figure 5.**
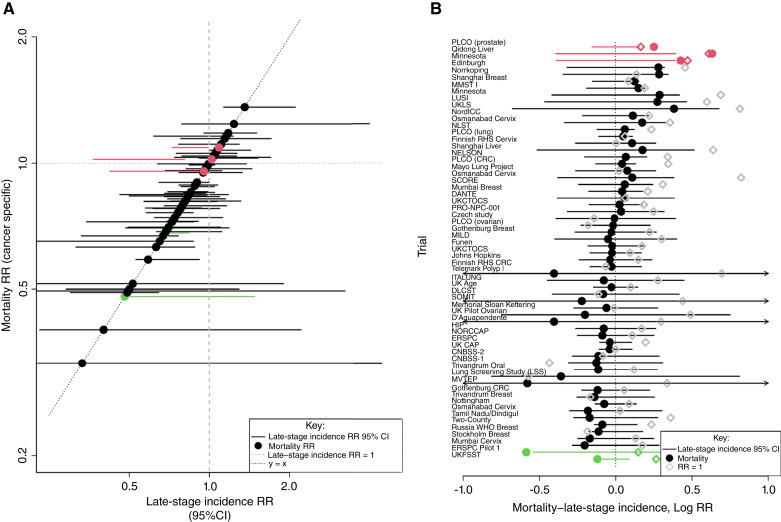
Exploratory evaluation of the utility of the 95% CI RR for late-stage incidence as a “forecast” of the final trial result for cancer-specific mortality. **A,** The observed RR for mortality (point estimates) versus the “predicted” 95% CI for the RR based on the observed incidence of late-stage cancer in the intervention versus the control arm. **B,** The data from **A** are transformed so that observed – predicted mortality is shown by trial, ordered by where on the prediction the observed mortality fell (mortality at top end of range at top and bottom end of range at bottom of the plot). Estimates marked red are from trials in which the observed mortality exceeds predicted range based on the late-stage outcome; those marked green are trials in which the observed mortality is lower than predicted; and observed mortality RR was included within the 95% CI range of the late-stage outcome for all the other trials shown in black. Wide confidence intervals are due to inclusion of smaller trials.

Figure 5 shows that the 95% CI of the RR for the incidence of late-stage cancer included the observed cancer-specific mortality RR (at the “main” timepoint) in 56 of 61 (92%) of trial comparisons; mortality was greater than the 95% CI range in three of 61 trial comparisons and less than the 95% CI range in two of 61 trial comparisons. This pattern was observed across all forecast intervals, and forecast calibration could not be rejected (*P* = 0.35). However, we also note that CIs were wide in several trials because of small sample size, limiting information. Among these, when the 95% CI of the RR for the incidence of late-stage cancer was less than 1 (suggesting benefit of the intervention), it included the observed mortality RR in 16 of 19 (84%) of trial comparisons. The clearest “outlier” from the five trials (with mortality observed outside the 95% CI from late-stage incidence) was the Qidong liver trial. With regard to the other four trials, the mortality outcome in the prostate part of the US Prostate, Lung, Colorectal, and Ovarian Cancer Screening trial was within the 95% CI range with extended follow-up (26); the Minnesota bowel screening trial (biennial fecal occult blood test) would have been within the range had we chosen Dukes stage C + D as the surrogate (not Dukes D only); in the European Randomised Study of Screening for Prostate Cancer trial pilot, we used Gleason 7+ rather than our preferred 8+ because it was not reported; and the UK Flexible Sigmoidoscopy Screening Trial showed a larger effect on mortality than predicted based on the 95% CI range for incidence, as might be anticipated for screening tests that also prevent cancer (not tabulated). Similar results were observed in the subgroup of 15 trial arm comparisons, with late-stage incidence reported at our ideal “midpoint” timepoint (Supplementary Methods and Tables, Section 4; and Supplementary Figure S4).

## Discussion

### Principal findings

Data from 57 randomized controlled trials in cancer screening showed a clear trial-level association between the screening effects on the incidence of late-stage cancer and the effects on cancer-specific mortality. Despite substantial differences between the trials in their designs and contexts in which they were undertaken, this overall association was observed across cancer types, particularly for cancer types for which most trials have been run (lung, breast, and bowel). Exploratory analysis suggested that for the majority of trials, the RR associated with cancer mortality was within the 95% CI of the RR for late-stage incidence. There was also consistency in evaluation of the utility of screening tests based on the surrogate or mortality in a screening test meta-analysis context. However, most trials did not report the incidence of late-stage cancer in advance of mortality. Furthermore, liver cancer trials were an outlier in which the incidence of late-stage cancer was a poor surrogate for mortality because (in these trials) there was not enough difference in survival by stage at detection and even stage I cancers had abysmal survival.

We did not find evidence to support the use of the relative proportion of cancers diagnosed at a late stage as a surrogate for cancer mortality. This may be partly due to susceptibility to cancers detected early which may progress very slowly; a high number of such cancers in the denominator would reduce the proportion of cancers that are late stage even though their treatment would not improve cancer-specific mortality within a typical observation period. Furthermore, the relative proportion of cancers diagnosed at a late stage does not respect randomization since the denominator is a post-randomization event. We and others (16) therefore do not recommend using this measure as a trial endpoint (neither primary nor secondary) in cancer screening trials.

### Strengths and weaknesses of the study

A key strength of our study is that we worked according to a pre-published protocol and a prespecified statistical analysis plan, and our study is the most comprehensive systematic review in this area (Supplementary Methods and Tables, Section 1). However, our analysis shares several limitations with all previous meta-analyses (17–20). As far as outcome surrogacy assessment is concerned, the quality of the reported data varied between trials: only 14 of 57 trials reported late-stage disease outcomes consistently and at our “primary” timepoint between the end of their intervention phases and the main mortality endpoint for which a validated surrogate would have the greatest utility for policy making; 42 of 57 trials did not report data on (differences in) the offered cancer treatment or treatment adherence by arm; and 40 of 57 trials either reported substantial proportions of individuals with missing data on the stage at diagnosis, causes of deaths, or differences therein between the arms or the outcomes or did not report enough detail to determine the completeness of the data on these items (Supplementary Methods and Tables, Section 5, Supplementary Table S19). These issues, compounded by the differences in trial designs, may have influenced the observed relationships between the late-stage and mortality outcomes. Finally, the main analysis focuses on correlation coefficients which have a number of weaknesses for evaluating surrogate endpoints. In particular, stronger correlation is expected for cancer types with a greater differential in survival between early and late stages, irrespective of how much of the screening effect is mediated by the surrogate (6).

### Comparison with the literature and policy implications

Two previous meta-analyses by Feng and colleagues (19) and Dai and colleagues (20) addressed the issue of outcome surrogacy but included fewer trials (39 and 33, respectively). Overall, estimated correlation between late-stage cancer incidence and cancer-specific mortality reported in the earlier publications is broadly consistent with our findings (Supplementary Methods and Tables, Section 1, Supplementary Table S1). This is unsurprising as many of the same trials feature in all analyses, and the number of screening trials conducted is modest; for a detailed description of the overlap between the three meta-analyses in terms of the included trials, see Supplementary Methods and Tables, Section 1, Supplementary Table S2. However, the two previous meta-analyses reached opposite conclusions about the suitability of trial outcome surrogacy, largely based on the data from the same trials. Some of this is likely to be due to subjective nature of data interpretation and not the data (or facts) themselves (27). But to an extent, differences in the conclusions are likely also related to review eligibility criteria, definition of late stage, differences in statistical methodology (e.g., weighted vs. unweighted analysis and methods for CIs), and choice of summary measures. In addition, there are differences in the choice of the timing of mortality effect, with Dai and colleagues choosing the latest timepoint possible, suggesting that (in general if not always) it is more likely to represent the true screening effect on mortality; Feng and colleagues used earlier mortality endpoints, but secondary analyses using later mortality results were more in agreement. They may also be related to the inclusion of smaller trials that cause considerable noise despite contributing to the totality of the evidence (Fig. 2) and the variation in the observed screening effects over time within each trial (Fig. 4). Interpretation in both studies focused on the point estimates of correlation coefficients and *R*^2^ values rather than their CIs.

One of the most visible differences between the available meta-analyses is the definition of cancer-specific late-stage outcomes. Feng and colleagues (19), for example, attempted to define late-stage cancer as stage III+ across all cancer types and concluded against using this as a surrogate in breast screening trials. We might have drawn the same conclusion based on our sensitivity analysis (Supplementary Methods and Tables, Section 4, Supplementary Table S17). In contrast, all other meta-analyses that covered breast cancer screening trials (17, 18, 20) and our primary analysis used broader definitions of late-stage breast cancer including stage II+ and its equivalents and provided considerably more supportive evidence of outcome correlations. Stage III+ only represents a small proportion of diagnosed cases and will be more common in non-attenders in the screening arm (11, 20), so the observed reductions in breast cancer mortality in trials (28) are likely to have, at least in part, been derived from a reduction in stage II cases. Similar examples showing the impact of the definition of late-stage diagnoses can be found for other cancers. In the context of screening for ovarian cancer, the trialists behind the UK Collaborative Trial of Ovarian Cancer Screening commented on the significant reduction in stage IV cases (*post hoc* analysis) and a nonsignificant reduction in mortality (primary endpoint) due to ovarian cancer and concluded that late stage is not a suitable surrogate endpoint in this case (29). However, all three multi-cancer meta-analyses considered stages III + IV as late-stage cancer, which seems to account for a considerable fraction of the observed mortality endpoints across the three ovarian screening trials included in the analyses.

These examples all reflect the lack of an established framework for the evaluation of outcome surrogacy in cancer screening and show that a concerted effort to further develop and optimize methodologic designs and validate a framework to appraise the risk of bias in the studies contributing the evidence would be beneficial (16). The development of both has been advanced for decades in the field of (cancer) treatment (30–34). However, in this setting, there are many more trials run for each class of treatment than are available for cancer screening. Therefore, it is likely that it will always be difficult to apply the methods from this field directly to cancer screening. Indeed, our review considered all cancer screening randomized controlled trials to report cancer-specific mortality since the 1960s and still included only 57 trials. In this context, making more efficient use of individual data from trials could be beneficial, including through widening access to individual-level trial data. Whether individual-level or aggregate-level data are undertaken, however, analyses will need to be tailored to the period within disease progression that offers the greatest opportunity to favorably affect screening outcomes as opposed to a common-stage endpoint across all cancers.

The topic of mortality outcome surrogacy in cancer screening has received renewed attention because of the emergence of new multi-cancer early detection (MCED) tests. In particular, a randomized controlled clinical trial of 140,000 participants is currently ongoing in England (NHS-Galleri trial, NCT05611632) to evaluate a cell-free DNA test (35). In this trial, the primary endpoint is a reduction in the number of cancers (of any type) diagnosed at stage III and IV. Most of the discussion in our article has been on screening tests designed to reduce mortality for individual cancer types and relates to a large extent to imaging tests which may detect a different stage distribution of cancer to MCEDs. Primary analysis in trials for MCEDs will use aggregate endpoints across multiple cancers. Our meta-analysis results do not rule out that late-stage cancer incidence might be considered as a surrogate outcome when combining multiple cancer types (Figs. 2, 3, and 5). However, there are caveats. The natural history of many cancers is complex with many subtypes of different prognosis and treatment effectiveness by stage, making it more challenging to predict when late-stage cancer is a sufficient surrogate for mortality. In our data, there were no sufficiently large trials of MCEDs, in particular using cell-free DNA technology. Biologically, positive screening results on cell-free DNA tests are expected to be associated with poorer cancer prognosis given the stage, and data support this (36). Therefore, there is a risk that a reduction in advanced cancer incidence does not translate into a reduction in mortality (37). Studies to evaluate cancer-specific survival by stage and MCED test positivity could provide insights into this issue, as well as trials in which modeling supports an evaluation of advanced stage and mortality reductions (38).

Lastly, the Chinese Qidong liver screening trial was an outlier with important implications for evidence on the surrogacy of late-stage cancer incidence for cancers with abysmal prognosis across all stages. It highlights that the relative incidence of late-stage cancer might not be a good surrogate for long-term mortality benefits for cancers in which there is little difference in survival between early and late stages. On this note, the trialists in the Qidong study also considered a Poisson regression model using their individual-level data, not available to us. They estimated that the rate ratio for death in the screening group was 0.83 (95% CI, 0.68–1.03) relative to the control group. These results are consistent with a small benefit on mortality that ultimately was not sustained because of the very poor prognosis across all stage groups in the trial population. One may also note that in this trial, 2-year survival was under 10% (in both arms), so the gain of using advanced stage, even if it worked, would be small. At the time that one could have used advanced stage as an outcome, there would already have been many deaths from individuals diagnosed with early-stage cancers. In general, this example shows that there might be some types of cancers detected by an MCED test for which the stage shift gained through screening is inconsequential for an improved outcome. Such cases should not deter researchers from investigating, for example, a refined surrogate that could be a useful early endpoint for a reduction in the incidence of advanced disease for most cancer types detectable by an MCED test.

### Conclusion

A reduction in the incidence of late-stage cancer diagnoses was correlated with a reduction in cancer-specific mortality across the majority of previous randomized controlled trials of cancer screening. In other words, a trial-level relationship between late-stage cancer incidence and mortality was evident. However, the evidence was based on a correlation of a limited range of cancer types and screening tests and limited data on late-stage incidence effects at an earlier timepoint than mortality results (but after the screening period had ended). Therefore, although the evidence supports further investigations into utilizing tumor characteristics as surrogates in future evaluations of new cancer screening tests, and acting now to develop methodologic guidance for including surrogates into future study designs, the interpretation of these data do not allow us to conclude that the incidence of late-stage disease can reliably replace disease-specific mortality for all cancers. Given the slow pace of starting and completing cancer screening trials, it would be prudent for trialists to act now to collect relevant stage and other prognostic tumor features so that these investigations can advance and the potential would exist to act on these findings if they were sufficiently persuasive.

## Supplementary Material

Supplementary Methods (Protocol) Supplementary Methods Preplanned Protocol is the final version of the pre-planned protocol after amendments.

Supplementary Methods and Tables Supplementary Methods and Tables: includes five sections. Section 1 outlines the comparison with previously published meta-analyses of mortality outcome surrogacy in cancer screening trials (including Table S1 and Table S2, and description of this work). Section 2 outlines the systematic searches in bibliographic databases and study selection (including Tables S3-S11 and description of this work). Section 3 provides detailed information on trials and the reported outcomes for the assessment of mortality outcome surrogacy (including Table S12-15, and a reference list for those four tables). Section 4 details the detailed meta-analytic results, including sensitivity and subgroup analyses (Including the methods that surround Figures S1-4, Table S16, Table S17). Section 5 includes selected aspects of limitations in the reported trial data (Table S19 and a description of this work).

Figure S1 Figure S1 shows the association between the screening effect on the incidence of late-stage cancer and the screening effect on mortality (trial-level surrogacy), by cancer type where at least three trials were available for analysis.

Figure S2 Figure S2 shows The effect of the timing of the reporting of late-stage incidence on the association with the mortality outcome, by cancer (if data were available for at least two time points).

Figure S3 Figure S3 shows he association between the screening effect on the proportion of late-stage cancers (among all cancers diagnosed up to the time of the reporting) and the screening effect on mortality (trial-level surrogacy), by cancer type where at least three trials were available for analysis.

Figure S4 Figure S4 shows the Evaluation of the utility of regarding the 95% CI RR for late-stage incidence as a “forecast” of the final trial result for cancer-specific mortality, only including 15 trial arm comparisons with the late-stage outcome RR reported prior to the primary analysis for mortality but after the screening period had ended.
