# Supplementary material for: Late-Stage Outcomes as Surrogates for Mortality in Cancer Screening Trials: A Systematic Review and Meta-analysis
Source: Cancer Epidemiol Biomarkers Prev. 2025 Jul 22;34(10):1694–709. doi: 10.1158/1055-9965.EPI-25-0201 (PMC12491949; doi:10.1158/1055-9965.EPI-25-0201)
Supplement: Supplementary Methods (Protocol) — Supplementary Methods Preplanned Protocol is the final version of the pre-planned protocol after amendments. [file epi-25-0201_supplementary_methods_protocol_suppsm1.pdf]

SURROGATES FOR MORTALITY IN CANCER SCREENING TRIALS (SUMS) – A SYSTEMATIC REVIEW AND META-ANALYSIS

PROTOCOL

Project team:

Prof Sian Taylor-Phillips* (University of Warwick)
Dr Matejka Rebolj* (Queen Mary University of London)
Dr Adam Brentnall (Queen Mary University of London)
Dr Julia Geppert (University of Warwick)
Nefeli Kouppa (Queen Mary University of London)
Dr Karoline Freeman (University of Warwick)
Dr Chris Stinton (University of Warwick)
Sam Johnson (University of Warwick)
Prof Peter Sasieni (Queen Mary University of London)
Prof Stephen Duffy (Queen Mary University of London)
Prof Robert Smith (American Cancer Society)
Prof Ruth Etzioni (Fred Hutchinson Cancer Center)
Prof Sarah Pinder (King's College London)
Prof Sam Janes (University College London)
Prof Keith Abrams (University of Warwick)
Eleanor Cozens (Public/patient)

Correspondence to:

Prof Sian Taylor-Phillips	Dr Matejka Rebolj
Warwick Medical School	Wolfson Institute of Population Health
University of Warwick	Queen Mary University of London
Coventry CV4 7AL, UK	London E1 1HH, UK
Email: S.Taylor-Phillips@warwick.ac.uk	m.rebolj@qmul.ac.uk

Version: 1.5
Date completed: 3 January 2024

* Dr Matejka Rebolj and Prof Sian Taylor-Phillips are joint co-leads and joint first authors.

The review was commissioned by Cancer Research UK (application reference: EDDCTR-2022/100001).

The views expressed in this protocol are those of the authors and not necessarily those of Cancer Research UK.

Any errors are the responsibility of the authors.

Version history

Version 1.1 – 22/09/2022 – Included draft search strategy, draft predefined list of surrogates and draft analysis plan.

Version 1.2 – 14/01/2023 – Finalised pre-defined list of surrogates (added absolute incidence of early stage cancer and edited exact definition of other surrogates), and added paper by Owens et al. (2022)[1] to analysis plan, plus edits to wording throughout.

Version 1.3 – 07/03/2023 – Further wording changes but no substantive change to content.

Version 1.4 – 06/04/2023 – Further wording changes but no substantive change to content, this version will be published on PROSPERO, and university website.

Version 1.5 – 03/01/2023 – Further wording changes but no substantive changes to content; this version will be used to update the PROSPERO record.

Research purpose

A comprehensive systematic review and meta-analysis of cancer screening randomised controlled trials (RCTs) will establish the strength of the evidence to support using intermediate outcomes as surrogate endpoints in future trials, for two purposes:

1. ***Sufficiency*** of the effect on the surrogate to conclude that screening is very likely to have a clinically significant impact on mortality. This would enable policymakers to plan for potential implementation and start pilot programmes and implementation research whilst awaiting mortality outcomes.
2. ***Futility*** of continuing/starting RCTs. Knowledge that the size of effect on the surrogate is small enough to rule out a clinically significant effect on mortality would enable researchers and funders to identify ineffective tests, prevent funding similar trials, and stop trials early (entirely, or by dropping the least promising interventions in multiarm-multistage trials).

Background

The literature evaluating surrogates in cancer treatment trials is extensive.[2-5] Meta-analysis of the correlation of differences between effect of intervention on surrogate and mortality across trials is widely used, often referred to as 'trial-level surrogacy'.[6] While certain agencies accept the use of surrogates such as tumour response or various definitions of survival to expedite approval of novel therapies,[7 8] many scientific reviewers advocate caution.[9-15]

Validation of surrogate or intermediate outcomes has additional methodological challenges for screening than for treatment trials. Studies need to determine whether surrogate endpoints in screening (including intermediate endpoints such as stage at diagnosis) reliably predict mortality and, hence, detecting a cancer **earlier** extends life. In breast screening, a meta-analysis of eight mammography RCTs provided some support to consider advanced cancer as a surrogate marker, after finding a very high correlation (≥0.90, p<0.001) between the effect of mammography on the rates of advanced breast cancer and breast cancer mortality.[16] For other cancers, candidate surrogates for screening have been discussed based on data from single RCTs. Within the context of an English RCT using flexible sigmoidoscopy for colorectal screening (Flexisig), projected mortality weighting incident cases by the probability of dying within a pre-specified time since randomisation was considered a reasonable surrogate for actual colorectal cancer mortality.[17] In the UKCTOCS RCT, which investigated two screening methods and reported results on at least two occasions, multi-modal screening using longitudinal CA125 testing led to an almost 50% increase in stage I ovarian cancer and a 24% decrease in stage IV, but did not reduce cancer-specific mortality.[18] Hence, the authors concluded that future trials of ovarian cancer screening should not

use stage distribution as a surrogate endpoint for ovarian cancer mortality.[18] Recent technological advances including the development of a variety of multi-cancer early detection screening tests (MCEDs*)* further emphasise the practical need to explore and validate surrogate endpoints to support conditional approval of promising new interventions. This would prevent interventions becoming obsolete while RCTs based on mortality are on-going or not started at all due to prohibitive costs.[19]

Approach

We will comprehensively review the literature on cancer screening trials reporting cancer-specific mortality, across all technologies and cancer types, to assess the strength of the evidence for predicting sufficiency or futility using selected surrogate endpoints in future trials.

Research Questions

In randomised controlled trials of cancer screening, is the effect of the screening interventions (compared to no screening or different screening) on the absolute incidence of late-stage cancer, predicted mortality, the proportion of cancer diagnosed at a late stage, or absolute incidence of early-stage cancer a sufficient surrogate for the effect on cancer-specific mortality and/or to predict the futility of continuation to mortality outcomes (overall, by modality, by cancer site, by site-modality combination) (RQ1)?

In randomised controlled trials of cancer screening, is the proportion of target cancers that are screen-detected, the proportion of aggressive target cancers that are screen-detected, or the diagnostic yield in the screening intervention arm a sufficient surrogate for the effect of the intervention on cancer-specific mortality and/or to predict the futility of continuation to mortality outcomes (overall, by modality, by cancer site, by site-modality combination) (RQ2)?

Outcome

Cancer-specific mortality for the target cancer for each trial. We will also examine all-cause mortality if reported in sufficient studies with adequate statistical power.

Surrogates

Where possible, we will extract and analyse the predefined surrogates detailed in **table 1** below, but due to the limitations of what trial authors report we will also extract and analyse reasonable approximations to the surrogates listed, documenting any deviations in definitions.

The surrogate of primary interest for *sufficiency* is absolute incidence of late-stage cancer (surrogate 1) and its translation into predicted mortality (surrogate 2 and analysis plan). Late-stage cancer often is far along on the causal pathway to cancer-specific mortality (poor prognosis), so is more likely to be a sufficient surrogate. However, this will vary by cancer type so different thresholds will be used and accounted for in the analysis where possible.

For surrogates 1, 2, 3 and 4 we would compare the treatment effect on the surrogate to the treatment effect on mortality. Surrogates 5, 6 and 7 are measured in the intervention arm of the trial only, so we would compare the surrogate (not the treatment effect on the surrogate) to the treatment effect on mortality.

Table 1. Potential surrogates and their definition

**Surrogate**	**Definition**
1. Absolute incidence of late-stage target* cancer<br><br>*target means the cancer that is the target of the screening programme (e.g. breast, bowel, oral, liver etc)	Numerator: Number of late-stage target cancers diagnosed after randomisation, by arm.<br>Denominator: Person-years of follow up in randomised individuals, or where not available number of randomised individuals, by arm.<br>Define 'late-stage' target cancer as:<br>• Using Stage: Stage II or worse, stage IIB or worse, stage III or worse, stage IV or worse;<br>• Using TNM: T4, and/or N≥1 and/or M1 (advanced);<br>• Cancer-specific alternatives: e.g., Dukes stage C or D for colorectal cancer;<br>• Or close cancer-specific approximations of the above.
2. Predicted mortality	Predicted mortality reported by trial authors, for example if the authors have combined the stage distribution in both arms of the trial with published survival rates by stage to predict mortality.
3. Proportion of target cancers diagnosed at late stage	Numerator: Number of late-stage target cancers diagnosed after randomisation, by arm.<br>Denominator: Total number of target cancers during follow-up, by arm.<br>Define 'late-stage' target cancer as stage II or worse, stage IIB or worse, stage III or worse, stage IV or worse, or cancer-specific alternatives (as above).
4. Absolute incidence of early-stage target* cancer	Numerator: Number of early-stage target cancers diagnosed after randomisation, by arm.<br>Denominator: Person-years of follow up in randomised individuals or number of randomised individuals, by arm.<br>Define 'early-stage' target cancer using numerical stage with different thresholds, or cancer-specific alternatives.<br>If data permit also evaluate absolute incidence of early-stage high grade cancer detection defined as:<br>• Grade 3 and stage I or II;<br>• Grade 2+ and stage I or II;<br>• Or cancer-specific, e.g. prostate cancer Gleason 7+ or Gleason 8+ and stage I or II.
5. Proportion of target cancers that are screen-detected	Numerator: Number of screen-detected target cancers in intervention arm.<br>Denominator: Total number of target cancers detected (screen-detected and symptomatically detected) in intervention arm.
6. Proportion of high-grade target cancers that are screen-detected	Numerator: Number of grade 3 target cancers that are screen-detected in intervention arm.<br>Denominator: Total number of grade 3 target cancers detected in intervention arm (screen-detected and symptomatically detected).<br>Where available and appropriate repeat for grade 2&3

	(Or cancer-specific, e.g. prostate: Gleason 7+ or 8+)
7. Diagnostic yield of screening	Numerator: Number of cancers that are screen-detected in intervention arm.<br>Denominator: Number of individuals randomised into the intervention arm.<br>Where available and appropriate also include number of 'pre-cancers' detected (e.g. CIN 2-3 for cervical cancer, acute adenoma for colorectal cancer, ductal carcinoma in situ in breast cancer)<br>Where available and appropriate also include subset of high-grade (2/3) cancers detected.

Objectives

i. Narratively synthesise previous studies evaluating sufficiency of screening surrogates and trial-level surrogacy by cancer and modality from included RCTs (RQ1&2).
ii. Meta-analyse trial-level surrogacy from included RCTs (overall, by modality, by cancer site, by site-modality combination) and explore influences on surrogate sufficiency and futility through meta-regression (RQ1&2).
iii. Report findings and recommendations for research and policy in journals, to the funder (Cancer Research UK), and to policy and practice advisory group.

Search Strategy

We will first identify eligible screening trials with a mortality outcome reported ('Search 1'), then identify additional papers from each eligible trial reporting (at least) one of our pre-specified surrogates or further mortality outcomes ('Search 2').

IDENTIFICATION OF TRIALS WITH RELEVANT DATA

Search 1
(Mortality outcomes)

Search strategy, eligibility criteria and review strategy

Aim	To find all relevant RCTs in primary cancer screening reporting mortality outcomes in the intervention and control arms and published to date.
Search strategy	Pre-specified electronic search in bibliographical databases, using a list of keywords/terms compiled and tested by the study team, developed in collaboration with an expert librarian (**Appendix 1**).
Supplemental searches	Contacting experts in the field,<br>most recent USPSTF cancer screening reviews (as identified by https://www.uspreventiveservicestaskforce.org/uspstf/topic_search_results?topic_status=P&category%5B%5D=15&type%5B%5D=5&searchterm=),<br>IARC handbooks of cancer prevention (colorectal, breast, and cervical).
Review strategy	Titles and abstracts of the publications found by the search screened independently by two reviewers, consulting all full text publications considered potentially relevant by either reviewer;<br>full text articles assessed against the inclusion/exclusion criteria independently by two reviewers, with disagreements resolved by a third reviewer.
Inclusion criteria	PICOS (Search 1):<br>Population: people (any age: children or adults) without apparent symptoms of cancer that the trial is aiming to detect at a pre-symptomatic stage or to prevent, from the general population or from a higher cancer-risk group.<br>Intervention: a screening regimen for cancer of any type, with the screening intervention meeting the following criteria:<br>• Single or repeated test;<br>• Test should be defined (including any cut-point) prior to the study;<br>• The test may be performed in or outside of a health care facility (including e.g., self-sampling tests or self-examination);<br>• There should be an agreed policy on further diagnostic investigation of individuals with a positive test result and on the choices available to those individuals.<br>Comparator: no screening or another type of screening.<br>Outcome: must report cancer-specific or all-cause mortality.<br>Study design: the test must have been studied in an individually or cluster randomised controlled trial.
Exclusion criteria	• Non-randomised studies;<br>• Non-human studies;<br>• Publications with no mortality outcomes reported;<br>• Letters, reviews, editorials and communications with insufficient information on methods and/or no numerical outcomes data;<br>• Grey literature and conference abstracts;<br>• Articles not available in the English language.
Key outcome	List of eligible screening trials reporting mortality outcomes.
Documentation	PRISMA flow diagram of publications included and excluded at each stage of the review; reasons for exclusion of records at full text level will be documented.

Search 2

(Related publications of trials identified in 'Search 1' reporting intermediate outcomes or further mortality outcomes)

Search strategy, eligibility criteria and review strategy

Aim	To find all relevant publications to date from the trials identified in 'Search 1' that report intermediate outcomes that might be considered as surrogates or further mortality outcomes.
Search strategy	Pre-specified electronic search in bibliographical databases using identifying terms for each trial, developed in collaboration with an expert librarian (**Appendix 2**).
Supplemental searches	If, at the data extraction stage, the text of an included record mentions a reference to another publication with mortality and/or intermediate outcome data that was missed by our search, we will assess it for inclusion and, if deemed relevant, extract the data.
Review strategy	Titles and abstracts of the publications found by the search screened independently by two reviewers, consulting all full text publications considered potentially relevant by either reviewer;<br>full text articles assessed against the inclusion/exclusion criteria independently by two reviewers, with disagreements resolved by a third reviewer.
Key criteria for selection of relevant publications	As 'Search 1', except<br>PICOS Outcome (Search 2): Presenting at least one intermediate outcome in the intervention and the control arm (potential surrogates 1.-4. from the pre-specified list, see **table 1**) or further cancer-specific or all-cause mortality outcomes.
Exclusion criteria	As 'Search 1', except<br>Publications with no intermediate outcomes in the intervention and control arms (potential surrogates 1.-4. from the pre-specified list, see **table 1**) or mortality outcomes reported.
Key outcome	1. List of trials with a full list of their publications reporting on observed mortality and intermediate endpoints to date;<br>2. Numbering of each publication for reference throughout the project (publication IDs);<br>3. List of trials reporting mortality but not also intermediate outcomes.
Documentation	PRISMA flow diagram of publications included and excluded at each stage of the review; reasons of records excluded at full text level will be documented.

EXTRACTION OF DATA TO INFORM THE SYSTEMATIC REVIEW AND META-ANALYSIS

For each eligible screening trial, all identified publications ('Search 1' and 'Search 2' combined) will be mapped by outcomes (mortality, intermediate endpoints) and timepoints reported. If, for a specific trial, we only identify articles with mortality outcomes, but no surrogate outcomes, the trial will be excluded from data extraction. If several papers from the same trial report the same

outcomes at the same timepoint and for the same group of participants, only papers providing the most comprehensive information will be included in the data extraction process.

All data extraction will be entered into a piloted electronic data collection form. All identified publications from one screening trial will be extracted in the same Excel file. We will extract general information on the trial design and methods (e.g., eligibility criteria, randomisation process, study flow, population, intervention, and comparator) as well as paper-specific statistical methods and results on the reported mortality and surrogate outcomes.

Data extraction will be performed by one reviewer and checked by a second reviewer. Disagreements will be resolved by a third reviewer and/or by contacting trial authors.

ASSESSMENT OF METHODOLOGICAL QUALITY

The risk of bias in our analysis will be assessed using a tailored version of the revised (version 2) 'Cochrane risk-of bias tool for randomized trials' (RoB 2).[20] The tailored version will be defined by the project team to include items of relevance for studies of outcome surrogacy rather than focus on biases affecting screening efficacy/effectiveness. For example, items such as contamination or attrition taking place between the time point of surrogate measurement and mortality measurement may affect surrogacy assessment to a greater extent than overall contamination or attrition in the trial. Risk of bias will be assessed for outcomes measured at the "main" timepoint for each trial. The trial-specific "main" timepoint will be determined by group consensus using judgement alongside available information such as from the statistical analysis plan or the power calculation for that trial.

Critical appraisal will be performed by one reviewer and checked by a second reviewer. Disagreements will be resolved through consensus, with the inclusion of a third reviewer if required. The results of each risk of bias item will be presented in table and/or graph form.

DATA ANALYSIS

Aim

Meta-analyse trial-level surrogacy from included RCTs (overall, by modality, by cancer site, by site-modality combination) and explore influences on surrogate sufficiency through meta-regression.

Effect measures

To assess how well the surrogate endpoint predicts subsequent cancer-specific mortality, we will use relative risks associated with screening for both surrogates and mortality that have been extracted. Some trials might instead report hazard or rate ratios, but mortality is a rare outcome in screening trials so these would be treated as equivalent to relative risks.

The primary analysis will use the main analysis mortality timepoint for each trial as identified by reports and, where available, an earlier timepoint for the surrogate endpoint.

The best choice for the timepoint of the surrogate may depend on the natural history of the cancer and the nature of the screening test. If follow-up time is too short (for example shorter than the lead times of the cancer) then later stage cancers will not yet have emerged in the control group. If follow-up time is too long then the surrogate does not provide the same potential benefit in earlier decision-making and may be affected by newly developed cancers after the screening intervention. To account for these, we will use the closest timepoint to the midpoint between the end of the intervention and the main mortality timepoint. The timepoint used will need to have sufficient follow-up after the intervention has ended. In cases where this is not available or where the mortality endpoint is measured before the intervention has ended, we will use the same timepoint as the main analysis timepoint for mortality.

Analysis will be done separately for the surrogates extracted both:

(1) for all cancer types combined (surrogates 1-6,where data are available)*

(2) for each cancer type separately (as subgroup analysis of (1), see below).

* cancers for which the intervention has the role of prevention (e.g. bowel, cervix) will not be included in analysis (1) for the assessment of surrogate 4 (absolute incidence of early-stage target cancer).

Comparisons

The analysis will use intention-to-treat effect estimates based on the trial outcomes.

Adjustments

Primary analysis will use unadjusted effect estimates when there is a choice, or with the same adjustments for both surrogate and cancer mortality outcomes.

Data synthesis

The layout of result tables is presented in **Appendix 3**.

The trials will be summarised (**table A1**), as will be the relative risk estimates for the screening effect on surrogates and mortality from each trial with 95% CIs (**table A2**).

To assess the association between late-stage incidence and cancer-specific mortality across multiple cancer types we will use the methodology reported by Owens et al.[1] This method will be applied to cancer types and trials for which appropriate input data (survival estimates) are promptly available.

The association between surrogates and mortality relative risks will be shown graphically using bubble plots wherever R^2 is reported in the tables **(figure A1)**. Forest plots may be used (and chosen post hoc) to show the univariate surrogate relative risk data graphically from tables where it aids interpretation, but without formal meta-analytic summaries.

Quantitative summaries across studies will be undertaken when there are sufficient studies of adequate quality (minimum three). For these, the primary measures of the utility of different surrogate endpoints will be 95% CI for the correlation (of the logarithm relative-risk) and variance explained (R^2) between the screening effect on the surrogate and on cancer-specific mortality across trials **(table A3a)**. This will follow the methodology of multiple meta-analyses of surrogate endpoints for cancer treatment trials. The main analysis will use one data point from each study (i.e., an overall effect for the surrogate, and for cancer-specific mortality).

Heterogeneity and subgroups

Heterogeneity between studies is expected from our wide inclusion criteria, but valuable because it will likely yield the variation in observed cancer-specific mortality and surrogate effects that are needed to assess association. Secondary analysis will consider independent subgroups within each study, which will be accounted for in the analysis through random effects for study (see heterogeneity and subgroups below) **(table A3b)**. That is, we will explore use of within-trial subgroups to better assess correlation in the meta-analysis (e.g., if a trial reports relative risks by age at entry groups or trial centre these may be used as independent data points to assess correlation).

Trial-level sources of heterogeneity will be explored using standard meta-regression analyses. In particular, we will assess potential heterogeneity using study specific covariates for:

- Cancer type (as defined in review criteria)
- Population type (population risk vs. high risk)
- Test type (this will depend on the cancer and test identified in the review, and post-hoc expert judgement on comparability of test types)
- Epoch (chosen post hoc based on distribution of epochs in the data and expert judgement)
- Timing of endpoint relative to natural history of cancer. The time difference in the reporting of the two outcomes may amount to several years, depending on what is reported. Different follow-up times may be assessed post hoc based on expert judgement for each surrogate and cancer type.
- Trial design (e.g., with respect to number of screening rounds in the intervention and control arms)

Separate result summaries will be presented by subgroup (**tables A4-A9**).

Sensitivity analysis

We will assess robustness of the results to the definition of the main analysis time for each trial by using (reasonable) earlier and later timepoints available for the surrogate and mortality endpoints and repeating the analysis **(table A10)**. We will also assess the robustness depending on the adjustments in the reported effect measures **(table A11)**.

Exploratory analysis

Alternative methods to assess surrogacy may be used. This might include methods proposed by Burzykowski & Buyse,[21] Buyse et al.[21 22] and Baker.[23]

REFERENCE LIST

1. Owens L, Gulati R, Etzioni R. Stage Shift as an Endpoint in Cancer Screening Trials: Implications for Evaluating Multicancer Early Detection Tests. *Cancer Epidemiol Biomarkers Prev* 2022;31(7):1298-304. doi: 10.1158/1055-9965.EPI-22-0024
2. Bujkiewicz S, Jackson D, Thompson JR, et al. Bivariate network meta-analysis for surrogate endpoint evaluation. *Stat Med* 2019;38(18):3322-41. doi: 10.1002/sim.8187 [published Online First: 20190526]
3. Bujkiewicz S, Thompson JR, Riley RD, et al. Bayesian meta-analytical methods to incorporate multiple surrogate endpoints in drug development process. *Stat Med* 2016;35(7):1063-89. doi: 10.1002/sim.6776 [published Online First: 20151103]
4. Bujkiewicz S, Thompson JR, Spata E, et al. Uncertainty in the Bayesian meta-analysis of normally distributed surrogate endpoints. *Stat Methods Med Res* 2017;26(5):2287-318. doi: 10.1177/0962280215597260 [published Online First: 20150813]
5. Papanikos T, Thompson JR, Abrams KR, et al. Bayesian hierarchical meta-analytic methods for modeling surrogate relationships that vary across treatment classes using aggregate data. *Stat Med* 2020;39(8):1103-24. doi: 10.1002/sim.8465 [published Online First: 20200128]
6. Buyse M, Sargent DJ, Grothey A, et al. Biomarkers and surrogate end points--the challenge of statistical validation. *Nat Rev Clin Oncol* 2010;7(6):309-17. doi: 10.1038/nrclinonc.2010.43 [published Online First: 20100406]
7. Ciani O, Grigore B, Blommestein H, et al. Validity of Surrogate Endpoints and Their Impact on Coverage Recommendations: A Retrospective Analysis across International Health Technology Assessment Agencies. *Med Decis Making* 2021;41(4):439-52. doi: 10.1177/0272989X21994553 [published Online First: 20210310]
8. Gyawali B, Hey SP, Kesselheim AS. Evaluating the evidence behind the surrogate measures included in the FDA's table of surrogate endpoints as supporting approval of cancer drugs. *EClinicalMedicine* 2020;21:100332. doi: 10.1016/j.eclinm.2020.100332 [published Online First: 20200413]
9. Baker SG. Surrogate endpoints: wishful thinking or reality? *J Natl Cancer Inst* 2006;98(8):502-3. doi: 10.1093/jnci/djj153
10. Ciani O, Davis S, Tappenden P, et al. Validation of surrogate endpoints in advanced solid tumors: systematic review of statistical methods, results, and implications for policy makers. *Int J Technol Assess Health Care* 2014;30(3):312-24. doi: 10.1017/S0266462314000300
11. Cooper K, Tappenden P, Cantrell A, et al. A systematic review of meta-analyses assessing the validity of tumour response endpoints as surrogates for progression-free or overall survival in cancer. *Br J Cancer* 2020;123(11):1686-96. doi: 10.1038/s41416-020-01050-w [published Online First: 20200911]
12. Haslam A, Hey SP, Gill J, et al. A systematic review of trial-level meta-analyses measuring the strength of association between surrogate end-points and overall survival in oncology. *Eur J Cancer* 2019;106:196-211. doi: 10.1016/j.ejca.2018.11.012 [published Online First: 20181205]
13. Prasad V, Kim C, Burotto M, et al. The Strength of Association Between Surrogate End Points and Survival in Oncology: A Systematic Review of Trial-Level Meta-analyses. *JAMA Intern Med* 2015;175(8):1389-98. doi: 10.1001/jamainternmed.2015.2829
14. Xie W, Halabi S, Tierney JF, et al. A Systematic Review and Recommendation for Reporting of Surrogate Endpoint Evaluation Using Meta-analyses. *JNCI Cancer Spectr* 2019;3(1):pkz002. doi: 10.1093/jncics/pkz002 [published Online First: 20190206]
15. Zhao F. Surrogate End Points and Their Validation in Oncology Clinical Trials. *J Clin Oncol* 2016;34(13):1436-7. doi: 10.1200/JCO.2016.66.4581 [published Online First: 20160307]
16. Tabar L, Yen AM, Wu WY, et al. Insights from the breast cancer screening trials: how screening affects the natural history of breast cancer and implications for evaluating service screening programs. *Breast J* 2015;21(1):13-20. doi: 10.1111/tbj.12354 [published Online First: 20141120]

17. Cuzick J, Cafferty FH, Edwards R, et al. Surrogate endpoints for cancer screening trials: general principles and an illustration using the UK Flexible Sigmoidoscopy Screening Trial. *J Med Screen* 2007;14(4):178-85. doi: 10.1258/096914107782912059
18. Menon U, Gentry-Maharaj A, Burnell M, et al. Ovarian cancer population screening and mortality after long-term follow-up in the UK Collaborative Trial of Ovarian Cancer Screening (UKCTOCS): a randomised controlled trial. *Lancet* 2021;397(10290):2182-93. doi: 10.1016/S0140-6736(21)00731-5 [published Online First: 20210512]
19. Raoof S, Lee RJ, Jajoo K, et al. Multicancer Early Detection Technologies: A Review Informed by Past Cancer Screening Studies. *Cancer Epidemiol Biomarkers Prev* 2022;31(6):1139-45. doi: 10.1158/1055-9965.EPI-21-1443
20. Sterne JAC, Savovic J, Page MJ, et al. RoB 2: a revised tool for assessing risk of bias in randomised trials. *BMJ* 2019;366:l4898. doi: 10.1136/bmj.l4898 [published Online First: 20190828]
21. Burzykowski T, Buyse M. Surrogate threshold effect: an alternative measure for meta-analytic surrogate endpoint validation. *Pharm Stat* 2006;5(3):173-86. doi: 10.1002/pst.207
22. Buyse M, Molenberghs G, Burzykowski T, et al. The validation of surrogate endpoints in meta-analyses of randomized experiments. *Biostatistics* 2000;1(1):49-67. doi: 10.1093/biostatistics/1.1.49
23. Baker SG. A simple meta-analytic approach for using a binary surrogate endpoint to predict the effect of intervention on true endpoint. *Biostatistics* 2006;7(1):58-70. doi: 10.1093/biostatistics/kxi040 [published Online First: 20050622]

APPENDIX 1

Bibliographical search terms to identify relevant randomised controlled trials (‘Search 1’)

Ovid MEDLINE(R) ALL <1946 to September 21, 2022>

1	cancer*.mp.	2155014
2	neoplasm*.mp.	3168201
3	exp Neoplasms/	3737394
4	1 or 2 or 3	4385892
5	screen*.mp.	990088
6	exp Mass Screening/	141720
7	exp "Early Detection of Cancer"/	34964
8	5 or 6 or 7	1008832
9	exp Mortality/ or mortality.mp.	1463207
10	exp Randomized Controlled Trial/ or exp Random Allocation/	667865
11	randomized controlled trial.pt.	577339
12	10 or 11	667865
13	4 and 8 and 9 and 12	1106

Embase Classic+Embase <1947 to 2022 September 21>

1	cancer*.mp.	4372531
2	neoplasm*.mp.	983532
3	exp neoplasm/	5648200
4	1 or 2 or 3	6467573
5	screen*.mp.	1612332
6	exp mass screening/	293380
7	exp early cancer diagnosis/	11575
8	5 or 6 or 7	1620653
9	mortality.mp. or exp cancer mortality/ or mortality/	1841601
10	exp randomized controlled trial/	733200
11	random allocation.mp. or exp randomization/	97605
12	10 or 11	806321
13	4 and 8 and 9 and 12	1987
14	limit 13 to (article or article in press or "review")	1160

Web of Science
https://www.webofscience.com/wos/woscc/summary/f4a58e16-29cb-4e99-b809-0c2750a3b0ee-50bdb465/relevance/1

cancer* or neoplasm* (Topic) and screen* or "early detect*" (Topic) and rct* or "randomized controlled trial*" or "random allocat*" or "randomised controlled trial*" (Topic) and mortality

The results of these searches will be combined into a single file and de-duplicated.

APPENDIX 2

Bibliographical search terms to identify publications reporting intermediate outcomes from trials identified in 'Search 1' ('Search 2')

'Search 2' will comprise of a series of mini-searches based on the individual trials found from 'Search 1'. The following template will be used to retrieve papers in Medline (OVID) and Embase.

Medline search template.

[screen*.mp. OR exp Mass Screening/ OR exp "Early Detection of Cancer"/]
AND
[exp Random Allocation/ OR exp Randomized Controlled Trial/ OR randomized controlled trial.pt.]
AND
[Cancer type]
AND
[Screening tool]
AND
[Geographical location]

These results were ORed with the trial name or code/number.

Embase search template.

[screen*.mp. OR exp Mass Screening/ OR exp early cancer diagnosis/]
AND
[random*.mp. OR exp Randomized Controlled Trial/ OR exp randomization]
AND
[Cancer type]
AND
[Screening tool]
AND
[Geographical location]

These results will be ORed with the trial name or code/number.

The results of these searches will be combined into a single file and de-duplicated for each trial.

If, for any trial, the resulting number of publications will be higher than 200, we will limit the selection to papers authored by at least one of the main investigators on the trial (minimum two names, but for most trials this will be three or higher).

APPENDIX 3

Statistical analysis: figure and table layout

Figure A1. Bubble plot. Legend will include details of correlation coefficient, what size of ellipse indicates (width of both confidence intervals), R^2 value. This plot will be produced for each analysis where correlation or R^2 is reported.

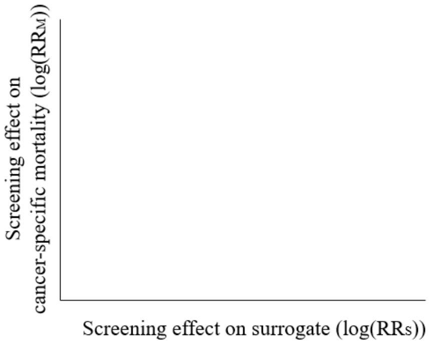

Table A1. Characteristics of included trials.

Trial abbreviation	Trial name	Population type	Age range	Country	Cancer type	Screening test	Timing of first screening	Timing of last screening	Frequency of screening	Management in the control arm

Randomisation design	N randomised		Level of Compliance	Level of Contamination	Notes	Sources
	Intervention	Control				

Table A2. Summary of results from included trials.

Trial abbreviation	Endpoint	Time point for analysis	Analysis timing	Category timing	Comparison	N individuals		N events		Same sample size as primary analysis	Person years	
						Intervention	Control	Intervention	Control		Intervention	Control

Risk measure	Risk		RR	L95%CI	U95%CI	RR Method	Adjustments	Notes	Sources
	Intervention	Control							

Table A3a. Primary analysis, one observation per trial.

Cancer type	Surrogate	N trials	Correlation (95%CI)	R^2 (95%CI)

Table A3b. Secondary analysis, multiple independent units per trial.

Cancer type	Surrogate	N trials	Correlation (95%CI)	R^2 (95%CI)

Table A4. Subgroup analysis, population type

Cancer type	Population type	Surrogate	Comparison type	N trials	Correlation (95%CI)	R^2 (95%CI)

Table A5. Subgroup analysis, comparison type

Cancer type	Comparison	Surrogate	Comparison type	N trials	Correlation (95%CI)	R^2 (95%CI)

Table A6. Subgroup analysis, follow-up time

Cancer type	Surrogate Follow-up	Mortality Follow-up	Surrogate	Comparison type	N trials	Correlation (95%CI)	R^2 (95%CI)

Table A7. Subgroup analysis, test type

Cancer type	Test type	Surrogate	Comparison type	N trials	Correlation (95%CI)	R^2 (95%CI)

Table A8. Subgroup analysis, epoch

Cancer type	Epoch	Surrogate	Comparison type	N trials	Correlation (95%CI)	R^2 (95%CI)

Table A9. Subgroup analysis, trial design

Cancer type	Trial design	Surrogate	Comparison type	N trials	Correlation (95%CI)	R^2 (95%CI)

Table A10. Sensitivity analysis, mortality follow-up

Cancer type	Mortality Follow-up	Surrogate	Comparison type	N trials	Correlation (95%CI)	R^2 (95%CI)

Table A11. Sensitivity analysis, adjustment

Cancer type	Adjustment	Surrogate	Comparison type	N trials	Correlation (95%CI)	R^2 (95%CI)
