# Supplementary material for: Late-Stage Outcomes as Surrogates for Mortality in Cancer Screening Trials: A Systematic Review and Meta-analysis
Source: Cancer Epidemiol Biomarkers Prev. 2025 Jul 22;34(10):1694–709. doi: 10.1158/1055-9965.EPI-25-0201 (PMC12491949; doi:10.1158/1055-9965.EPI-25-0201)
Supplement: Supplementary Methods and Tables — includes five sections. Section 1 outlines the comparison with previously published meta-analyses of mortality outcome surrogacy in cancer screening trials (including Table S1 and Table S2, and description of this work). Section 2 outlines the systematic searches in bibliographic databases and study selection (including Tables S3-S11 and description of this work). Section 3 provides detailed information on trials and the reported outcomes for the assessment of mortality outcome surrogacy (including Table S12-15, and a reference list for those four tables). Section 4 details the detailed meta-analytic results, including sensitivity and subgroup analyses (Including the methods that surround Figures S1-4, Table S16, Table S17). Section 5 includes selected aspects of limitations in the reported trial data (Table S19 and a description of this work). [file epi-25-0201_supplementary_methods_and_tables_suppsm2.docx]

# Supplementary Methods and Tables for “Late-stage outcomes as surrogates for mortality in cancer screening trials: a systematic review and meta-analysis”

# Section 1. Comparison with previously published meta-analyses of mortality outcome surrogacy in cancer screening trials

To our knowledge, two other meta-analyses investigating the role of late-stage cancer outcomes as surrogates for cancer-specific mortality in cancer screening have reported their results. Here, we compare their study designs and outcomes.

Notation:

**Feng et al.**: Feng X, Zahed H, Onwuka J, Callister MEJ, Johansson M, Etzioni R, Robbins HA. Cancer Stage Compared With Mortality as End Points in Randomized Clinical Trials of Cancer Screening: A Systematic Review and Meta-Analysis. JAMA 2024;331(22):1910-1917. doi: 10.1001/jama.2024.5814.

**Dai et al.**: Dai JY, Luebeck EG, Chang ET, Clarke CA, Hubbell EA, Zhang N, Duffy SW. Strong association between reduction of late-stage cancers and reduction of cancer-specific mortality in meta-regression of randomized screening trials across multiple cancer types. J Med Screen 2024: 9691413241256744. doi: 10.1177/09691413241256744. Online ahead of print.

**Rebolj et al.**: the present meta-analysis.

## Table S1. Comparison of the designs of the three meta-analyses exploring the relationship between late-stage cancer and cancer-specific mortality in cancer screening, and a summary of their results.

|  | **Feng et al.** | **Dai et al.** | **Rebolj et al.** |
| --- | --- | --- | --- |
| **Published protocol (registry name, ID)** | Yes (PROSPERO, CRD42023411132) | Not reported | Yes (PROSPERO, CRD42023369320) |
| **Databases and other sources searched** | PubMed (by 19/02/2024); previous reviews; ConnectedPapers | USPSTF reviews, updated to 22/08/2023 through PubMed; reference lists; reviews | Stage 1 (identification of trials): Medline, Embase, Web of Science up to 21/09/2022; expert suggestions, USPSTF reviews, IARC handbooks; introduction and discussion of included papers.  Stage 2 (identification of trial-specific publications): Medline and Embase (11/01 to 18/04/2023); introduction and discussion of included papers. |
| **N records screened** | 1209 (+2 from other sources) | 35 (USPSTF reviews) + 1099 (update of USPSTF reviews) + 366 (other cancer types) | Stage 1: 3154 (+188 from other sources),  Stage 2: 4249 (+16 from other sources) |
| **Eligibility criteria for trials** (other than: a randomised cancer screening trial for any cancer type, any test, reporting quantitative results for late-stage cancer and mortality in English language) | No exit screen; enrolled participants without health conditions; cancers reported so that they could be categorised into stage III+IV vs. stage I+II | “Rigorously conducted”, “informative” trials according to USPSTF criteria, adequate randomisation, reported power and sample size planning, adequately powered for the mortality outcome, reported TNM stage | Cancers categorised as late-stage by trialists, or could be categorised as such by the reviewers |
| **N trials / N comparisons including trials with multiple arms / N cancer types** | 39 / 41 / 8 | 33 / 33 / 7 | 57 / 61 / 10 |
| **Calculation of relative risks or similar indicators of the screening effect on the analysed outcomes** | Relative risks calculated by reviewers for all trials, based on N participants and N with an event, by arm:  $RR=\frac{\frac{N_{outcome}^{intervention}}{N_{participants}^{intervention}}}{\frac{N_{outcome}^{control}}{N_{participants}^{control}}}$ | Relative risks as reported by trialists. If not reported, calculated based on the reported counts and estimated the standard error by Poisson regression | Rate ratios, relative risks or hazard ratios as reported by trialists. If not reported, calculated rate ratios where person-years were available, otherwise risk ratios, with Wald-type 95% CI |
| **Definition of late-stage cancer in primary analyses** | Stages III+IV for all cancer types;  if needed, translated from tumour size (≥20 mm), Dukes stage (C or D), localised/advanced or resected early/late unresected categorisations (advanced or late stage unresected), Gleason score (8-10 or metastatic), prognostic group (high risk or advanced) | Cancer-specific, TNM stage translated by the reviewers according to pre-specified criteria (mortality difference, prevalence, within-group variability):  Lung, ovary: stage III+IV  Bowel: stage III+IV (Dukes’ stage C+D) or IV (Dukes’ D)  Breast: stage II+  Prostate: stage IV | As reported by trialists. If not reported: followed the example from trials within the same cancer type.  Two trials with preventive potential of screening: any cancer.  Prostate: preferred Gleason score |
| **Indicators of the late-stage cancer outcome** | 1 – Relative late-stage incidence reduction  2 – Relative reduction in the proportion of late-stage cancers | Relative late-stage incidence reduction | 1 – Relative late-stage incidence reduction  2 – Relative reduction in the proportion of late-stage cancers |
| **Selection of the main mortality endpoint** (if multiple available) | Earliest available | Latest available | “Main” mortality timepoint identified as such by trialists; otherwise, the most suitable timepoint (e.g., the latest) |
| **Statistical measures to judge the relationship between late-stage cancer and cancer mortality outcomes** | Cancer-specific analyses (bowel, breast, lung, ovary, prostate)  1 – Unweighted Pearson correlation coefficients if ≥4 observations (ideal: correlation close to 1).  2 – Binary agreement: comparison of the statistical significance at α=0.05 (or at α=0.10) for the screening effect on the late-stage vs. mortality outcomes (ideal: both significant or both insignificant).  3 – Linear regression slopes (ideal: close to 1).  All cancers combined (bowel, breast, lung, ovary, prostate)  1 - Pearson correlation coefficient (bowel, breast, lung, ovary, prostate).  2 – Binary agreement: idem, includes also 4 trials on liver, nasopharyngeal and oral cancers | Cancer-specific analyses (bowel, breast, lung, prostate)  Weighted linear meta-regression  1 - slope (ideal: significantly greater than 0),  2 - intercept (ideal: close to 0),  3 - R^2^ (ideal: close to 1)  All cancers combined (bowel, breast, lung, prostate, nasopharyngeal, oral, ovarian)  Weighted average R^2^ across cancers (adjusted for different slopes and intercepts among different cancer types). | Cancer-specific analyses (bowel, breast, cervix, lung, ovary, prostate, multiple [venous thromboembolism patients])  Weighted Pearson correlation coefficients/R^2^ based on weighted fixed-effects linear model of log relative risk estimates if ≥3 observations.  All cancers combined (bowel, breast, cervix, lung, ovary, prostate, multiple [venous thromboembolism patients], liver, nasopharyngeal, oral)  Idem, for unit of observation=trial or a combination of cancer type and test type |
| **Absolute incidence of late-stage cancer: R^2^ (95% CI or p, if reported)** | Bowel: 0.15  Breast: 0.49  Lung: 0.84  Prostate: 0.47  Ovary: 0.97 | Bowel: 0.75  Breast: 0.94  Lung: 0.79  Prostate: 0.98  All cancers: 0.75 | Bowel: 0.34 (0.07-0.86)  Breast: 0.62 (0.24-0.88)  Lung: 0.83 (0.71-0.92)  Prostate: 0.18 (0.00-1.00)  Ovary: 0.94 (0.00-1.00)  Cervix: 0.59 (0.52-1.00)  Multiple: 0.16 (0.00-1.00)  All cancers: 0.47 (0.23-0.71) |
| **Absolute incidence of late-stage cancer: regression slope (95% CI or p, if reported)** | Bowel: 0.40 (-0.22-1.02)  Breast: 0.28 (-0.001-0.57)  Lung: 0.75 (0.54-0.95)  Prostate: -3.58 (-8.84-1.69)  Ovary: 1.15 (0.89-1.41) | Bowel: 0.91 (0.01)  Breast: 0.84 (0.01)  Lung: 0.72 (0.0001)  Prostate: 0.45 (0.01) | Bowel: 0.42 (0.17-1.06)  Breast: 0.61 (0.37-0.87)  Lung: 0.70 (0.52-0.90)  Prostate: 0.59 (-3.83-9.04)  Ovary: 0.98 (-inf-inf)  Cervix: 0.69 (0.55-2.84)  Multiple: -0.75 (-inf-inf)  All cancers: 0.58 (0.36-0.82) |
| **Absolute incidence of late-stage cancer: other study-specific results from primary cancer-specific analyses** | Pearson correlation (95% CI)  Bowel: 0.39 (-0.27-0.80)  Breast: 0.70 (-0.26-0.96)  Lung: 0.92 (0.72-0.98)  Prostate: -0.69 (-0.99-0.81)  Ovary: 0.99 (0.51-1.00)  All cancers: 0.75 (0.51-0.89)  Binary agreement (at α=0.05)  Bowel: 4/6 concordant  Breast: 7/11 concordant  Lung: 10/12 concordant  Ovary: 3/4 concordant  Prostate: 2/4 concordant  Other: 3/4 concordant  All cancers: 29/41 (71%) concordant;  8/13 (62%) with reduced late-stage outcome showed no mortality reduction;  4/9 (44%) with reduced mortality showed no late-stage reduction | Intercept  Bowel: 0.03  Breast: 0.05  Lung: 0.038  Prostate: 0.003 | Pearson correlation (95% CI)  Bowel: 0.58 (0.27-0.93)  Breast: 0.79 (0.49-0.94)  Lung: 0.91 (0.84-0.96)  Prostate: 0.42 (-1.00-1.00)  Ovary: 0.97 (-1.00-1.00)  Cervix: 0.77 (0.72-1.00)  Multiple: -0.40 (-1.00-1.00)  All cancers: 0.69 (0.47 to 0.84) |
| **Proportion with late-stage cancer** | Poorer binary agreement compared with the absolute incidence indicator | Not reported | Substantially less strong and consistent relationships than with the absolute incidence indicator |
| **Conclusions as reported by reviewers (overall)** | “[…] incidence of late-stage cancer may be a suitable alternative end point to cancer-specific mortality for some cancer types, but is not suitable for others.”  “Stage III-IV cancer incidence is unlikely to be a suitable alternative end point to cancer specific mortality in screening trials for breast, colorectal, and prostate cancers.” | “[…] we observed consistent, strong linear trends across multiple targeted cancer types between reductions in late-stage cancer incidence and reductions in cancer-specific mortality.”  “A reduction of late-stage cancer incidence may serve as an effective early indicator for mortality benefit  in future cancer screening RCTs.” | “A reduction in the absolute incidence of late-stage cancer diagnoses was correlated with a reduction in cancer-specific mortality across the majority of previous randomised controlled trials of cancer screening. In other words, a trial-level relationship between late-stage cancer incidence and mortality was evident. However, the evidence was based on correlation of a limited range of cancer types and screening tests, and limited data on late-stage incidence effects at an earlier time point than mortality results. Therefore, although the evidence supports further investigations into utilising tumour characteristics as surrogates in future evaluations of new cancer screening tests, and acting now to develop methodological guidance for including surrogates into future study designs, the interpretation of these data do not allow us to conclude that the incidence of late-stage disease can reliably replace disease-specific mortality for all cancers. Given the slow pace of starting and completing cancer screening trials, it would be prudent for trialists to act now to collect relevant stage and other prognostic tumour features so that these investigations can advance, and the potential would exist to act on these findings if they were sufficiently persuasive.” |
| **Funding** | Not disclosed (lead authors employed at IARC) | Grail | Cancer Research UK, National Institute for Health and Care Research |
| **Conflicts of interest** | None reported | Five authors employed by Grail. One author has multiple patents in the field of cancer detection pending to Grail. One author receiving consulting fees from Grail. | One author reports membership in the Grail advisory board and two report funding from Grail to run trials. One author reports consultancy to Grail and Roche. One author reports advisory board meetings for Hologic. |

**Abbreviations.** CI, confidence interval; IARC, International Agency for Research on Cancer; TNM, tumour-node-metastasis; USPSTF, US Preventive Services Task Force.

## Table S2. Trials included in the three meta-analyses exploring the relationship between late-stage cancer and cancer-specific mortality in cancer screening.

| **Cancer type** | **Trial name/acronym** | **Feng et al.** | **Dai et al.** | **Rebolj et al.** |
| --- | --- | --- | --- | --- |
| Bowel | Burgundy | X |  |  |
| Bowel | Finnish |  |  | X |
| Bowel | Funen | X | X | X |
| Bowel | Gothenburg | X | X | X |
| Bowel | Israel | X |  |  |
| Bowel | Minnesota | X | X | X |
| Bowel | NORCCAP | X | X | X |
| Bowel | NordICC | X | X | X |
| Bowel | Nottingham | X | X | X |
| Bowel | PLCO | X | X | X |
| Bowel | SCORE | X | X | X |
| Bowel | Telemark |  |  | X |
| Bowel | UKFSST |  |  | X |
|  | **Total** | **10** | **8** | **11** |
| Breast | CNBSS-1 | X |  | X |
| Breast | CNBSS-2 | X |  | X |
| Breast | Edinburgh | X |  | X |
| Breast | Gothenburg |  |  | X |
| Breast | HIP NY |  | X | X |
| Breast | Malmo | X | X | X |
| Breast | Mumbai | X |  | X |
| Breast | Russia/WHO |  |  | X |
| Breast | Shanghai |  |  | X |
| Breast | Stockholm |  | X | X |
| Breast | Trivandrum | X | X | X |
| Breast | Two-County |  | X | X |
| Breast | UK Age |  |  | X |
|  | **Total** | **6** | **5** | **13** |
| Cervical | Finnish |  |  | X |
| Cervical | Osmanabad |  |  | X |
| Cervical | Tamil Nadu |  |  | X |
| Cervical | Mumbai |  |  | X |
|  | **Total** | **0** | **0** | **4** |
| Liver | Qidong | X |  | X |
| Liver | Shanghai | X |  | X |
|  | **Total** | **2** | **0** | **2** |
| Lung | Czech Study |  | X | X |
| Lung | DANTE | X | X | X |
| Lung | DLCST | X | X | X |
| Lung | ITALUNG | X | X | X |
| Lung | Johns Hopkins |  | X | X |
| Lung | LSS |  |  | X |
| Lung | LUSI | X | X | X |
| Lung | Mayo Lung Project | X | X | X |
| Lung | Memorial Sloan Kettering | X | X | X |
| Lung | MILD | X |  | X |
| Lung | NELSON | X | X | X |
| Lung | NLST | X | X | X |
| Lung | PLCO | X | X | X |
| Lung | Scotland | X |  |  |
| Lung | UKLS | X | X | X |
|  | **Total** | **12** | **12** | **14** |
| Nasopharyngeal | China NPC | X | X | X |
|  | **Total** | **1** | **1** | **1** |
| Oral | Trivandrum | X | X | X |
|  | **Total** | **1** | **1** | **1** |
| Ovarian | PLCO | X | X | X |
| Ovarian | UKCTOCS | X | X | X |
| Ovarian | UKCTOCS pilot | X |  | X |
|  | **Total** | **3** | **2** | **3** |
| Prostate | CAP | X | X | X |
| Prostate | ERSPC | X | X | X |
| Prostate | ERSPC – French |  | X |  |
| Prostate | ERSPC pilot |  |  | X |
| Prostate | Norrkoping | X |  | X |
| Prostate | PLCO | X | X | X |
|  | **Total** | **4** | **4** | **5** |
| Multiple | D’Aquapendente |  |  | X |
| Multiple | MVTEP |  |  | X |
| Multiple | SOMIT |  |  | X |
|  | **Total** | **0** | **0** | **3** |
| **Any cancer** | **Total** | **39** | **33** | **57** |

Trials included in the Feng et al. meta-analysis, not in the present meta-analysis:

- Bowel – Burgundy. Excluded after “Search 2” as it was not a randomised controlled trial. Dai et al.: excluded, not a randomised trial.
- Bowel – Israel. Not identified in “Search 1” or supplementary searches, and unclear whether the control arm clusters were selected at random. Dai et al: excluded, not a randomised trial.
- Lung – Scotland. Identified in “Search 1”, excluded because it was missed by both reviewers at title/abstract level, and it was not identified in supplementary searches. Dai et al.: not mentioned.

Trials included in the Dai et al. meta-analysis, not in the present meta-analysis:

- ERSPC – French. The relevant publications from the French part of ERSPC were included in the collection of the literature on the entire ERSPC trial but a different publication (excluding the data from France) was considered more representative for inclusion in the primary analysis. Feng et al: not mentioned.

We did not compare the selection of specific publications by trial between the three meta-analyses because the meta-analyses prioritised different lengths of follow-up for their primary analyses:

- Feng et al: the earliest reported mortality endpoint.
- Dai et al.: the longest reported mortality endpoint.
- Rebolj et al.: “main” mortality endpoint (defined as one that was clearly identified as such by trialists in the original publications, in the trial protocol, or in the statistical analysis plan and the power calculations).

# Section 2. Systematic searches in bibliographic databases and study selection

The searches were undertaken in two stages. The first stage (“Search 1”) was to identify the relevant randomised controlled trials. The second stage (“Search 2”) was to identify the relevant publications from the trials identified in “Search 1” (late-stage outcomes or additional mortality outcomes not captured previously). If additional trials were identified at this stage, they were included in the review. Below we specify the criteria for searches in each stage, as well as the outcomes of each search and reasons for rejection of publications at the full text level.

**“Search 1”: To identify relevant randomised controlled trials in cancer screening reporting mortality outcomes**

## Table S3. Search strategy, eligibility criteria and review strategy in “Search 1” of the bibliographic search.

| Aim | To find all relevant RCTs in primary cancer screening reporting mortality outcomes in the intervention and control arms and published to date. |
| --- | --- |
| Search strategy | Pre-specified electronic search in bibliographical databases, using a list of keywords/terms compiled and tested by the study team, developed in collaboration with an expert librarian. |
| Supplemental searches | Contacting experts in the field,  most recent USPSTF cancer screening reviews (as identified by <https://www.uspreventiveservicestaskforce.org/uspstf/topic_search_results?topic_status=P&category%5B%5D=15&type%5B%5D=5&searchterm=>), IARC handbooks of cancer prevention (colorectal, breast, and cervical). |
| Review strategy | Titles and abstracts of the publications found by the search screened independently by two reviewers, consulting all full text publications considered potentially relevant by either reviewer;  full text articles assessed against the inclusion/exclusion criteria independently by two reviewers, with disagreements resolved by a third reviewer. |
| Inclusion criteria | PICOS (“Search 1”):  Population: people (any age: children or adults) without apparent symptoms of cancer that the trial is aiming to detect at a pre-symptomatic stage or to prevent, from the general population or from a higher cancer-risk group.  Intervention: a screening regimen for cancer of any type, with the screening intervention meeting the following criteria:   - Single or repeated test; - Test should be defined (including any cut-point) prior to the study; - The test may be performed in or outside of a health care facility (including e.g., self-sampling tests or self-examination); - There should be an agreed policy on further diagnostic investigation of individuals with a positive test result and on the choices available to those individuals.   Comparator: no screening or another type of screening.  Outcome: must report cancer-specific or all-cause mortality.  Study design: the test must have been studied in an individually or cluster randomised controlled trial. |
| Exclusion criteria | - Non-randomised studies; - Non-human studies; - Publications with no mortality outcomes reported; - Letters, reviews, editorials and communications with insufficient information on methods and/or no numerical outcomes data; - Grey literature and conference abstracts; - Articles not available in the English language. |
| Key outcome | List of eligible screening trials reporting mortality outcomes. |
| Documentation | PRISMA flow diagram of publications included and excluded at each stage of the review; reasons for exclusion of records at full text level will be documented. |

**Literature search in Medline, Embase and Web of Science in “Search 1”**

Ovid MEDLINE(R) ALL <1946 to September 21, 2022>

1 cancer*.mp. 2,155,014

2 neoplasm*.mp. 3,168,201

3 exp Neoplasms/ 3,737,394

4 1 or 2 or 3 4,385,892

5 screen*.mp. 990,088

6 exp Mass Screening/ 141,720

7 exp "Early Detection of Cancer"/ 34,964

8 5 or 6 or 7 1,008,832

9 exp Mortality/ or mortality.mp. 1,463,207

10 exp Randomized Controlled Trial/ or exp Random Allocation/ 667,865

11 randomized controlled trial.pt. 577,339

12 10 or 11 667,865

13 4 and 8 and 9 and 12 1106

Embase Classic+Embase <1947 to 2022 September 21>

1 cancer*.mp. 4,372,531

2 neoplasm*.mp. 983,532

3 exp neoplasm/ 5,648,200

4 1 or 2 or 3 6,467,573

5 screen*.mp. 1,612,332

6 exp mass screening/ 293,380

7 exp early cancer diagnosis/ 11,575

8 5 or 6 or 7 1620,653

9 mortality.mp. or exp cancer mortality/ or mortality/ 1,841,601

10 exp randomized controlled trial/ 733,200

11 random allocation.mp. or exp randomization/ 97,605

12 10 or 11 806,321

13 4 and 8 and 9 and 12 1987

14 limit 13 to (article or article in press or "review") 1160

Web of Science

<https://www.webofscience.com/wos/woscc/summary/f4a58e16-29cb-4e99-b809-0c2750a3b0ee-50bdb465/relevance/1>

**cancer* or neoplasm***(Topic) and**screen* or "early detect*"**(Topic) and**rct* or "randomized controlled trial*" or "random allocat*" or "randomised controlled trial*"**(Topic) and**mortality**

## Table S4. Number of records retrieved in “Search 1”.

| **Database searched** | **Date searched** | **Number of records retrieved** |
| --- | --- | --- |
| Medline | 21/09/2022 | 1106 |
| Embase | 21/09/2022 | 1160 |
| Web of Science | 21/09/2022 | 1701 |
| Total number of records retrieved: 3967  Duplicates removed (EndNote): 813  Final number for screening: 3154 | | |

## Table S5. Reason for exclusion of publications at full text level identified during “Search 1” (n = 105).

| **Citation** | **Reason for exclusion** |
| --- | --- |
| **No full text available (n = 1)** | |
| Schroder FH. The European Screening Study for Prostate Cancer. The Canadian journal of oncology. 1994;4 Suppl 1:102-9. | Exclude as no full text available |
| **Duplicate (n = 2)** | |
| Miller AB, To T, Baines CJ, Wall C. The Canadian national breast screening study-1: Breast cancer mortality after 11 to 16 years of follow-up: A randomized screening trial of mammography in women age 40 to 49 years. Annals of Internal Medicine. 2002;137(5 I):305-12. | Exclude as duplicate |
| Tabar L, Gad A, Holmberg LH. Reduction in mortality from breast cancer after mass screening with mammography. Lancet. 1985;1(8433):829-32. | Exclude as duplicate |
| **Intervention not relevant (n = 1)** | |
| Selby JV, Friedman GD, Collen MF. Sigmoidoscopy and mortality from colorectal cancer: the Kaiser Permanente Multiphasic Evaluation Study. Journal of clinical epidemiology. 1988;41(5):427-34. | Exclude on intervention |
| **Non-English language (n = 6)** | |
| Anonymous. Initial results from a randomized study in the counties of Kopparberg and Ostergotland (the WE project). Reduced mortality in breast cancer through health control with mammography. Nordisk medicin. 1985;100(6-7):175-8. | Exclude on language |
| Gao DL, Thomas DB, Ray RM, Wang WW, Allison CJ, Chen FL, et al. Randomized trial of breast self-examination in 266,064 women in Shanghai. Zhonghua zhong liu za zhi [Chinese journal of oncology]. 2005;27(6):350-4. | Exclude on language |
| Kronborg O, Fenger C, Olsen J, Jorgensen OD, Sondergaard O. Randomized population study of screening for intestinal cancer with Hemoccult-II. Ugeskrift for laeger. 1997;159(33):4977-81. | Exclude on language |
| Liu X, Zheng S, Ma X, Zhou L, Yu H, Yao K, et al. Randomized controlled trial of sequence mass screening program for colorectal cancer. Zhonghua liu xing bing xue za zhi = Zhonghua liuxingbingxue zazhi. 2000;21(6):430-3. | Exclude on language |
| Yang B, Zhang B, Tang Z. Randomized controlled prospective study of secondary prevention for primary liver cancer. Zhonghua yi xue za zhi. 1999;79(12):887-9. | Exclude on language |
| Zambrano GT, Galan ML, Mateo CP, Tello AG, Rodriguez N, Sanchez AB. Preliminary data of the Spanish contribution to the European Randomized Study on Screening of Prostate Cancer (ERSPC). Archivos Espanoles de Urologia. 2007;60(7):737-43. | Exclude on language |
| **No mortality outcomes reported (n = 48)** | |
| Anderson TJ, Alexander FE, Lamb J, Smith A, Forrest AP. Pathology characteristics that optimize outcome prediction of a breast screening trial. British journal of cancer. 2000;83(4):487-92. | Exclude on outcomes |
| Bergdahl AG, Aus G, Lilja H, Hugosson J. Risk of dying from prostate cancer in men randomized to screening: differences between attendees and nonattendees. Cancer. 2009;115(24):5672-9. | Exclude on outcomes |
| Blamey RW, Day NE, Young JR, Ellis IO, Pinder SE, Duffy SW, et al. The frequency of breast cancer screening: Results from the UKCCCR Randomised Trial. European Journal of Cancer. 2002;38(11):1458-64. | Exclude on outcomes |
| Bokhorst LP, Kranse R, Venderbos LDF, Salman JW, van Leenders GJLH, Schroder FH, et al. Differences in Treatment and Outcome After Treatment with Curative Intent in the Screening and Control Arms of the ERSPC Rotterdam. European urology. 2015;68(2):179-82. | Exclude on outcomes |
| Boniol M, Boyle P, Autier P, Ruffion A, Perrin P. Critical role of prostate biopsy mortality in the number of years of life gained and lost within a prostate cancer screening programme. BJU international. 2012;110(11):1648-52. | Exclude on outcomes |
| Breast Screening Frequency Trial G. The frequency of breast cancer screening: results from the UKCCCR Randomised Trial. United Kingdom Co-ordinating Committee on Cancer Research. European journal of cancer (Oxford, England : 1990). 2002;38(11):1458-64. | Exclude on outcomes |
| Burnell M, Gentry-Maharaj A, Ryan A, Apostolidou S, Habib M, Kalsi J, et al. Impact on mortality and cancer incidence rates of using random invitation from population registers for recruitment to trials. Trials. 2011;12:61. | Exclude on outcomes |
| Chiu SY-H, Duffy S, Yen AM-F, Tabar L, Smith RA, Chen H-H. Effect of baseline breast density on breast cancer incidence, stage, mortality, and screening parameters: 25-year follow-up of a Swedish mammographic screening. Cancer epidemiology, biomarkers & prevention : a publication of the American Association for Cancer Research, cosponsored by the American Society of Preventive Oncology. 2010;19(5):1219-28. | Exclude on outcomes |
| Chu KC, Connor RJ. Analysis of the temporal patterns of benefits in the Health Insurance Plan of Greater New York trial by stage and age. American journal of epidemiology. 1991;133(10):1039-49. | Exclude on outcomes |
| Ciatto S, Zappa M, Villers A, Paez A, Otto SJ, Auvinen A. Contamination by opportunistic screening in the European Randomized Study of Prostate Cancer Screening. BJU International, Supplement. 2003;92(2):97-100. | Exclude on outcomes |
| De Koning HJ, Blom J, Merkelbach JW, Raaijmakers R, Verhaegen H, Van Vliet P, et al. Determining the cause of death in randomized screening trial(s) for prostate cancer. BJU international. 2003;92 Suppl 2:71-8. | Exclude on outcomes |
| Doroudi M, Schoen RE, Pinsky PF. Early detection versus primary prevention in the PLCO flexible sigmoidoscopy screening trial: Which has the greatest impact on mortality? Cancer. 2017;123(24):4815-22. | Exclude on outcomes |
| Finne P, Stenman UH, Maattanen L, Makinen T, Tammela TLJ, Martikainen P, et al. The Finnish trial of prostate cancer screening: where are we now? BJU international. 2003;92 Suppl 2:22-6. | Exclude on outcomes |
| Frost JK, Ball WC, Jr., Levin ML, Tockman MS, Baker RR, Carter D, et al. Early lung cancer detection: results of the initial (prevalence) radiologic and cytologic screening in the Johns Hopkins study. The American review of respiratory disease. 1984;130(4):549-54. | Exclude on outcomes |
| Gao F, Chia KS, Ng FC, Ng EH, Machin D. Interval cancers following breast cancer screening in Singaporean women. International Journal of Cancer. 2002;101(5):475-9. | Exclude on outcomes |
| Halpern JA, Shoag JE, Mittal S, Oromendia C, Ballman KV, Hershman DL, et al. Prognostic Significance of Digital Rectal Examination and Prostate Specific Antigen in the Prostate, Lung, Colorectal and Ovarian (PLCO) Cancer Screening Arm. The Journal of urology. 2017;197(2):363-8. | Exclude on outcomes |
| Hatschek T, Grontoft O, Fagerberg G, Stal O, Sullivan S, Carstensen J, et al. Cytometric and histopathologic features of tumors detected in a randomized mammography screening program: correlation and relative prognostic influence. Breast cancer research and treatment. 1990;15(3):149-60. | Exclude on outcomes |
| Holmberg LH, Tabar L, Adami HO, Bergstrom R. Survival in breast cancer diagnosed between mammographic screening examinations. Lancet (London, England). 1986;2(8497):27-30. | Exclude on outcomes |
| Ikeda DM, Andersson I, Wattsgard C, Janzon L, Linell F. Interval carcinomas in the Malmo Mammographic Screening Trial: radiographic appearance and prognostic considerations. AJR American journal of roentgenology. 1992;159(2):287-94. | Exclude on outcomes |
| Jodal HC, Loberg M, Holme O, Adami H-O, Bretthauer M, Emilsson L, et al. Mortality From Postscreening (Interval) Colorectal Cancers Is Comparable to That From Cancer in Unscreened Patients-A Randomized Sigmoidoscopy Trial. Gastroenterology. 2018;155(6):1787-94.e3. | Exclude on outcomes |
| Kilpelainen TP, Talala K, Taari K, Raitanen J, Kujala P, Pylvalainen J, et al. Patients' education level and treatment modality for prostate cancer in the Finnish Randomized Study of Screening for Prostate Cancer. European journal of cancer (Oxford, England : 1990). 2020;130:204-10. | Exclude on outcomes |
| Kinnunen PTT, Murtola TJ, Talala K, Taari K, Tammela TLJ, Auvinen A. Anticoagulants and cancer mortality in the Finnish randomized study of screening for prostate cancer. Cancer causes & control : CCC. 2019;30(8):877-88. | Exclude on outcomes |
| Kinnunen PTT, Murtola TJ, Talala K, Taari K, Tammela TLJ, Auvinen A. Prostate cancer-specific survival among warfarin users in the Finnish Randomized Study of Screening for Prostate Cancer. BMC Cancer. 2017;17(1):585. | Exclude on outcomes |
| Koskenvuo L, Malila N, Pitkaniemi J, Miettinen J, Heikkinen S, Sallinen V. Sex differences in faecal occult blood test screening for colorectal cancer. The British journal of surgery. 2019;106(4):436-47. | Exclude on outcomes |
| Landy R, Houghton LC, Berg CD, Grubb RL, 3rd, Katki HA, Black A. Risk of Prostate Cancer-related Death Following a Low PSA Level in the PLCO Trial. Cancer prevention research (Philadelphia, Pa). 2020;13(4):367-76. | Exclude on outcomes |
| Mapp TJ, Hardcastle JD, Moss SM, Robinson MH. Survival of patients with colorectal cancer diagnosed in a randomized controlled trial of faecal occult blood screening. The British journal of surgery. 1999;86(10):1286-91. | Exclude on outcomes |
| Marcus PM, Bergstralh EJ, Zweig MH, Harris A, Offord KP, Fontana RS. Extended lung cancer incidence follow-up in the Mayo Lung Project and overdiagnosis. Journal of the National Cancer Institute. 2006;98(11):748-56. | Exclude on outcomes |
| Melamed MR, Flehinger BJ. Detection of lung cancer: highlights of the Memorial Sloan-Kettering Study in New York City. Schweizerische medizinische Wochenschrift. 1987;117(39):1457-63. | Exclude on outcomes |
| Melamed MR. Lung cancer screening results in the National Cancer Institute New York study. Cancer. 2000;89(11 Suppl):2356-62. | Exclude on outcomes |
| Murtola TJ, Virkku A, Talala K, Stenman U-H, Taari K, Tammela TLJ, et al. Outcomes of Prostate Cancer Screening by 5alpha-Reductase Inhibitor Use. The Journal of urology. 2017;198(2):305-9. | Exclude on outcomes |
| Nevalainen J, Stenman U-H, Tammela TL, Roobol M, Carlsson S, Talala K, et al. What explains the differences between centres in the European screening trial? A simulation study. Cancer epidemiology. 2017;46:14-9. | Exclude on outcomes |
| Patz EF, Jr., Greco E, Gatsonis C, Pinsky P, Kramer BS, Aberle DR. Lung cancer incidence and mortality in National Lung Screening Trial participants who underwent low-dose CT prevalence screening: a retrospective cohort analysis of a randomised, multicentre, diagnostic screening trial. The Lancet Oncology. 2016;17(5):590-9. | Exclude on outcomes |
| Pinsky PF, Black A, Parnes HL, Grubb R, David Crawford E, Miller A, et al. Prostate cancer specific survival in the Prostate, Lung, Colorectal, and Ovarian (PLCO) Cancer Screening Trial. Cancer epidemiology. 2012;36(6):e401-6. | Exclude on outcomes |
| Pinsky PF, Gierada DS, Hocking W, Patz EF, Jr., Kramer BS. National Lung Screening Trial findings by age: Medicare-eligible versus under-65 population. Annals of internal medicine. 2014;161(9):627-33. | Exclude on outcomes |
| Pinsky PF, Miller A, Kramer BS, Church T, Reding D, Prorok P, et al. Evidence of a healthy volunteer effect in the prostate, lung, colorectal, and ovarian cancer screening trial. American journal of epidemiology. 2007;165(8):874-81. | Exclude on outcomes |
| Prosper AE, Inoue K, Brown K, Bui AAT, Aberle D, Hsu W. Association of Inclusion of More Black Individuals in Lung Cancer Screening With Reduced Mortality. JAMA network open. 2021;4(8):e2119629. | Exclude on outcomes |
| Rojewski AM, Tanner NT, Dai L, Ravenel JG, Gebregziabher M, Silvestri GA, et al. Tobacco Dependence Predicts Higher Lung Cancer and Mortality Rates and Lower Rates of Smoking Cessation in the National Lung Screening Trial. Chest. 2018;154(1):110-8. | Exclude on outcomes |
| Saadatmand S, Geuzinge HA, Rutgers EJT, Mann RM, de Roy van Zuidewijn DBW, Zonderland HM, et al. MRI versus mammography for breast cancer screening in women with familial risk (FaMRIsc): a multicentre, randomised, controlled trial. The Lancet Oncology. 2019;20(8):1136-47. | Exclude on outcomes |
| Saarimaki L, Hugosson J, Tammela TL, Carlsson S, Talala K, Auvinen A. Impact of Prostatic-specific Antigen Threshold and Screening Interval in Prostate Cancer Screening Outcomes: Comparing the Swedish and Finnish European Randomised Study of Screening for Prostate Cancer Centres. European urology focus. 2019;5(2):186-91. | Exclude on outcomes |
| Sankaranarayanan R, Mathew B, Jacob BJ, Thomas G, Somanathan T, Pisani P, et al. Early findings from a community-based, cluster-randomized, controlled oral cancer screening trial in Kerala, India. The Trivandrum Oral Cancer Screening Study Group. Cancer. 2000;88(3):664-73. | Exclude on outcomes |
| Shapiro S, Strax P, Venet L. Evaluation of periodic breast cancer screening with mammography. Methodology and early observations. JAMA. 1966;195(9):731-8. | Exclude on outcomes |
| Shoag J, Mittal S, Halpern JA, Scherr D, Hu JC, Barbieri CE. Lethal Prostate Cancer in the PLCO Cancer Screening Trial. European urology. 2016;70(1):2-5. | Exclude on outcomes |
| Tabar L, Akerlund E, Gad A. Five-year experience with single-view mammography randomized controlled screening in Sweden. Recent results in cancer research Fortschritte der Krebsforschung Progres dans les recherches sur le cancer. 1984;90:105-13. | Exclude on outcomes |
| Tabar L, Duffy SW, Burhenne LW. New Swedish breast cancer detection results for women aged 40-49. Cancer. 1993;72(4 Suppl):1437-48. | Exclude on outcomes |
| Taghipour S, Banjevic D, Fernandes J, Miller AB, Montgomery N, Jardine AKS, et al. Predictors of competing mortality to invasive breast cancer incidence in the Canadian National Breast Screening study. BMC Cancer. 2012;12:299. | Exclude on outcomes |
| Temkin SM, Miller EA, Samimi G, Berg CD, Pinsky P, Minasian L. Outcomes from ovarian cancer screening in the PLCO trial: Histologic heterogeneity impacts detection, overdiagnosis and survival. European journal of cancer (Oxford, England : 1990). 2017;87:182-8. | Exclude on outcomes |
| Warwick J, Tabar L, Vitak B, Duffy SW. Time-dependent effects on survival in breast carcinoma: results of 20 years of follow-up from the Swedish Two-County Study. Cancer. 2004;100(7):1331-6. | Exclude on outcomes |
| Zhu X, van Leeuwen PJ, Bul M, Otto SJ, de Koning HJ, Bangma CH, et al. Disease-specific survival of men with prostate cancer detected during the screening interval: results of the European randomized study of screening for prostate cancer-Rotterdam after 11 years of follow-up. European urology. 2011;60(2):330-6. | Exclude on outcomes |
| **Letters, reviews, editorials and communications with insufficient information on methods and/or no numerical outcomes data; grey literature and conference abstracts (n = 23)** | |
| Black WC, Haggstrom DA, Welch HG. All-cause mortality in randomized trials of cancer screening. Journal of the National Cancer Institute. 2002;94(3):167-73. | Exclude on publication type |
| Dominioni L, Poli A, Mantovani W, Rotolo N, Imperatori A. Volunteer effect and compromised randomization in the Mayo Project of screening for lung cancer. European Journal of Epidemiology. 2011;26(1):79-80. | Exclude on publication type |
| Gupta S. Screening for Colorectal Cancer. Hematology-Oncology Clinics of North America. 2022;36(3):393-414. | Exclude on publication type |
| Jacobs IJ, Menon U, Ryan A, Gentry-Maharaj A, Burnell M, Kalsi JK, et al. Ovarian cancer screening and mortality in the UK collaborative trial of ovarian cancer screening (UKCTOCS): A randomised controlled trial. Cancer Research. 2016;76. | Exclude on publication type |
| Jacobs IJ, Menon U, Ryan A, Gentry-Maharaj A, Burnell M, Kalsi JK, et al. Ovarian Cancer Screening and Mortality in the UK Collaborative Trial of Ovarian Cancer Screening (UKCTOCS): A Randomized Controlled Trial. Obstetrical & Gynecological Survey. 2016;71(6):346-8. | Exclude on publication type |
| Kane KY, Lindbloom EJ, Stevermer JJ. Does mammography add any benefit to a thorough clinical breast examination (CBE)? The Journal of family practice. 2000;49(12):1078. | Exclude on publication type |
| Klein A, Shepshelovich D, Spectre G, Goldvaser H, Raanani P, Gafter-Gvili A. Screening for occult cancer in idiopathic venous thromboembolism Systemic review and meta-analysis. European Journal of Internal Medicine. 2017;42:74-80. | Exclude on publication type |
| Lacasse Y. Screening with chest radiography did not reduce lung cancer mortality in older patients. Annals of Internal Medicine. 2012;156(6):JC3-8. | Exclude on publication type |
| Menon U, Gentry-Maharaj A, Burnell M, Singh N, Ryan A, Karpinskyj C, et al. Ovarian Cancer Population Screening and Mortality After Long-Term Follow-up in the UK Collaborative Trial of Ovarian Cancer Screening (UKCTOCS): A Randomised Controlled Trial. Obstetrical & Gynecological Survey. 2021;76(9):537-8. | Exclude on publication type |
| Miller AB, Baines CJ, To T. The Gothenburg breast screening trial: first results on mortality, incidence, and mode of detection for women ages 39-49 years at randomization. Cancer. 1998;83(1):186-90. | Exclude on publication type |
| Miller ZA, Lee KS. Screening for hepatocellular carcinoma in high-risk populations. Clinical Imaging. 2016;40(2):311-4. | Exclude on publication type |
| Pavlik EJ. Ovarian cancer screening effectiveness: A realization from the UK Collaborative Trial of Ovarian Cancer Screening. Womens Health. 2016;12(5):475-9. | Exclude on publication type |
| Pejovic T, Nezhat F. Effect of Screening on Ovarian Cancer Mortality: The Prostate, Lung, Colorectal and Ovarian (PLCO) Cancer Screening Randomized Controlled Trial. Journal of Minimally Invasive Gynecology. 2011;18(6):823-5. | Exclude on publication type |
| Rajkumar SV, Hartmann LC. Screening mammography in women aged 40-49 years. Medicine. 1999;78(6):410-6. | Exclude on publication type |
| Roobol MJ, Bangma CH, Loeb S. Prostate-specific antigen screening can be beneficial to younger and at-risk men. Canadian Medical Association Journal. 2013;185(1):47-51. | Exclude on publication type |
| Saito H, Kudo S-E, Takahashi N, Yamamoto S, Kodama K, Nagata K, et al. Efficacy of screening using annual fecal immunochemical test alone versus combined with one-time colonoscopy in reducing colorectal cancer mortality: the Akita Japan population-based colonoscopy screening trial (Akita pop-colon trial). International journal of colorectal disease. 2020;35(5):933-9. | Exclude on publication type |
| Sankaranarayanan R, Dinshaw K, Nene BM, Ramadas K, Esmy PO, Jayant K, et al. Cervical and oral cancer screening in India. Journal of Medical Screening. 2006;13:S35-S8. | Exclude on publication type |
| Segnan N, Senore C, Bonelli LA, Andreoni B, Giliani O, Giacomin A, et al. Once-Only Sigmoidoscopy Screening for Colorectal Cancer: Incidence and Mortality Follow-up of the Italian Randomized Controlled Trial (SCORE). Gastroenterology. 2011;140(5):S15-S. | Exclude on publication type |
| Shastri SS, Mittra I, Mishra G, Gupta S, Dikshit R, Badwe RA. Effect of visual inspection with acetic acid (VIA) screening by primary health workers on cervical cancer mortality: A cluster randomized controlled trial in Mumbai India. Journal of Clinical Oncology. 2013;31(15). | Exclude on publication type |
| Shastri SS, Mittra I, Mishra G, Gupta S, Dikshit R, Badwe RA. Effect of visual inspection with acetic acid (VIA) screening by primary health workers on cervical cancer mortality: A cluster randomized controlled trial in Mumbai, India. Journal of Clinical Oncology. 2013;31(18). | Exclude on publication type |
| Shastri SS. Effect of screening by clinical breast examination on breast cancer incidence and mortality after 20 years: prospective, cluster randomised controlled trial in Mumbai (vol 372, n256, 2021). Bmj-British Medical Journal. 2021;372. | Exclude on publication type |
| Tabar L, Gad A, Homberg L, Ljungquist U. PERIODIC SCREENING WITH ONE-VIEW MAMMOGRAPHY - THE EFFECT OF MORTALITY FROM BREAST-CANCER IN A RANDOMIZED, CONTROLLED TRIAL. British Journal of Radiology. 1986;59(702):640-. | Exclude on publication type |
| Verbeek AL, Hendriks JH, Holland R, Mravunac M, Sturmans F, Day NE, et al. Screening and breast cancer. Lancet (London, England). 1984;2(8404):690. | Exclude on publication type |
| **Non-randomised studies (n = 9)** | |
| Bul M, van Leeuwen PJ, Zhu X, Schroder FH, Roobol MJ. Prostate cancer incidence and disease-specific survival of men with initial prostate-specific antigen less than 3.0 ng/ml who are participating in ERSPC Rotterdam. European urology. 2011;59(4):498-505. | Exclude on study design |
| Chiles C, Duan F, Gladish GW, Ravenel JG, Baginski SG, Snyder BS, et al. Association of Coronary Artery Calcification and Mortality in the National Lung Screening Trial: A Comparison of Three Scoring Methods. Radiology. 2015;276(1):82-90. | Exclude on study design |
| Faivre J, Dancourt V, Lejeune C, Tazi MA, Lamour J, Gerard D, et al. Reduction in colorectal cancer mortality by fecal occult blood screening in a French controlled study. Gastroenterology. 2004;126(7):1674-80. | Exclude on study design |
| Ko CW, Sonnenberg A. Comparing risks and benefits of colorectal cancer screening in elderly patients. Gastroenterology. 2005;129(4):1163-70. | Exclude on study design |
| Lenner P, Jonsson H. Excess mortality from breast cancer in relation to mammography screening in northern Sweden. Journal of Medical Screening. 1997;4(1):6-9. | Exclude on study design |
| Lundgren P-O, Kjellman A, Norming U, Gustafsson O. Long-Term Outcome of a Single Intervention Population Based Prostate Cancer Screening Study. The Journal of urology. 2018;200(1):82-8. | Exclude on study design |
| McMahon PM, Chung YK, Johnson BE, Weinstein MC, Weeks JC, Kuntz KM, et al. Estimating long-term effectiveness of lung cancer screening in the Mayo CT screening study. Radiology. 2008;248(1):278-87. | Exclude on study design |
| Tabar L, Dean PB, Chen THH, Yen AMF, Chen SLS, Fann JCY, et al. The incidence of fatal breast cancer measures the increased effectiveness of therapy in women participating in mammography screening. Cancer. 2019;125(4):515-23. | Exclude on study design |
| Verbeek AL, Hendriks JH, Holland R, Mravunac M, Sturmans F, Day NE. Reduction of breast cancer mortality through mass screening with modern mammography. First results of the Nijmegen project, 1975-1981. Lancet (London, England). 1984;1(8388):1222-4. | Exclude on study design |
| **Disagreement between the two reviewers; excluded after discussion (n = 15)** | |
| Alexander F, Roberts MM, Lutz W, Hepburn W. Randomisation by cluster and the problem of social class bias. Journal of epidemiology and community health. 1989;43(1):29-36. | Excluded after discussion:  Overall mortality analysed to determine self-selection bias rather than the screening effect. |
| Bokhorst LP, Venderbos LDF, Schroder FH, Bangma CH, Steyerberg EW, Roobol MJ. Do Treatment Differences between Arms Affect the Main Outcome of ERSPC Rotterdam? The Journal of urology. 2015;194(2):336-42. | Excluded after discussion:  Not a primary analysis of the effect of screening on mortality. |
| Carlsson SV, Mansson M, Moss S, Kwiatkowski M, Recker F, Tammela TLJ, et al. Could Differences in Treatment Between Trial Arms Explain the Reduction in Prostate Cancer Mortality in the European Randomized Study of Screening for Prostate Cancer? European urology. 2019;75(6):1015-22. | Excluded after discussion:  Not a primary analysis of the effect of screening on mortality. |
| Duffy SW, Tabar L, Olsen AH, Vitak B, Allgood PC, Chen TH, et al. Absolute numbers of lives saved and overdiagnosis in breast cancer screening, from a randomized trial and from the Breast Screening Programme in England. Journal of medical screening. 2010;17(1):25-30. | Excluded after discussion:  We have identified several other mortality publications from this trial. |
| Grenabo Bergdahl A, Holmberg E, Moss S, Hugosson J. Incidence of prostate cancer after termination of screening in a population-based randomised screening trial. European urology. 2013;64(5):703-9. | Excluded after discussion:  Does not look at the effect of screening from the time of randomisation but compares the numbers of deaths only after screening has ended. |
| Jensen BM, Kronborg O, Fenger C. Interval cancers in screening with fecal occult blood test for colorectal cancer. Scandinavian journal of gastroenterology. 1992;27(9):779-82. | Excluded after discussion:  We have identified several mortality publications from this trial. |
| Kranse R, van Leeuwen PJ, Hakulinen T, Hugosson J, Tammela TL, Ciatto S, et al. Excess all-cause mortality in the evaluation of a screening trial to account for selective participation. Journal of medical screening. 2013;20(1):39-45. | Excluded after discussion:  This is a reanalysis of the data with a more complex statistical model. |
| Nixon R, Prevost TC, Duffy SW, Tabar L, Vitak B, Chen HH. Some random-effects models for the analysis of matched-cluster randomised trials: application to the Swedish two-county trial of breast-cancer screening. Journal of epidemiology and biostatistics. 2000;5(6):349-58. | Excluded after discussion:  This is a reanalysis of the data with a more complex statistical model. |
| Otto SJ, Schroder FH, de Koning HJ. Low all-cause mortality in the volunteer-based Rotterdam section of the European randomised study of screening for prostate cancer: self-selection bias? Journal of medical screening. 2004;11(2):89-92. | Excluded after discussion:  The deaths counted in this trial have nothing to do with the screening effect (follow-up is 2.8 years on average). They are presented to help (future) interpretation of any screening effect. |
| Pinsky PF, Zhu C, Skates SJ, Black A, Partridge E, Buys SS, et al. Potential effect of the risk of ovarian cancer algorithm (ROCA) on the mortality outcome of the Prostate, Lung, Colorectal and Ovarian (PLCO) trial. International journal of cancer. 2013;132(9):2127-33. | Excluded after discussion:  Although mortality rates were presented, this reanalysis used a different cut-off for the screening test and that different cut-off point was not used for the management of women. |
| Strauss GM, Gleason RE, Sugarbaker DJ. Screening for lung cancer re-examined. A reinterpretation of the Mayo Lung Project randomized trial on lung cancer screening. Chest. 1993;103(4 Suppl):337S-41S. | Excluded after discussion:  We have other records for this trial with the same/similar follow-up time and better description of the methods and data. Not a publication by the trial team. |
| Tabar L, Chen HH, Fagerberg G, Duffy SW, Smith TC. Recent results from the Swedish Two-County Trial: the effects of age, histologic type, and mode of detection on the efficacy of breast cancer screening. Journal of the National Cancer Institute Monographs. 1997(22):43-7. | Excluded after discussion:  We have other records for this trial with the same/similar follow-up time. |
| Tsodikov A, Gulati R, Heijnsdijk EAM, Pinsky PF, Moss SM, Qiu S, et al. Reconciling the effects of screening on prostate cancer mortality in the ERSPC and PLCO trials. Annals of Internal Medicine. 2017;167(7):449-55. | Excluded after discussion:  Both trials were already included in our lists with comparable lengths of follow-up. |
| van Leeuwen PJ, Connolly D, Gavin A, Roobol MJ, Black A, Bangma CH, et al. Prostate cancer mortality in screen and clinically detected prostate cancer: estimating the screening benefit. European journal of cancer (Oxford, England : 1990). 2010;46(2):377-83. | Excluded after discussion:  Control data are from Northern Ireland, not from the ERSPC. |
| Whynes DK, Mangham CM, Balfour TW, Scholefield JH. Analysis of deaths occurring within the Nottingham trial of faecal occult blood screening for colorectal cancer. Gut. 2010;59(8):1088-93. | Excluded after discussion:  We have other publications from the same trial that looked at the mortality for intervention vs. control arm, for similar length of the follow-up. Another reason was that the paper doesn't report absolute numbers but only proportions among all deaths. |

Reasons for exclusion. **No full text available**: we could not find a copy of the full text of the publication. **Duplicate**: the same abstract was included twice for full text assessment i.e., it was identified from different databases and not picked up as a duplicate at the de-duplication stage. **Intervention not relevant**: the intervention was not invitation or no invitation to screening with a well-defined test, but study group subjects were urged to have annual multiphasic health checkups, with screening tests not being defined. **Non-English language**: the report was not published in English. **No mortality outcomes reported**: no relevant mortality outcomes reported for all arms (all-cause or cancer-specific). **Letters, reviews, editorials and communications with insufficient information on methods and/or no numerical outcomes data; grey literature and conference abstracts**: included letters, reviews, editorials, and communications with insufficient information on methods and/or no numerical outcomes data; grey literature and conference abstracts. **Non-randomised studies**: not a randomised controlled trial. **Disagreement between the two reviewers; excluded after discussion**: these are full texts where the two reviewers (MR/JG) initially disagreed on the inclusion but agreed on the exclusion after a discussion.

**“Search 2”: Publications reporting intermediate outcomes (or additional mortality outcomes) from trials identified in “Search 1”**

## Table S6. Search strategy, eligibility criteria and review strategy in “Search 2” of the bibliographic search.

| Aim | To find all relevant publications to date from the trials identified in “Search 1” that report intermediate outcomes that might be considered as surrogates or further mortality outcomes. |
| --- | --- |
| Search strategy | Pre-specified electronic search in bibliographical databases using identifying terms for each trial, developed in collaboration with an expert librarian. |
| Supplemental searches | If, at the data extraction stage, the text of an included record mentions a reference to another publication with mortality and/or intermediate outcome data that was missed by our search, we will assess it for inclusion and, if deemed relevant, extract the data. |
| Review strategy | Titles and abstracts of the publications found by the search screened independently by two reviewers, consulting all full text publications considered potentially relevant by either reviewer;  full text articles assessed against the inclusion/exclusion criteria independently by two reviewers, with disagreements resolved by a third reviewer. |
| Key criteria for selection of relevant publications | As “Search 1”, except  PICOS Outcome (“Search 2”): Presenting at least one intermediate outcome in the intervention and the control arm (potential surrogates #1-4 from the pre-specified list) or further cancer-specific or all-cause mortality outcomes. |
| Exclusion criteria | As “Search 1”, except  Publications with no intermediate outcomes in the intervention and control arms (potential surrogates #1-4 from the pre-specified list) or mortality outcomes reported. |
| Key outcome | 1. List of trials with a full list of their publications reporting on observed mortality and intermediate endpoints to date; 2. Numbering of each publication for reference throughout the project (publication IDs); 3. List of trials reporting mortality but not also intermediate outcomes. |
| Documentation | PRISMA flow diagram of publications included and excluded at each stage of the review; reasons of records excluded at full text level will be documented. |

The pre-specified protocol included more potential surrogate outcomes than reported in this manuscript. These were as follows (the # numbers are the same as used in Tables S6 and S9):

- #1: absolute incidence of late-stage target cancer;
- #2: predicted mortality;
- #3: proportion of target cancers diagnoses at a late stage;
- #4: absolute incidence of early-stage target cancer;
- #5: proportion of target cancers that are screen-detected;
- #6: proportion of high-grade target cancers that are screen-detected; and
- #7: diagnostic yield of screening.

**Literature search in Medline and Embase in “Search 2”**

“Search 2” comprised of a series of mini-searches based on the individual trials found from “Search 1” (or identified when screening records from other trials as part of “Search 2”).

Medline (OVID) search template:

[screen*.mp. OR exp Mass Screening/ OR exp "Early Detection of Cancer"/]

AND

[exp Random Allocation/ OR exp Randomized Controlled Trial/ OR randomized controlled trial.pt.]

AND

[Cancer type]

AND

[Screening tool]

AND

[Geographical location]

These results were ORed with the trial name or code/number.

Embase search template:

[screen*.mp. OR exp Mass Screening/ OR exp early cancer diagnosis/]

AND

[random*.mp. OR exp Randomized Controlled Trial/ OR exp randomization]

AND

[Cancer type]

AND

[Screening tool]

AND

[Geographical location]

These results were ORed with the trial name or code/number.

If, for any trial, the resulting number of publications was higher than 200, we limited the selection to papers authored by at least one of the main investigators on the trial (minimum two names, but for most trials this was three or higher).

## Table S7. Author names used to limit number of records in “Search 2” in case the resulting number of publications in a trial was higher than 200, by trial.

| **Trial** | **Author names used to limit number of records** |
| --- | --- |
| CNBSS | Miller, Baines, To, Wall |
| ERSPC | Lujan, Berenguer, Villers, Bessaoud, Soulié, Bokhorst; Schröder, Schroder, Roobol, de Koning, Bangma, Hakama, Hugosson, Moss; Zappa; Hakulinen; Auvinen; Ciatto; Tammela; Kwiatkowski; Lilja |
| HIP NY | Shapiro, Roeser, Strax, Egan, Venet, Shimkin |
| Memorial Sloan-Kettering | Melamed, Flehinger, Zaman |
| NLST | Aberle, Pinsky, Berg, Sicks, Marcus, National Lung Screening Trial Research Team |
| PLCO | Pinsky, Prorok, Miller, Berg, Gohagan, Andriole, Kramer, Buys, Schoen, Oken, Hayes |
| Stockholm Breast | Frisell, Lidbrink, Rutqvist |
| Two-County | Tabar, Chen, Duffy, Smith, Fagerberg, Gröntoft |
| UKCTOCS and pilot | Jacobs, Menon, Skates, Oram |

The results of these searches were combined into a single file and de-duplicated for each trial.

The table below reports the deduplicated results for each trial with database source (Embase or Medline). As half of the original Medline search results (prior to deduplication) were inadvertently overwritten by mistake, the original number retrieved is not available.

## Table S8. Deduplicated results from “Search 1” and “Search 2”, by trial.

| **Trial** | **Final #**  **deduplicated** | **Embase –**  **# deduplicated** | **Medline –**  **# deduplicated** |
| --- | --- | --- | --- |
| **Search of trials identified via “Search 1”:**  **Search date for both databases: 11/01/2023** | | | |
| Burgundy | 43 | 32 | 11 |
| CAP Prostate UK | 22 | 4 | 18 |
| China Nasopharynx | 17 | 13 | 4 |
| CNBSS | 228 | 79 | 149 |
| CNBSS limited by author names | 113 | 23 | 90 |
| Czech Study | 10 | 4 | 6 |
| DANTE | 41 | 23 | 18 |
| DLCST | 62 | 6 | 56 |
| Edinburgh | 43 | 21 | 22 |
| ERSPC Europe | 133 | 91 | 42 |
| ERSCP Finland | 48 | 6 | 42 |
| ERSPC France | 8 | 7 | 1 |
| ESPRC Netherlands | 158 | 81 | 77 |
| ESPRC Spain | 2 | 1 | 1 |
| ERSPC Combined | 793 | 208 | 585 |
| ERSPC Combined limited by author names | 458 | 85 | 373 |
| Finnish Cervix | 27 | 16 | 11 |
| Finnish Colorectal | 20 | 8 | 12 |
| Funen | 82 | 64 | 18 |
| Gothenburg Breast | 161 | 85 | 76 |
| Gothenburg Colorectal | 45 | 34 | 11 |
| Gothenburg Prostate | 106 | 61 | 45 |
| HIP NY | 204 | 78 | 126 |
| HIP NY limited by author names | 38 | 21 | 17 |
| Italung | 62 | 17 | 45 |
| Jiashan | 56 | 41 | 15 |
| John Hopkins | 15 | 1 | 14 |
| Johns/Sloan | 22 | 6 | 16 |
| LSS | 75 | 4 | 71 |
| LUSI | 104 | 98 | 6 |
| Malmo | 164 | 84 | 80 |
| Mayo Lung Project | 56 | 13 | 43 |
| Memorial Sloan-Kettering | 276 | 8 | 268 |
| Memorial Sloan-Kettering limited by author names | 12 | 1 | 11 |
| MILD | 60 | 19 | 41 |
| Minnesota | 42 | 22 | 20 |
| Mumbai Breast | 45 | 37 | 8 |
| Mumbai Cervix | 70 | 48 | 22 |
| Mumbai Combined | 115 | 85 | 30 |
| NELSON | 131 | 69 | 62 |
| NLST | 713 | 641 | 72 |
| NLST limited by author names | 114 | 43 | 71 |
| NORCAPP | 88 | 37 | 51 |
| Norrkoping Prostate | 84 | 49 | 35 |
| Nottingham | 44 | 14 | 30 |
| Osmanabad Cervix | 78 | 59 | 19 |
| PLCO | 836 | 62 | 774 |
| PLCO limited by author names | 361 | 10 | 351 |
| Qidong Liver | 33 | 29 | 4 |
| Quebec Prostate | 49 | 40 | 9 |
| SCORE | 127 | 99 | 28 |
| Shanghai Liver | 36 | 29 | 7 |
| SOMIT | 65 | 59 | 14 |
| Stockholm Breast | 227 | 113 | 114 |
| Stockholm Breast limited by author names | 29 | 8 | 21 |
| Tamil Nadu Cervix | 66 | 48 | 18 |
| Telemark | 64 | 31 | 33 |
| Trivandrum Breast | 49 | 30 | 19 |
| Trivandrum Oral | 17 | 8 | 9 |
| Two County | 213 | 83 | 130 |
| Two County limited by author names | 83 | 21 | 62 |
| UK Age Trial | 177 | 84 | 93 |
| UKCTOCS and pilot | 285 | 152 | 133 |
| UKCTOCS and pilot limited by author names | 110 | 25 | 85 |
| UKFSST | 166 | 134 | 32 |
| UKLS | 38 | 30 | 8 |
| **Searches for additional trials identified via “Search 2” only:**  **Search date for both databases: 07/03/2023 (all but Russia/WHO) and 28/04/2023 (Russia/WHO)** | | | |
| D'Acquapendente | 4 | 2 | 2 |
| MVTEP | 9 | 4 | 5 |
| NordICC | 126 | 59 | 67 |
| Russia/WHO | 13 | 7 | 6 |
| Shanghai Breast | 32 | 8 | 24 |
| SOME | 7 | 2 | 5 |

## Table S9. Reason for exclusion of publications at full text level identified during “Search 2” (n = 428).

| **Citation** | **Reason for exclusion** |
| --- | --- |
| **Conference abstract (n = 10)** | |
| Fagerberg G. Experience from randomized controlled breast screening with mammography in Ostergotland county, Sweden: a preliminary report. Recent results in cancer research Fortschritte der Krebsforschung Progres dans les recherches sur le cancer. 1984;90:117. | Exclude - Conference abstract |
| Hoff G, Grotmol T, Eva S, Bretthauer M. First randomised trial on the risk of colorectal cancer after flexible sigmoidoscopy screening. Gastroenterology. 2009;136(5 SUPPL. 1):A55. | Exclude - Conference abstract |
| Holme O, Loberg M, Kalager M, Bretthauer M, Aas E, Hoff G. Effect of flexible sigmoidoscopy on incidence and mortality from colorectal cancer; first largescale populaton-based trial. United European Gastroenterology Journal. 2013;1(1 SUPPL. 1):A69. | Exclude - Conference abstract |
| Holme O, Loberg M, Kalager M, Bretthauer M, Hernan MA, Aas E, et al. Long-term effectiveness of sigmoidoscopy screening on colorectal cancer incidence and mortality in women and men: A randomized trial. United European Gastroenterology Journal. 2017;5(8):1143. | Exclude - Conference abstract |
| Jodal HC, Loberg M, Adami HO, Bretthauer M, Hoff G, Kalager M. Interval colorectal cancers after screening-more aggressive than clinical cancers? Gastroenterology. 2016;150(4 SUPPL. 1):S451. | Exclude - Conference abstract |
| Piccioli A, Bernardi E, Dalla Valle F, Visona A, Tropeano PF, Bova C, et al. The value of ct scanning for the detection of occult cancer in patients with idiopathic VTE. Blood Transfusion. 2012;10(SUPPL. 4):s139. | Exclude - Conference abstract |
| Piccioli A, Bernardi E, Dalla Valle F, Visona A, Tropeano PF, Bova C, et al. The value of thoraco-abdominal CT scanning for the detection of occult cancer in patients with unprovoked venous thromboembolism. A randomized study. Thrombosis Research. 2012;129(SUPPL. 1):S164. | Exclude - Conference abstract |
| Tabar L, Fagerberg G, Day NE, Duffy SW. The Swedish two-county trial of mammographic screening for breast cancer: recent results on mortality and tumor characteristics. Pathologie-biologie. 1992;39(9):846. | Exclude - Conference abstract |
| Thiis-Evensen E, Hoff G. Once-only colonoscopy screening for prevention of colorectal cancer-11 years of follow-up. Gastroenterology. 2009;136(5 SUPPL. 1):A627-A8. | Exclude - Conference abstract |
| Thiis-Evensen E, Kalager M, Bretthauer M, Hoff G. First randomized trial of colonoscopy screening for the prevention of colorectal cancer. Gastrointestinal Endoscopy. 2011;73(4 SUPPL. 1):AB159. | Exclude - Conference abstract |
| **Duplicate (n = 7)** | |
| Autier P, Hery C, Haukka J, Boniol M, Byrnes G. Advanced breast cancer and breast cancer mortality in randomized controlled trials on mammography screening. Journal of Clinical Oncology. 2009;27(35):5919-23. | Exclude - Duplicate |
| Juul FE, Cross AJ, Schoen RE, Senore C, Pinsky P, Miller E, et al. 15-Year Benefits of Sigmoidoscopy Screening on Colorectal Cancer Incidence and Mortality. Annals of Internal Medicine. 2022;175(11):1525-33. | Exclude - Duplicate |
| Robin P, Le Roux PY, Planquette B, et al. Limited screening with versus without <sup>18</sup>F-fluorodeoxyglucose PET/CT for occult malignancy in unprovoked venous thromboembolism: An open-label randomised controlled trial. The Lancet Oncology 2016;17(2):193-99. doi: https://dx.doi.org/10.1016/S1470-2045%2815%2900480-5 | Exclude - Duplicate |
| Shapiro S. Evaluation of periodic breast cancer screening with mammography. methodology and early observations. JAMA (Chicago, Ill). 1966;195(9):440-52. | Exclude - Duplicate |
| Strax P, Venet L, Shapiro S, Gross S. Mammography and clinical examination in mass screening for cancer of the breast. Cancer (Philad ). 1967;20(12):388-93. | Exclude - Duplicate |
| Strax P, Venet L, Shapiro S. Mass screening in mammary cancer. Cancer. 1969;23(4):92-7. | Exclude - Duplicate |
| Tabar L, Gad A, Holmberg LH. Reduction in mortality from breast cancer after mass screening with mammography. Lancet. 1985;1(8433):829-32. | Exclude - Duplicate |
| **Letter/comment/review without additional data (n = 85)** | |
| Al-Shamsi HO. Mammography screening for breast cancer-the UK Age trial. The Lancet Oncology. 2020;21(11):e505. | Exclude - Letter/comment/review without additional data |
| Anonymous. Corrigendum to: Effect of VIA Screening by Primary Health Workers: Randomized Controlled Study in Mumbai, India. Journal of the National Cancer Institute. 2022;114(3):480. | Exclude - Letter/comment/review without additional data |
| Anonymous. Effect of screening by clinical breast examination on breast cancer incidence and mortality after 20 years: prospective, cluster randomised controlled trial in Mumbai. BMJ (Clinical research ed). 2021;372:n738. | Exclude - Letter/comment/review without additional data |
| Anonymous. Once-only flex sig decreases 11-year cancer mortality. Journal of the National Medical Association. 2010;102(10):967-8. | Exclude - Letter/comment/review without additional data |
| Baines CJ. Evaluating of mammography and physical examination as independent screening modalities in the Canadian National Breast Screening Study. Practical modalities of an efficient screening for breast cancer in the European Community: proceedings of an International Symposium of the Association Against Cancer (Belgium) ICS865. 1989:3-9. | Exclude - Letter/comment/review without additional data |
| Barratt AL, Coates AS. Screening decreases prostate cancer death: first analysis of the 1988 Quebec Prospective Randomized Controlled Trial. The Medical journal of Australia. 2004;181(4):213-4. | Exclude - Letter/comment/review without additional data |
| Basu P, Zhang L, Hariprasad R, et al. A pragmatic approach to tackle the rising burden of breast cancer through prevention & early detection in countries 'in transition'. Indian Journal of Medical Research 2020;152(4):343-55. doi: https://dx.doi.org/10.4103/ijmr.IJMR_1868_19 | Exclude - Letter/comment/review without additional data |
| Bell KJL, Irwig L, Nickel B, Hersch J, Hayen A, Barratt A. Mammography screening for breast cancer-the UK Age trial. The Lancet Oncology. 2020;21(11):e504. | Exclude - Letter/comment/review without additional data |
| Burki TK. Flexible sigmoidoscopy screening for colorectal cancer. The Lancet Oncology. 2014;15(10):e425. | Exclude - Letter/comment/review without additional data |
| Cameron D. Mammographic screening does not result in a reduction in breast cancer mortality when added to annual clinical breast examinations. Breast Cancer Research. 2000;2(1):66701. | Exclude - Letter/comment/review without additional data |
| Carrier M. The author replies. New England Journal of Medicine. 2015;373(25):2475. | Exclude - Letter/comment/review without additional data |
| Crawford ED, Miller GJ, Labrie F, Hirano D, Batuello J, Glode LM. Prostate cancer pathology, screening, and epidemiology. Reviews in urology. 2001;3 Suppl 2:S2-S10. | Exclude - Letter/comment/review without additional data |
| Cuzick J, Cafferty FH, Edwards R, Moller H, Duffy SW. Surrogate endpoints for cancer screening trials: general principles and an illustration using the UK Flexible Sigmoidoscopy Screening Trial. Journal of medical screening. 2007;14(4):178-85. | Exclude - Letter/comment/review without additional data |
| Darrason M, Grolleau E, De Bermont J, Couraud S. UKLS trial: looking beyond negative results. The Lancet regional health Europe. 2021;10:100184. | Exclude - Letter/comment/review without additional data |
| Di Nisio M, Otten HM, Piccioli A, Lensing AWA, Prandoni P, Buller HR, et al. Decision analysis for cancer screening in idiopathic venous thromboembolism. Journal of thrombosis and haemostasis : JTH. 2005;3(11):2391-6. | Exclude - Letter/comment/review without additional data |
| Dietze E, Jones V, Seewaldt V. Breast Self-Examination: the Case for a Second Look. Current Breast Cancer Reports 2020;12(2):118-24. doi: https://dx.doi.org/10.1007/s12609-020-00358-z | Exclude - Letter/comment/review without additional data |
| Dominioni L, Poli A, Mantovani W, Rotolo N, Imperatori A. Volunteer effect and compromised randomization in the Mayo Project of screening for lung cancer. European journal of epidemiology. 2011;26(1):79-80. | Exclude - Letter/comment/review without additional data |
| Donzelli A, Giudicatti G, Sghedoni D. Mammography screening for breast cancer-the UK Age trial. The Lancet Oncology. 2020;21(11):e509. | Exclude - Letter/comment/review without additional data |
| Duffy SW, Sasieni PD. Mammography screening for breast cancer-the UK Age trial - Authors' reply. The Lancet Oncology. 2020;21(11):e510. | Exclude - Letter/comment/review without additional data |
| Farley SJ. Prostate cancer: PSA-based screening in Sweden dramatically reduces disease-specific mortality. Nature Reviews Urology. 2010;7(8):415. | Exclude - Letter/comment/review without additional data |
| Frost JK, Ball WC, Jr., Levin ML, Tockman MS, Erozan YS, Gupta PK, et al. Sputum cytopathology: use and potential in monitoring the workplace environment by screening for biological effects of exposure. Journal of occupational medicine : official publication of the Industrial Medical Association. 1986;28(8):692-703. | Exclude - Letter/comment/review without additional data |
| Gaskie S, Nashelsky J. Are breast self-exams or clinical exams effective for screening breast cancer? Journal of Family Practice 2005;54(9):803-04. | Exclude - Letter/comment/review without additional data |
| Gaskie S, Nashelsky J. Are breast self-exams or clinical exams effective for screening breast cancer? Journal of Family Practice 2005;54(9):803-04. | Exclude - Letter/comment/review without additional data |
| Gera R, Michell MJ, Mokbel K. Mammography screening for breast cancer-the UK Age trial. The Lancet Oncology. 2020;21(11):e507. | Exclude - Letter/comment/review without additional data |
| Gohagan JK, Prorok PC, Greenwald P, Kramer BS. The PLCO Cancer Screening Trial: Background, Goals, Organization, Operations, Results. Reviews on recent clinical trials. 2015;10(3):173-80. | Exclude - Letter/comment/review without additional data |
| Grubb RL, Pinsky P, Prorok PC, Andriole GL. Screening for cancer: lessons learned from the prostate, lung, colorectal, and ovarian cancer screening trial. European urology. 2015;68(4):545-6. | Exclude - Letter/comment/review without additional data |
| Hardcastle J. Randomized control trial of faecal occult blood screening for colorectal cancer: results for the first 144,103 patients. European journal of cancer prevention : the official journal of the European Cancer Prevention Organisation (ECP). 1991;1 Suppl 2:21. | Exclude - Letter/comment/review without additional data |
| Haug U. Flexible sigmoidoscopy screening for colorectal cancer. BMJ (Online). 2017;356:j75. | Exclude - Letter/comment/review without additional data |
| Heleno B, Siersma V, Brodersen J. Estimation of Overdiagnosis of Lung Cancer in Low-Dose Computed Tomography Screening: A Secondary Analysis of the Danish Lung Cancer Screening Trial. JAMA internal medicine. 2018;178(10):1420-2. | Exclude - Letter/comment/review without additional data |
| Holme O, Schoen RE, Senore C, Segnan N, Hoff G, Loberg M, et al. Effectiveness of flexible sigmoidoscopy screening in men and women and different age groups: Pooled analysis of randomised trials. BMJ (Online). 2017;356:i6673. | Exclude - Letter/comment/review without additional data |
| Hugosson J. Stopping screening, when and how? Translational andrology and urology. 2018;7(1):46-53. | Exclude - Letter/comment/review without additional data |
| Imperiale TF. Sigmoidoscopy screening for colorectal cancer. BMJ (Clinical research ed). 2009;338:b2084. | Exclude - Letter/comment/review without additional data |
| Isaac D. Effectiveness of Colonoscopy for Colorectal Cancer Screening in Reducing Cancer-Related Mortality: Interpreting the Results From Two Ongoing Randomized Trials. Journal of Clinical Outcomes Management 2022;29(6):513-21. doi: https://dx.doi.org/10.12788/jcom.0115 | Exclude - Letter/comment/review without additional data |
| Jacobs IJ, Parmar M, Skates SJ, Menon U. Ovarian cancer screening: UKCTOCS trial - Authors' reply. Lancet (London, England). 2016;387(10038):2603-4. | Exclude - Letter/comment/review without additional data |
| Jatoi I. Mammography screening for breast cancer-the UK Age trial. The Lancet Oncology. 2020;21(11):e506. | Exclude - Letter/comment/review without additional data |
| Kopans DB. The UK Age Trial: screening women in their forties. The Lancet Oncology. 2015;16(9):1012-3. | Exclude - Letter/comment/review without additional data |
| Kosters JP, Gotzsche PC. Regular self-examination or clinical examination for early detection of breast cancer. Cochrane database of systematic reviews (Online) 2003(2):CD003373. | Exclude - Letter/comment/review without additional data |
| Kosters JP, Gotzsche PC. Regular self-examination or clinical examination for early detection of breast cancer. Cochrane Database of Systematic Reviews 2003;2010(1) (no pagination) doi: https://dx.doi.org/10.1002/14651858.CD003373 | Exclude - Letter/comment/review without additional data |
| Kramer BS, Berg CD, Aberle DR, Prorok PC. Lung cancer screening with low-dose helical CT: results from the National Lung Screening Trial (NLST). Journal of medical screening. 2011;18(3):109-11. | Exclude - Letter/comment/review without additional data |
| Labrie F. PSA screening for prostate cancer: Why so much controversy? Asian Journal of Andrology. 2013;15(5):603-7. | Exclude - Letter/comment/review without additional data |
| Marcus PM, Bergstralh EJ, Kramer BS, Fontana R. The Mayo Lung Project lung cancer mortality findings are unlikely to be biased by a volunteer effect. European journal of epidemiology. 2011;26(6):503-4. | Exclude - Letter/comment/review without additional data |
| Martin RM, Dixon P, Turner E, Keeney E. Contribution of the Cluster randomised triAl of PSA testing for Prostate cancer (CAP) to the ongoing debate on the value of prostate cancer screening. BJU international. 2022;129(3):269-70. | Exclude - Letter/comment/review without additional data |
| Mascalchi M, Lopes Pegna A, Carrozzi L, Carozzi F, Falaschi F, Picozzi G, et al. Does UKLS strategy increase the yield of screen-detected lung cancers? A comparison with ITALUNG. Thorax. 2016;71(10):950-1. | Exclude - Letter/comment/review without additional data |
| Mascalchi M, Lopes Pegna A, Carrozzi L, Carozzi F, Falaschi F, Picozzi G, et al. Does UKLS strategy increase the yield of screen-detected lung cancers? A comparison with ITALUNG. Thorax. 2016;71(10):950-1. | Exclude - Letter/comment/review without additional data |
| Mascalchi M, Sali L. Risk of Second Lung Cancer in ITALUNG LDCT Screening. Journal of thoracic oncology : official publication of the International Association for the Study of Lung Cancer. 2018;13(6):e105-e6. | Exclude - Letter/comment/review without additional data |
| Melamed MR. Lung cancer screening results in the National Cancer Institute New York study. Cancer. 2000;89(11 Suppl):2356-62. | Exclude - Letter/comment/review without additional data |
| Miller AB, Baines CJ, Sickles EA. Canadian National Breast Screening Study. AJR American journal of roentgenology. 1990;155(5):1133-4. | Exclude - Letter/comment/review without additional data |
| Miller AB, Baines CJ, To T. The Gothenburg breast screening trial: first results on mortality, incidence, and mode of detection for women ages 39-49 years at randomization. Cancer. 1998;83(1):186-90. | Exclude - Letter/comment/review without additional data |
| Miller AB. Final results of the UK Age trial on breast cancer screening age. The Lancet Oncology. 2020;21(9):1125-6. | Exclude - Letter/comment/review without additional data |
| Miller ZA, Lee KS. Screening for hepatocellular carcinoma in high-risk populations. Clinical Imaging. 2016;40(2):311-4. | Exclude - Letter/comment/review without additional data |
| Miller ZA, Lee KS. Screening for hepatocellular carcinoma in high-risk populations. Clinical Imaging. 2016;40(2):311-4. | Exclude - Letter/comment/review without additional data |
| Mwirigi A, McDonald V, Shapiro S, Hunt BJ, Young J, Glaves R, et al. Is there benefit in extensive screening for cancer in patients with unprovoked venous thromboembolism? Thrombosis Research. 2015;136(6):1332-3. | Exclude - Letter/comment/review without additional data |
| Narod SA. Mammography screening for breast cancer-the UK Age trial. The Lancet Oncology. 2020;21(11):e508. | Exclude - Letter/comment/review without additional data |
| Nicholson S, Farndon JR. Edinburgh trial of screening for breast cancer. Lancet (London, England). 1990;335(8700):1290-1. | Exclude - Letter/comment/review without additional data |
| Otten HM, Prins MH. A number needed to screen and cost-effectiveness analysis of the SOMIT-data. Haemostasis. 2001;31 Suppl 1:40-2. | Exclude - Letter/comment/review without additional data |
| Oudkerk M, Heuvelmans MA. Screening for lung cancer by imaging: the Nelson study. JBR-BTR : organe de la Societe royale belge de radiologie (SRBR) = orgaan van de Koninklijke Belgische Vereniging voor Radiologie (KBVR). 2013;96(3):163-6. | Exclude - Letter/comment/review without additional data |
| Patz EF, Jr. Lung cancer screening, overdiagnosis bias, and reevaluation of the Mayo Lung Project. Journal of the National Cancer Institute. 2006;98(11):724-5. | Exclude - Letter/comment/review without additional data |
| Pinsky PF, Loberg M, Senore C, Wooldrage K, Atkin W, Bretthauer M, et al. Number of Adenomas Removed and Colorectal Cancers Prevented in Randomized Trials of Flexible Sigmoidoscopy Screening. Gastroenterology. 2018;155(4):1059-68.e2. | Exclude - Letter/comment/review without additional data |
| Powell K, Prasad V. Interpreting the results from the first randomised controlled trial of colonoscopy: does it save lives? BMJ Evidence-Based Medicine 2023;(no pagination) doi: https://dx.doi.org/10.1136/bmjebm-2022-112155 | Exclude - Letter/comment/review without additional data |
| Rota M, Pizzato M, La Vecchia C, Boffetta P. Efficacy of lung cancer screening appears to increase with prolonged intervention: results from the MILD trial and a meta-analysis. Annals of oncology : official journal of the European Society for Medical Oncology. 2019;30(7):1040-3. | Exclude - Letter/comment/review without additional data |
| Ruparel M, Janes SM. Lung cancer screening: what we can learn from UKLS? Thorax. 2016;71(2):103-4. | Exclude - Letter/comment/review without additional data |
| Saab S. Liver cancer screening in a high-risk population in China fails to reduce mortality. Evidence-Based Healthcare. 2004;8(4):221-3. | Exclude - Letter/comment/review without additional data |
| Sankaranarayanan R, Dinshaw K, Nene BM, Ramadas K, Esmy PO, Jayant K, et al. Cervical and oral cancer screening in India. Journal of medical screening. 2006;13 Suppl 1:S35-8. | Exclude - Letter/comment/review without additional data |
| Sankaranarayanan R, Nene BM, Dinshaw K, Rajkumar R, Shastri S, Wesley R, et al. Early detection of cervical cancer with visual inspection methods: A summary of completed and on-going studies in India. Salud Publica de Mexico. 2003;45(SUPPL. 3):S399-S407. | Exclude - Letter/comment/review without additional data |
| Sankaranarayanan R, Nene BM, Dinshaw K, Rajkumar R, Shastri S, Wesley R, et al. Early detection of cervical cancer with visual inspection methods: A summary of completed and on-going studies in India. Salud Publica de Mexico. 2003;45(SUPPL. 3):S399-S407. | Exclude - Letter/comment/review without additional data |
| Schabath MB, Aberle DR. MILD trial, strong confirmation of lung cancer screening efficacy. Nature reviews Clinical oncology. 2019;16(9):529-30. | Exclude - Letter/comment/review without additional data |
| Schroder FH, Denis LJ, Roobol M, Nelen V, Auvinen A, Tammela T, et al. The story of the European Randomized Study of Screening for Prostate Cancer. BJU international. 2003;92 Suppl 2:1-13. | Exclude - Letter/comment/review without additional data |
| Schroder FH, Habbema DF, Roobol MJ, Bangma CH. Prostate cancer in the Swedish section of ERSPC--evidence for less metastases at diagnosis but not for mortality reduction. European urology. 2007;51(3):588-90. | Exclude - Letter/comment/review without additional data |
| Schroder FH. Screening for prostate cancer (PC)--an update on recent findings of the European Randomized Study of Screening for Prostate Cancer (ERSPC). Urologic oncology. 2008;26(5):533-41. | Exclude - Letter/comment/review without additional data |
| Schroder FH. Screening for prostate cancer: current status of ERSPC and screening-related issues. Recent results in cancer research Fortschritte der Krebsforschung Progres dans les recherches sur le cancer. 2014;202:47-51. | Exclude - Letter/comment/review without additional data |
| Shapiro S. Screening: assessment of current studies. Cancer. 1994;74(1 Suppl):231-8. | Exclude - Letter/comment/review without additional data |
| Shaukat A, Kaalby L, Baatrup G, Kronborg O, Duval S, Shyne M, et al. Effects of Screening Compliance on Long-term Reductions in All-Cause and Colorectal Cancer Mortality. Clinical Gastroenterology and Hepatology. 2021;19(5):967-75.e2. | Exclude - Letter/comment/review without additional data |
| Singer RB. Fecal occult blood testing and the incidence of colorectal cancer. Journal of insurance medicine (New York, NY). 2002;34(2):89-91. | Exclude - Letter/comment/review without additional data |
| Sobue T, Nakayama T. Re: Lung cancer mortality in the Mayo Lung Project: impact of extended follow-up. Journal of the National Cancer Institute. 2001;93(4):320-3. | Exclude - Letter/comment/review without additional data |
| Strauss GM, Gleason RE, Sugarbaker DJ, Caro. Screening for lung cancer: Another look; A different view. Chest. 1997;111(3):754-68. | Exclude - Letter/comment/review without additional data |
| Strauss GM, Gleason RE, Sugarbaker DJ. Chest X-ray screening improves outcome in lung cancer. A reappraisal of randomized trials on lung cancer screening. Chest. 1995;107(6 Suppl):270S-9S. | Exclude - Letter/comment/review without additional data |
| Strauss GM, Gleason RE, Sugarbaker DJ. Screening for lung cancer re-examined. A reinterpretation of the Mayo Lung Project randomized trial on lung cancer screening. Chest. 1993;103(4 Suppl):337S-41S. | Exclude - Letter/comment/review without additional data |
| Strauss GM, Gleason RE, Sugarbaker DJ. Screening for lung cancer. Another look; a different view. Chest. 1997;111(3):754-68. | Exclude - Letter/comment/review without additional data |
| Tabar L, Chen TH-H, Hsu C-Y, Wu WY-Y, Yen AM-F, Chen SL-S, et al. Evaluation issues in the Swedish Two-County Trial of breast cancer screening: An historical review. Journal of medical screening. 2017;24(1):27-33. | Exclude - Letter/comment/review without additional data |
| Tabar L, Duffy S. Malmo mammographic screening trial. BMJ (Clinical research ed). 1989;298(6665):48-9. | Exclude - Letter/comment/review without additional data |
| Taghipour S, Banjevic D, Fernandes J, Miller AB, Montgomery N, Jardine AKS, et al. Predictors of competing mortality to invasive breast cancer incidence in the Canadian National Breast Screening study. BMC cancer. 2012;12:299. | Exclude - Letter/comment/review without additional data |
| Taneja SS. Re: Effect of a Low-Intensity PSA-Based Screening Intervention on Prostate Cancer Mortality: The CAP Randomized Clinical Trial. The Journal of urology. 2018;200(4):699. | Exclude - Letter/comment/review without additional data |
| Vastag B. Lung screening study to test popular CT scans. JAMA. 2002;288(14):1705-6. | Exclude - Letter/comment/review without additional data |
| Wieszczy P, Kaminski MF, Loberg M, Bugajski M, Bretthauer M, Kalager M. Estimation of overdiagnosis in colorectal cancer screening with sigmoidoscopy and faecal occult blood testing: comparison of simulation models. BMJ open. 2021;11(4):e042158. | Exclude - Letter/comment/review without additional data |
| Zappa M, Puliti D, Hugosson J, Schroder FH, van Leeuwen PJ, Kranse R, et al. A different method of evaluation of the ERSPC trial confirms that prostate-specific antigen testing has a significant impact on prostate cancer mortality. European urology. 2014;66(3):401-3. | Exclude - Letter/comment/review without additional data |
| **No intermediate outcomes or further mortality endpoints comparing trial arms reported (n = 67)** | |
| Alexander F, Roberts MM, Lutz W, Hepburn W. Randomisation by cluster and the problem of social class bias. Journal of epidemiology and community health. 1989;43(1):29-36. | Exclude - No relevant outcomes |
| Alexander FE. Estimation of sojourn time distributions and false negative rates in screening programmes which use two modalities. Statistics in medicine. 1989;8(6):743-55. | Exclude - No relevant outcomes |
| Andersson I, Andren L, Hildell J, Linell F, Ljungqvist U, Pettersson H. Breast cancer screening with mammography: a population-based, randomized trial with mammography as the only screening mode. Radiology. 1979;132(2):273-6. | Exclude - No relevant outcomes |
| Andersson I. Radiographic screening for breast carcinoma. I. Program and primary findings in 45-69 year old women. Acta Radiologica - Series Diagnosis. 1981;22(2):185-94. | Exclude - No relevant outcomes |
| Baines CJ, To T, Miller AB. Revised estimates of overdiagnosis from the Canadian National Breast Screening Study. Preventive medicine. 2016;90:66-71. | Exclude - No relevant outcomes |
| Bertoletti L, Robin P, Jara-Palomares L, et al. Predicting the risk of cancer after unprovoked venous thromboembolism: external validation of the RIETE score. Journal of thrombosis and haemostasis : JTH 2017;15(11):2184-87. doi: https://dx.doi.org/10.1111/jth.13842 | Exclude - No relevant outcomes |
| Burki TK. Flexible sigmoidoscopy screening for colorectal cancer. The Lancet Oncology. 2014;15(10):e425. | Exclude - No relevant outcomes |
| Ciatto S, Zappa M, Villers A, Paez A, Otto SJ, Auvinen A. Contamination by opportunistic screening in the European Randomized Study of Prostate Cancer Screening. BJU International, Supplement. 2003;92(2):97-100. | Exclude - No relevant outcomes |
| Colby TV, Tazelaar HD, Travis WD, Bergstralh EJ, Jett JR. Pathologic review of the Mayo Lung Project cancers [corrected]. Is there a case for misdiagnosis or overdiagnosis of lung carcinoma in the screened group? Cancer. 2002;95(11):2361-5. | Exclude - No relevant outcomes |
| Coyle K, Carrier M, Lazo-Langner A, et al. Cost effectiveness of the addition of a comprehensive CT scan to the abdomen and pelvis for the detection of cancer after unprovoked venous thromboembolism. Thrombosis Research 2017;151:67-71. doi: https://dx.doi.org/10.1016/j.thromres.2017.01.007 | Exclude - No relevant outcomes |
| Croswell JM, Baker SG, Marcus PM, Clapp JD, Kramer BS. Cumulative incidence of false-positive test results in lung cancer screening: a randomized trial. Annals of internal medicine. 2010;152(8):505-80. | Exclude - No relevant outcomes |
| Dinshaw K, Mishra G, Shastri S, Badwe R, Kerkar R, Ramani S, et al. Determinants of compliance in a cluster randomised controlled trial on screening of breast and cervix cancer in mumbai, India. 2. Compliance to referral and treatment. Oncology. 2007;73(3-4):154-61. | Exclude - No relevant outcomes |
| Duffy SW, Agbaje O, Tabar L, Vitak B, Bjurstam N, Bjorneld L, et al. Overdiagnosis and overtreatment of breast cancer: estimates of overdiagnosis from two trials of mammographic screening for breast cancer. Breast cancer research : BCR. 2005;7(6):258-65. | Exclude - No relevant outcomes |
| Duffy SW, Day NE, Tabar L, Chen HH, Smith TC. Markov models of breast tumor progression: some age-specific results. Journal of the National Cancer Institute Monographs. 1997(22):93-7. | Exclude - No relevant outcomes |
| Ederer F, Church TR, Mandel JS. Fecal occult blood screening in the Minnesota study: role of chance detection of lesions. Journal of the National Cancer Institute. 1997;89(19):1423-8. | Exclude - No relevant outcomes |
| Fink R, Shapiro S. Significance of increased efforts to gain participation in screening for breast cancer. American journal of preventive medicine. 1990;6(1):34-41. | Exclude - No relevant outcomes |
| Finne P, Stenman UH, Maattanen L, Makinen T, Tammela TLJ, Martikainen P, et al. The Finnish trial of prostate cancer screening: where are we now? BJU international. 2003;92 Suppl 2:22-6. | Exclude - No relevant outcomes |
| Fletcher SW, Black W, Harris R, Rimer BK, Shapiro S. Report of the International Workshop on Screening for Breast Cancer. Journal of the National Cancer Institute. 1993;85(20):1644-56. | Exclude - No relevant outcomes |
| Fontana RS, Sanderson DR, Miller WE. The Mayo Lung Project: Preliminary report of early cancer detection phase. Cancer. 1972;30(5):1373-9. | Exclude - No relevant outcomes |
| Fontana RS, Sanderson DR, Taylor WF, Woolner LB, Miller WE, Muhm JR, et al. Early lung cancer detection: results of the initial (prevalence) radiologic and cytologic screening in the Mayo Clinic study. The American review of respiratory disease. 1984;130(4):561-5. | Exclude - No relevant outcomes |
| Frisell J, von Rosen A, Wiege M, Nilsson B, Goldman S. Interval cancer and survival in a randomized breast cancer screening trial in Stockholm. Breast cancer research and treatment. 1992;24(1):11-6. | Exclude - No relevant outcomes |
| Godtman RA, Remmers S, Aus G, Nelen V, van Eycken L, Villers A, et al. Intervention-related Deaths in the European Randomized Study of Screening for Prostate Cancer. European urology open science. 2021;34:27-32. | Exclude - No relevant outcomes |
| Horeweg N, van der Aalst CM, Thunnissen E, Nackaerts K, Weenink C, Groen HJM, et al. Characteristics of lung cancers detected by computer tomography screening in the randomized NELSON trial. American journal of respiratory and critical care medicine. 2013;187(8):848-54. | Exclude - No relevant outcomes |
| Howe GR, Sherman GJ, Semenciw RM, Miller AB. Estimated benefits and risks of screening for breast cancer. Canadian Medical Association Journal. 1981;124(4):399-403. | Exclude - No relevant outcomes |
| Huynh-Le M-P, Fan CC, Karunamuni R, Walsh EI, Turner EL, Lane JA, et al. A Genetic Risk Score to Personalize Prostate Cancer Screening, Applied to Population Data. Cancer epidemiology, biomarkers & prevention : a publication of the American Association for Cancer Research, cosponsored by the American Society of Preventive Oncology. 2020;29(9):1731-8. | Exclude - No relevant outcomes |
| Jang H, Kim S, Wu D. Bayesian lead time estimation for the Johns Hopkins Lung Project data. Journal of epidemiology and global health. 2013;3(3):157-63. | Exclude - No relevant outcomes |
| Keeney E, Sanghera S, Martin RM, Gulati R, Wiklund F, Walsh EI, et al. Cost-Effectiveness Analysis of Prostate Cancer Screening in the UK: A Decision Model Analysis Based on the CAP Trial. PharmacoEconomics. 2022;40(12):1207-20. | Exclude - No relevant outcomes |
| Kim S, Jang H, Wu D, Abrams J. A Bayesian nonlinear mixed-effects disease progression model. Journal of biometrics & biostatistics. 2015;6(5). | Exclude - No relevant outcomes |
| Kim S, Wu D. Estimation of sensitivity depending on sojourn time and time spent in preclinical state. Statistical methods in medical research. 2016;25(2):728-40. | Exclude - No relevant outcomes |
| Kranse R, van Leeuwen PJ, Hakulinen T, Hugosson J, Tammela TL, Ciatto S, et al. Excess all-cause mortality in the evaluation of a screening trial to account for selective participation. Journal of medical screening. 2013;20(1):39-45. | Exclude - No relevant outcomes |
| Lane JA, Hamdy FC, Martin RM, Turner EL, Neal DE, Donovan JL. Latest results from the UK trials evaluating prostate cancer screening and treatment: the CAP and ProtecT studies. European journal of cancer (Oxford, England : 1990). 2010;46(17):3095-101. | Exclude - No relevant outcomes |
| Lindholm E, Berglund B, Haglind E, Kewenter J. Factors associated with participation in screening for colorectal cancer with faecal occult blood testing. Scandinavian journal of gastroenterology. 1995;30(2):171-6. | Exclude - No relevant outcomes |
| Loeb S, Zhu X, Schroder FH, Roobol MJ. Long-term radical prostatectomy outcomes among participants from the European Randomized Study of Screening for Prostate Cancer (ERSPC) Rotterdam. BJU international. 2012;110(11):1678-83. | Exclude - No relevant outcomes |
| Lopes Pegna A, Picozzi G, Mascalchi M, Maria Carozzi F, Carrozzi L, Comin C, et al. Design, recruitment and baseline results of the ITALUNG trial for lung cancer screening with low-dose CT. Lung cancer (Amsterdam, Netherlands). 2009;64(1):34-40. | Exclude - No relevant outcomes |
| Lujan M, Paez A, Angulo JC, Granados R, Nevado M, Torres GM, et al. Long-term prostate-specific antigen contamination in the Spanish arm of the European Randomized Study of Screening for Prostate Cancer (ERSPC). Actas urologicas espanolas. 2016;40(3):164-72. | Exclude - No relevant outcomes |
| MacKenzie TA, Loberg M, O'Malley AJ. Patient Centered Hazard Ratio Estimation Using Principal Stratification Weights: Application to the NORCCAP Randomized Trial of Colorectal Cancer Screening. Observational studies. 2016;2:29-50. | Exclude - No relevant outcomes |
| Mandel JS, Church TR, Bond JH, Ederer F, Geisser MS, Mongin SJ, et al. The effect of fecal occult-blood screening on the incidence of colorectal cancer. The New England journal of medicine. 2000;343(22):1603-7. | Exclude - No relevant outcomes |
| Marcus PM, Bergstralh EJ, Zweig MH, Harris A, Offord KP, Fontana RS. Extended lung cancer incidence follow-up in the Mayo Lung Project and overdiagnosis. Journal of the National Cancer Institute. 2006;98(11):748-56. | Exclude - No relevant outcomes |
| Marcus PM. Lung cancer screening: an update. Journal of clinical oncology : official journal of the American Society of Clinical Oncology. 2001;19(18 Suppl):83S-6S. | Exclude - No relevant outcomes |
| Miller AB, Howe GR, Wall C. The National Study of Breast Cancer Screening Protocol for a Canadian Randomized Controlled trial of screening for breast cancer in women. Clinical and investigative medicine Medecine clinique et experimentale. 1981;4(3-4):227-58. | Exclude - No relevant outcomes |
| Miller AB. Is routine mammography screening appropriate for women 40-49 years of age? American Journal of Preventive Medicine. 1991;7(1):55-62. | Exclude - No relevant outcomes |
| Miller AB. The costs and benefits of breast cancer screening. American journal of preventive medicine. 1993;9(3):175-80. | Exclude - No relevant outcomes |
| Nene B, Jayant K, Arrossi S, Shastri S, Budukh A, Hingmire S, et al. Determinants of womens participation in cervical cancer screening trial, Maharashtra, India. Bulletin of the World Health Organization. 2007;85(4):264-72. | Exclude - No relevant outcomes |
| Olsson A, Garne JP, Tengrup I, Zackrisson S, Manjer J. Body mass index and breast cancer survival in relation to the introduction of mammographic screening. European Journal of Surgical Oncology. 2009;35(12):1261-7. | Exclude - No relevant outcomes |
| Parker RD, Harber P, Kessler LG. Evaluation of screening effectiveness. Journal of Medical Systems. 1983;7(1):11-24. | Exclude - No relevant outcomes |
| Pashkevich MA, Sigal BM, Plevritis SK. Modeling the transition of lung cancer from early to advanced stage. Cancer causes & control : CCC. 2009;20(9):1559-69. | Exclude - No relevant outcomes |
| Pinsky PF, Loberg M, Senore C, Wooldrage K, Atkin W, Bretthauer M, et al. Number of Adenomas Removed and Colorectal Cancers Prevented in Randomized Trials of Flexible Sigmoidoscopy Screening. Gastroenterology. 2018;155(4):1059-68.e2. | Exclude - No relevant outcomes |
| Pinsky PF, Loberg M, Senore C, Wooldrage K, Atkin W, Bretthauer M, et al. Number of Adenomas Removed and Colorectal Cancers Prevented in Randomized Trials of Flexible Sigmoidoscopy Screening. Gastroenterology. 2018;155(4):1059-68.e2. | Exclude - No relevant outcomes |
| Pinsky PF, Miller A, Kramer BS, Church T, Reding D, Prorok P, et al. Evidence of a healthy volunteer effect in the prostate, lung, colorectal, and ovarian cancer screening trial. American journal of epidemiology. 2007;165(8):874-81. | Exclude - No relevant outcomes |
| Prosper AE, Inoue K, Brown K, Bui AAT, Aberle D, Hsu W. Association of Inclusion of More Black Individuals in Lung Cancer Screening with Reduced Mortality. JAMA Network Open. 2021:e2119629. | Exclude - No relevant outcomes |
| Scholefield JH, Robinson MHE, Mangham CM, Hardcastle JD. Screening for colorectal cancer reduces emergency admissions. European Journal of Surgical Oncology. 1998;24(1):47-50. | Exclude - No relevant outcomes |
| Schroder FH, Kranse R, Rietbergen J, Hoedemaeke R, Kirkels W. The European Randomized Study of Screening for Prostate Cancer (ERSPC): an update. Members of the ERSPC, Section Rotterdam. European urology. 1999;35(5-6):539-43. | Exclude - No relevant outcomes |
| Senore C, Bonelli L, Sciallero S, Casella C, Santarelli A, Armaroli P, et al. Assessing generalizability of the findings of sigmoidoscopy screening trials: The case of SCORE trial. Journal of the National Cancer Institute. 2015;107(1). | Exclude - No relevant outcomes |
| Shaevitch D, Taghipour S, Miller AB, Montgomery N, Harvey B. Tumor size distribution of invasive breast cancers and the sensitivity of screening methods in the Canadian National Breast Screening Study. Journal of cancer research and therapeutics. 2017;13(3):562-9. | Exclude - No relevant outcomes |
| Shapiro S, Venet W, Strax P, Venet L, Roeser R. Prospects for eliminating racial differences in breast cancer survival rates. American journal of public health. 1982;72(10):1142-5. | Exclude - No relevant outcomes |
| Shaukat A, Shyne M, Mandel JS, Snover D, Church TR. Colonoscopy With Polypectomy Reduces Long-Term Incidence of Colorectal Cancer in Both Men and Women: Extended Results From the Minnesota Colon Cancer Control Study. Gastroenterology. 2021;160(4):1397-9.e3. | Exclude - No relevant outcomes |
| Strax P, Venet L, Shapiro S. Mass screening in mammary cancer. Cancer. 1969;23(4):875-8. | Exclude - No relevant outcomes |
| Strax P. Results of mass screening for breast cancer in 50,000 examinations. Cancer. 1976;37(1):30-5. | Exclude - No relevant outcomes |
| Tabar L, Chen HH, Fagerberg G, Duffy SW, Smith TC. Recent results from the Swedish Two-County Trial: the effects of age, histologic type, and mode of detection on the efficacy of breast cancer screening. Journal of the National Cancer Institute Monographs. 1997(22):43-7. | Exclude - No relevant outcomes |
| Taghipour S, Banjevic D, Fernandes J, Miller AB, Montgomery N, Harvey BJ, et al. Incidence of invasive breast cancer in the presence of competing mortality: the Canadian National Breast Screening Study. Breast cancer research and treatment. 2012;134(2):839-51. | Exclude - No relevant outcomes |
| Turner EL, Metcalfe C, Donovan JL, Noble S, Sterne JAC, Lane JA, et al. Design and preliminary recruitment results of the Cluster randomised triAl of PSA testing for Prostate cancer (CAP). British journal of cancer. 2014;110(12):2829-36. | Exclude - No relevant outcomes |
| van Leeuwen PJ, Roobol MJ, Kranse R, Zappa M, Carlsson S, Bul M, et al. Towards an optimal interval for prostate cancer screening. European urology. 2012;61(1):171-6. | Exclude - No relevant outcomes |
| Villers A, Malavaud B, Rebillard X, Bataille V, Iborra F. ERSPC: features and preliminary results of France. BJU international. 2003;92 Suppl 2:27-9. | Exclude - No relevant outcomes |
| Walter SD, Hu J, Talala K, Tammela T, Taari K, Auvinen A. Estimating the rate of overdiagnosis with prostate cancer screening: evidence from the Finnish component of the European Randomized Study of Screening for Prostate Cancer. Cancer causes & control : CCC. 2021;32(11):1299-313. | Exclude - No relevant outcomes |
| Warwick J, Tabar L, Vitak B, Duffy SW. Time-dependent effects on survival in breast carcinoma: results of 20 years of follow-up from the Swedish Two-County Study. Cancer. 2004;100(7):1331-6. | Exclude - No relevant outcomes |
| Zackrisson S, Andersson I, Manjer J, Janzon L. Non-attendance in breast cancer screening is associated with unfavourable socio-economic circumstances and advanced carcinoma. International journal of cancer. 2004;108(5):754-60. | Exclude - No relevant outcomes |
| Zhu X, Kranse R, Bul M, Bangma CH, Schroder FH, Roobol MJ. Overestimation of prostate cancer mortality and other-cause mortality by the Kaplan-Meier method. The Canadian journal of urology. 2013;20(3):6756-60. | Exclude - No relevant outcomes |
| **No relevant cancer screening randomised controlled trial (n = 23)** | |
| Anderson TJ, Waller M, Ellis IO, Bobrow L, Moss S. Influence of annual mammography from age 40 on breast cancer pathology. Human pathology. 2004;35(10):1252-9. | Exclude - No relevant trial |
| Andersson I, Andren L, Hildell J, Linell F, Ljungqvist U, Pettersson H. Breast cancer screening with mammography: a population-based, randomized trial with mammography as the only screening mode. Radiology. 1979;132(2):273-6. | Exclude - No relevant trial |
| Andersson I, Fagerberg G, Lundgren B, Tabar L. Breast cancer screening in Sweden. The single modality approach. Der Radiologe. 1980;20(12):608-11. | Exclude - No relevant trial |
| Andersson I. Mammographic screening of an urban population in Sweden: Implications of results from a randomized trial. Practical modalities of an efficient screening for breast cancer in the European Community: proceedings of an International Symposium of the Association Against Cancer (Belgium) ICS865. 1989:95-100. | Exclude - No relevant trial |
| Ebell MH. Routine CT scans for occult malignancy not useful in patients with unprovoked VTE. American Family Physician. 2016;93(1):59-60. | Exclude - No relevant trial |
| Faivre J, Dancourt V, Lejeune C, Tazi MA, Lamour J, Gerard D, et al. Reduction in colorectal cancer mortality by fecal occult blood screening in a French controlled study. Gastroenterology. 2004;126(7):1674-80. | Exclude - No relevant trial |
| Farley SJ. Prostate cancer: PSA-based screening in Sweden dramatically reduces disease-specific mortality. Nature Reviews Urology. 2010;7(8):415. | Exclude - No relevant trial |
| Guldbrandt LM. The effect of direct referral for fast CT scan in early lung cancer detection in general practice. A clinical, cluster-randomised trial. Danish medical journal. 2015;62(3). | Exclude - No relevant trial |
| Hoff G, Botteri E, Hoie O, et al. Polyp detection rates as quality indicator in clinical versus screening colonoscopy. Endoscopy International Open 2019;7(2):E195-E202. doi: https://dx.doi.org/10.1055/a-0796-6477 | Exclude - No relevant trial |
| Ihaddadene R, Corsi DJ, Lazo-Langner A, et al. Risk factors predictive of occult cancer detection in patients with unprovoked venous thromboembolism. Blood 2016;127(16):2035-7. doi: https://dx.doi.org/10.1182/blood-2015-11-682963 | Exclude - No relevant trial |
| Infante M, Sestini S, Galeone C, Marchiano A, Lutman FR, Angeli E, et al. Lung cancer screening with low-dose spiral computed tomography: evidence from a pooled analysis of two Italian randomized trials. European journal of cancer prevention : the official journal of the European Cancer Prevention Organisation (ECP). 2017;26(4):324-9. | Exclude - No relevant trial |
| Lejeune C, Arveux P, Dancourt V, Fagnani F, Bonithon-Kopp C, Faivre J. A simulation model for evaluating the medical and economic outcomes of screening strategies for colorectal cancer. European Journal of Cancer Prevention. 2003;12(1):77-84. | Exclude - No relevant trial |
| Melamed MR. Lung cancer screening results in the National Cancer Institute New York study. Cancer. 2000;89(11 Suppl):2356-62. | Exclude - No relevant trial |
| Moss S, Waller M, Anderson TJ, Cuckle H. Randomised controlled trial of mammographic screening in women from age 40: Predicted mortality based on surrogate outcome measures. British Journal of Cancer. 2005;92(5):955-60. | Exclude - No relevant trial |
| Patz EF, Jr., Swensen SJ, Herndon JE, 2nd. Estimate of lung cancer mortality from low-dose spiral computed tomography screening trials: implications for current mass screening recommendations. Journal of clinical oncology : official journal of the American Society of Clinical Oncology. 2004;22(11):2202-6. | Exclude - No relevant trial |
| Picozzi G, Paci E, Lopes Pegna A, Bartolucci M, Roselli G, De Francisci A, et al. Screening of lung cancer with low dose spiral CT: Results of a three year pilot study and design of the randomised controlled trial "Italung-CT". Radiologia Medica. 2005;109(1-2):17-26. | Exclude - No relevant trial |
| Pinsky PF, Black A, Grubb R, Crawford ED, Andriole G, Thompson I, et al. Projecting prostate cancer mortality in the PCPT and REDUCE chemoprevention trials. Cancer. 2013;119(3):593-601. | Exclude - No relevant trial |
| Saab S. Liver cancer screening in a high-risk population in China fails to reduce mortality. Evidence-Based Healthcare. 2004;8(4):221-3. | Exclude - No relevant trial |
| Schreiber R. Adding CT to screening for occult cancer did not increase diagnosis of cancer from screening in unprovoked VTE. Annals of Internal Medicine. 2015;163(10):JC9. | Exclude - No relevant trial |
| Segnan N, Senore C, Andreoni B, Azzoni A, Bisanti L, Cardelli A, et al. Comparing Attendance and Detection Rate of Colonoscopy With Sigmoidoscopy and FIT for Colorectal Cancer Screening. Gastroenterology. 2007;132(7):2304-12. | Exclude - No relevant trial |
| Tabar L, Akerlund E, Gad A. Five-year experience with single-view mammography randomized controlled screening in Sweden. Recent results in cancer research Fortschritte der Krebsforschung Progres dans les recherches sur le cancer. 1984;90:105-13. | Exclude - No relevant trial |
| Tabar L, Akerlund E, Gad A. Five-year experience with single-view mammography randomized controlled screening in Sweden. Recent results in cancer research Fortschritte der Krebsforschung Progres dans les recherches sur le cancer. 1984;90:105-13. | Exclude - No relevant trial |
| Thulaseedharan JV, Malila N, Hakama M, Esmy PO, Cherian M, Swaminathan R, et al. Effect of screening on the risk estimates of socio demographic factors on cervical cancer - a large cohort study from rural India. Asian Pacific journal of cancer prevention : APJCP. 2013;14(1):589-94. | Exclude - No relevant trial |
| **Non-English language (n = 4)** | |
| Ebert M, Nitschmann S. Long-term results of colorectal cancer screening using sigmoidoscopy: UK flexible sigmoidoscopy screening trial (UKFSST). Internist. 2017;58(10):1111-3. | Exclude - Non-English language |
| Jegu J, Tretarre B, Grosclaude P, Rebillard X, Bataille V, Malavaud B, et al. Results and participation factors to the European Randomized study of Screening for Prostate Cancer (ERSPC) with Prostate Specific Antigen: French departments of Tarn and Herault. Progres en urologie : journal de l'Association francaise d'urologie et de la Societe francaise d'urologie. 2009;19(7):487-98. | Exclude - Non-English language |
| Kronborg O, Fenger C, Olsen J, Jorgensen OD, Sondergaard O. Randomized population study of screening for intestinal cancer with Hemoccult-II. Ugeskrift for laeger. 1997;159(33):4977-81. | Exclude - Non-English language |
| Tabar L, Gad A, Holmberg LH. The first results of a random study in Kopparberg and Ostergotland counties. Reduced mortality in breast cancer by health control with mammography. Lakartidningen. 1985;82(17):1551-4. | Exclude - Non-English language |
| **Screen-detected outcomes only (n = 57)** | |
| Andersson I, Fagerberg G, Lundgren B, Tabar L. Breast cancer screening in Sweden. The single modality approach. Der Radiologe. 1980;20(12):608-11. | Exclude - Surrogates #5-7 only |
| Andersson I, Fagerberg G, Lundgren B, Tabar L. Breast cancer screening in Sweden. The single modality approach. Der Radiologe. 1980;20(12):608-11. | Exclude - Surrogates #5-7 only |
| Auvinen A, Raitanen J, Moss S, de Koning HJ, Hugosson J, Tammela T, et al. Test sensitivity in the European prostate cancer screening trial: results from Finland, Sweden, and the Netherlands. Cancer epidemiology, biomarkers & prevention : a publication of the American Association for Cancer Research, cosponsored by the American Society of Preventive Oncology. 2009;18(7):2000-5. | Exclude - Surrogates #5-7 only |
| Bangma CH, Rietbergen JB, Schroder FH. Prostate-specific antigen as a screening test. The Netherlands experience. The Urologic clinics of North America. 1997;24(2):307-14. | Exclude - Surrogates #5-7 only |
| Bonkhoff H, Fixemer T, Hunsicker I, Remberger K. Evaluation of prostatic specific antigen and digital rectal examination as screening tests for prostate cancer. Prostate. 2000;45(1):19-35. | Exclude - Surrogates #5-7 only |
| Bretthauer M, Kaminski MF, Loberg M, et al. Population-Based Colonoscopy Screening for Colorectal Cancer: A Randomized Clinical Trial. JAMA Internal Medicine 2016;176(7):894-902. doi: https://dx.doi.org/10.1001/jamainternmed.2016.0960 | Exclude - Surrogates #5-7 only |
| Chen HH, Thurfjell E, Duffy SW, Tabar L. Evaluation by Markov chain models of a non-randomised breast cancer screening programme in women aged under 50 years in Sweden. Journal of epidemiology and community health. 1998;52(5):329-35. | Exclude - Surrogates #5-7 only |
| Chuang S-L, Chen SL-S, Yu C-P, Chang K-J, Yen AM-F, Chiu SY-H, et al. Using tumor phenotype, histological tumor distribution, and mammographic appearance to explain the survival differences between screen-detected and clinically detected breast cancers. APMIS : acta pathologica, microbiologica, et immunologica Scandinavica. 2014;122(8):699-707. | Exclude - Surrogates #5-7 only |
| Church TR, Ederer F, Mandel JS. Fecal occult blood screening in the Minnesota study: sensitivity of the screening test. Journal of the National Cancer Institute. 1997;89(19):1440-8. | Exclude - Surrogates #5-7 only |
| Ciatto S, Gervasi G, Frullini P, Zendron P, Zappa M. Specific features of the Italian section of the ERSPC. BJU international. 2003;92 Suppl 2:30-2. | Exclude - Surrogates #5-7 only |
| de Koning HJ, Auvinen A, Berenguer Sanchez A, Calais da Silva F, Ciatto S, Denis L, et al. Large-scale randomized prostate cancer screening trials: program performances in the European Randomized Screening for Prostate Cancer trial and the Prostate, Lung, Colorectal and Ovary cancer trial. International journal of cancer. 2002;97(2):237-44. | Exclude - Surrogates #5-7 only |
| Duffy SW, Agbaje O, Tabar L, Vitak B, Bjurstam N, Bjorneld L, et al. Overdiagnosis and overtreatment of breast cancer: estimates of overdiagnosis from two trials of mammographic screening for breast cancer. Breast cancer research : BCR. 2005;7(6):258-65. | Exclude - Surrogates #5-7 only |
| Duffy SW, Chen HH, Tabar L, Fagerberg G, Paci E. Sojourn time, sensitivity and positive predictive value of mammography screening for breast cancer in women aged 40-49. International journal of epidemiology. 1996;25(6):1139-45. | Exclude - Surrogates #5-7 only |
| Field JK, Duffy SW, Baldwin DR, Brain KE, Devaraj A, Eisen T, et al. The UK lung cancer screening trial: A pilot randomised controlled trial of low-dose computed tomography screening for the early detection of lung cancer. Health Technology Assessment. 2016;20(40):1-146. | Exclude - Surrogates #5-7 only |
| Field JK, Duffy SW, Baldwin DR, Whynes DK, Devaraj A, Brain KE, et al. UK Lung Cancer RCT Pilot Screening Trial: Baseline findings from the screening arm provide evidence for the potential implementation of lung cancer screening. Thorax. 2016;71(2):161-70. | Exclude - Surrogates #5-7 only |
| Gondal G, Grotmol T, Hofstad B, Bretthauer M, Eide TJ, Hoff G. The Norwegian Colorectal Cancer Prevention (NORCCAP) screening study: baseline findings and implementations for clinical work-up in age groups 50-64 years. Scandinavian journal of gastroenterology. 2003;38(6):635-42. | Exclude - Surrogates #5-7 only |
| Grubb RL, 3rd, Pinsky PF, Greenlee RT, Izmirlian G, Miller AB, Hickey TP, et al. Prostate cancer screening in the Prostate, Lung, Colorectal and Ovarian cancer screening trial: update on findings from the initial four rounds of screening in a randomized trial. BJU international. 2008;102(11):1524-30. | Exclude - Surrogates #5-7 only |
| Gunsoy NB, Garcia-Closas M, Moss SM. Modelling the overdiagnosis of breast cancer due to mammography screening in women aged 40 to 49 in the United Kingdom. Breast cancer research : BCR. 2012;14(6):R152. | Exclude - Surrogates #5-7 only |
| Hugosson J, Aus G, Bergdahl S, Fernlund P, Frosing R, Lodding P, et al. Population-based screening for prostate cancer by measuring free and total serum prostate-specific antigen in Sweden. BJU international. 2003;92 Suppl 2:39-43. | Exclude - Surrogates #5-7 only |
| Jodal HC, Loberg M, Holme O, Adami H-O, Bretthauer M, Emilsson L, et al. Mortality From Postscreening (Interval) Colorectal Cancers Is Comparable to That From Cancer in Unscreened Patients-A Randomized Sigmoidoscopy Trial. Gastroenterology. 2018;155(6):1787-94.e3. | Exclude - Surrogates #5-7 only |
| Kilpelainen TP, Pogodin-Hannolainen D, Kemppainen K, Talala K, Raitanen J, Taari K, et al. Estimate of Opportunistic Prostate Specific Antigen Testing in the Finnish Randomized Study of Screening for Prostate Cancer. The Journal of urology. 2017;198(1):50-7. | Exclude - Surrogates #5-7 only |
| Kwiatkowski M, Huber A, Moschopulos M, Lehmann K, Wernli M, Hafeli A, et al. Prostate cancer screening: results of a prospective trial in Canton Aargau, Switzerland. Swiss medical weekly. 2004;134(39-40):580-5. | Exclude - Surrogates #5-7 only |
| Labrie F, Candas B, Cusan L, Gomez JL, Diamond P, Suburu R, et al. Diagnosis of advanced or noncurable prostate cancer can be practically eliminated by prostate-specific antigen. Urology. 1996;47(2):212-7. | Exclude - Surrogates #5-7 only |
| Liu Z, Ji M-F, Huang Q-H, Fang F, Liu Q, Jia W-H, et al. Two Epstein-Barr virus-related serologic antibody tests in nasopharyngeal carcinoma screening: results from the initial phase of a cluster randomized controlled trial in Southern China. American journal of epidemiology. 2013;177(3):242-50. | Exclude - Surrogates #5-7 only |
| Lopes Pegna A, Picozzi G, Falaschi F, Carrozzi L, Falchini M, Carozzi FM, et al. Four-year results of low-dose CT screening and nodule management in the ITALUNG trial. Journal of thoracic oncology : official publication of the International Association for the Study of Lung Cancer. 2013;8(7):866-75. | Exclude - Surrogates #5-7 only |
| Lujan M, Paez A, Pascual C, Angulo J, Miravalles E, Berenguer A. Extent of prostate-specific antigen contamination in the Spanish section of the European Randomized Study of Screening for Prostate Cancer (ERSPC). European urology. 2006;50(6):1234-40. | Exclude - Surrogates #5-7 only |
| Maattanen L, Auvinen A, Stenman UH, Tammela T, Rannikko S, Aro J, et al. Three-year results of the Finnish prostate cancer screening trial. Journal of the National Cancer Institute. 2001;93(7):552-3. | Exclude - Surrogates #5-7 only |
| Malila N, Oivanen T, Malminiemi O, Hakama M. Test, episode, and programme sensitivities of screening for colorectal cancer as a public health policy in Finland: experimental design. BMJ (Clinical research ed). 2008;337:a2261. | Exclude - Surrogates #5-7 only |
| Menon U, Skates SJ, Lewis S, Rosenthal AN, Rufford B, Sibley K, et al. Prospective study using the risk of ovarian cancer algorithm to screen for ovarian cancer. Journal of clinical oncology : official journal of the American Society of Clinical Oncology. 2005;23(31):7919-26. | Exclude - Surrogates #5-7 only |
| Menon U, Talaat A, Rosenthal AN, MacDonald ND, Jeyerajah AR, Skates SJ, et al. Performance of ultrasound as a second line test to serum CA125 in ovarian cancer screening. BJOG: An International Journal of Obstetrics and Gynaecology. 2014;121(s7):35-9. | Exclude - Surrogates #5-7 only |
| Moss S, Thomas I, Evans A, Thomas B, Johns L, Trial Management G. Randomised controlled trial of mammographic screening in women from age 40: results of screening in the first 10 years. British journal of cancer. 2005;92(5):949-54. | Exclude - Surrogates #5-7 only |
| Muhm JR, Miller WE, Fontana RS, Sanderson DR, Uhlenhopp MA. Lung cancer detected during a screening program using four-month chest radiographs. Radiology. 1983;148(3):609-15. | Exclude - Surrogates #5-7 only |
| Patz EF, Jr., Pinsky P, Gatsonis C, Sicks JD, Kramer BS, Tammemagi MC, et al. Overdiagnosis in low-dose computed tomography screening for lung cancer. JAMA internal medicine. 2014;174(2):269-74. | Exclude - Surrogates #5-7 only |
| Pedersen JH, Ashraf H, Dirksen A, Bach K, Hansen H, Toennesen P, et al. The Danish randomized lung cancer CT screening trial--overall design and results of the prevalence round. Journal of thoracic oncology : official publication of the International Association for the Study of Lung Cancer. 2009;4(5):608-14. | Exclude - Surrogates #5-7 only |
| Pinsky PF, Gierada DS, Black W, Munden R, Nath H, Aberle D, et al. Performance of Lung-RADS in the National Lung Screening Trial: a retrospective assessment. Annals of internal medicine. 2015;162(7):485-91. | Exclude - Surrogates #5-7 only |
| Rasmussen M, Fenger C, Kronborg O. Diagnostic yield in a biennial Hemoccult-II screening program compared to a once-only screening with flexible sigmoidoscopy and Hemoccult-II. Scandinavian journal of gastroenterology. 2003;38(1):114-8. | Exclude - Surrogates #5-7 only |
| Robin P, Kumar S, Salaun P-Y, et al. In patients with unprovoked VTE, does the addition of FDG PET/CT to a limited occult cancer screening strategy offer good value for money? A cost-effectiveness analysis from the publicly funded health care systems. Thrombosis research 2018;171:97-102. doi: https://dx.doi.org/10.1016/j.thromres.2018.09.050 | Exclude - Surrogates #5-7 only |
| Robin P, Le Roux PY, Lacut K, et al. Performance of <sup>18</sup>F-fluorodesoxyglucose positron-emission tomography combined with low-dose computed tomography for cancer screening in patients with unprovoked venous thromboembolism. PLoS ONE 2017;12(6) (no pagination) doi: https://dx.doi.org/10.1371/journal.pone.0178849 | Exclude - Surrogates #5-7 only |
| Robin P, Le Roux P-Y, Tromeur C, et al. Risk factors of occult malignancy in patients with unprovoked venous thromboembolism. Thrombosis research 2017;159:48-51. doi: https://dx.doi.org/10.1016/j.thromres.2017.08.021 | Exclude - Surrogates #5-7 only |
| Robinson MH, Hardcastle JD, Moss SM, Amar SS, Chamberlain JO, Armitage NC, et al. The risks of screening: data from the Nottingham randomised controlled trial of faecal occult blood screening for colorectal cancer. Gut. 1999;45(4):588-92. | Exclude - Surrogates #5-7 only |
| Roemeling S, Roobol MJ, Otto SJ, Habbema DF, Gosselaar C, Lous JJ, et al. Feasibility study of adjustment for contamination and non-compliance in a prostate cancer screening trial. The Prostate. 2007;67(10):1053-60. | Exclude - Surrogates #5-7 only |
| Roobol MJ, Kirkels WJ, Schroder FH. Features and preliminary results of the Dutch centre of the ERSPC (Rotterdam, the Netherlands). BJU international. 2003;92 Suppl 2:48-54. | Exclude - Surrogates #5-7 only |
| Saarimaki L, Hugosson J, Tammela TL, Carlsson S, Talala K, Auvinen A. Impact of Prostatic-specific Antigen Threshold and Screening Interval in Prostate Cancer Screening Outcomes: Comparing the Swedish and Finnish European Randomised Study of Screening for Prostate Cancer Centres. European urology focus. 2019;5(2):186-91. | Exclude - Surrogates #5-7 only |
| Sankaranarayanan R, Rajkumar R, Arrossi S, Theresa R, Esmy PO, Mahe C, et al. Determinants of participation of women in a cervical cancer visual screening trial in rural south India. Cancer detection and prevention. 2003;27(6):457-65. | Exclude - Surrogates #5-7 only |
| Schroder FH, Bangma CH. The European Randomized Study of Screening for Prostate Cancer (ERSPC). British journal of urology. 1997;79 Suppl 1:68-71. | Exclude - Surrogates #5-7 only |
| Schroder FH, Damhuis RAM, Kirkels WJ, De Koning HJ, Kranse R, Nijs HGT, et al. European randomized study of screening for prostate cancer - The Rotterdam pilot studies. International Journal of Cancer. 1996;65(2):145-51. | Exclude - Surrogates #5-7 only |
| Schroder FH, Denis LJ, Kirkels W, de Koning HJ, Standaert B. European randomized study of screening for prostate cancer. Progress report of Antwerp and Rotterdam pilot studies. Cancer. 1995;76(1):129-34. | Exclude - Surrogates #5-7 only |
| Tabar L, Gad A. Screening for breast cancer: the Swedish trial. Radiology. 1981;138(1):219-22. | Exclude - Surrogates #5-7 only |
| Thulaseedharan JV, Malila N, Hakama M, Esmy PO, Cherian M, Swaminathan R, et al. Effect of screening on the risk estimates of socio demographic factors on cervical cancer - a large cohort study from rural India. Asian Pacific journal of cancer prevention : APJCP. 2013;14(1):589-94. | Exclude - Surrogates #5-7 only |
| van der Cruijsen-Koeter IW, van der Kwast TH, Schroder FH. Interval carcinomas in the European Randomized Study of Screening for Prostate Cancer (ERSPC)-Rotterdam. Journal of the National Cancer Institute. 2003;95(19):1462-6. | Exclude - Surrogates #5-7 only |
| van Leeuwen PJ, Connolly D, Tammela TLJ, Auvinen A, Kranse R, Roobol MJ, et al. Balancing the harms and benefits of early detection of prostate cancer. Cancer. 2010;116(20):4857-65. | Exclude - Surrogates #5-7 only |
| van Leeuwen PJ, Roobol MJ, Kranse R, Zappa M, Carlsson S, Bul M, et al. Towards an optimal interval for prostate cancer screening. European urology. 2012;61(1):171-6. | Exclude - Surrogates #5-7 only |
| Walter SD, Kubik A, Parkin DM, Reissigova J, Adamec M, Khlat M. The natural history of lung cancer estimated from the results of a randomized trial of screening. Cancer causes & control : CCC. 1992;3(2):115-23. | Exclude - Surrogates #5-7 only |
| Woolner LB, Fontana RS, Sanderson DR. Mayo lung project. Evaluation of lung cancer screening through December 1979. Mayo Clinic Proceedings. 1981;56(9):544-55. | Exclude - Surrogates #5-7 only |
| Wu GHM, Auvinen A, Maattanen L, Tammela TLJ, Stenman UH, Hakama M, et al. Number of screens for overdetection as an indicator of absolute risk of overdiagnosis in prostate cancer screening. International Journal of Cancer. 2012;131(6):1367-75. | Exclude - Surrogates #5-7 only |
| Yousaf-Khan U, van der Aalst C, de Jong PA, Heuvelmans M, Scholten E, Lammers J-W, et al. Final screening round of the NELSON lung cancer screening trial: the effect of a 2.5-year screening interval. Thorax. 2017;72(1):48-56. | Exclude - Surrogates #5-7 only |
| Zhang B, Yang B. Combined alpha fetoprotein testing and ultrasonography as a screening test for primary liver cancer. Journal of medical screening. 1999;6(2):108-10. | Exclude - Surrogates #5-7 only |
| **Already included in “Search 1” (n = 175)** | |
| Aberle DR, Abtin F, Brown K. Computed tomography screening for lung cancer: Has it finally arrived? implications of the national lung screening trial. Journal of Clinical Oncology. 2013;31(8):1002-8. | Excluded - “Search 1” IN |
| Alexander FE, Anderson TJ, Brown HK, Forrest AP, Hepburn W, Kirkpatrick AE, et al. 14 years of follow-up from the Edinburgh randomised trial of breast-cancer screening. Lancet (London, England). 1999;353(9168):1903-8. | Excluded - “Search 1” IN |
| Alexander FE, Anderson TJ, Brown HK, Forrest AP, Hepburn W, Kirkpatrick AE, et al. The Edinburgh randomised trial of breast cancer screening: results after 10 years of follow-up. British journal of cancer. 1994;70(3):542-8. | Excluded - “Search 1” IN |
| Alexander FE. The Edinburgh Randomized Trial of Breast Cancer Screening. Journal of the National Cancer Institute Monographs. 1997(22):31-5. | Excluded - “Search 1” IN |
| Andersson I, Aspegren K, Janzon L, Landberg T, Lindholm K, Linell F, et al. Mammographic screening and mortality from breast cancer: the Malmo mammographic screening trial. BMJ (Clinical research ed). 1988;297(6654):943-8. | Excluded - “Search 1” IN |
| Andersson I, Janzon L. Reduced breast cancer mortality in women under age 50: updated results from the Malmo Mammographic Screening Program. Journal of the National Cancer Institute Monographs. 1997(22):63-7. | Excluded - “Search 1” IN |
| Andriole GL, Crawford ED, Grubb RL, 3rd, Buys SS, Chia D, Church TR, et al. Mortality results from a randomized prostate-cancer screening trial. The New England journal of medicine. 2009;360(13):1310-9. | Excluded - “Search 1” IN |
| Andriole GL, Crawford ED, Grubb RL, 3rd, Buys SS, Chia D, Church TR, et al. Prostate cancer screening in the randomized Prostate, Lung, Colorectal, and Ovarian Cancer Screening Trial: mortality results after 13 years of follow-up. Journal of the National Cancer Institute. 2012;104(2):125-32. | Excluded - “Search 1” IN |
| Anttila A, Pokhrel A, Kotaniemi-Talonen L, Hakama M, Malila N, Nieminen P. Cervical cancer patterns with automation-assisted and conventional cytological screening: a randomized study. International journal of cancer. 2011;128(5):1204-12. | Excluded - “Search 1” IN |
| Arnsrud Godtman R, Holmberg E, Lilja H, Stranne J, Hugosson J. Opportunistic testing versus organized prostate-specific antigen screening: outcome after 18 years in the Goteborg randomized population-based prostate cancer screening trial. European urology. 2015;68(3):354-60. | Excluded - “Search 1” IN |
| Atkin W, Wooldrage K, Parkin DM, Kralj-Hans I, MacRae E, Shah U, et al. Long term effects of once-only flexible sigmoidoscopy screening after 17 years of follow-up: the UK Flexible Sigmoidoscopy Screening randomised controlled trial. Lancet (London, England). 2017;389(10076):1299-311. | Excluded - “Search 1” IN |
| Atkin WS, Edwards R, Kralj-Hans I, Wooldrage K, Hart AR, Northover JMA, et al. Once-only flexible sigmoidoscopy screening in prevention of colorectal cancer: a multicentre randomised controlled trial. Lancet (London, England). 2010;375(9726):1624-33. | Excluded - “Search 1” IN |
| Becker N, Motsch E, Trotter A, Heussel CP, Dienemann H, Schnabel PA, et al. Lung cancer mortality reduction by LDCT screening-Results from the randomized German LUSI trial. International journal of cancer. 2020;146(6):1503-13. | Excluded - “Search 1” IN |
| Berenguer A, Lujan M, Paez A, Santonja C, Pascual T. The Spanish contribution to the European Randomized Study of Screening for Prostate Cancer. BJU international. 2003;92 Suppl 2:33-8. | Excluded - “Search 1” IN |
| Bjurstam N, Bjorneld L, Duffy SW, Smith TC, Cahlin E, Erikson O, et al. The Gothenburg Breast Cancer Screening Trial: preliminary results on breast cancer mortality for women aged 39-49. Journal of the National Cancer Institute Monographs. 1997(22):53-5. | Excluded - “Search 1” IN |
| Bjurstam N, Bjorneld L, Duffy SW, Smith TC, Cahlin E, Eriksson O, et al. The Gothenburg breast screening trial: first results on mortality, incidence, and mode of detection for women ages 39-49 years at randomization. Cancer. 1997;80(11):2091-9. | Excluded - “Search 1” IN |
| Bjurstam N, Bjorneld L, Warwick J, Sala E, Duffy SW, Nystrom L, et al. The Gothenburg Breast Screening Trial. Cancer. 2003;97(10):2387-96. | Excluded - “Search 1” IN |
| Bjurstam NG, Bjorneld LM, Duffy SW. Updated results of the Gothenburg Trial of Mammographic Screening. Cancer. 2016;122(12):1832-5. | Excluded - “Search 1” IN |
| Bokhorst LP, Bangma CH, van Leenders GJLH, Lous JJ, Moss SM, Schroder FH, et al. Prostate-specific antigen-based prostate cancer screening: reduction of prostate cancer mortality after correction for nonattendance and contamination in the Rotterdam section of the European Randomized Study of Screening for Prostate Cancer. European urology. 2014;65(2):329-36. | Excluded - “Search 1” IN |
| Bretthauer M, Loberg M, Wieszczy P, et al. Effect of Colonoscopy Screening on Risks of Colorectal Cancer and Related Death. New England Journal of Medicine 2022;387(17):1547-56. doi: https://dx.doi.org/10.1056/NEJMoa2208375 | Excluded - “Search 1” IN |
| Buys SS, Partridge E, Black A, Johnson CC, Lamerato L, Isaacs C, et al. Effect of screening on ovarian cancer mortality: the Prostate, Lung, Colorectal and Ovarian (PLCO) Cancer Screening Randomized Controlled Trial. JAMA. 2011;305(22):2295-303. | Excluded - “Search 1” IN |
| Carrier M, Lazo-Langner A, Shivakumar S, et al. Screening for Occult Cancer in Unprovoked Venous Thromboembolism. The New England journal of medicine 2015;373(8):697-704. doi: https://dx.doi.org/10.1056/NEJMoa1506623 | Excluded - “Search 1” IN |
| Chen HH, Tabar L, Fagerberg G, Duffy SW. Effect of breast cancer screening after age 65. Journal of medical screening. 1995;2(1):10-4. | Excluded - “Search 1” IN |
| Chen JG, Parkin DM, Chen QG, Lu JH, Shen QJ, Zhang BC, et al. Screening for liver cancer: results of a randomised controlled trial in Qidong, China. Journal of medical screening. 2003;10(4):204-9. | Excluded - “Search 1” IN |
| Crawford ED, Grubb R, 3rd, Black A, Andriole GL, Jr., Chen M-H, Izmirlian G, et al. Comorbidity and mortality results from a randomized prostate cancer screening trial. Journal of clinical oncology : official journal of the American Society of Clinical Oncology. 2011;29(4):355-61. | Excluded - “Search 1” IN |
| de Koning HJ, van der Aalst CM, de Jong PA, Scholten ET, Nackaerts K, Heuvelmans MA, et al. Reduced Lung-Cancer Mortality with Volume CT Screening in a Randomized Trial. The New England journal of medicine. 2020;382(6):503-13. | Excluded - “Search 1” IN |
| Doria-Rose VP, Marcus PM, Miller AB, Bergstralh EJ, Mandel JS, Tockman MS, et al. Does the source of death information affect cancer screening efficacy results? A study of the use of mortality review versus death certificates in four randomized trials. Clinical Trials. 2010;7(1):69-77. | Excluded - “Search 1” IN |
| Doria-Rose VP, Marcus PM, Szabo E, Tockman MS, Melamed MR, Prorok PC. Randomized controlled trials of the efficacy of lung cancer screening by sputum cytology revisited: a combined mortality analysis from the Johns Hopkins Lung Project and the Memorial Sloan-Kettering Lung Study. Cancer. 2009;115(21):5007-17. | Excluded - “Search 1” IN |
| Doria-Rose VP, Marcus PM, Szabo E, Tockman MS, Melamed MR, Prorok PC. Randomized controlled trials of the efficacy of lung cancer screening by sputum cytology revisited: a combined mortality analysis from the Johns Hopkins Lung Project and the Memorial Sloan-Kettering Lung Study. Cancer. 2009;115(21):5007-17. | Excluded - “Search 1” IN |
| Doria-Rose VP, Marcus PM. Death certificates provide an adequate source of cause of death information when evaluating lung cancer mortality: an example from the Mayo Lung Project. Lung cancer (Amsterdam, Netherlands). 2009;63(2):295-300. | Excluded - “Search 1” IN |
| Doroudi M, Pinsky PF, Marcus PM. Lung Cancer Mortality in the Lung Screening Study Feasibility Trial. JNCI cancer spectrum. 2018;2(3):pky042. | Excluded - “Search 1” IN |
| Duffy S, Vulkan D, Cuckle H, Parmar D, Sheikh S, Smith R, et al. Annual mammographic screening to reduce breast cancer mortality in women from age 40 years: long-term follow-up of the UK Age RCT. Health technology assessment (Winchester, England). 2020;24(55):1-24. | Excluded - “Search 1” IN |
| Duffy SW, Tabar L, Vitak B, Day NE, Smith RA, Chen HHT, et al. The relative contributions of screen-detected in situ and invasive breast carcinomas in reducing mortality from the disease. European journal of cancer (Oxford, England : 1990). 2003;39(12):1755-60. | Excluded - “Search 1” IN |
| Duffy SW, Tabar L, Vitak B, Yen MF, Warwick J, Smith RA, et al. The Swedish Two-County Trial of mammographic screening: cluster randomisation and end point evaluation. Annals of oncology : official journal of the European Society for Medical Oncology. 2003;14(8):1196-8. | Excluded - “Search 1” IN |
| Duffy SW, Vulkan D, Cuckle H, Parmar D, Sheikh S, Smith RA, et al. Effect of mammographic screening from age 40 years on breast cancer mortality (UK Age trial): final results of a randomised, controlled trial. The Lancet Oncology. 2020;21(9):1165-72. | Excluded - “Search 1” IN |
| Field JK, Vulkan D, Davies MPA, Baldwin DR, Brain KE, Devaraj A, et al. Lung cancer mortality reduction by LDCT screening: UKLS randomised trial results and international meta-analysis. The Lancet regional health Europe. 2021;10:100179. | Excluded - “Search 1” IN |
| Fontana RS, Sanderson DR, Woolner LB, Taylor WF, Miller WE, Muhm JR, et al. Screening for lung cancer. A critique of the Mayo Lung Project. Cancer. 1991;67(4 Suppl):1155-64. | Excluded - “Search 1” IN |
| Fontana RS. The Mayo Lung Project: a perspective. Cancer. 2000;89(11 Suppl):2352-5. | Excluded - “Search 1” IN |
| Franlund M, Mansson M, Godtman RA, Aus G, Holmberg E, Kollberg KS, et al. Results from 22 years of Followup in the Goteborg Randomized Population-Based Prostate Cancer Screening Trial. Journal of Urology. 2022;208(2):292-300. | Excluded - “Search 1” IN |
| Frisell J, Eklund G, Hellstrom L, Lidbrink E, Rutqvist LE, Somell A. Randomized study of mammography screening--preliminary report on mortality in the Stockholm trial. Breast cancer research and treatment. 1991;18(1):49-56. | Excluded - “Search 1” IN |
| Frisell J, Lidbrink E, Hellstrom L, Rutqvist LE. Followup after 11 years--update of mortality results in the Stockholm mammographic screening trial. Breast cancer research and treatment. 1997;45(3):263-70. | Excluded - “Search 1” IN |
| Frisell J, Lidbrink E. The Stockholm Mammographic Screening Trial: Risks and benefits in age group 40-49 years. Journal of the National Cancer Institute Monographs. 1997(22):49-51. | Excluded - “Search 1” IN |
| Hakama M, Moss SM, Stenman U-H, Roobol MJ, Zappa M, Carlsson S, et al. Design-corrected variation by centre in mortality reduction in the ERSPC randomised prostate cancer screening trial. Journal of medical screening. 2017;24(2):98-103. | Excluded - “Search 1” IN |
| Hardcastle JD, Chamberlain JO, Robinson MH, Moss SM, Amar SS, Balfour TW, et al. Randomised controlled trial of faecal-occult-blood screening for colorectal cancer. Lancet (London, England). 1996;348(9040):1472-7. | Excluded - “Search 1” IN |
| Hoff G, Grotmol T, Skovlund E, Bretthauer M, Norwegian Colorectal Cancer Prevention Study G. Risk of colorectal cancer seven years after flexible sigmoidoscopy screening: randomised controlled trial. BMJ (Clinical research ed). 2009;338:b1846. | Excluded - “Search 1” IN |
| Holme O, Loberg M, Kalager M, Bretthauer M, Hernan MA, Aas E, et al. Effect of flexible sigmoidoscopy screening on colorectal cancer incidence and mortality: a randomized clinical trial. JAMA. 2014;312(6):606-15. | Excluded - “Search 1” IN |
| Holme O, Loberg M, Kalager M, Bretthauer M, Hernan MA, Aas E, et al. Long-Term Effectiveness of Sigmoidoscopy Screening on Colorectal Cancer Incidence and Mortality in Women and Men: A Randomized Trial. Annals of internal medicine. 2018;168(11):775-82. | Excluded - “Search 1” IN |
| Hugosson J, Carlsson S, Aus G, Bergdahl S, Khatami A, Lodding P, et al. Mortality results from the Goteborg randomised population-based prostate-cancer screening trial. The Lancet Oncology. 2010;11(8):725-32. | Excluded - “Search 1” IN |
| Hugosson J, Godtman RA, Carlsson SV, Aus G, Grenabo Bergdahl A, Lodding P, et al. Eighteen-year follow-up of the Goteborg Randomized Population-based Prostate Cancer Screening Trial: effect of sociodemographic variables on participation, prostate cancer incidence and mortality. Scandinavian journal of urology. 2018;52(1):27-37. | Excluded - “Search 1” IN |
| Hugosson J, Roobol MJ, Mansson M, Tammela TLJ, Zappa M, Nelen V, et al. A 16-yr Follow-up of the European Randomized study of Screening for Prostate Cancer. European urology. 2019;76(1):43-51. | Excluded - “Search 1” IN |
| Infante M, Cavuto S, Lutman FR, Brambilla G, Chiesa G, Ceresoli G, et al. A randomized study of lung cancer screening with spiral computed tomography: three-year results from the DANTE trial. American journal of respiratory and critical care medicine. 2009;180(5):445-53. | Excluded - “Search 1” IN |
| Infante M, Cavuto S, Lutman FR, Passera E, Chiarenza M, Chiesa G, et al. Long-Term Follow-up Results of the DANTE Trial, a Randomized Study of Lung Cancer Screening with Spiral Computed Tomography. American journal of respiratory and critical care medicine. 2015;191(10):1166-75. | Excluded - “Search 1” IN |
| Infante M, Sestini S, Galeone C, Marchiano A, Lutman FR, Angeli E, et al. Lung cancer screening with low-dose spiral computed tomography: evidence from a pooled analysis of two Italian randomized trials. European journal of cancer prevention : the official journal of the European Cancer Prevention Organisation (ECP). 2017;26(4):324-9. | Excluded - “Search 1” IN |
| Infante M, Sestini S, Galeone C, Marchiano A, Lutman FR, Angeli E, et al. Lung cancer screening with low-dose spiral computed tomography: evidence from a pooled analysis of two Italian randomized trials. European journal of cancer prevention : the official journal of the European Cancer Prevention Organisation (ECP). 2017;26(4):324-9. | Excluded - “Search 1” IN |
| Jacobs IJ, Menon U, Ryan A, Gentry-Maharaj A, Burnell M, Kalsi JK, et al. Ovarian cancer screening and mortality in the UK Collaborative Trial of Ovarian Cancer Screening (UKCTOCS): a randomised controlled trial. Lancet (London, England). 2016;387(10022):945-56. | Excluded - “Search 1” IN |
| Jacobs IJ, Skates SJ, MacDonald N, Menon U, Rosenthal AN, Davies AP, et al. Screening for ovarian cancer: a pilot randomised controlled trial. Lancet (London, England). 1999;353(9160):1207-10. | Excluded - “Search 1” IN |
| Ji MF, Sheng W, Cheng WM, Ng MH, Wu BH, Yu X, et al. Incidence and mortality of nasopharyngeal carcinoma: interim analysis of a cluster randomized controlled screening trial (PRO-NPC-001) in southern China. Annals of oncology : official journal of the European Society for Medical Oncology. 2019;30(10):1630-7. | Excluded - “Search 1” IN |
| Jorgensen OD, Kronborg O, Fenger C. A randomised study of screening for colorectal cancer using faecal occult blood testing: results after 13 years and seven biennial screening rounds. Gut. 2002;50(1):29-32. | Excluded - “Search 1” IN |
| Kilpelainen TP, Talala K, Raitanen J, Taari K, Kujala P, Tammela TLJ, et al. Prostate Cancer and Socioeconomic Status in the Finnish Randomized Study of Screening for Prostate Cancer. American journal of epidemiology. 2016;184(10):720-31. | Excluded - “Search 1” IN |
| Kilpelainen TP, Tammela TL, Malila N, Hakama M, Santti H, Maattanen L, et al. Prostate cancer mortality in the Finnish randomized screening trial. Journal of the National Cancer Institute. 2013;105(10):719-25. | Excluded - “Search 1” IN |
| Kilpelainen TP, Tammela TLJ, Malila N, Hakama M, Santti H, Maattanen L, et al. The Finnish prostate cancer screening trial: analyses on the screening failures. International journal of cancer. 2015;136(10):2437-43. | Excluded - “Search 1” IN |
| Kovalchik SA, Tammemagi M, Berg CD, Caporaso NE, Riley TL, Korch M, et al. Targeting of low-dose CT screening according to the risk of lung-cancer death. The New England journal of medicine. 2013;369(3):245-54. | Excluded - “Search 1” IN |
| Kronborg O, Fenger C, Olsen J, Bech K, Sondergaard O. Repeated screening for colorectal cancer with fecal occult blood test. A prospective randomized study at Funen, Denmark. Scandinavian journal of gastroenterology. 1989;24(5):599-606. | Excluded - “Search 1” IN |
| Kronborg O, Fenger C, Olsen J, Jorgensen OD, Sondergaard O. Randomised study of screening for colorectal cancer with faecal-occult-blood test. Lancet (London, England). 1996;348(9040):1467-71. | Excluded - “Search 1” IN |
| Kronborg O, Fenger C, Worm J, Pedersen SA, Hem J, Bertelsen K, et al. Causes of death during the first 5 years of a randomized trial of mass screening for colorectal cancer with fecal occult blood test. Scandinavian journal of gastroenterology. 1992;27(1):47-52. | Excluded - “Search 1” IN |
| Kronborg O, Jorgensen OD, Fenger C, Rasmussen M. Randomized study of biennial screening with a faecal occult blood test: results after nine screening rounds. Scandinavian journal of gastroenterology. 2004;39(9):846-51. | Excluded - “Search 1” IN |
| Kubik A, Haerting J. Survival and mortality in a randomized study of lung cancer detection. Neoplasma. 1990;37(4):467-75. | Excluded - “Search 1” IN |
| Kubik A, Parkin DM, Khlat M, Erban J, Polak J, Adamec M. Lack of benefit from semi-annual screening for cancer of the lung: follow-up report of a randomized controlled trial on a population of high-risk males in Czechoslovakia. International journal of cancer. 1990;45(1):26-33. | Excluded - “Search 1” IN |
| Kubik AK, Parkin DM, Zatloukal P. Czech Study on Lung Cancer Screening: post-trial follow-up of lung cancer deaths up to year 15 since enrollment. Cancer. 2000;89(11 Suppl):2363-8. | Excluded - “Search 1” IN |
| Labrie F, Candas B, Cusan L, Gomez JL, Belanger A, Brousseau G, et al. Screening decreases prostate cancer mortality: 11-year follow-up of the 1988 Quebec prospective randomized controlled trial. The Prostate. 2004;59(3):311-8. | Excluded - “Search 1” IN |
| Labrie F, Candas B, Dupont A, Cusan L, Gomez JL, Suburu RE, et al. Screening decreases prostate cancer death: first analysis of the 1988 Quebec prospective randomized controlled trial. The Prostate. 1999;38(2):83-91. | Excluded - “Search 1” IN |
| Larsson LG, Andersson I, Bjurstam N, Fagerberg G, Frisell J, Tabar L, et al. Updated overview of the Swedish Randomized Trials on Breast Cancer Screening with Mammography: age group 40-49 at randomization. Journal of the National Cancer Institute Monographs. 1997(22):57-61. | Excluded - “Search 1” IN |
| Larsson LG, Andersson I, Bjurstam N, Fagerberg G, Frisell J, Tabar L, et al. Updated overview of the Swedish Randomized Trials on Breast Cancer Screening with Mammography: age group 40-49 at randomization. Journal of the National Cancer Institute Monographs. 1997(22):57-61. | Excluded - “Search 1” IN |
| Larsson LG, Andersson I, Bjurstam N, Fagerberg G, Frisell J, Tabar L, et al. Updated overview of the Swedish Randomized Trials on Breast Cancer Screening with Mammography: age group 40-49 at randomization. Journal of the National Cancer Institute Monographs. 1997(22):57-61. | Excluded - “Search 1” IN |
| Larsson LG, Andersson I, Bjurstam N, Fagerberg G, Frisell J, Tabar L, et al. Updated overview of the Swedish Randomized Trials on Breast Cancer Screening with Mammography: age group 40-49 at randomization. Journal of the National Cancer Institute Monographs. 1997(22):57-61. | Excluded - “Search 1” IN |
| Lidbrink E, Frisell J, Brandberg Y, Rosendahl I, Rutqvist LE. Nonattendance in the Stockholm mammography screening trial: relative mortality and reasons for nonattendance. Breast cancer research and treatment. 1995;35(3):267-75. | Excluded - “Search 1” IN |
| Lindberg A, Talala K, Kujala P, Stenman U-H, Taari K, Kilpelainen TP, et al. Bias-corrected estimates of effects of PSA screening decisions on the risk of prostate cancer diagnosis and death: Analysis of the Finnish randomized study of screening for prostate cancer. International journal of cancer. 2019;145(3):632-8. | Excluded - “Search 1” IN |
| Lindholm E, Brevinge H, Haglind E. Survival benefit in a randomized clinical trial of faecal occult blood screening for colorectal cancer. The British journal of surgery. 2008;95(8):1029-36. | Excluded - “Search 1” IN |
| Lujan Galan M, Paez Borda A, Llanes Gonzalez L, Romero Cajigal I, Berenguer Sanchez A. Results of the spanish section of the European Randomized Study of Screening for Prostate Cancer (ERSPC). Update after 21 years of follow-up. Resultados de la rama espanola del Estudio Randomizado Europeo de Screening del Cancer de Prostata (ERSPC) Actualizacion tras 21 anos de seguimiento. 2020;44(6):430-6. | Excluded - “Search 1” IN |
| Lujan M, Paez A, Angulo JC, Andres G, Gimbernat H, Redondo C, et al. Update of the results of the Spanish branch of the European Randomized Study on Screening for Prostate Cancer (ERSPC). Actas urologicas espanolas. 2015;39(7):405-13. | Excluded - “Search 1” IN |
| Lujan M, Paez A, Angulo JC, Granados R, Nevado M, Torres GM, et al. Prostate cancer incidence and mortality in the Spanish section of the European Randomized Study of Screening for Prostate Cancer (ERSPC). Prostate cancer and prostatic diseases. 2014;17(2):187-91. | Excluded - “Search 1” IN |
| Lujan M, Paez A, Berenguer A, Rodriguez JA. Mortality due to prostate cancer in the Spanish arm of the European Randomized Study of Screening for Prostate Cancer (ERSPC). Results after a 15-year follow-up. Actas Urologicas Espanolas. 2012;36(7):403-9. | Excluded - “Search 1” IN |
| Mandel JS, Bond JH, Church TR, Snover DC, Bradley GM, Schuman LM, et al. Reducing mortality from colorectal cancer by screening for fecal occult blood. Minnesota Colon Cancer Control Study. The New England journal of medicine. 1993;328(19):1365-71. | Excluded - “Search 1” IN |
| Mandel JS, Church TR, Ederer F, Bond JH. Colorectal cancer mortality: Effectiveness of biennial screening for fecal occult blood. Journal of the National Cancer Institute. 1999;91(5):434-7. | Excluded - “Search 1” IN |
| Martin RM, Donovan JL, Turner EL, Metcalfe C, Young GJ, Walsh EI, et al. Effect of a Low-Intensity PSA-Based Screening Intervention on Prostate Cancer Mortality: The CAP Randomized Clinical Trial. JAMA. 2018;319(9):883-95. | Excluded - “Search 1” IN |
| Melamed MR, Flehinger BJ, Zaman MB, Heelan RT, Perchick WA, Martini N. Screening for early lung cancer. Results of the Memorial Sloan-Kettering study in New York. Chest. 1984;86(1):44-53. | Excluded - “Search 1” IN |
| Melamed MR, Flehinger BJ, Zaman MB. Impact of early detection on the clinical course of lung cancer. The Surgical clinics of North America. 1987;67(5):909-24. | Excluded - “Search 1” IN |
| Menon U, Gentry-Maharaj A, Burnell M, Singh N, Ryan A, Karpinskyj C, et al. Ovarian cancer population screening and mortality after long-term follow-up in the UK Collaborative Trial of Ovarian Cancer Screening (UKCTOCS): a randomised controlled trial. Lancet (London, England). 2021;397(10290):2182-93. | Excluded - “Search 1” IN |
| Miller AB, Baines CJ, To T, Wall C. Canadian National Breast Screening Study: 1. Breast cancer detection and death rates among women aged 40 to 49 years. CMAJ : Canadian Medical Association journal = journal de l'Association medicale canadienne. 1992;147(10):1459-76. | Excluded - “Search 1” IN |
| Miller AB, Baines CJ, To T, Wall C. Canadian National Breast Screening Study: 2. Breast cancer detection and death rates among women aged 50 to 59 years. CMAJ : Canadian Medical Association journal = journal de l'Association medicale canadienne. 1992;147(10):1477-88. | Excluded - “Search 1” IN |
| Miller AB, To T, Baines CJ, Wall C. Canadian National Breast Screening Study-2: 13-year results of a randomized trial in women aged 50-59 years. Journal of the National Cancer Institute. 2000;92(18):1490-9. | Excluded - “Search 1” IN |
| Miller AB, To T, Baines CJ, Wall C. The Canadian National Breast Screening Study: update on breast cancer mortality. Journal of the National Cancer Institute Monographs. 1997(22):37-41. | Excluded - “Search 1” IN |
| Miller AB, To T, Baines CJ, Wall C. The Canadian National Breast Screening Study-1: breast cancer mortality after 11 to 16 years of follow-up. A randomized screening trial of mammography in women age 40 to 49 years. Annals of internal medicine. 2002;137(5 Part 1):305-12. | Excluded - “Search 1” IN |
| Miller AB, Wall C, Baines CJ, Sun P, To T, Narod SA. Twenty five year follow-up for breast cancer incidence and mortality of the Canadian National Breast Screening Study: randomised screening trial. BMJ (Clinical research ed). 2014;348:g366. | Excluded - “Search 1” IN |
| Miller EA, Pinsky PF, Black A, Andriole GL, Pierre-Victor D. Secondary prostate cancer screening outcomes by race in the Prostate, Lung, Colorectal, and Ovarian (PLCO) Screening Trial. The Prostate. 2018;78(11):830-8. | Excluded - “Search 1” IN |
| Miller EA, Pinsky PF, Schoen RE, Prorok PC, Church TR. Effect of flexible sigmoidoscopy screening on colorectal cancer incidence and mortality: long-term follow-up of the randomised US PLCO cancer screening trial. The lancet Gastroenterology & hepatology. 2019;4(2):101-10. | Excluded - “Search 1” IN |
| Mittra I, Mishra GA, Dikshit RP, Gupta S, Kulkarni VY, Shaikh HKA, et al. Effect of screening by clinical breast examination on breast cancer incidence and mortality after 20 years: prospective, cluster randomised controlled trial in Mumbai. BMJ (Clinical research ed). 2021;372:n256. | Excluded - “Search 1” IN |
| Moss SM, Cuckle H, Evans A, Johns L, Waller M, Bobrow L, et al. Effect of mammographic screening from age 40 years on breast cancer mortality at 10 years' follow-up: a randomised controlled trial. Lancet (London, England). 2006;368(9552):2053-60. | Excluded - “Search 1” IN |
| Moss SM, Wale C, Smith R, Evans A, Cuckle H, Duffy SW. Effect of mammographic screening from age 40 years on breast cancer mortality in the UK Age trial at 17 years' follow-up: a randomised controlled trial. The Lancet Oncology. 2015;16(9):1123-32. | Excluded - “Search 1” IN |
| Narod SA, Wall C, Baines C, Miller AB, Sun P. Impact of screening mammography on mortality from breast cancer before age 60 in women 40 to 49 years of age. Current Oncology. 2014;21(5):217-21. | Excluded - “Search 1” IN |
| National Lung Screening Trial Research T, Aberle DR, Adams AM, Berg CD, Black WC, Clapp JD, et al. Reduced lung-cancer mortality with low-dose computed tomographic screening. The New England journal of medicine. 2011;365(5):395-409. | Excluded - “Search 1” IN |
| Neupane S, Nevalainen J, Raitanen J, Talala K, Kujala P, Taari K, et al. Prognostic Index for Predicting Prostate Cancer Survival in a Randomized Screening Trial: Development and Validation. Cancers. 2021;13(3). | Excluded - “Search 1” IN |
| Neupane S, Steyerberg E, Raitanen J, Talala K, Pylvalainen J, Taari K, et al. Prognostic factors of prostate cancer mortality in a Finnish randomized screening trial. International journal of urology : official journal of the Japanese Urological Association. 2018;25(3):270-6. | Excluded - “Search 1” IN |
| Nystrom L, Andersson I, Bjurstam N, Frisell J, Nordenskjold B, Rutqvist LE. Long-term effects of mammography screening: updated overview of the Swedish randomised trials. Lancet (London, England). 2002;359(9310):909-19. | Excluded - “Search 1” IN |
| Nystrom L, Andersson I, Bjurstam N, Frisell J, Nordenskjold B, Rutqvist LE. Long-term effects of mammography screening: Updated overview of the Swedish randomised trials. Lancet. 2002;359(9310):909-19. | Excluded - “Search 1” IN |
| Nystrom L, Andersson I, Bjurstam N, Frisell J, Nordenskjold B, Rutqvist LE. Long-term effects of mammography screening: Updated overview of the Swedish randomised trials. Lancet. 2002;359(9310):909-19. | Excluded - “Search 1” IN |
| Nystrom L, Larsson LG, Wall S, Rutqvist LE, Andersson I, Bjurstam N, et al. An overview of the Swedish randomised mammography trials: total mortality pattern and the representivity of the study cohorts. Journal of medical screening. 1996;3(2):85-7. | Excluded - “Search 1” IN |
| Nystrom L, Rutqvist LE, Wall S, Lindgren A, Lindqvist M, Ryden S, et al. Breast cancer screening with mammography: overview of Swedish randomised trials. Lancet (London, England). 1993;341(8851):973-8. | Excluded - “Search 1” IN |
| Nystrom L, Rutqvist LE, Wall S, Lindgren A, Lindqvist M, Ryden S, et al. Breast cancer screening with mammography: overview of Swedish randomised trials. Lancet (London, England). 1993;341(8851):973-8. | Excluded - “Search 1” IN |
| Nystrom L, Rutqvist LE, Wall S, Lindgren A, Lindqvist M, Ryden S, et al. Breast cancer screening with mammography: overview of Swedish randomised trials. Lancet (London, England). 1993;341(8851):973-8. | Excluded - “Search 1” IN |
| Nystrom L, Rutqvist LE, Wall S, Lindgren A, Lindqvist M, Ryden S, et al. Breast cancer screening with mammography: overview of Swedish randomised trials. Lancet (London, England). 1993;341(8851):973-8. | Excluded - “Search 1” IN |
| Oken MM, Hocking WG, Kvale PA, Andriole GL, Buys SS, Church TR, et al. Screening by chest radiograph and lung cancer mortality: the Prostate, Lung, Colorectal, and Ovarian (PLCO) randomized trial. JAMA. 2011;306(17):1865-73. | Excluded - “Search 1” IN |
| Paci E, Puliti D, Lopes Pegna A, Carrozzi L, Picozzi G, Falaschi F, et al. Mortality, survival and incidence rates in the ITALUNG randomised lung cancer screening trial. Thorax. 2017;72(9):825-31. | Excluded - “Search 1” IN |
| Pastorino U, Rossi M, Rosato V, Marchiano A, Sverzellati N, Morosi C, et al. Annual or biennial CT screening versus observation in heavy smokers: 5-year results of the MILD trial. European journal of cancer prevention : the official journal of the European Cancer Prevention Organisation (ECP). 2012;21(3):308-15. | Excluded - “Search 1” IN |
| Pastorino U, Silva M, Sestini S, Sabia F, Boeri M, Cantarutti A, et al. Prolonged lung cancer screening reduced 10-year mortality in the MILD trial: new confirmation of lung cancer screening efficacy. Annals of oncology : official journal of the European Society for Medical Oncology. 2019;30(7):1162-9. | Excluded - “Search 1” IN |
| Pastorino U, Sverzellati N, Sestini S, Silva M, Sabia F, Boeri M, et al. Ten-year results of the Multicentric Italian Lung Detection trial demonstrate the safety and efficacy of biennial lung cancer screening. European journal of cancer (Oxford, England : 1990). 2019;118:142-8. | Excluded - “Search 1” IN |
| Piccioli A, Lensing AWA, Prins MH, Falanga A, Scannapieco GL, Ieran M, et al. Extensive screening for occult malignant disease in idiopathic venous thromboembolism: a prospective randomized clinical trial. Journal of thrombosis and haemostasis : JTH. 2004;2(6):884-9. | Excluded - “Search 1” IN |
| Pinsky PF, Church TR, Izmirlian G, Kramer BS. The National Lung Screening Trial: results stratified by demographics, smoking history, and lung cancer histology. Cancer. 2013;119(22):3976-83. | Excluded - “Search 1” IN |
| Pinsky PF, Miller E, Prorok P, Grubb R, Crawford ED, Andriole G. Extended follow-up for prostate cancer incidence and mortality among participants in the Prostate, Lung, Colorectal and Ovarian randomized cancer screening trial. BJU international. 2019;123(5):854-60. | Excluded - “Search 1” IN |
| Pinsky PF, Miller EA, Zhu CS, Prorok PC. Overall mortality in men and women in the randomized Prostate, Lung, Colorectal, and Ovarian Cancer Screening Trial. Journal of medical screening. 2019;26(3):127-34. | Excluded - “Search 1” IN |
| Pinsky PF, Prorok PC, Yu K, Kramer BS, Black A, Gohagan JK, et al. Extended mortality results for prostate cancer screening in the PLCO trial with median follow-up of 15 years. Cancer. 2017;123(4):592-9. | Excluded - “Search 1” IN |
| Pinsky PF, Yu K, Kramer BS, Black A, Buys SS, Partridge E, et al. Extended mortality results for ovarian cancer screening in the PLCO trial with median 15years follow-up. Gynecologic oncology. 2016;143(2):270-5. | Excluded - “Search 1” IN |
| Pitkaniemi J, Seppa K, Hakama M, Malminiemi O, Palva T, Vuoristo MS, et al. Effectiveness of screening for colorectal cancer with a faecal occult-blood test, in Finland. BMJ open gastroenterology. 2015;2(1):e000034. | Excluded - “Search 1” IN |
| Prandoni P, Bernardi E, Valle FD, et al. Extensive Computed Tomography versus Limited Screening for Detection of Occult Cancer in Unprovoked Venous Thromboembolism: A Multicenter, Controlled, Randomized Clinical Trial. Seminars in thrombosis and hemostasis 2016;42(8):884-90. | Excluded - “Search 1” IN |
| Prorok PC, Wright P, Riley TR, Kramer BS, Berg CD, Gohagan JK. Overall and Multiphasic Findings of the Prostate, Lung, Colorectal and Ovarian (PLCO) Randomized Cancer Screening Trial. Reviews on recent clinical trials. 2018;13(4):257-73. | Excluded - “Search 1” IN |
| Puliti D, Mascalchi M, Carozzi FM, Carrozzi L, Falaschi F, Paci E, et al. Decreased cardiovascular mortality in the ITALUNG lung cancer screening trial: Analysis of underlying factors. Lung cancer (Amsterdam, Netherlands). 2019;138:72-8. | Excluded - “Search 1” IN |
| Ramadas K, Basu P, Mathew BS, Muwonge R, Venugopal M, Prakasan AM, et al. Effectiveness of triennial screening with clinical breast examination: 14-years follow-up outcomes of randomized clinical trial in Trivandrum, India. Cancer. 2023;129(2):272-82. | Excluded - “Search 1” IN |
| Ramadas K, Sankaranarayanan R, Jacob BJ, Thomas G, Somanathan T, Mahe C, et al. Interim results from a cluster randomized controlled oral cancer screening trial in Kerala, India. Oral oncology. 2003;39(6):580-8. | Excluded - “Search 1” IN |
| Roberts MM, Alexander FE, Anderson TJ, Chetty U, Donnan PT, Forrest P, et al. Edinburgh trial of screening for breast cancer: mortality at seven years. Lancet (London, England). 1990;335(8684):241-6. | Excluded - “Search 1” IN |
| Robin P, Le Roux P-Y, Planquette B, et al. Limited screening with versus without (18)F-fluorodeoxyglucose PET/CT for occult malignancy in unprovoked venous thromboembolism: an open-label randomised controlled trial. The Lancet Oncology 2016;17(2):193-99. doi: https://dx.doi.org/10.1016/S1470-2045(15)00480-5 | Excluded - “Search 1” IN |
| Roobol MJ, Kerkhof M, Schroder FH, Cuzick J, Sasieni P, Hakama M, et al. Prostate cancer mortality reduction by prostate-specific antigen-based screening adjusted for nonattendance and contamination in the European Randomised Study of Screening for Prostate Cancer (ERSPC). European urology. 2009;56(4):584-91. | Excluded - “Search 1” IN |
| Roobol MJ, Kranse R, Bangma CH, van Leenders AGJLH, Blijenberg BG, van Schaik RHN, et al. Screening for prostate cancer: results of the Rotterdam section of the European randomized study of screening for prostate cancer. European urology. 2013;64(4):530-9. | Excluded - “Search 1” IN |
| Saghir Z, Dirksen A, Ashraf H, Bach KS, Brodersen J, Clementsen PF, et al. CT screening for lung cancer brings forward early disease. The randomised Danish Lung Cancer Screening Trial: status after five annual screening rounds with low-dose CT. Thorax. 2012;67(4):296-301. | Excluded - “Search 1” IN |
| Sandblom G, Varenhorst E, Lofman O, Rosell J, Carlsson P. Clinical consequences of screening for prostate cancer: 15 years follow-up of a randomised controlled trial in Sweden. European urology. 2004;46(6):717-24. | Excluded - “Search 1” IN |
| Sandblom G, Varenhorst E, Rosell J, Lofman O, Carlsson P. Randomised prostate cancer screening trial: 20 year follow-up. BMJ (Clinical research ed). 2011;342:d1539. | Excluded - “Search 1” IN |
| Sankaranarayanan R, Esmy PO, Rajkumar R, Muwonge R, Swaminathan R, Shanthakumari S, et al. Effect of visual screening on cervical cancer incidence and mortality in Tamil Nadu, India: a cluster-randomised trial. Lancet (London, England). 2007;370(9585):398-406. | Excluded - “Search 1” IN |
| Sankaranarayanan R, Nene BM, Shastri SS, Jayant K, Muwonge R, Budukh AM, et al. HPV screening for cervical cancer in rural India. The New England journal of medicine. 2009;360(14):1385-94. | Excluded - “Search 1” IN |
| Sankaranarayanan R, Ramadas K, Thara S, Muwonge R, Thomas G, Anju G, et al. Long term effect of visual screening on oral cancer incidence and mortality in a randomized trial in Kerala, India. Oral oncology. 2013;49(4):314-21. | Excluded - “Search 1” IN |
| Sankaranarayanan R, Ramadas K, Thomas G, Muwonge R, Thara S, Mathew B, et al. Effect of screening on oral cancer mortality in Kerala, India: a cluster-randomised controlled trial. Lancet (London, England). 2005;365(9475):1927-33. | Excluded - “Search 1” IN |
| Schoen RE, Pinsky PF, Weissfeld JL, Yokochi LA, Church T, Laiyemo AO, et al. Colorectal-cancer incidence and mortality with screening flexible sigmoidoscopy. The New England journal of medicine. 2012;366(25):2345-57. | Excluded - “Search 1” IN |
| Scholefield JH, Moss S, Sufi F, Mangham CM, Hardcastle JD. Effect of faecal occult blood screening on mortality from colorectal cancer: results from a randomised controlled trial. Gut. 2002;50(6):840-4. | Excluded - “Search 1” IN |
| Scholefield JH, Moss SM, Mangham CM, Whynes DK, Hardcastle JD. Nottingham trial of faecal occult blood testing for colorectal cancer: A 20-year follow-up. Gut. 2012;61(7):1036-40. | Excluded - “Search 1” IN |
| Schroder FH, Hugosson J, Roobol MJ, Tammela TLJ, Ciatto S, Nelen V, et al. Prostate-cancer mortality at 11 years of follow-up. The New England journal of medicine. 2012;366(11):981-90. | Excluded - “Search 1” IN |
| Schroder FH, Hugosson J, Roobol MJ, Tammela TLJ, Ciatto S, Nelen V, et al. Screening and prostate-cancer mortality in a randomized European study. The New England journal of medicine. 2009;360(13):1320-8. | Excluded - “Search 1” IN |
| Schroder FH, Hugosson J, Roobol MJ, Tammela TLJ, Zappa M, Nelen V, et al. Screening and prostate cancer mortality: results of the European Randomised Study of Screening for Prostate Cancer (ERSPC) at 13 years of follow-up. Lancet (London, England). 2014;384(9959):2027-35. | Excluded - “Search 1” IN |
| Segnan N, Armaroli P, Bonelli L, Risio M, Sciallero S, Zappa M, et al. Once-only sigmoidoscopy in colorectal cancer screening: Follow-up findings of the italian randomized controlled trial - SCORE. Journal of the National Cancer Institute. 2011;103(17):1310-22. | Excluded - “Search 1” IN |
| Senore C, Riggi E, Armaroli P, Bonelli L, Sciallero S, Zappa M, et al. Long-Term Follow-up of the Italian Flexible Sigmoidoscopy Screening Trial. Annals of internal medicine. 2022;175(1):36-45. | Excluded - “Search 1” IN |
| Shapiro S, Venet W, Strax P, Venet L, Roeser R. Selection, follow-up, and analysis in the Health Insurance Plan Study: a randomized trial with breast cancer screening. National Cancer Institute monograph. 1985;67:65-74. | Excluded - “Search 1” IN |
| Shapiro S, Venet W, Strax P, Venet L, Roeser R. Ten- to fourteen-year effect of screening on breast cancer mortality. Journal of the National Cancer Institute. 1982;69(2):349-55. | Excluded - “Search 1” IN |
| Shapiro S. Evidence on screening for breast cancer from a randomized trial. Cancer. 1977;39(6 ,sup):2772-82. | Excluded - “Search 1” IN |
| Shapiro S. Periodic screening for breast cancer: the HIP Randomized Controlled Trial. Health Insurance Plan. Journal of the National Cancer Institute Monographs. 1997(22):27-30. | Excluded - “Search 1” IN |
| Shapiro S. The status of breast cancer screening: a quarter of a century of research. World journal of surgery. 1989;13(1):9-18. | Excluded - “Search 1” IN |
| Shastri SS, Mittra I, Mishra GA, Gupta S, Dikshit R, Singh S, et al. Effect of VIA screening by primary health workers: randomized controlled study in Mumbai, India. Journal of the National Cancer Institute. 2014;106(3):dju009. | Excluded - “Search 1” IN |
| Shaukat A, Mongin SJ, Geisser MS, Lederle FA, Bond JH, Mandel JS, et al. Long-term mortality after screening for colorectal cancer. The New England journal of medicine. 2013;369(12):1106-14. | Excluded - “Search 1” IN |
| Strauss GM. The Mayo Lung Cohort: a regression analysis focusing on lung cancer incidence and mortality. Journal of clinical oncology : official journal of the American Society of Clinical Oncology. 2002;20(8):1973-83. | Excluded - “Search 1” IN |
| Tabar L, Chen TH-H, Yen AM-F, Chen SL-S, Fann JC-Y, Chiu SY-H, et al. Effect of Mammography Screening on Mortality by Histological Grade. Cancer epidemiology, biomarkers & prevention : a publication of the American Association for Cancer Research, cosponsored by the American Society of Preventive Oncology. 2018;27(2):154-7. | Excluded - “Search 1” IN |
| Tabar L, Duffy SW, Yen MF, Warwick J, Vitak B, Chen HH, et al. All-cause mortality among breast cancer patients in a screening trial: support for breast cancer mortality as an end point. Journal of medical screening. 2002;9(4):159-62. | Excluded - “Search 1” IN |
| Tabar L, Fagerberg CJ, Gad A, Baldetorp L, Holmberg LH, Grontoft O, et al. Reduction in mortality from breast cancer after mass screening with mammography. Randomised trial from the Breast Cancer Screening Working Group of the Swedish National Board of Health and Welfare. Lancet (London, England). 1985;1(8433):829-32. | Excluded - “Search 1” IN |
| Tabar L, Fagerberg G, Chen HH, Duffy SW, Gad A. Screening for breast cancer in women aged under 50: mode of detection, incidence, fatality, and histology. Journal of medical screening. 1995;2(2):94-8. | Excluded - “Search 1” IN |
| Tabar L, Fagerberg G, Chen HH, Duffy SW, Smart CR, Gad A, et al. Efficacy of breast cancer screening by age. New results from the Swedish Two-County Trial. Cancer. 1995;75(10):2507-17. | Excluded - “Search 1” IN |
| Tabar L, Fagerberg G, Duffy SW, Day NE, Gad A, Grontoft O. Update of the Swedish two-county program of mammographic screening for breast cancer. Radiologic clinics of North America. 1992;30(1):187-210. | Excluded - “Search 1” IN |
| Tabar L, Fagerberg G, Duffy SW, Day NE. The Swedish two county trial of mammographic screening for breast cancer: recent results and calculation of benefit. Journal of epidemiology and community health. 1989;43(2):107-14. | Excluded - “Search 1” IN |
| Tabar L, Vitak B, Chen HH, Duffy SW, Yen MF, Chiang CF, et al. The Swedish Two-County Trial twenty years later. Updated mortality results and new insights from long-term follow-up. Radiologic clinics of North America. 2000;38(4):625-51. | Excluded - “Search 1” IN |
| Tabar L, Vitak B, Chen TH-H, Yen AM-F, Cohen A, Tot T, et al. Swedish two-county trial: impact of mammographic screening on breast cancer mortality during 3 decades. Radiology. 2011;260(3):658-63. | Excluded - “Search 1” IN |
| Tabar L, Vitak B, Yen MFA, Chen HHT, Smith RA, Duffy SW. Number needed to screen: lives saved over 20 years of follow-up in mammographic screening. Journal of medical screening. 2004;11(3):126-9. | Excluded - “Search 1” IN |
| Taylor WF, Fontana RS, Uhlenhopp MA, Davis CS. Some results of screening for early lung cancer. Cancer. 1981;47(5 Suppl):1114-20. | Excluded - “Search 1” IN |
| Thiis-Evensen E, Hoff GS, Sauar J, Langmark F, Majak BM, Vatn MH. Population-based surveillance by colonoscopy: effect on the incidence of colorectal cancer. Telemark Polyp Study I. Scandinavian journal of gastroenterology. 1999;34(4):414-20. | Excluded - “Search 1” IN |
| Thiis-Evensen E, Kalager M, Bretthauer M, Hoff G. Long-term effectiveness of endoscopic screening on incidence and mortality of colorectal cancer: A randomized trial. United European Gastroenterology Journal. 2013;1(3):162-8. | Excluded - “Search 1” IN |
| Thomas DB, Gao DL, Ray RM, et al. Randomized trial of breast self-examination in Shanghai: final results. Journal of the National Cancer Institute 2002;94(19):1445-57. | Excluded - “Search 1” IN |
| van Leeuwen PJ, Kranse R, Hakulinen T, Hugosson J, Tammela TL, Ciatto S, et al. Impacts of a population-based prostate cancer screening programme on excess total mortality rates in men with prostate cancer: a randomized controlled trial. Journal of medical screening. 2013;20(1):33-8. | Excluded - “Search 1” IN |
| van Leeuwen PJ, Kranse R, Hakulinen T, Roobol MJ, de Koning HJ, Bangma CH, et al. Disease-specific mortality may underestimate the total effect of prostate cancer screening. Journal of medical screening. 2010;17(4):204-10. | Excluded - “Search 1” IN |
| Villers A, Bessaoud F, Tretarre B, Grosclaude P, Malavaud B, Rebillard X, et al. Contamination in control group led to no effect of PSA-based screening on prostate cancer mortality at 9 years follow-up: Results of the French section of European Randomized Study of Screening for Prostate Cancer (ERSPC). Progres en urologie : journal de l'Association francaise d'urologie et de la Societe francaise d'urologie. 2020;30(5):252-60. | Excluded - “Search 1” IN |
| Wille MMW, Dirksen A, Ashraf H, Saghir Z, Bach KS, Brodersen J, et al. Results of the Randomized Danish Lung Cancer Screening Trial with Focus on High-Risk Profiling. American journal of respiratory and critical care medicine. 2016;193(5):542-51. | Excluded - “Search 1” IN |
| Zhang B-H, Yang B-H, Tang Z-Y. Randomized controlled trial of screening for hepatocellular carcinoma. Journal of cancer research and clinical oncology. 2004;130(7):417-22. | Excluded - “Search 1” IN |
| Zheng S, Chen K, Liu X, Ma X, Yu H, Chen K, et al. Cluster randomization trial of sequence mass screening for colorectal cancer. Diseases of the colon and rectum. 2003;46(1):51-8. | Excluded - “Search 1” IN |

Reasons for exclusion. **Conference abstract**: only an abstract was reported. **Duplicate**: the same abstract was included twice for full text assessment i.e., it was identified from different databases and not picked up as a duplicate at the de-duplication stage. **Letter/comment/review without additional data**: these are letters or reviews that reported sufficient information on methods, but did not report any additional outcomes data that we have not already identified from other reports. **No intermediate outcomes or further mortality endpoints comparing trial arms reported**: no relevant comparative surrogate outcomes specified in our protocol were reported. **No relevant cancer screening randomised controlled trial**: this report was not on a trial that could be included in the study. **Non-English language**: the report was not published in English. **Screen-detected outcomes only**: these reports only included outcomes from the intervention arm e.g., proportion of screen-detected cancers. **Already included in ‘Search 1’**: reports identified in ‘Search 2’ that were already included after ‘Search 1’.

## Table S10. “Search 2” flow of records through the review, by trial.

| **Cancer type** | **Trial name/acronym** | **“Search 2” database searches** | | | | **Other sources** | **# Included in review*** | **# Included in data extraction*** | **# Included in present analyses*** |
| --- | --- | --- | --- | --- | --- | --- | --- | --- | --- |
|  |  | **# Screened** | **# Full texts assessed** | **# Included*** | **# Excluded, by reason** | **# Included*** |  |  |  |
| Bowel | Burgundy | 43 | 2 | 0 | No RCT: 2 | NA | NA | NA | NA |
| Bowel | Finnish | 20 | 5 | 3 | Already IN “Search 1”: 1  No relevant outcomes: 1 | 1 (“Search 1”) | 4 | 4 | 4 |
| Bowel | Funen | 82 | 10 | 3 | Already IN “Search 1”: 5  Non-English language: 1  No relevant outcomes: 1 | 5 (“Search 1”) | 8 | 8 | 8 |
| Bowel | Gothenburg | 45 | 5 | 3 | Already IN “Search 1”: 1  No relevant outcomes: 1 | 1 (“Search 1”) | 4 | 4 | 4 |
| Bowel | Jiashan | 56 | 1 | 0 | Already IN “Search 1”: 1 | 1 (“Search 1”) | 1 | 0 (no comparative surrogate data) | 0 (no comparative surrogate data) |
| Bowel | Minnesota | 42 | 10 | 0 | Already IN “Search 1”: 4  Letter/comment/review without additional data: 2  No relevant outcomes: 4 | 4 (“Search 1”) | 4 | 3 | 3 |
| Bowel | NORCCAP | 88 | 13 | 1 | Already IN “Search 1”: 3  Conference abstract: 4  Letter/comment/review without additional data: 2  No relevant outcomes: 3 | 3 (“Search 1”) | 4 | 3 | 3 |
| Bowel | NordICC | 126 | 5 | 0 | Already IN “Search 1”: 1  Letter/comment/review without additional data: 2  No relevant trial: 1  No relevant outcomes: 1 | 1 (“Search 1”) | 1 | 1 | 1 |
| Bowel | Nottingham | 44 | 10 | 4 | Already IN “Search 1”: 3  Letter/comment/review without additional data: 1  No relevant outcomes: 2 | 3 (“Search 1”) | 7 | 6 | 6 |
| Bowel | SCORE | 127 | 7 | 1 | Already IN “Search 1”: 2  Letter/comment/review without additional data: 1  No relevant outcomes: 2  No relevant trial: 1 | 2 (“Search 1”) | 3 | 3 | 3 |
| Bowel | Telemark | 64 | 9 | 2 | Already IN “Search 1”: 2  Conference abstract: 2  Letter/comment/review without additional data: 2  No relevant outcomes: 1 | 2 (“Search 1”) | 4 | 4 | 4 |
| Bowel | UKFSST | 166 | 8 | 2 | Already IN “Search 1”: 2  Letter/comment/review without additional data: 3  Non-English language: 1 | 2 (“Search 1”) | 4 | 3 | 3 |
| Breast | CNBSS | 113 | 18 | 1 | Already IN “Search 1”: 7  Letter/comment/review without additional data: 3  No relevant outcomes: 7 | 7 (“Search 1”) | 8 | 4 (CNBSS-1)  2 (CNBSS-2)  2 (CNBSS-1 and -2) | 4 (CNBSS-1)  2 (CNBSS-2)  2 (CNBSS-1 and -2) |
| Breast | Edinburgh | 43 | 12 | 2 | Already IN “Search 1”: 4  Letter/comment/review without additional data: 2  No relevant outcomes: 2  No relevant trial: 2 | 4 (“Search 1”)  1 (Reference list) | 7 | 5 | 5 |
| Breast | Gothenburg | 161 | 14 | 1 | Already IN “Search 1”: 7  Letter/comment/review without additional data: 1  No relevant outcomes: 1  No relevant trial: 4 | 9 (“Search 1”) | 10 | 6 | 6 |
| Breast | HIP NY | 38 | 23 | 9 | Already IN “Search 1”: 5  Duplicate: 3  Letter/comment/review without additional data: 1  No relevant outcomes: 5 | 8 (“Search 1”) | 17 | 13 | 13 |
| Breast | Malmo | 164 | 19 | 7 | Already IN “Search 1”: 5  Letter/comment/review without additional data: 1  No relevant outcomes: 5  No relevant trial: 1 | 7 (“Search 1”) | 14 | 9 | 9 |
| Breast | Russia/WHO | 13 | 5 | 2 | Letter/comment/review without additional data: 3 | 1 (“Search 1”) | 3 | 3 | 3 |
| Breast | Shanghai | 32 | 5 | 1 | Already IN “Search 1”: 1  Letter/comment/review without additional data: 3 | 1 (“Search 1”) | 2 | 2 | 2 |
| Breast | Stockholm | 29 | 10 | 2 | Already IN “Search 1”: 7  No relevant outcomes: 1 | 9 (“Search 1”) | 11 | 9 | 9 |
| Breast | Trivandrum | 49 | 2 | 1 | Already IN “Search 1”: 1 | 1 (“Search 1”) | 2 | 2 | 2 |
| Breast | Two-County | 83 | 42 | 12 | Already IN “Search 1”: 16  Conference abstract: 2  Duplicate: 1  Letter/comment/review without additional data: 1  No relevant outcomes: 9  Non-English language: 1 | 18 (“Search 1”) | 30 | 16 | 16 |
| Breast | UK Age | 177 | 17 | 2 | Already IN “Search 1”: 4  Letter/comment/review without additional data: 9  No relevant outcomes: 2 | 4 (“Search 1”) | 6 | 5 | 5 |
| Cervical | Finnish | 27 | 3 | 2 | Already IN “Search 1”: 1 | 1 (“Search 1”) | 3 | 1 | 1 |
| Cervical | Osmanabad | 78 | 3 | 1 | Already IN “Search 1”: 1  No relevant outcomes: 1 | 1 (“Search 1”) | 2 | 2 | 2 |
| Cervical | Tamil Nadu | 66 | 5 | 1 | Already IN “Search 1”: 1  Letter/comment/review without additional data: 1  No relevant outcomes: 2 | 1 (“Search 1”) | 2 | 2 | 2 |
| Liver | Qidong | 33 | 3 | 0 | Already IN “Search 1”: 1  Letter/comment/review without additional data: 2 | 1 (“Search 1”) | 1 | 1 | 1 |
| Liver | Shanghai | 36 | 5 | 1 | Already IN “Search 1”: 1  Letter/comment/review without additional data: 1  No relevant trial: 1  No relevant outcomes: 1 | 1 (“Search 1”) | 2 | 1 | 1 |
| Lung | Czech Study | 10 | 6 | 1 | Already IN “Search 1”: 3  Letter/comment/review without additional data: 1  No relevant outcomes: 1 | 3 (“Search 1”) | 4 | 4 | 4 |
| Lung | DANTE | 41 | 4 | 1 | Already IN “Search 1”: 3 | 3 (“Search 1”) | 4 | 3 | 3 |
| Lung | DLCST | 62 | 6 | 1 | Already IN “Search 1”: 2  Letter/comment/review without additional data: 1  No relevant trial: 1  No relevant outcomes: 1 | 2 (“Search 1”) | 3 | 2 | 2 |
| Lung | ITALUNG | 62 | 10 | 2 | Already IN “Search 1”: 2  Letter/comment/review without additional data: 2  No relevant outcomes: 2  No relevant trial: 2 | 2 (“Search 1”) | 4 | 3 | 3 |
| Lung | Johns Hopkins | 15 | 9 | 2 | Already IN “Search 1”: 1  Letter/comment/review without additional data: 1  No relevant outcomes: 4  No relevant trial: 1 | 1 (“Search 1”) | 3 | 3 | 3 |
| Lung | LSS | 75 | 6 | 2 | Already IN “Search 1”: 1  Letter/comment/review without additional data: 1  No relevant outcomes: 2 | 1 (“Search 1”) | 3 | 3 | 3 |
| Lung | LUSI | 104 | 3 | 2 | Already IN “Search 1”: 1 | 1 (“Search 1”) | 3 | 3 | 3 |
| Lung | Mayo Lung Project | 56 | 27 | 7 | Already IN “Search 1”: 5  Letter/comment/review without additional data: 7  No relevant outcomes: 7  No relevant trial: 1 | 6 (“Search 1”)  1 (Reference list) | 14 | 8 | 8 |
| Lung | Memorial Sloan Kettering | 12 | 6 | 2 | Already IN “Search 1”: 3  Letter/comment/review without additional data: 1 | 3 (“Search 1”) | 5 | 4 | 4 |
| Lung | MILD | 60 | 7 | 1 | Already IN “Search 1”: 4  Letter/comment/review without additional data: 2 | 4 (“Search 1”) | 5 | 5 | 5 |
| Lung | NELSON | 131 | 4 | 0 | Already IN “Search 1”: 1  Letter/comment/review without additional data: 1  No relevant outcomes: 2 | 1 (“Search 1”) | 1 | 1 | 1 |
| Lung | NLST | 114 | 12 | 4 | Already IN “Search 1”: 4  Letter/comment/review without additional data: 1  No relevant outcomes: 3 | 6 (“Search 1”) | 10 | 4 | 4 |
| Lung | UKLS | 38 | 6 | 0 | Already IN “Search 1”: 1  Letter/comment/review without additional data: 3  No relevant outcomes: 2 | 1 (“Search 1”) | 1 | 1 | 1 |
| Nasopharyngeal | China NPC | 17 | 3 | 1 | Already IN “Search 1”: 1  No relevant outcomes: 1 | 1 (“Search 1”) | 2 | 1 | 1 |
| Oral | Trivandrum | 17 | 7 | 3 | Already IN “Search 1”: 3  Letter/comment/review without additional data: 1 | 3 (“Search 1”) | 6 | 4 | 4 |
| Ovarian | UKCTOCS + Pilot | 110 | 7 | 1 | Already IN “Search 1”: 3  Letter/comment/review without additional data: 1  No relevant outcomes: 2 | 3 (“Search 1”) | 4 | 1 (Pilot)  2 (UKCTOCS) | 1 (Pilot)  2 (UKCTOCS) |
| Prostate | CAP | 22 | 7 | 0 | Already IN “Search 1”: 1  Letter/comment/review without additional data: 2  No relevant outcomes: 4 | 1 (“Search 1”) | 1 | 1 | 1 |
| Prostate | ERSPC + Pilot | 564 | 91 | 27 | Already IN “Search 1”: 26  Letter/comment/review without additional data: 7  No relevant outcomes: 30  Non-English language: 1 | 27 (“Search 1”) | 54 | 23 (ERSPC)  1 (ERSPC Pilot) | 23 (ERSPC)  1 (ERSPC Pilot) |
| Prostate | Norrkoping | 84 | 3 | 0 | Already IN “Search 1”: 2  No relevant trial: 1 | 2 (“Search 1”) | 2 | 2 | 2 |
| Prostate | Quebec | 49 | 8 | 1 | Already IN “Search 1”: 2  Letter/comment/review without additional data: 3  No relevant outcomes: 2 | 2 (“Search 1”) | 3 | 0 (no comparative surrogate data) | 0 (no comparative surrogate data) |
| Breast, cervical | Mumbai Combined | 115 | 8 | 1 | Already IN “Search 1”: 2  Letter/comment/review without additional data: 3  No relevant outcomes: 1  No relevant trial: 1 | 2 (“Search 1”) | 3 | 1 (Breast)  1 (Cervical) | 1 (Breast)  1 (Cervical) |
| Prostate, lung, bowel, ovarian | PLCO | 361 | 24 | 5 | Already IN “Search 1”: 13  Letter/comment/review without additional data: 2  No relevant outcomes: 3  No relevant trial: 1 | 13 (“Search 1”) | 18 | 4 (Bowel)**  3 (Lung)**  5 (Ovarian)**  9 (Prostate)** | 4 (Bowel)**  3 (Lung)**  5 (Ovarian)**  9 (Prostate)** |
| Multiple*** | D’Aquapendente | 4 | 2 | 0 | Already IN “Search 1”: 1  No relevant trial: 1 | 1 (“Search 1”) | 1 | 1 | 1 |
| Multiple*** | MVTEP | 9 | 7 | 1 | Already IN “Search 1”: 1  Duplicate: 1  No relevant outcomes: 4 | 1 (“Search 1”) | 2 | 1 | 1 |
| Multiple*** | SOME | 7 | 2 | 0 | Already IN “Search 1”: 1  No relevant outcomes: 1 | 1 (“Search 1”) | 1 | 1 | 0 (no relevant surrogate data) |
| Multiple*** | SOMIT | 65 | 9 | 0 | Already IN “Search 1”: 1  Conference abstract: 2  Letter/comment/review without additional data: 4  No relevant trial: 2 | 1 (“Search 1”) | 1 | 1 | 1 |

**Abbreviations.** IN, Included; NA, Not applicable; NPC, nasopharyngeal cancer; RCT, randomised controlled trial; WHO, World Health Organization.

**Trial name abbreviations.** CAP, Cluster Randomized Trial of PSA Testing for Prostate Cancer; CNBSS, Canadian National Breast Screening Study; DANTE, Detection and Screening of Early Lung Cancer by Novel Imaging Technology and Molecular Essays Trial; DLCST, Danish Lung Cancer Screening Trial; ERSPC, European Randomized Study of Screening for Prostate Cancer; HIP NY, Health Insurance Plan of Greater New York; ITALUNG, Italian Lung Cancer Screening Trial; LSS, Lung Screening Study; LUSI, German Lung Cancer Screening Intervention Trial; MILD, Multicentric Italian Lung Detection; MVTEP, Standard Diagnostic Procedures With or Without Fludeoxyglucose F 18 Positron Emission Tomography in Finding Cancer in Patients With a Blood Clot in a Vein; NELSON, Nederlands–Leuvens Longkanker Screenings Onderzoek; NLST, National Lung Screening Trial; NORCCAP, Norwegian colorectal cancer prevention; NordICC, Nordic-European Initiative on Colorectal Cancer; PLCO, Prostate, Lung, Colorectal and Ovarian Cancer Screening Trial; SCORE, Screening for COlon Rectum trial; SOMIT, Subsequent diagnosis Of Malignancy in patients presenting with Idiopathic venous Thromboembolism; UKCTOS, UK Collaborative Trial of Ovarian Cancer Screening; UKFSST, UK Flexible Sigmoidoscopy Screening Trial; UKLS, UK Lung Cancer Screening Trial.

* Several papers are included for >1 trial (e.g., overviews or pooled analyses).

** Several papers are included for >1 cancer type.

*** Different target cancers screened for in each trial.

## Table S11. Publications included in data extraction and available outcomes (57 trials).

| **Cancer type** | **Trial name/acronym** | **Publication**  **(Author name, year)** | **Available mortality outcomes** | | **Available intermediate outcomes** | |
| --- | --- | --- | --- | --- | --- | --- |
|  |  |  | **All-cause** | **Target cancer** | **Late-stage cancer incidence** | **Proportion late-stage cancers** |
| Bowel | Finnish | Chiu 2017^1^ | **-** | **-** | **✓** | **✓** |
|  |  | Koskenvuo 2019^2^ | **-** | **-** | **✓** | **✓** |
|  |  | Paimela 2010^3^ | **-** | **-** | **✓** | **✓** |
|  |  | Pitkaniemi 2015^4^ | **✓** | **✓** | **-** | **-** |
| Bowel | Funen | Bech 1991^5^ | **-** | **-** | **-** | **-** |
|  |  | Jorgensen 2002^6^ | **✓** | **✓** | **-** | **-** |
|  |  | Kronborg 1987^7^ | **✓** | **✓** | **✓** | **✓** |
|  |  | Kronborg 1989^8^ | **✓** | **✓** | **✓** | **✓** |
|  |  | Kronborg 1992^9^ | **✓** | **✓** | **-** | **-** |
|  |  | Kronborg 1996^10^ | **✓** | **✓** | **✓** | **✓** |
|  |  | Kronborg 2004^11^ | **✓** | **✓** | **-** | **-** |
|  |  | Shaukat 2021^12^ | **✓** | **✓** | **-** | **-** |
| Bowel | Gothenburg | Kewenter 1988^13^ | **-** | **-** | **✓** | **✓** |
|  |  | Kewenter 1994a^14^ | **-** | **-** | **✓** | **✓** |
|  |  | Kewenter 1994b^15^ | **-** | **-** | **✓** | **✓** |
|  |  | Lindholm 2008^16^ | **✓** | **✓** | **✓** | **✓** |
| Bowel | Minnesota | Mandel 1993^17^ | **✓** | **✓** | **✓** | **✓** |
|  |  | Mandel 1999^18^ | **✓** | **✓** | **✓** | **-** |
|  |  | Shaukat 2013^19^ | **✓** | **✓** | **-** | **-** |
| Bowel | NORCCAP | Hoff 2009^20^ | **✓** | **✓** | **✓** | **✓** |
|  |  | Holme 2014^21^ | **✓** | **✓** | **✓** | **✓** |
|  |  | Holme 2018^22^ | **✓** | **✓** | **-** | **-** |
| Bowel | NordICC | Bretthauer 2022^23^ | **✓** | **✓** | **✓** | **✓** |
| Bowel | Nottingham | Hardcastle 1989^24^ | **-** | **-** | **✓** | **✓** |
|  |  | Hardcastle 1996^25^ | **✓** | **✓** | **✓** | **✓** |
|  |  | Mapp 1999^26^ | **-** | **-** | **✓** | **✓** |
|  |  | Scholefield 2002^27^ | **✓** | **✓** | - | **-** |
|  |  | Scholefield 2012^28^ | **✓** | **✓** | **✓** | **✓** |
|  |  | Whynes 2010^29^ | **✓** | **✓** | - | **-** |
| Bowel | PLCO (bowel) | Miller 2019^30^ | - |  | **✓** | **✓** |
|  |  | Pinsky 2019^31^ | **✓** | **✓** | - | - |
|  |  | Prorok 2018^32^ | **✓** | **✓** | - | - |
|  |  | Schoen 2012^33^ | - | **✓** | **✓** | **✓** |
| Bowel | SCORE | Juul 2022^34^ | - | **✓** | - | - |
|  |  | Segnan 2011^35^ | **✓** | **✓** | **✓** | **✓** |
|  |  | Senore 2022^36^ | **✓** | **✓** | - | - |
| Bowel | Telemark | Hoff 1996^37^ | **✓** | **✓** | **✓** | **✓** |
|  |  | Hoff 2001^38^ | **✓** | - | - | - |
|  |  | Thiis-Evensen 1999^39^ | **✓** | **✓** | **✓** | **✓** |
|  |  | Thiis-Evensen 2013^40^ | **✓** | **✓** | **✓** | - |
| Bowel | UKFSST | Atkin 2010^41^ | **✓** | **✓** | **✓** | - |
|  |  | Atkin 2017^42^ | **✓** | **✓** | **✓** | - |
|  |  | Cross 2022^43^ | - | **✓** | **✓** | - |
| Breast | CNBSS | Miller 1992a^44^ | **✓** | **✓** | **✓** | **✓** |
|  |  | Miller 1992b^45^ | **✓** | **✓** | **✓** | **✓** |
|  |  | Miller 1997^46^ | **✓** | **✓** | - | - |
|  |  | Miller 2000^47^ | **✓** | **✓** | **✓** | **✓** |
|  |  | Miller 2002^48^ | **✓** | **✓** | **✓** | **✓** |
|  |  | Miller 2014^49^ | **✓** | **✓** | **✓** | **✓** |
|  |  | Narod 2014^50^ | - | **✓** | - | - |
|  |  | Shen 2005^51^ | - | - | **✓** | **✓** |
| Breast | Edinburgh | Alexander 1994^52^ | **✓** | **✓** | **✓** | **✓** |
|  |  | Alexander 1999^53^ | - | **✓** | - | - |
|  |  | Anderson 1991^54^ | - | - | **✓** | **✓** |
|  |  | Anderson 2000^55^ | - | - | - | **✓** |
|  |  | Roberts 1990^56^ | **✓** | **✓** | **✓** | **✓** |
| Breast | Gothenburg | Bjurstam 1997^57^ | **✓** | **✓** | **✓** | **✓** |
|  |  | Bjurstam 2003^58^ | **✓** | **✓** | **✓** | **✓** |
|  |  | Bjurstam 2016^59^ | - | **✓** | **✓** | **✓** |
|  |  | Larsson 1997^60^ | - | **✓** | - | - |
|  |  | Nystrom 1993^61^ | - | **✓** | - | - |
|  |  | Nystrom 2002^62^ | **✓** | **✓** | - | - |
| Breast | HIP NY | Aron 1986^63^ | **✓** | **✓** | - | - |
|  |  | Habbema 1986^64^ | - | **✓** | - | - |
|  |  | Shapiro 1966^65^ | - | - | **✓** | **✓** |
|  |  | Shapiro 1973^66^ | - | **✓** | - | - |
|  |  | Shapiro 1974^67^ | - | **✓** | - | - |
|  |  | Shapiro 1977^68^ | **✓** | **✓** | **✓** | **✓** |
|  |  | Shapiro 1978^69^ | - | **✓** | - | - |
|  |  | Shapiro 1982^70^ | - | **✓** | - | - |
|  |  | Shapiro 1985^71^ | - | **✓** | - | - |
|  |  | Shapiro 1989^72^ | - | **✓** | - | - |
|  |  | Strax 1967^73^ | - | - | **✓** | **✓** |
|  |  | Strax 1971^74^ | - | **✓** | - | - |
|  |  | Strax 1984^75^ | - | **✓** | - | - |
| Breast | Malmo | Andersson 1985^76^ | - | - | **✓** | **✓** |
|  |  | Andersson 1987^77^ | - | - | **✓** | **✓** |
|  |  | Andersson 1988^78^ | **✓** | **✓** | **✓** | **✓** |
|  |  | Autier 2009^79^ | - | **✓** | **✓** | - |
|  |  | Larsson 1997^60^ | - | **✓** | - | - |
|  |  | Nystrom 1993^61^ | - | **✓** | - | - |
|  |  | Nystrom 2002^62^ | **✓** | **✓** | - | - |
|  |  | Nystrom 2017^80^ | - | **✓** | - | - |
|  |  | Zackrisson 2006^81^ | **✓** | **✓** | - | - |
| Breast | Mumbai | Mittra 2021^82^ | **✓** | **✓** | **✓** | **✓** |
| Breast | Russia/WHO | Semiglazov 1987^83^ | - | **✓** | **✓** | **✓** |
|  |  | Semiglazov 1992^84^ | - | - | **✓** | **✓** |
|  |  | Semiglazov 1999^85^ | - | **✓** | **✓** | **✓** |
| Breast | Shanghai | Thomas 1997^86^ | **✓** | **✓** | **-** | - |
|  |  | Thomas 2002^87^ | **✓** | **✓** | **✓** | **✓** |
| Breast | Stockholm | Frisell 1986^88^ | - | - | **✓** | **✓** |
|  |  | Frisell 1989^89^ | - | - | **✓** | **✓** |
|  |  | Frisell 1991^90^ | - | **✓** | **✓** | **✓** |
|  |  | Frisell 1997a^91^ | - | - | - | - |
|  |  | Frisell 1997b^92^ | - | **✓** | **✓** | **✓** |
|  |  | Larsson 1997^60^ | - | **✓** | - | - |
|  |  | Nystrom 1993^61^ | - | **✓** | - | - |
|  |  | Nystrom 2002^62^ | **✓** | **✓** | - | - |
|  |  | Nystrom 2017^80^ | - | **✓** | - | - |
| Breast | Trivandrum | Ramadas 2023^93^ | **✓** | **✓** | **✓** | **✓** |
|  |  | Sankaranarayanan 2011^94^ | - | - | **✓** | **✓** |
| Breast | Two-County | Chen 1995^95^ | - | **✓** | **✓** | **✓** |
|  |  | Duffy 1991^96^ | - | - | **✓** | **✓** |
|  |  | Fagerberg 1985^97^ | - | - | **✓** | **✓** |
|  |  | Larsson 1997^60^ | - | **✓** | - | - |
|  |  | Nystrom 1993^61^ | - | **✓** | - | - |
|  |  | Tabar 1984^98^ | - | - | **✓** | **✓** |
|  |  | Tabar 1985a^99^ | - | - | **✓** | **✓** |
|  |  | Tabar 1985b^100^ | - | **✓** | **✓** | **✓** |
|  |  | Tabar 1989^101^ | **✓** | **✓** | - | - |
|  |  | Tabar 1992^102^ | - | **✓** | **✓** | **✓** |
|  |  | Tabar 1995^103^ | - | **✓** | **✓** | **✓** |
|  |  | Tabar 1999^104^ | - | **✓** | - | - |
|  |  | Tabar 2000^105^ | - | **✓** | **✓** | **✓** |
|  |  | Tabar 2002^106^ | - | **✓** | - | - |
|  |  | Tabar 2011^107^ | - | **✓** | - | - |
|  |  | Yen 2012^108^ | - | - | **✓** | - |
| Breast | UK Age | Anderson 2004^109^ | - | - | **✓** | **✓** |
|  |  | Duffy 2020^110^ | **✓** | **✓** | **✓** | **✓** |
|  |  | Moss 2005^111^ | - | - | **✓** | **✓** |
|  |  | Moss 2006^112^ | **✓** | **✓** | - | - |
|  |  | Moss 2015^113^ | **✓** | **✓** | - | - |
| Cervical | Finnish | Anttila 2011^114^ | **✓** | **✓** | **✓** | **-** |
| Cervical | Mumbai | Shastri 2014^115^ | **✓** | **✓** | **✓** | **✓** |
| Cervical | Osmanabad | Sankaranarayanan 2005^116^ | - | - | **✓** | - |
|  |  | Sankaranarayanan 2009^117^ | - | **✓** | **✓** | **✓** |
| Cervical | TamilNadu | Sankaranarayanan 2004^118^ | - | - | **✓** | **✓** |
|  |  | Sankaranarayanan 2007^119^ | **✓** | **✓** | **✓** | **✓** |
| Liver | Qidong | Chen 2003^120^ | **✓** | **✓** | **✓** | **✓** |
| Liver | Shanghai | Zhang 2004^121^ | - | **✓** | **✓** | **✓** |
| Lung | Czech Study | Kubik 1986^122^ | **✓** | **✓** | **✓** | **✓** |
|  |  | Kubik 1990a^123^ | **✓** | **✓** | **✓** | **✓** |
|  |  | Kubik 1990b^124^ | - | **✓** | - | - |
|  |  | Kubik 2000^125^ | - | **✓** | - | - |
| Lung | DANTE | Infante 2008^126^ | - | - | **✓** | **✓** |
|  |  | Infante 2009^127^ | **✓** | **✓** | **✓** | **✓** |
|  |  | Infante 2015^128^ | **✓** | **✓** | **✓** | **✓** |
| Lung | DLCST | Saghir 2012^129^ | **✓** | **✓** | **✓** | **✓** |
|  |  | Wille 2016^130^ | **✓** | **✓** | **✓** | **✓** |
| Lung | ITALUNG | Paci 2017^131^ | **✓** | **✓** | **✓** | **✓** |
|  |  | Paci 2021^132^ | - | - | **✓** | **✓** |
|  |  | Puliti 2019^133^ | **✓** | **✓** | - | - |
| Lung | Johns Hopkins | Doria-Rose 2009^134^ | - | **✓** | **✓** | **✓** |
|  |  | Frost 1984^135^ | - | - | **✓** | **✓** |
|  |  | Levin 1982^136^ | - | **✓** | **✓** | **✓** |
| Lung | LSS | Doroudi 2018^137^ | **✓** | **✓** | - | - |
|  |  | Gohagan 2004^138^ | - | - | **✓** | **✓** |
|  |  | Gohagan 2005^139^ | - | - | **✓** | **✓** |
| Lung | LUSI | Becker 2015^140^ | - | - | **✓** | **✓** |
|  |  | Becker 2020^141^ | **✓** | **✓** | **✓** | **✓** |
|  |  | Gonzalez Maldonado 2021^142^ | **✓** | **✓** | - | - |
| Lung | Mayo Lung Project | Baker 2008^143^ | - | **✓** | - | - |
|  |  | Doria-Rose 2009^144^ | **✓** | **✓** | - | - |
|  |  | Fontana 1978^145^ | **✓** | **✓** | **✓** | **✓** |
|  |  | Fontana 1985^146^ | **✓** | **✓** | **✓** | **✓** |
|  |  | Fontana 1991^147^ | - | **✓** | **✓** | **✓** |
|  |  | Marcus 2000^148^ | **✓** | **✓** | - | - |
|  |  | Sanderson 1982^149^ | - | **✓** | **✓** | **✓** |
|  |  | Taylor 1981^150^ | - | **✓** | **✓** | **✓** |
| Lung | Memorial Sloan Kettering | Doria-Rose 2009^134^ | - | **✓** | **✓** | **✓** |
|  |  | Flehinger 1984^151^ | - | - | **✓** | **✓** |
|  |  | Melamed 1984^152^ | **✓** | **✓** | **✓** | **✓** |
|  |  | Melamed 1987^153^ | - | **✓** | - | - |
| Lung | MILD | Infante 2017^154^ | **✓** | **✓** | **-** | - |
|  |  | Pastorino 2012^155^ | **✓** | **✓** | - | - |
|  |  | Pastorino 2019a^156^ | **✓** | **✓** | **✓** | **✓** |
|  |  | Pastorino 2019b^157^ | **✓** | **✓** | **✓** | **✓** |
|  |  | Sverzellati 2016^158^ | - | - | **✓** | **✓** |
| Lung | NELSON | de Koning 2020^159^ | **✓** | **✓** | **✓** | **✓** |
| Lung | NLST | NLST Research Team 2011^160^ | **✓** | **✓** | **✓** | **✓** |
|  |  | NLST Research Team 2019^161^ | **✓** | **✓** | **✓** | **✓** |
|  |  | Pinsky 2013^162^ | **✓** | **✓** | **-** | **-** |
| Lung | PLCO (Lung) | Oken 2011^163^ | - | **✓** | **✓** | **✓** |
|  |  | Pinsky 2019^31^ | **✓** | **✓** | - | - |
|  |  | Prorok 2018^32^ | **✓** | - | - | - |
| Lung | UKLS | Field 2021^164^ | **✓** | **✓** | **✓** | **✓** |
| Nasopharyngeal | China NPC | Ji 2019^165^ | **✓** | **✓** | **✓** | **✓** |
| Oral | Trivandrum | Ramadas 2003^166^ | - | **✓** | **✓** | **✓** |
|  |  | Sankaranarayanan 2000^167^ | - | - | **✓** | **✓** |
|  |  | Sankaranarayanan 2005^168^ | - | **✓** | **✓** | **✓** |
|  |  | Sankaranarayanan 2013^169^ | - | **✓** | **✓** | **✓** |
| Ovarian | PLCO (Ovarian) | Buys 2011^170^ | - | **✓** | **✓** | **✓** |
|  |  | Pinsky 2016^171^ | - | **✓** | - | - |
|  |  | Pinsky 2019^31^ | **✓** | **✓** | - | - |
|  |  | Prorok 2018^32^ | **✓** | - | - | - |
|  |  | Temkin 2017^172^ | - | - | **-** | - |
| Ovarian | UK Pilot | Jacobs 1999^173^ | - | **✓** | **✓** | **✓** |
| Ovarian | UKCTOCS | Jacobs 2016^174^ | **✓** | **✓** | **✓** | **✓** |
|  |  | Menon 2021^175^ | - | **✓** | **✓** | **✓** |
| Prostate | CAP | Martin 2018^176^ | **✓** | **✓** | **✓** | **✓** |
| Prostate | ERSPC | Aus 2007^177^ | - | - | **✓** | **✓** |
|  |  | Berenguer 2003^178^ | **✓** | - | **✓** | **✓** |
|  |  | Boevee 2010^179^ | - | - | **✓** | **✓** |
|  |  | Franlund 2022^180^ | **✓** | **✓** | **✓** | **✓** |
|  |  | Hugosson 2004^181^ | - | - | **✓** | **✓** |
|  |  | Hugosson 2010^182^ | **✓** | **✓** | **✓** | **✓** |
|  |  | Hugosson 2018^183^ | **✓** | **✓** | **✓** | **✓** |
|  |  | Hugosson 2019^184^ | **✓** | **✓** | **✓** | **✓** |
|  |  | Kilpelainen 2010^185^ | - | - | **✓** | **✓** |
|  |  | Kilpelainen 2013^186^ | **✓** | **✓** | **✓** | **✓** |
|  |  | Lujan 2014^187^ | **✓** | **✓** | - | - |
|  |  | Lujan 2015^188^ | **✓** | **✓** | **✓** | **✓** |
|  |  | Lujan 2020^189^ | **✓** | **✓** | **✓** | **✓** |
|  |  | Makinen 2003^190^ | - | - | **✓** | **✓** |
|  |  | Pakarainen 2019^191^ | **✓** | **✓** | - | - |
|  |  | Postma 2006^192^ | - | - | **✓** | **✓** |
|  |  | Roobol 2013^193^ | - | **✓** | **✓** | **✓** |
|  |  | Schroder 2009^194^ | **✓** | **✓** | **✓** | **✓** |
|  |  | Schroder 2012^195^ | **✓** | **✓** | **✓** | **✓** |
|  |  | Schroder 2014^196^ | **✓** | **✓** | **✓** | **✓** |
|  |  | van der Cruijsen-Koeter 2005^197^ | - | - | **✓** | **✓** |
|  |  | Van Leeuwen 2010^198^ | **✓** | **✓** | - | - |
|  |  | Villers 2020^199^ | **✓** | **✓** | **✓** | **✓** |
| Prostate | ERSPC Pilot 1 | Osses 2019^200^ | **✓** | **✓** | **✓** | **✓** |
| Prostate | Norrkoping | Sandblom 2004^201^ | - | **✓** | **✓** | **✓** |
|  |  | Sandblom 2011^202^ | - | **✓** | **✓** | **✓** |
| Prostate | PLCO (Prostate) | Andriole 2009^203^ | **✓** | **✓** | **✓** | **✓** |
|  |  | Andriole 2012^204^ | - | **✓** | **✓** | **✓** |
|  |  | Crawford 2011^205^ | - | **✓** | **✓** | **✓figure** |
|  |  | Pinsky 2012^206^ | - | - | **✓** | **✓** |
|  |  | Pinsky 2017^207^ | - | **✓** | - | - |
|  |  | Pinsky 2019a^31^ | **✓** | **✓** | - | - |
|  |  | Pinsky 2019b^208^ | - | - | **✓** | **✓** |
|  |  | Pinsky 2019c^209^ | - | - | **✓** | **✓** |
|  |  | Prorok 2018^32^ | **✓** | - | - | - |
| Multiple* | D’Aquapendente | Prandoni 2016^210^ | **✓** | **✓** | **✓** | **✓** |
| Multiple* | MVTEP | Robin 2016^211^ | **✓** | **✓** | **✓** | **✓** |
| Multiple* | SOMIT | Piccioli 2004^212^ | **-** | **✓** | **✓** | **✓** |

**Trial name abbreviations.** CAP, Cluster Randomized Trial of PSA Testing for Prostate Cancer; CNBSS, Canadian National Breast Screening Study; DANTE, Detection and Screening of Early Lung Cancer by Novel Imaging Technology and Molecular Essays Trial; DLCST, Danish Lung Cancer Screening Trial; ERSPC, European Randomized Study of Screening for Prostate Cancer; HIP NY, Health Insurance Plan of Greater New York; ITALUNG, Italian Lung Cancer Screening Trial; LSS, Lung Screening Study; LUSI, German Lung Cancer Screening Intervention Trial; MILD, Multicentric Italian Lung Detection; MLP, Mayo Lung Project; MSK; Memorial Sloan Kettering Lung Study; MVTEP, Standard Diagnostic Procedures With or Without Fludeoxyglucose F 18 Positron Emission Tomography in Finding Cancer in Patients With a Blood Clot in a Vein; NELSON, Nederlands–Leuvens Longkanker Screenings Onderzoek; NLST, National Lung Screening Trial; NORCCAP, Norwegian colorectal cancer prevention; NordICC, Nordic-European Initiative on Colorectal Cancer; PLCO, Prostate, Lung, Colorectal and Ovarian Cancer Screening Trial; SCORE, Screening for COlon Rectum trial; SOMIT, Subsequent diagnosis Of Malignancy in patients presenting with Idiopathic venous Thromboembolism; UKCTOS, UK Collaborative Trial of Ovarian Cancer Screening; UKFSST, UK Flexible Sigmoidoscopy Screening Trial; UKLS, UK Lung Cancer Screening Trial.

**✓**, Outcome available; - Outcome not available.

* Different target cancers screened for in each trial.

# Section 3. Detailed information on trials and the reported outcomes for the assessment of mortality outcome surrogacy

## Table S12. Included trials (n=57): description of the populations.

| **Cancer type** | **Trial acronym/ name** | **Country**  **(no. of centres)** | **Calendar years of randomisation/enrolment** | **Population type (general/ high risk)** | **Sex and age inclusion criteria** | **Randomisation type and timing regarding consent** | **N randomised to intervention arm** | **N randomised to comparator arm** |
| --- | --- | --- | --- | --- | --- | --- | --- | --- |
| Bowel | Finnish ^1-4^ | Finland  (multicentre) | 2004-2012 (extended to 2014) | General | Men and women;  60-69 years | Individual;  before consent | Enrolled 2004-2012:  181,080 | Enrolled 2004-2012:  181,085 |
| Bowel | Funen ^5-12^ | Denmark  (1 centre) | Jan 1985 | General | Men and women;  45-74 years | Individual;  before consent | 30,967 | 30,966 |
| Bowel | Gothenburg ^13-16^ | Sweden  (1 centre) | Cohort 1: 1982  Cohort 2: 1987  Cohort 3: 1990 | General | Men and women;  60-64 years | Individual;  before consent | Cohorts 1-3:  34,144 | Cohorts 1-3:  34,164 |
| Bowel | Minnesota ^17-19^ | USA  (1 centre) | 1975-1978 | General | Men and women;  50-80 years | Individual;  after consent | 31,157 -  annual: 15,570  biennial: 15,587 | 15,394 |
| Bowel | NORCCAP ^20-22^ | Norway  (2 centres) | Jan 1999-Dec 2000 (55-64 years)  Jan-Dec 2001 (50-54 years) | General | Men and women;  50-64 years | Individual;  before consent | 20,780 -  FS only: 10,392  FS and FOBT: 10,388 | 79,430 |
| Bowel | NordICC ^23^ | Poland, Norway, Sweden  (Netherlands)  (multicentre) | 2009-2014 | General | Men and women;  55-64 years | Individual;  before consent | Poland, Norway, Sweden:  28,395 | Poland, Norway, Sweden:  56,784 |
| Bowel | Nottingham ^24-29^ | UK (England)  (1 centre) | Pilot study: Feb 1981-June 1983  Main study: Feb 1985-Jan 1991 | General | Men and women;  Pilot study: 45-74 years; Main study: 50-74 years | Individual;  before consent | 76,466 | 76,384 |
| Bowel | PLCO (bowel) ^30-33^ | USA  (10 centres) | Nov 1993-July 2001 | General | Men and women;  55-74 years | Individual;  after consent | 77,443 | 77,444 |
| Bowel | SCORE ^34-36^ | Italy  (6 centres) | June 1995-May 1999 | General | Men and women;  55-64 years | Individual or cluster (depending on centre);  after consent | 17,148 | 17,144 |
| Bowel | Telemark ^37-40^ | Norway  (1 centre) | Mar-Apr 1983 | General | Men and women;  50-59 years | Individual;  before consent | 400 | 399 |
| Bowel | UKFSST ^41-43^ | UK (England, Wales, Scotland)  (14 centres) | Nov 1994-Mar 1999 | General | Men and women;  55-64 years | Individual;  after consent | 57,237 | 113,195 |
| Breast | CNBSS-1 ^45 46 48 50 51^ | Canada  (15 centres) | Jan 1980-end of Mar 1985 | General | Women;  40-49 years | Individual;  after consent | 25,214 | 25,216 |
| Breast | CNBSS-2 ^44 47 49 50^ | Canada  (15 centres) | Jan 1980-end of Mar 1985 | General | Women;  50-59 years | Individual;  after consent | 19,711 | 19,694 |
| Breast | Edinburgh ^52-56^ | UK (Scotland)  (1 centre) | Cohort 1: June 1979 (1 practice Sep 1978)-Dec 1981  Cohort 2: 1982-1983  Cohort 3: 1984-1985 | General | Women;  45-64 years | Cluster;  after consent | 28,654 -  Cohort 1: 22,944  Cohorts 2-3: 5,710 | 26,017 -  Cohort 1: 21,344  Cohorts 2-3: 4,673 |
| Breast | Gothenburg ^57-62^ | Sweden  (1 centre) | Dec 1982-Apr 1984 | General | Women;  39-59 years | Individual^a^ (1936-1944 cohorts) or cluster (1923-1935 cohorts);  before consent | 22,158 | 30,675 |
| Breast | HIP NY ^63-75^ | USA  (23 medical groups) | Dec 1963 through 1964 | General | Women;  40-64 years | Individual;  before consent | ~31,000 | ~31,000 |
| Breast | Malmo ^60-62 76-81^ | Sweden  (1 centre) | Oct 1976-1978 | General | Women;  45-69 years | Individual;  before consent | 21,088 | 21,195 |
| Breast | Mumbai ^82^ | India  (1 centre) | May 1998-Apr 2002 | General | Women;  35-64 years | Cluster;  before consent | 75,360 | 76,178 |
| Breast | Russia / WHO ^83-85^ | Russia  (multicentre) | 1985-1989 | General | Women;  40-64 years | Cluster;  before consent | Leningrad:  57,712 | Leningrad:  64,759 |
| Breast | Shanghai ^86 87^ | China  (519 factories) | Oct 1989-Oct 1991 | General | Women;  Born 1925 through 1958. | Cluster;  before consent | 133,682 | 133,718 |
| Breast | Stockholm ^60-62 80 88-92^ | Sweden  (1 centre) | Mar 1981-May 1983 | General | Women;  40-64 years | Individual;  before consent | 40,318 | 19,943 |
| Breast | Trivandrum ^93 94^ | India  (multicentre) | Initiated on 01/01/2006 | General | Women;  30-69 years | Cluster;  before consent | 55,843 | 59,447 |
| Breast | Two-County ^60 61 95-108^ | Sweden  (multicentre) | Kopparberg (Dalarna) County:  July 1977-Feb 1980;  Ostergotland County:  May 1978-Mar 1981 | General | Women;  40+ years  (40-74 years in most analyses) | Cluster;  before consent | 94,390 -  Kopparberg:  47,389  Ostergotland: 47,001 | 68,591 -  Kopparberg:  22,658  Ostergotland:  45,933 |
| Breast | UK Age ^109-113^ | UK (England, Wales, Scotland)  (23 centres) | Oct 1990-Sep 1997 | General | Women;  39-41 years | Individual;  before consent | 53,914 | 107,007 |
| Cervical | Finnish ^114^ | Finland  (multicentre) | Jan 1999-Dec 2003 | General and high-risk | Women;  30-60 years  (25-65 years in some municipalities) | Individual;  before consent | 174,912 | 345,400 |
| Cervical | Mumbai ^115^ | India  (1 centre) | May 1998-Apr 2002 | General | Women;  35-64 years | Cluster;  before consent | 75,360 | 76,178 |
| Cervical | Osmanabad ^116 117^ | India  (multicentre) | Initiated Jan 2000 | General | Women;  30-59 years | Cluster;  before consent | VIA: 34,087  cytology: 32,077  HPV: 34,136 | 31,506 |
| Cervical | Tamil Nadu ^118 119^ | India  (multicentre) | Started in Oct 1999 | General | Women;  30-59 years | Cluster;  before consent | 49,320 | 30,962 |
| Liver | Qidong ^120^ | China  (multicentre) | Oct 1989: 15 townships;  Apr 1992: Additional 8 townships | High-risk | Men;  30-69 years | Cluster;  unclear timing | 3,712 | 1,869 |
| Liver | Shanghai ^121^ | China  (multicentre) | Jan 1993-Dec 1995 | High-risk | Men and women;  35-59 years | Cluster;  before consent | 9,757 | 9,443 |
| Lung | Czech Study ^122-125^ | Czech Republic  (multicentre) | June 1976-June 1977 | High-risk | Men;  40-64 years | Individual;  after consent | 3,171 | 3,174 |
| Lung | DANTE ^126-128^ | Italy  (multicentre) | Mar 2001-Feb 2006 | High-risk | Men;  60-74 years | Individual;  before consent | 1,403 | 1,408 |
| Lung | DLCST ^129 130^ | Denmark  (1 centre) | Oct 2004-Mar 2006 | High-risk | Men and women;  50-70 years | Individual;  after consent | 2,052 | 2,052 |
| Lung | ITALUNG ^131-133^ | Italy  (3 centres) | 2004-2006 | High-risk | Men and women;  55-69 years | Individual;  after consent | 1,613 | 1,593 |
| Lung | Johns Hopkins ^134-136^ | USA  (1 centre) | 1973-Jan 1978 | High-risk | Men;  45+ years | Individual;  after consent | 5,226 | 5,161 |
| Lung | LSS ^137-139^ | USA  (6 centres) | Sep 2000-mid Nov 2000 | High-risk | Men and women;  55-74 years | Individual;  after consent | 1,660 | 1,658 |
| Lung | LUSI ^140-142^ | Germany  (1 centre) | Oct 2007-Apr 2011 | High-risk | Men and women;  50-69 years | Individual;  after consent | 2,029 | 2,023 |
| Lung | Mayo Lung Project ^143-150^ | USA  (1 centre) | Nov 1971-July 1976 | High-risk | Men;  45+ years | Individual;  after consent | 4,618 | 4,593 |
| Lung | Memorial Sloan Kettering ^134 151-153^ | USA  (1 centre) | June 1974-Jan 1978 | High-risk | Men;  45+ years | Individual;  after consent | 4,968 | 5,072 |
| Lung | MILD ^154-158^ | Italy  (1 centre) | Sep 2005-Jan 2011 | High-risk | Men and women;  49-75 years | Individual^b^;  after consent | 2,376 -  annual: 1,190  biennial: 1,186 | 1,723 |
| Lung | NELSON ^159^ | Belgium, Netherlands  (multicentre) | Dec 2003 through July 2006 | High-risk | Men and women;  50-74 years | Individual;  after consent | 7,900 | 7,892 |
| Lung | NLST ^160-162^ | USA  (33 centres) | Aug 2002 through Apr 2004 | High-risk | Men and women;  55-74 years | Individual;  after consent | 26,722 | 26,732 |
| Lung | PLCO (lung) ^31 32 163^ | USA  (10 centres) | Nov 1993-July 2001 | General | Men and women;  55-74 years | Individual;  after consent | 77,443 | 77,444 |
| Lung | UKLS ^164^ | UK (England)  (2 centres) | Oct 2011-Feb 2013 | High-risk | Men and women;  50-75 years | Individual;  after consent | 2,028 | 2,027 |
| Naso-pharyngeal | China NPC ^165^ | China  (multicentre) | Interim analysis (Zhongshan City only):  2009-2014 | General | Men and women;  30-59 years | Cluster;  before consent | 71,415 | 50,659 |
| Oral | Trivandrum ^166-169^ | India  (multicentre) | Oct 1995-2004 | General | Men and women;  35+ years | Cluster;  before consent | 96,517 | 95,356 |
| Ovarian | PLCO (ovarian) ^31 32 170-172^ | USA  (10 centres) | Nov 1993-July 2001 | General | Women;  55-74 years | Individual;  after consent | 39,104 | 39,111 |
| Ovarian | UK Pilot ^173^ | UK (England)  (1 centre) | 1989 | General - volunteered for a previous prevalence screen | Women;  45+ years and postmenopausal | Individual;  after consent | 10,958 | 10,977 |
| Ovarian | UKCTOCS ^174 175^ | UK (England, Wales, NI)  (13 centres) | Apr 2001-Oct 2005 | General | Women;  50-74 years | Individual;  after consent | 101,279 -  CA125 (and TVS): 50,640  TVS: 50,639 | 101,359 |
| Prostate | CAP ^176^ | UK (England, Wales)  (8 centres) | 2001-2009 | General | Men;  50-69 years | Cluster;  before consent | 195,912 | 219,445 |
| Prostate | ERSPC ^177-199^ | Belgium, Finland, France,  Italy, Netherlands, Portugal, Spain, Sweden, Switzerland  (multicentre) | Belgium: June 1991-Dec 2003  Finland: Jan 1996-Jan 1999  France: 2000-2005  Italy: Oct 1996-Oct 2000  Netherlands: Nov 1993-Mar 2000  Portugal: discontinued Oct 2000  Spain: Feb 1996-June 1999  Sweden: June 1991-Dec 2003  Switzerland: Sept 1998-Aug 2003 | General | Men;  50-74 years;  core age group: 55-69 years | Individual;  before or after consent depending on centre/country | 125,268 -  Belgium: 5,188  Finland: 31,970  France: 42,452  Italy: 7,497  Netherlands: 21,206  Spain: 1,840  Sweden: 9,957  Switzerland: 5,158 | 141,244 -  Belgium: 5,171  Finland: 48,409  France: 42,061  Italy: 7,474  Netherlands: 21,162  Spain: 1,862  Sweden: 9,954  Switzerland: 5,151 |
| Prostate | ERSPC Pilot 1 ^200^ | Netherlands  (1 centre) | 1991-1992 | Low risk (PSA <10.0 ng/ml at baseline) | Men;  55-74 years | Individual;  after consent | 553 | 581 |
| Prostate | Norrkoping ^201 202^ | Sweden  (1 centre) | 1987 | General | Men;  50-69 years | Individual;  before consent | 1,494 | 7,532 |
| Prostate | PLCO (prostate) ^31 32 203-209^ | USA  (10 centres) | Nov 1993-July 2001 | General | Men;  55-74 years | Individual;  after consent | 38,340 | 38,343 |
| Multiple* | D’Aquapendente ^210^ | Italy  (5 centres) | NR (likely early 2010s) | High-risk | Men and women;  18+ years | Individual;  after consent | 98 | 97 |
| Multiple* | MVTEP ^211^ | France  (4 centres) | Mar 2009-Aug 2012 | High-risk | Men and women;  18+ years | Individual;  after consent | 200 | 199 |
| Multiple* | SOMIT ^212^ | Italy  (5 centres) | Jan 1993-Dec 1997 | High-risk | Men and women;  25+ years | Individual;  before consent | 99 | 102 |

**Abbreviations.** Apr, April; Aug, August; CA125, cancer antigen 125; Dec, December; Feb, February; FOBT, faecal occult blood test; FS, flexible sigmoidoscopy; HPV, human papillomavirus; Jan, January; Mar, March; NI; Northern Ireland; Nov, November; NPC, nasopharyngeal cancer; Oct, October; Sep, September; TVS, transvaginal ultrasound scan; VIA, visual inspection with acetic acid; WHO, World Health Organization.

**Trial name abbreviations.** CAP, Cluster Randomized Trial of PSA Testing for Prostate Cancer; CNBSS, Canadian National Breast Screening Study; DANTE, Detection and Screening of Early Lung Cancer by Novel Imaging Technology and Molecular Essays Trial, DLCST, Danish Lung Cancer Screening Trial; ERSPC, European Randomized Study of Screening for Prostate Cancer; HIP NY, Health Insurance Plan of Greater New York; ITALUNG, Italian Lung Cancer Screening Trial; LSS, Lung Screening Study; LUSI, German Lung Cancer Screening Intervention Trial; MILD, Multicentric Italian Lung Detection; MVTEP, Standard Diagnostic Procedures With or Without Fludeoxyglucose F 18 Positron Emission Tomography in Finding Cancer in Patients With a Blood Clot in a Vein; NELSON, Nederlands–Leuvens Longkanker Screenings Onderzoek; NLST, National Lung Screening Trial; NORCCAP, Norwegian colorectal cancer prevention; NordICC, Nordic-European Initiative on Colorectal Cancer; PLCO, Prostate, Lung, Colorectal and Ovarian Cancer Screening Trial; SCORE, Screening for COlon Rectum trial; SOME, Screening for Occult Malignancy in Patients with Idiopathic Venous Thromboembolism; SOMIT, Subsequent diagnosis Of Malignancy in patients presenting with Idiopathic venous Thromboembolism; UKCTOS, UK Collaborative Trial of Ovarian Cancer Screening; UKFSST, UK Flexible Sigmoidoscopy Screening Trial; UKLS, UK Lung Cancer Screening Trial.

* Different target cancers screened for in each trial.

^a^ Randomisation ratio 1 to 1.0-1.2 in the 39–49-years age group and 1 to 1.0-1.6 in the 50–59-years age group resulting in slight differences in age distribution between study and control groups.

^b^ Two recruitment methods: Initial randomisation to annual (n=326) or biennial (n=327) LDCT screening; final protocol randomised to LDCT arm (n=1,723; 864 annual and 859 biennial) or control arm (n=1,723). LDCT (combined) arm and control arm significantly different in age and gender.

## Table S13. Included trials (n=57): description of the interventions.

| **Cancer type** | **Trial acronym/ name** | **Intervention arm** | | | | **Control arm** | | | | |
| --- | --- | --- | --- | --- | --- | --- | --- | --- | --- | --- |
|  |  | **Intervention:**  **Screening test** | | | | **Intervention:**  **Another screening test** | | | | **Intervention:**  **No standardised screening** |
|  |  | **Screening test** | **Intended no. of screening rounds and interval** | **Timing of the 1^st^ and last screening round** | **Overall screening participation** | **Screening test** | **Intended no. of screening rounds and interval** | **Timing of the 1^st^ and last screening round** | **Overall screening participation** | **Contamination / exit screen: type and frequency** |
| Bowel | Finnish ^1-4^ | FOBT | Up to 5 (re-invitation until age 69) (biennial) | First: 2004.  Last: NA (routine population screening) | 2004-2011: 69.2%  2004-2012: 68.8% | NA | NA | NA | NA | Contamination: “likely to be small”; CRC screening not common.  Exit screen: No |
|  | Funen ^5-12^ | FOBT | Originally 3,  extended to 9  (biennial) | First: Aug 1985-Sep 1986  Last: NR (2000/2001?) | 9^th^ round:  91% of invited;  44% of subjects alive. | NA | NA | NA | NA | Contamination: “H-II has not been purchased for general use in the county of Funen during the screening.”  Exit screen: No |
|  | Gothenburg ^13-16^ | FOBT | 2 (Cohort 2),  3 (Cohorts 1 and 3).  T0-T1: 16-24 months;  T2-T3: ~8 years (Cohort 1);  21-24 months (Cohort 3). | First:  Aug 1982 (Cohort 1)  Jan 1987 (Cohort 2)  Jan 1990 (Cohort 3)  Last:  May 1995 (Cohort 1)  May 1989 (Cohort 2)  Nov 1993 (Cohort 3) | Cohorts 1-3:  Total: 61.8%  ≥1 test: 70.0%  All tests: 47.2% | NA | NA | NA | NA | Contamination: No CRC diagnosed with Hemoccult only.  Exit screen: No |
|  | Minnesota ^17-19^ | FOBT  (annual) | 11 (annual)  (Phase I: 5;  Phase II: 6) | First:  Phase I: Feb 1976  Phase II: Feb 1986  Last:  Phase I: Dec 1982  Phase II: Feb 1992 | Total 75.2%  ≥1 test 90.2%  All tests 46.2% | NA | NA | NA | NA | Contamination: 1.8% of patients with CRC ever had FOBT performed.  Exit screen: No |
|  |  | FOBT (biennial) | 6 (biennial)  (Phase I: 3;  Phase II: 3) |  | Total 78.4%  ≥1 test 89.9%  All tests 59.7% |  |  |  |  |  |
|  | NORCCAP ^20-22^ | FS (+/- FOBT; 1:1) | Single screen | Jan 1999-Dec 2000 (age 55-64);  Jan-Dec 2001  (age 50-54) | FS and FOBT: 60.9%;  FS only: 65.1%. | NA | NA | NA | NA | Contamination: Virtually no screening colonoscopies outside the trial. All CRC cases identified symptomatically.  Exit screen: No |
|  | NordICC ^23^ | Colonoscopy | Single screen | June 2009-June 2014 | 42.0%  (33.0% Poland -  60.7% Norway) | NA | NA | NA | NA | Contamination: No screening colonoscopies outside the trial.  Exit screen: No |
|  | Nottingham ^24-29^ | FOBT | 3-6 (biennial) | First: 1985?  Last: Feb 1995 | All tests 38.2%  ≥1 test 59.6% | NA | NA | NA | NA | Contamination: NR  Exit screen: No |
|  | PLCO (bowel) ^30-33^ | FS | 2 (3-5 years) | First: 1993  Last: NR | ≥1 FS 86.6%  2 FS 50.9% | NA | NA | NA | NA | Contamination: FS 25.8%, Colonoscopy 34.4%,  Either test 46.5%.  Exit screen: No  Post-screening phase:  years 11-18: FS 26.3%,  Colonoscopy 65.3%,  Either test 72.1%. |
|  | SCORE ^34-36^ | FS | Single screen | 1995-1999 | 57.8% | NA | NA | NA | NA | Contamination: Invitation to population-based CRC screening using immuno-chemical FOBT from 2006 (8 screen-detected CRC).  Exit screen: No |
|  | Telemark ^37-40^ | FS (+/- colonoscopy after 13 years) | 2-3  FS: 1-2  (Single screen or 2-year interval);  Colonoscopy (or FS) after 13 years from entry | First:  Mar/Apr 1983  Last:  FS: Apr 1985  Colonoscopy (or FS): 1996 | 1983: 81%  1985: 86%  1996: 75% | NA | NA | NA | NA | Contamination: 6.3% referred to colonoscopy in 1983-1993 (5% symptomatic; 1.3% family history).  Exit screen: Endoscopic examination (FS or a full colonoscopy) in 1996.  Uptake: 67% |
|  | UKFSST ^41-43^ | FS | Single screen | 1994-1999 | 71% | NA | NA | NA | NA | Contamination: NR  Exit screen: No.  Post-screening phase:  BCSP introduced in July 2006.  30.3% participated in BCSP.  45 CRC diagnosed via FOBT screening. |
| Breast | CNBSS-1 ^45 46 48 50 51^ | Mammography and CBE | 4-5 (annual) | First: 1980-1985  Last: June 1988 | T0 100%  Mean 4.2 mammograms | CBE | Single screen | 1980-1985 | 100% | NA |
|  | CNBSS-2 ^44 47 49 50^ | Mammography and CBE | 4-5 (annual) | First: 1980-1985  Last: June 1988 | T0 100%  T1-T4 >85% | CBE | 4-5 (annual) | First: 1980-1985  Last: June 1988 | T0 100%  T1 89.1%  T4 85.4% | NA |
|  | Edinburgh ^52-56^ | Mammography and CBE | Cohort 1: 7  (4 MMG)  Cohort 2: 5  (3 MMG)  Cohort 3: 3  (2 MMG)  MMG and CBE: years 0, 3, 5, 7  CBE: years 2, 4, 6 | First: within 1 year of entry.  Last: 1988 | T0 61.3%  T1 54.6%  T2 53.1%  T6 44.1%.  Last screen overall: just over 50%. | NA | NA | NA | NA | Contamination:  Little screening available during the study period.  Exit screen: No  Post-screening phase:  Service screening in the NHS introduced gradually since June 1988. Uptake NR. |
|  | Gothenburg ^57-62^ | Mammography | 4-5 (18-month interval) | First: Dec 1982-May 1984  Last: Oct 1987 | 75-85%  (T0 83-85% according to age;  Rescreens 76-78%) | NA | NA | NA | NA | Contamination: Believed to be high; MMG in previous 2 years: 18% in a random sample.  Exit screen: yes (Nov 1987-June 1991).  Uptake: 66% (64-78% depending on age group) |
|  | HIP NY ^63-75^ | Mammography and CBE | 4 (annual) | First: Dec 1963 through June 1966  Last: June 1970 | Of those who attended T0 (67%):  All tests: 60%  2-3 tests: 28%  Only T0: 12% | NA | NA | NA | NA | Contamination:  MMG not routinely included in general physical examination.  Exit screen: No |
|  | Malmo ^60-62 76-81^ | Mammography | 6-8  (every 18-24 months) | First: Oct 1976-Sept 1978  Last: Trial ended 31/12/1986 but screening continued for women <70 years (up to 31/12/1989) | T0 74%  Rescreens 70% | NA | NA | NA | NA | Contamination:  24% MMG during the study period, most only once.  20% of breast cancers first detected by MMG.  Exit screen: yes (Oct 1992-Feb 1993); women born between 1908-1922 not invited. Uptake NR.  Post-screening phase:  Service screening programme from 1990; women born 1908-1922 not invited. |
|  | Mumbai ^82^ | CBE | 4 (biennial) | First: 1998  Last: Dec 2007 | Mean 67.1%  ≥1 CBE: 94.8% | NA | NA | NA | NA | Contamination: NR  Exit screen: No |
|  | Russia / WHO ^83-85^ | BSE | Monthly BSE;  2-4 BSE education sessions (every 3 years) | First: 1985-1989  Last: BSE ongoing;  NR for BSE education sessions | ≥5 BSE/year  56-82%;  Monthly BSE 18-54% (depending on year).  BSE education: NR | NA | NA | NA | NA | Contamination: No BSE public education programme; limited possibilities to obtain information on BSE.  Exit screen: No |
|  | Shanghai ^86 87^ | BSE | Monthly BSE;  BSE instruction: 3  (T0 and ~1 and ~3 years after T0);  Supervised BSE practice sessions: 11-13  (3-5 in year 1, then every 6 months);  Monthly reminders | Baseline BSE instruction:  Oct 1989-Oct 1991.  Last: BSE ongoing;  Supervised BSE and reinforcement activities:  1993-July 1995. | BSE: NR  BSE instruction:  T0: 98.5%  T1: 95.1%  T2: 83.1%  All 3: 79.2%.  Supervised BSE:  mean 12 times in 4-5 years of the trial, roughly every 4-5 months. | NA | NA | NA | NA | Contamination: 3.6% may have received information about BSE (transfer to instruction group factory).  3.6% of the tumours initially found by CBE.  1.7% of breast cancer patients reported ever having received training on BSE.  Exit screen: No |
|  | Stockholm ^60-62 80 88-92^ | Mammography | 2 (biennial) | First: Mar 1981-June 1983  Last: Sep 1983-Oct 1985 | T0 80.7%  T1 80% | NA | NA | NA | NA | Contamination:  MMG before first invitation to the screening program: ~20% (mostly symptomatic).  Exit screen: yes, after T0 and T1 completed (Oct 1985-Dec 1986); uptake: 77% |
|  | Trivandrum^93 94^ | CBE | 3 (every 3 years) | First: 2006  Last: 2017 | T0 90.2%  T1 62.1%  T2 57.3%  ≥1 test 95.7% | NA | NA | NA | NA | Contamination: CBE or MMG screening almost non-existent in routine care in the study setting.  Exit screen: No |
|  | Two-County ^60 61 95-108^ | Mammography | 2-4  (40-49 years at entry: 2-year interval;  50+ years at entry: 3-year interval) | First: Kopparberg: July 1977-Feb 1980.  Ostergotland: May 1978-Mar 1981.  Last: 1984-1985. | Average 85% | NA | NA | NA | NA | Contamination: 13% MMG as part of routine medical care up to 1984.  Exit screen: Yes (1984-1985), women ≥70 years not invited.  Uptake: 79.9-90.4% depending on age. |
|  | UK Age ^109-113^ | Mammography | 8 (annual) | First: 1990-1991  Last: 2006 | ≥1 test: 81%  Mean 4.8 (SD 3.3) screens attended | NA | NA | NA | NA | Contamination: 4% had MMG in previous 3 years (sample of 2,000 women).  Exit screen: All women eligible to join NHSBSP between the ages of 50-52 (triennial MMG). Uptake: NR |
| Cervical | Finnish ^114^ | Papnet | 1-2 (every 5 years; ongoing screening programme) | First: 1999-2003  Last: 2007 | T0 71.8% | Conventional cytology | 1-2 (every 5 years; ongoing screening programme) | First: 1999-2003  Last: 2007 | T0: 72.0% | NA |
|  | Mumbai ^115^ | VIA | 4 (biennial) | First: 1998  Last: Dec 2007 | T0 71.5%  T1 61.5%  T2 57.8%  T3 58.1%  ≥1 screen 89% | NA | NA | NA | NA | Contamination: NR  Exit screen: No |
|  | Osmanabad ^116 117^ | VIA | Single screen | Jan 2000-Nov 2003 (Apr 2003) | 71.9% (78.5%) | NA | NA | NA | NA | Contamination: 6% requested screening.  Exit screen: No |
|  |  | Conventional cytology | Single screen | Jan 2000-Nov 2003 (Apr 2003) | 72.9% (79.5%) |  |  |  |  |  |
|  |  | HPV | Single screen | Jan 2000-Nov 2003 (Apr 2003) | 69.5% (79.7%) |  |  |  |  |  |
|  | Tamil Nadu ^118 119^ | VIA | Single screen | Jan 2000-Apr 2003 | 63.6% | NA | NA | NA | NA | Contamination: 3.1% sought VIA screening from 2000-2006.  Exit screen: No |
| Liver | Qidong ^120^ | AFP and ALT | 6 (enrolled Oct 1989)  3 (enrolled Apr 1992)  (6-monthly) | First: Oct 1989 or Apr 1992  Last: Apr 1993 | All tests:  23.5%  (enrolled 1989);  40.6%  (enrolled 1992).  T0 only: 10.8% | AFP and ALT | Single screen | Oct 1989 or Apr 1992 | NR | NA |
|  | Shanghai ^121^ | AFP and US | 5-10 (6-monthly) | First: Jan 1993  Last: Dec 1997 | All tests: 58.2%  Median 5 (range 1-10) rounds completed. | NA | NA | NA | NA | Contamination: NR  Exit screen: No |
| Lung | Czech Study ^122-125^ | CXR and sputum cytology | 9 (6-monthly CXR and sputum cytology for 3 years;  annual CXR years 4-6) | First: June 1976-June 1977  Last: 6 years after baseline screen (1982/1983) | Years 1-3:  mean 92.5%  (range, 89.9-95.1%).  Years 4-6:  mean 91.2%. | CXR and sputum cytology | 5 (CXR and sputum cytology at T0 and after 3 years;  annual CXR years 4-6) | First: June 1976-June 1977  Last: 6 years after baseline screen (1982/1983) | Year 3: 94.7%;  Years 4-6: mean 93% | NA |
|  | DANTE ^126-128^ | LDCT and clinical review (baseline: + CXR and sputum cytology) | 5 (annual) | First: Mar 2001-Feb 2006  Last: NR | ≥3 scans: 96.8%  All 5 scans: 93.7% | Clinical review (baseline:  + CXR  and sputum cytology) | 5 (annual) | First: Mar 2001-Feb 2006  Last: NR | Jan 2008:  ≥1 test 89%  ≥2 tests 62%  ≥3 tests 37%  ≥4 tests 15% | NA |
|  | DLCST ^129 130^ | LDCT | 5 (annual) | First: Oct 2004-Mar 2006  Last: Mar 2010 | Mean 95.5% | NA | NA | NA | NA | Contamination: 7.5% had ≥1 chest CT scans outside the trial during the 4-year trial duration.  Exit screen: No |
|  | ITALUNG ^131-133^ | LDCT | 4 (annual) | First: NR  Last: NR | 80.7% compliant with protocol | NA | NA | NA | NA | Contamination: NR  Exit screen: No |
|  | Johns Hopkins ^134-136^ | CXR and sputum cytology | CXR: 6-8 (annual)  Sputum cytology: 16-22 (every 4 months) | First: 1973  Last: NR | Overall (5 years):  CXR: 76%  Sputum cytology: 67%  T6 <50%.  T7 ~20%. | CXR | 6-8 (annual) | First: 1973  Last: NR | Overall (5 years): 79%  T6 <50%.  T7 ~20%. | NA |
|  | LSS ^137-139^ | LDCT | 2 (annual) | First: completed Jan 2001  Last: Dec 2001 | T0 95.5%  T1 85.8%  Overall: 90.7% | CXR | 2 (annual) | First: completed Jan 2001  Last: Dec 2001 | T0 93.5%  T1 79.9%  Overall: 86.7% | NA |
|  | LUSI ^140-142^ | LDCT | 5 (annual) | First: Oct 2007  Last: May 2016 | ≥3 scans 93%  ≥4 scans 90%  All 5 scans 84% | NA | NA | NA | NA | Contamination: Screening CXR or LDCT during active screening period: 4.8%.  Exit screen: No.  Post-screening phase:  Screening CXR or LDCT after active screening period: 8.2% |
|  | Mayo Lung Project ^143-150^ | CXR and sputum cytology | 18 (4-monthly) | First: Nov 1971-Jul 1976  Last: July 1982 | 6-year screening period: average 75% | NA | NA | NA | NA | Contamination:  6-year intervention period: 26.4% of cancers detected by non-study CXR (incidental findings); 73.6% by symptoms.  Exit screen: No |
|  | Memorial Sloan Kettering ^134 151-153^ | CXR and sputum cytology | CXR: 6-9 (annual)  Sputum cytology: 16-25 (4-monthly) | First: July 1974  Last: Oct 1982 | Overall (first 5 years):  CXR 77%  Sputum cytology 76%  T6 <50%  T7 ~20% | CXR | 6-9 (annual) | First: July 1974  Last: Oct 1982 | Overall (first 5 years): 79%  T6 <50%  T7 ~20% | NA |
|  | MILD ^154-158^ | LDCT  (annual) | 7 (annual) | First: 2005  Last: Median duration of LDCT screening 6.2 years (IQR 5.5-6.4). | 96.1% adherent;  ≥1 scan: 96.8%;  Median 7 scans (June 2018) | NA | NA | NA | NA | Contamination: 1.2%, including 1 cancer diagnosis and 1 death (unknown cause).  Exit screen: No |
|  |  | LDCT (biennial) | 4 (biennial) |  | 95.1% adherent;  ≥1 scan: 97.0%;  Median 4 scans (June 2018) |  |  |  |  |  |
|  | NELSON ^159^ | LDCT | 4 (1, 2 and 2.5-year intervals) | First: Jan 2004  Last: Dec 2012 | Male participants: Mean 90.0%  3 rounds attended: 87.6% | NA | NA | NA | NA | Contamination: Low risk because of the health care system structure.  Exit screen: No |
|  | NLST ^160-162^ | LDCT | 3 (annual) | First: Aug 2002  Last: Sep 2007 | ≥1 screen: 99.0%  Across 3 rounds: 95% | CXR | 3 (annual) | First: Aug 2002  Last: Sep 2007 | ≥1 screen 98.1%  Across 3 rounds: 93% | NA |
|  | PLCO (lung) ^31 32 163^ | CXR | 4 (annual) | First: 1993  Last: 2004 | Overall 83.5%,  ≥1 CXR 91.2% | NA | NA | NA | NA | Contamination: 11% during the screening phase of the trial.  Exit screen: No |
|  | UKLS ^164^ | LDCT | Single screen | “Soon” after randomisation | 98.3% | NA | NA | NA | NA | Contamination: NR  Exit screen: No |
| Naso-pharyngeal | China NPC ^165^ | EBV serology and indirect mirror and lymphatic palpation | Single screen | 2008 in Sihui City; 2009 in Zhongshan City | Interim analysis, 1 city only:  41.8% | NA | NA | NA | NA | Contamination: NR  Exit screen: No |
| Oral | Trivandrum ^166-169^ | Visual inspection | 4 (3-year interval) | First: 1996-1998  Last: 2006-2009 | Overall (1996-2009) 92.0% | NA | NA | NA | NA | Contamination: NR  Exit screen: Yes (2006-2009).  Uptake: 46.1% |
| Ovarian | PLCO (ovarian)^31 32 170-172^ | CA125 +/- TVU | CA125 blood test: 4-6 (annual)  TVU: 4 (annual for first 3 years) | First: 1993  Last: NR | T0 85% CA125; 84% TVU.  T3 79% CA125; 78% TVU.  T4 75% CA125.  T5 73% CA125. | NA | NA | NA | NA | Contamination:  CA125: 2.3%-3.2% per year;  TVU 2.7%-4.6% per year.  Exit screen: No  Post-screening period:  CA125 in prior year: 1.8%  TVU in prior year: 2.9%  Ever had CA125: 7.5%  Ever had TVU: 14.9% |
|  | UK Pilot ^173^ | CA125 (and TVU as 2^nd^ tier) | 3 (annual) | First: 1989?  Last: 1991? | All screens: 70.7%  ≥1 screen 85.5% | NA | NA | NA | NA | Contamination: NR  Exit screen: No |
|  | UKCTOCS ^174 175^ | CA125 (and TVU as 2^nd^ tier) | 6 (annual), extended to 7-11 | First: 2001-2005  Last: Dec 2011 | Total 80.8% | NA | NA | NA | NA | Contamination: 4.3% (2014 questionnaire)  Exit screen: No |
|  |  | TVU | 6 (annual), extended to 7-11 | First: 2001-2005  Last: Dec 2011 | Total 78.0% |  |  |  |  |  |
| Prostate | CAP ^176^ | Blood PSA | Single screen | 2001-2009 | 36% had blood sample taken | NA | NA | NA | NA | Contamination:  ~10%-15% over 10 years.  Exit screen: No |
|  | ERSPC ^177-199^ | Blood PSA +/-DRE +/- TRUS (depending on centre) | Core age group (55-69 years):  2-8 rounds.  Sweden: 3-10 rounds.  4-year interval: 5/8 centres;  2-year interval: Sweden, France;  7-year interval: Belgium between T0 and T1, then 4 years. | First: At randomisation (ranged from 1993 in Netherlands and Belgium to 2003 in France).  Last: Stopping age 67-78 years depending on centre; duration of screening ranged from 4 years to 16 years. | Centres with consent before randomisation: 88-100%;  Centres with consent after randomisation: 62-68%.  ≥1 screen by 31/12/2014: 83% excluding France (64% including France) | NA | NA | NA | NA | Contamination: ~20% per year underwent PSA screening during the early follow-up period.  Exit screen: No  Post-screening phase:  Sweden: after 18 years of follow-up, almost 40% of cancers diagnosed through opportunistic screening. |
|  | ERSPC Pilot 1 ^200^ | Blood PSA | Up to 5 (4-year interval) | First: 1991/1992  Last: Up to age 74 years (2010/2011?) | NR | NA | NA | NA | NA | Contamination: Cumulative PSA contamination rate +/- 4.5%, with the first 4 years rate of 1.8%.  Exit screen: No |
|  | Norrkoping ^201 202^ | DRE only first, later DRE and blood PSA | 4 (3-year interval; T3 invitation of men ≤69 years only) | First: 1987  Last: 1996 | Average over 4 rounds: 74% | NA | NA | NA | NA | Contamination: NR  Exit screen: No |
|  | PLCO (prostate) ^31 32 203-209^ | Blood PSA and DRE | 6 for PSA  (T0-T5, annual)  4 for DRE  (T0-T3, annual) | First: 1993-2001  Last: Oct 2006 | Overall 85% for PSA and 86% for DRE. | NA | NA | NA | NA | Contamination: 45% had ≥1 PSA test in the 3 years preceding randomisation.  Screening period (year 0-5):  PSA: 40% in the first year; increased to 52% in the year 5.  DRE: 41-46%.  ≥1 screening PSA: 74%.  Exit screen: No  Post-screening period: PSA test within the past year for screening 45.9%; for any reason 54.6%.  Through 13 years of follow-up: 88% received any PSA testing. |
| Multiple* | D’Aquapendente ^210^ | Torso CT and FOBT | Single screen | Within 4 weeks of inclusion. | CT: 100%  FOBT: 98% | NA | NA | NA | NA | Contamination:  CT (not as first line test) 14%  FOBT 33%.  Exit screen: No |
|  | MVTEP ^211^ | Limited screening and PET-CT | Single screen | Within 1 month of VTE diagnosis (Mar 2009-Aug 2012) | PET-CT: 88% | NA | NA | NA | NA | Contamination:  PET-CT 0%  Abdominal US and thoracic-abdominal pelvic CT 0.5%.  Exit screen: No |
|  | SOMIT ^212^ | Extensive screening including torso CT | Single screen | Within 4-week period from VTE diagnosis (Jan 1993-Dec 1997) | Complete battery of tests: 80% | NA | NA | NA | NA | Contamination:  ≥1 screening test 22.5%.  Exit screen: No |

**Abbreviations.** AFP, alpha-fetoprotein; ALT, alanine aminotransferase; Apr, April; Aug, August; BCSP, NHS Bowel Cancer Screening Programme; BSE, breast self-examination; CA125, cancer antigen 125; CBE, clinical breast examination; CRC, colorectal cancer; CT, computed tomography; CXR; chest X-ray; Dec, December; DRE, digital rectal examination; EBV, Epstein-Barr virus; Feb, February; FOBT, faecal occult blood test; FS, flexible sigmoidoscopy; H-II, hemoccult II; HPV, human papillomavirus; Jan, January; LDCT, low-dose computed tomography; Mar, March; MMG; mammography; NA, not applicable; NHS, National Health Service; NHSBSP, National Health Service Breast Screening Programme; Nov, November; NPC, nasopharyngeal cancer; NR, not reported; Oct, October; PET-CT, positron emission tomography – computed tomography; PSA, prostate-specific antigen; SD, standard deviation; Sep, September; T0, baseline screen; T1, first rescreen; T2, second rescreen; T3, third rescreen, T4, fourth rescreen; T5, fifth rescreen; T6, sixth rescreen; TRUS, transrectal ultrasound; TVU, transvaginal ultrasound; US, ultrasound; VIA, visual inspection with acetic acid; VTE, venous thromboembolism; WHO, Word Health Organization.

**Trial name abbreviations.** See Table S12 legend.

* Different target cancers screened for in each trial.

## Table S14. “Main” mortality and “primary” late-stage endpoints (with rationale for the choice of the time point); analysis investigating the absolute incidence of late-stage cancer (57 trials).

| **Cancer type** | **Trial acronym/ name** | **“Primary” intermediate outcome (Absolute incidence of late-stage cancer)** | | | **“Main” mortality outcome** | | |
| --- | --- | --- | --- | --- | --- | --- | --- |
|  |  | **Definition and timing** | **Estimated effect**  **(95% CI)** | **Rationale for choice of timepoint** | **Definition and timing** | **Estimated effect**  **(95% CI)** | **Rationale for choice of timepoint** |
| Bowel | Finnish | Incidence of CRC with N≥1 (left and right colon) in individuals randomised from 2004-2011;  FU until 31/12/2011 (relative risk)^2^ | 1.07  (0.90-1.27) | Intervention is ongoing when mortality is measured. Same FU as mortality used for the late-stage outcome. | CRC mortality in individuals randomised from 2004-2012;  FU until 31/12/2012 (median 4.5, range 0.0-8.3 years) (rate ratio)^4^ | 1.04  (0.84-1.28) | Only timepoint available |
| Bowel | Funen | Incidence of late-stage CRC (Dukes’ C, distant spread, no classification);  FU until Aug 1995 (mean 9.1 years) (rate ratio)^10^ | 0.84  (0.70-1.01) | Alternative timepoints are soon after randomisation, screening still ongoing. | CRC mortality (deaths from CRC or complications from treatment);  FU until Aug 1995 (mean 9.1 years) (rate ratio)^10^ | 0.82  (0.68-0.99) | Originally 3 screening rounds over 5-year period followed by 5 years of passive FU. |
| Bowel | Gothenburg | Incidence of Dukes’ D CRC; all cohorts;  FU until 31/12/2001 (mean 15.5 years) (rate ratio)^16^ | 0.95  (0.76-1.18) | Screening for all cohorts was not completed until May 1995 (cohort 1). | CRC mortality; all cohorts;  FU until 31/12/2001 (mean 15.5 years) (rate ratio)^16^ | 0.84  (0.71-0.99) | Only timepoint available |
| Bowel | Minnesota | Incidence of Dukes’ D CRC;  13 years of FU from entry (rate ratio)  annual FOBT vs. UC^17^ | 0.50  (0.33-0.76) | Only timepoint available | CRC mortality;  13 years of FU from entry;  annual FOBT vs. UC (rate ratio)^17^ | 0.67  (0.51-0.89) | Decision to publish results after review of most recent data on CRC mortality showed stopping boundary had been crossed in the annually screened group.  Through year 13, last complete year of FU. |
|  |  | Incidence of Dukes’ D CRC;  13 years of FU from entry (rate ratio)  biennial FOBT vs. UC^17^ | 0.62  (0.42-0.92) |  | 13 years of FU from entry;  biennial FOBT vs. UC (rate ratio)^17^ | 0.96  (0.74-1.23) |  |
| Bowel | NORCCAP | Incidence of Dukes’ C or distant spread CRC in people aged 55-64 years;  FU until 31/12/2006 (median 7 years (range 6-8) of FU) (relative risk)^20^ | 0.90  (0.70-1.15) | Single screen up to Dec 2001. Timepoint provides sufficient FU after intervention and is earlier than mortality. | CRC mortality in people aged 55-64 years;  FU until 31/12/2015 (median 15 years) (hazard ratio)^22^ | 0.82  (0.66-1.02) | Planned end points were cumulative incidence and mortality of colorectal cancer after 5, 10, and 15 years. Original NORCCAP included 55-64 year olds. Chosen to fit UKFSST (15 years). |
| Bowel | NordICC | Incidence of Dukes’ C or D CRC;  median 10-year FU from entry (relative risk)^23^ | 0.80  (0.65-1.00) | Only timepoint available | CRC mortality;  10-year risk (median 10.0 years) (Kaplan-Meier estimate)^23^ | 0.91  (0.64-1.16) | Only timepoint available |
| Bowel | Nottingham | Incidence of Dukes’ C or D CRC;  FU until 30/06/1995 (median 7.8 years) (rate ratio)^25^ | 0.91  (0.80-1.04) | 3-6 screening rounds (biennial), so screening is ongoing for minimum up to 4 years since entry (Feb 1995). Mean FU of alternative timepoint is before screening has ended. | CRC mortality (verified cause of death);  FU until 30/06/1995 (median 7.8 years) (rate ratio)^25^ | 0.85  (0.74-0.98) | Hardcastle 1989^24^ reported that both groups would be followed up for at least 7 years. |
| Bowel | PLCO (bowel) | Incidence of stage III-IV CRC;  13 years of FU from entry or until 31/12/2009 (rate ratio)^33^ | 0.71  (0.62-0.81) | Only timepoint available | CRC mortality;  13 years of FU from entry or until 31/12/2009 (rate ratio)^33^ | 0.74  (0.63-0.87) | Main analysis timepoint: 13 years of FU or until 31/12/2009. |
| Bowel | SCORE | Incidence of stage III-IV CRC;  FU until 31/12/2007 (median 10.5 years) (rate ratio)^35^ | 0.73  (0.57-0.94) | Only timepoint available | CRC mortality;  FU until 31/12/2008 (5 centres) or 31/12/2007 (1 centre) (median 11.4 years) (rate ratio)^35^ | 0.78  (0.56-1.08) | A statistically significant reduction in mortality was expected to be detected after 11 years of FU. |
| Bowel | Telemark | Incidence of Dukes’ C or D CRC;  FU until 1993 (relative risk)^37^ | 0.50  (0.05-5.48) | 1-2 rounds  (single screen or 2-year interval); screening would have ended after 2 years of FU. Alternative timepoint provides sufficient FU and is earlier than mortality. | CRC mortality;  FU until 1995 (relative risk)^39^ | 0.33  (0.03-3.18) | No SAP or power calculation.  “Specified and all-cause mortality from 1983 up to and including 1995 were end points in this study…”  Chose 1995 to not include the exit screen. |
| Bowel | UKFSST | Incidence of CRC (all sites);  FU until 31/12/2008 (mean 10.8 years) (hazard ratio)^41^ | 0.77  (0.70-0.84) | Only timepoint available | CRC mortality (death verified by expert coder);  FU until 31/12/2008 (median 11.2 years) (hazard ratio)^41^ | 0.68  (0.59-0.80) | Higher than expected attendance rates; revised estimates suggested that the required number of endpoints to show a significant difference would be achieved at 11 years. |
| Breast | CNBSS-1 | Incidence of node-positive breast cancers;  7 years of FU (relative risk)^45^ | 1.55  (1.13-2.11) | Same timepoint as chosen mortality outcome.  Node-positivity was chosen over size to be consistent with what has been used in this analysis for other breast cancer trials. | Breast cancer mortality; 7 years of FU (relative risk)^45^ | 1.36  (0.84-2.21) | 5 years from entry; however, because insufficient deaths from breast cancer had occurred by 5 years to attain the planned power, FU was extended for 2 years, by which time there were enough breast cancer deaths to reach the planned power. |
| Breast | CNBSS-2 | Incidence of node-positive breast cancers:  7 years of FU (relative risk)^44^ | 1.09  (0.82-1.45) | As CNBSS-1 | Breast cancer mortality;  7 years of FU (relative risk)^44^ | 0.97  (0.62-1.52) | As CNBSS-1 |
| Breast | Edinburgh | Incidence of stage III-IV breast cancer (Cohort 1);  7 years of FU from entry (mean 6.8 years) (rate ratio)^56^ | 0.63  (0.46-0.87) | Only timepoint available. | Breast cancer mortality (Cohort 1);  7 years of FU from entry (mean 6.8 years) (rate ratio)^56^ | 0.84  (0.60-1.16) | 7 years of FU used for power calculations. |
| Breast | Gothenburg | Incidence of lymph-node positive, invasive breast cancer detected in screening period (mean 6 years) (relative risk)^59^ | 0.80  (0.61-1.05) | Same cancers included as in mortality outcome | Breast cancer mortality in cases diagnosed in screening period (blinded review of cause of death);  FU until 31/12/1996 (up to 14 years) (relative risk)^58^ | 0.78  (0.57-1.06) | Primary outcome: Deaths of cases diagnosed in screening period up to 31/12/1996. |
| Breast | HIP NY | Incidence of lymph-node positive breast cancer;  FU for 5 years from entry (relative risk)^68^ | 0.84  (0.65-1.10) | Screening ended at 3 years of FU from entry (4 rounds, annually; June 1970). Screening still ongoing for alternative timepoint. | Breast cancer mortality;  FU for 10 years from entry (rate ratio)^63^ | 0.78  (0.63-0.96) | Minimum of 5 years FU after the screening program has been completed. Everything else sounds post hoc. |
| Breast | Malmo | Incidence of stage II-IV breast cancer;  FU until 31/12/1986 (mean 8.8 years) (rate ratio)^78^ | 0.83  (0.68-1.00) | Intervention is ongoing when mortality is measured. Same FU as mortality used for the late-stage outcome. | Breast cancer mortality;  FU until 31/12/1986 (mean 8.8 years) (rate ratio)^78^ | 0.96  (0.68-1.35) | Predetermined end of trial was 31/12/1986; no interim analyses have been performed. |
| Breast | Mumbai | Incidence of stage III-IV breast cancer;  FU until May 2018 (March 2019) (median 18 years) (rate ratio)^82^ | 0.81  (0.68-0.97) | Only timepoint available | Breast cancer mortality;  FU until May 2018 (March 2019) (median 18 years) (rate ratio)^82^ | 0.85  (0.70-1.02) | Study was planned for a period of 16 years to include 4 screening rounds followed by 4 monitoring rounds. |
| Breast | Russia/WHO | Incidence of lymph-node positive breast cancer;  FU from 1985-1994 (relative risk)^85^ | 1.20  (1.01-1.43) | First intervention received between 1985-1989 and a min of 2 sessions (up to 4) were provided in 3-year intervals. Participants that received their first session in 1989 are still expected to receive their 2^nd^ session in 1992, so after our alternative timepoint. | Breast cancer mortality in cases diagnosed until 31/12/1994;  FU until 31/12/1997 (relative risk)^85^ | 1.07  (0.86-1.34) | 5 years and 10 years FU.  Registration of new breast cancer cases will be continued up to 1994, with follow-up until 1999. Only timepoint available (at least in English language). |
| Breast | Shanghai | Incidence of lymph-node positive breast cancer;  FU until 31/12/2000 (mean 10 years) (relative risk)^87^ | 0.92  (0.79-1.07) | Only timepoint available | Breast cancer mortality;  FU until 31/12/2000 (mean 10 years) (rate ratio)^87^ | 1.04  (0.82-1.33) | Total trial duration of 10 years. |
| Breast | Stockholm | Incidence of stage II-IV breast cancer detected in screening period (including exit screen) (1981-1986; up to 6 years) (relative risk)^92^ | 0.88  (0.68-1.12) | Intervention ended in 1985, followed by exit screen 1985-1986. Alternative timepoints do not have sufficient FU after the intervention has ended, therefore selecting the timepoint that includes the exit screen. | Breast cancer mortality in cases diagnosed in screening period (including exit screen);  FU until 31/12/1994 (rate ratio)^92^ | 0.74  (0.50-1.10) | SAP or protocol not reported.  FU until 31/12/1994 seems to be the main analysis time point for mortality. |
| Breast | Trivandrum | Incidence of stage III-IV breast cancer;  FU until 31/12/2019 (mean 12 years) (rate ratio)^93^ | 1.18  (0.92-1.52) | Screening did not end until 2017. Alternative timepoint too early. | Breast cancer mortality;  FU until 31/12/2019 (mean 12 years) (rate ratio)^93^ | 1.03  (0.75-1.41) | Only timepoint available |
| Breast | Two-County | Incidence of stage II-IV breast cancer in women aged 40-74 years;  FU until 31/12/1984 (screening period) (mean 6 years) (relative risk)^100^ | 0.79  (0.69-0.91) | Intervention is ongoing when mortality is measured. Same FU as mortality used for the late-stage outcome. | Breast cancer mortality in women aged 40-74 years;  FU until 31/12/1984 (mean 6 years) (rate ratio)^100^ | 0.72  (0.54-0.98) | First mortality outcome reported. |
| Breast | UK Age | Incidence of lymph-node positive, invasive breast cancer; diagnosed during the intervention period (before first NHSBSP screen) (mean 10.6 years) (rate ratio)^110^ | 0.90  (0.78-1.05) | Screening did not end until 2006 / 7. Alternative timepoint too early. | Breast cancer mortality in cases diagnosed during the intervention period (before first NHSBSP screen);  FU until 31/12/2011 (median 17.7 years) (rate ratio)^113^ | 0.88  (0.74-1.04) | Primary endpoint was mortality from breast cancers diagnosed during the intervention phase of the trial. Power calculations revised to 14 years of FU; originally 10 years of FU. |
| Cervical | Finnish | Incidence of invasive cervical cancer;  FU until 31/12/2007 (mean 6.3 years) (rate ratio)^114^ | 1.00  (0.76-1.29) | Only timepoint available | Cervical cancer mortality;  FU until 31/12/2007 (mean 6.3 years) (rate ratio)^114^ | 1.11  (0.62-1.92) | Only timepoint available |
| Cervical | Mumbai | Incidence of stage ≥IIB invasive cervical cancer;  FU until 31/12/2011 (mean 8.0 years; up to 12 years) (rate ratio)^115^ | 0.84  (0.63-1.12) | Only timepoint available | Cervical cancer mortality;  FU until 31/12/2011 (mean 8.0 years; up to 12 years) (rate ratio)^115^ | 0.69  (0.50-0.94) | 16 years since enrolment.  However, the observed mortality rate in the control group was higher than assumed, which enabled us to detect a statistically significant reduction in mortality earlier than planned, at the end of 12 years. |
| Cervical | Osmanabad | Incidence of stage II+ invasive cervical cancer;  FU until 31/12/2007 (mean 8 years) (rate ratio) - VIA vs. UC^117^ | 0.97  (0.72-1.32) | Screening has just ended in Nov 2003. Not sufficient time after end of intervention for alternative timepoint. | Cervical cancer mortality;  FU until 31/12/2007 (mean 8 years) (rate ratio) - VIA vs. UC^117^ | 0.81  (0.57-1.16) | Within 15 years of enrolment. Only timepoint available. |
|  |  | FU until 31/12/2007 (mean 8 years) (rate ratio) - cytology vs. UC^117^ | 0.70  (0.50-0.98) |  | FU until 31/12/2007 (mean 8 years) (rate ratio) - cytology vs. UC^117^ | 0.83  (0.58-1.20) |  |
|  |  | FU until 31/12/2007 (mean 8 years) (rate ratio) - HPV vs. UC^117^ | 0.44  (0.30-0.64) |  | FU until 31/12/2007 (mean 8 years) (rate ratio) - HPV vs. UC^117^ | 0.49  (0.32-0.74) |  |
| Cervical | Tamil Nadu | Incidence of stage II+ invasive cervical cancer;  FU until 31/12/2006 (mean 5.6-5.8 years) (rate ratio)^119^ | 0.70  (0.53-0.92) | Screening has just ended in 30/04/2003. Not sufficient time after end of intervention for alternative timepoint. | Cervical cancer mortality;  FU until 31/12/2006 (up to 7 years) (rate ratio)^119^ | 0.59  (0.44-0.79) | The observed mortality rate in the control population during the study was much higher than assumed, which enabled us to observe a significant effect on cancer rate and mortality 7 years from the beginning of the study, rather than waiting for 10 years, as predicted in the power calculations.  Only timepoint available. |
| Liver | Qidong | Incidence of stage III liver cancer (Chinese HCC staging system);  FU until 31/12/1995 (mean 62-63 months) (rate ratio)^120^ | 0.54  (0.37-0.81) | Only timepoint available | Liver cancer mortality;  FU until 31/12/1995 (mean 62-63 months) (rate ratio)^120^ | 1.02  (0.81-1.29) | Only timepoint available |
| Liver | Shanghai | Incidence of stage III liver cancer (Chinese HCC staging system);  FU until 31/12/1997 (mean 4.1-4.3 years) (relative risk)^121^ | 0.53  (0.32-0.88) | Only timepoint available | Liver cancer mortality;  FU until 31/12/1997 (mean 4.1-4.3 years) (rate ratio)^121^ | 0.63  (0.41-0.98) | Only timepoint available |
| Lung | Czech Study | Incidence of stage III lung cancer (incident) (clinical-diagnostic staging system with occult, I, II and III stages);  FU for 6 years from entry (relative risk)^123^ | 1.15  (0.78-1.71) | Screening was completed at 6y from entry. Screening is ongoing at the alternative timepoints (and just ended for selected). | Lung cancer mortality;  15 years of FU from entry (relative risk)^125^ | 1.14  (0.96-1.36) | No reference to SAP or power calculations.  Chose timepoint consistent with PLCO.  Used all cancers as cancers diagnosed in first 3 or 6 years seem not their primary analysis. |
| Lung | DANTE | Incidence of stage II-IV lung cancer;  FU until 15/05/2013 (median 8.35 years) (relative risk)^128^ | 0.94  (0.64-1.38) | Last recruited was in Feb 2006 and there were 5 rounds of screening (annually). Screening is still ongoing for some participants for the alternative timepoint. | Lung cancer mortality;  FU until 15/05/2013 (median 8.35 years) (rate ratio)^128^ | 1.00  (0.69-1.44) | Power calculation of 5 years of FU.  The only other available timepoint is until 25/01/2008 (median 35.7 and 31.5 months). |
| Lung | DLCST | Incidence of stage III-IV lung cancer;  FU until 07/04/2015 (median 9.8 years) (relative risk)^130^ | 1.12  (0.74-1.70) | Intervention ended in March 2010, not sufficient FU after the end of intervention for alternative timepoint. | Lung cancer mortality;  FU until 07/04/2015 (median 9.8 years) (rate ratio)^130^ | 1.03  (0.66-1.61) | FU time from power calculations was 10 years from randomisation. |
| Lung | ITALUNG | Incidence of stage III-IV lung cancer;  FU until 31/12/2013 (median 8.5 years) (relative risk)^131^ | 0.76  (0.48-1.19) | Alternative timepoint after mortality. | Lung cancer mortality;  FU until 31/12/2014 (median 9.3 years) (rate ratio)^131^ | 0.70  (0.47-1.03) | SAP: 10 years after randomisation |
| Lung | Johns Hopkins | Incidence of stage II-IV lung cancer;  FU for up to 9 years from entry (mean 7.2 years) (relative risk)^134^ | 0.86  (0.68-1.09) | Alternative timepoint only provides information on prevalence screen. | Lung cancer mortality;  FU for up to 9 years from entry (mean 7.2 years) (rate ratio)^134^ | 0.83  (0.67-1.04) | No SAP or power calculation;  chose longest FU time available; otherwise, late-stage outcome would have been measured after mortality, now it is at the same timepoint. |
| Lung | LSS | Incidence of stage III-IV lung cancer;  1 year FU from entry (relative risk)^139^ | 1.78  (0.79-4.01) | Only timepoint available | Lung cancer mortality;  FU until 31/12/2005 (median 5.2 years) (rate ratio)^137^ | 1.24  (0.74-2.08) | Only timepoint available |
| Lung | LUSI | Incidence of stage II-IV lung cancer;  7 years of FU since entry (relative risk)^141^ | 0.55  (0.34-0.88) | Halfway point between 4y since entry (last screen) and 10y since entry (median mortality FU). | Lung cancer mortality;  FU until 30/04/2018 (within 11 years of randomisation; median 8.9 years) (relative risk)^141^ | 0.72  (0.45-1.16) | From trial registration: Primary outcome measure is mortality from lung cancer at 5 and 10 years. |
| Lung | Mayo Lung Project | Incidence of stage III-IV lung cancer;  FU until 01/07/1983 (6-year screening period plus 1-5.5 years post-screen) (relative risk)^147^ | 0.98  (0.75-1.27) | Intervention did not end until July 1982. All alternative timepoints are earlier than that. | Lung cancer mortality;  FU until 01/07/1983 (6-year screening period plus 1-5.5 years post-screen) (relative risk)^147^ | 1.06  (0.82-1.36) | Mayo Lung Project ended on 01 July 1983. |
| Lung | Memorial Sloan Kettering | Incidence of stage II-IV lung cancer;  FU for up to 9 years from entry (mean 7.2 years) (relative risk)^134^ | 1.01  (0.77-1.33) | Alternative timepoint does not have sufficient FU after intervention has ended (Oct 1982). | Lung cancer mortality;  FU for up to 9 years from entry (mean 7.2 years) (rate ratio)^134^ | 0.95  (0.73-1.25) | No SAP or power calculation available.  Chose timepoint with longest follow-up; same follow-up time as late-stage outcome. |
| Lung | MILD | Incidence of stage II-IV lung cancer;  FU until June 2018 (mean 9.4-9.6 years) (rate ratio) - LDCT (combined) vs. UC^157^ | 0.74  (0.50-1.10) | Screening period is ~6 years since entry. Alternative timepoint does not have sufficient FU after intervention has ended (for most participants <6y) | Lung cancer mortality;  FU until June 2018 (mean 9.7 years) (rate ratio) - LDCT (combined) vs. UC ^157^ | 0.70  (0.45-1.09) | 10 years of FU.  Chose longest follow-up available; same analysis date as the chosen late-stage outcome. |
| Lung | NELSON | Incidence of stage III-IV lung cancer in men;  FU for 10 years from entry or until 31/12/2015 (mean 9.5 years) (relative risk)^159^ | 0.71  (0.58-0.87) | Only timepoint available. | Lung cancer mortality in men;  FU for 10 years from entry or 31/12/2015 (mean 9.5 years) (rate ratio)^159^ | 0.76  (0.61-0.94) | 10 years of FU in men. |
| Lung | NLST | Incidence of stage III-IV lung cancer;  FU until 31/12/2009 (median 6.5 years) (relative risk)^160^ | 0.79  (0.70-0.89) | Not sufficient FU after end of screening for alternative timepoints. | Lung cancer mortality;  FU until 31/12/2009 (median 6.5 years) (rate ratio)^162^ | 0.84  (0.75-0.95) | SAP: 15/01/2009 for LC mortality (time lags associated with the end point verification process). LC mortality results subsequently reported using all events through 31/12/2009 (same cut-off date as for all-cause mortality). |
| Lung | PLCO (lung) | Incidence of stage III-IV NSCLC;  FU for 7 years from entry (rate ratio)^163^ | 0.94  (0.84-1.05) | 4 annual screens - closest point between end of screening (3y fu) and mortality (median 11.9). | Lung cancer mortality;  FU for 13 years or 31/12/2009 (median 11.9 years) (rate ratio)^163^ | 0.99  (0.91-1.07) | 13 years or 31/12/2009, whichever came first. |
| Lung | UKLS | Incidence of stage III-IV lung cancer;  FU for 4 years from entry (relative risk)^164^ | 0.44  (0.23-0.87) | Single screen soon after randomisation (Oct 2011-Feb 2013). Halfway timepoint from randomisation to mortality timepoint (median 7.3y). | Lung cancer mortality;  FU until 29/02/2020 (median 7.3 years) (rate ratio)^164^ | 0.65  (0.41-1.02) | SAP: For the initial FU report, the FU period is up to 29 February 2020 for mortality and up to 31 December 2019 for incidence of lung cancer. |
| Naso-pharyngeal | China NPC | Incidence of stage III-IV NPC;  FU until 31/12/2014 (median 6.0 years) (relative risk)^165^ | 0.78  (0.57-1.07) | Only timepoint available | NPC mortality;  FU until 31/12/2014 (median 6.0 years) (rate ratio)^165^ | 0.81  (0.45-1.45) | Only timepoint available |
| Oral | Trivandrum | Incidence of stage III-IV oral cancer;  FU until 31/12/2004 (mean 4.4-4.9 years) (rate ratio)^168^ | 0.89  (0.68-1.16) | Intervention is ongoing (last: 2006-2009) when mortality is measured. Same FU as mortality. | Oral cancer mortality;  FU until 31/12/2004 (mean 4.4-4.9 years; up to 9 years) (rate ratio)^168^ | 0.79  (0.51-1.22) | 12 year of enrolment (10 years of FU?).  First time they have reported mortality. Before exit screen (which was performed 2006-2009). |
| Ovarian | PLCO (ovarian) | Incidence of stage III-IV ovarian cancer (including primary peritoneal and fallopian tube cancer);  FU for 13 years from entry or until 28/02/2010 (median 12.4 years) (rate ratio)^170^ | 1.20  (0.96-1.51) | Not sufficient FU after end of screening for alternative timepoint. | Ovarian cancer (including primary peritoneal and fallopian tube cancer) mortality;  FU for 13 years from entry or until 28/02/2010 (median 12.4 years) (rate ratio)^170^ | 1.18  (0.91-1.54) | Up to 13 years from randomisation or 28 February 2010, whichever came first. |
| Ovarian | UK Pilot | Incidence of stage III-IV ovarian cancer;  FU until Dec 1997 (relative risk)^173^ | 0.61  (0.29-1.30) | Only timepoint available | Ovarian cancer mortality;  FU until January 1998 (up to 8 years) (relative risk)^173^ | 0.50  (0.19-1.28) | Only timepoint available |
| Ovarian | UKCTOCS | Incidence of stage III-IV primary ovarian cancer;  FU until 31/12/2014 (mean 10.8 years) (rate ratio) - MMS vs. UC^174^ | 0.83  (0.70-0.99) | Alternative timepoint after mortality. | Ovarian cancer mortality;  FU until 31/12/2014 (median 11.1 years) (rate ratio) - MMS vs. UC^174^ | 0.85  (0.70-1.03) | Original protocol:  7 years after randomisation.  Extended FU until 31/12/2014; FU extended again to 30/06/2020.  Same analysis date as for the late-stage outcome. |
|  |  | Incidence of stage III-IV primary ovarian cancer;  FU until 31/12/2014 (mean 10.8 years) (rate ratio) - TVS vs. UC^174^ | 0.91  (0.77-1.08) | Alternative timepoint after mortality. | Ovarian cancer mortality;  FU until 31/12/2014 (median 11.1 years) (rate ratio) - TVS vs. UC^174^ | 0.89  (0.73-1.07) |  |
| Prostate | CAP | Incidence of prostate cancer with Gleason score 8-10;  <6 years of FU since randomisation (relative risk)^176^ | 1.00  (0.90-1.11) | Halfway point between 18 months (screening period) to 10 years (median mortality) | Definite, probable, or intervention-related prostate cancer mortality;  FU until 31/03/2016 (median 10 years) (rate ratio)^176^ | 0.96  (0.86-1.08) | Defined in trial protocol/SAP as primary outcome and used for power calculation. |
| Prostate | ERSPC | Incidence of prostate cancer with Gleason score 8-10 in men aged 55-69 years; whole trial excluding France and Portugal;  FU until 31/12/2008 (median 11.0 years) (relative risk)^195^ | 0.82  (0.74-0.91) | Intervention is ongoing when mortality is measured. Same FU as mortality used for the late-stage outcome. | Prostate cancer mortality in men aged 55-69 years; whole trial excluding France and Portugal;  FU until 31/12/2008 (mean 10.5 years) (rate ratio)^195^ | 0.79  (0.68-0.91) | FU through 2008 main analysis time point. |
| Prostate | ERSPC Pilot 1 | Incidence of prostate cancer with Gleason score ≥3+4;  FU for median 19 years from entry (IQR 12-24) (relative risk)^200^ | 0.86  (0.50-1.48) | Only timepoint available | Prostate cancer mortality;  FU for median 19 years from entry (IQR 12-24) (relative risk)^200^ | 0.48  (0.17-1.36) | Only timepoint available |
| Prostate | Norrkoping | Incidence of advanced tumours (T3-4, N1 or MX/M1);  FU until 31/12/1999 (relative risk)^202^ | 0.87  (0.62-1.23) | Only timepoint available | Prostate cancer mortality in cancers diagnosed up to 31/12/1999;  FU until 31/12/2008 (20 years) (relative risk)^202^ | 1.16  (0.78-1.73) | 20-year FU used in power calculations. |
| Prostate | PLCO (prostate) | Incidence of prostate cancer with Gleason score 8-10;  FU through 10 years (relative risk)^203^ | 0.85  (0.73-0.99) | Intervention period ended at 5 years since randomisation. The alternative timepoint provides sufficient FU after the end of the intervention and is before the mortality endpoint. | Prostate cancer mortality;  FU for 13 years from entry or until 31/12/2009 (mean 11.1 years) (rate ratio)^204^ | 1.09  (0.87-1.36) | Reported as the planned follow-up in the “Design of the PLCO trial” publication. |
| Multiple* | D’Aquapendente | Incidence of stage IV cancer;  24 months FU from entry (relative risk)^210^ | 0.74  (0.17-3.23) | Only timepoint available | Cancer-specific mortality;  24 months FU (relative risk)^210^ | 0.49  (0.09-2.64) | Only timepoint available |
| Multiple* | MVTEP | Incidence of “advanced” cancer;  24 months FU from entry (relative risk)^211^ | 0.71  (0.23-2.21) | Only timepoint available | Cancer-specific mortality;  24 months FU (relative risk)^211^ | 0.40  (0.08-2.04) | Only timepoint available |
| Multiple* | SOMIT | Incidence of cancer with N1 and/or M1;  24 months FU from entry (relative risk)^212^ | 0.64  (0.22-1.90) | Only timepoint available | Cancer-specific mortality;  24 months FU (relative risk)^212^ | 0.52  (0.10-2.75) | Only timepoint available |

**Abbreviations.** CI, confidence interval; CRC, colorectal cancer; Dec, December; FOBT, faecal occult blood test; Feb, February; FU, follow-up; HCC, hepatocellular cancer; HPV, human papillomavirus; IQR, interquartile range; LC, lung cancer; LDCT, low-dose computed tomography; M1, distant metastasis present; MMS, multimodal screening; MX, metastases cannot be measured; N1, cancer has spread to nearby lymph nodes; N1, N2, N3, number of nearby lymph nodes that have cancer; NHSBSP, NHS Breast Screening Programme; NPC, nasopharyngeal cancer; NSCLC, non-small cell lung cancer; Oct, October; SAP, statistical analysis plan; T3, tumour size or area: cancer has broken through the capsule (covering) of the prostate gland; T4, tumour size or area: cancer has spread into other body organs nearby; TVS, transvaginal ultrasound; UC, usual care; VIA, visual inspection with acetic acid; WHO, Word Health Organization.

**Trial name abbreviations.** See Table S12 legend.

* Different target cancers screened for in each trial.

## Table S15. “Main” mortality and “primary” late-stage endpoints (with rationale for the choice of the time point); analysis investigating the proportion of late-stage cancer (55 trials).

| **Cancer type** | **Trial acronym/ name** | **“Primary” intermediate outcome (Proportion of cancers diagnosed at late stage)** | | | **“Main” mortality outcome** | | |
| --- | --- | --- | --- | --- | --- | --- | --- |
|  |  | **Definition and timing** | **Estimated effect (95% CI)** | **Rationale for choice of timepoint** | **Definition and timing** | **Estimated effect (95% CI)** | **Rationale for choice of timepoint** |
| Bowel | Finnish | % of CRC that were node-positive (N≥1) at diagnosis in individuals randomised from 2004-2011  FU until 31/12/2011^2^ | 0.89 (0.78-1.01) | Intervention is ongoing when mortality is measured. Same FU as mortality used for the late-stage outcome. | CRC mortality in individuals randomised from 2004-2012;  FU until 31/12/2012 (median 4.5, range 0.0-8.3 years) (rate ratio)^4^ | 1.04 (0.84-1.28) | Only timepoint available |
| Bowel | Funen | % of CRC with Dukes' stage C, distant spread or no classification at diagnosis;  FU until August 1995^10^ | 0.84 (0.74-0.96) | Alternative timepoints are soon after randomisation, screening still ongoing. | CRC mortality (deaths from CRC or complications from treatment);  FU until Aug 1995 (mean 9.1 years) (rate ratio)^10^ | 0.82 (0.68-0.99) | Originally 3 screening rounds over 5-year period followed by 5 years of passive FU. |
| Bowel | Minnesota | % of CRC with Dukes’ stage D;  13 years of FU from entry -  annual FOBT vs. UC^17^ | 0.57 (0.38-0.84) | Only timepoint available | CRC mortality;  13 years of FU from entry (rate ratio) -  annual FOBT vs. UC^17^ | 0.67 (0.51-0.89) | Decision to publish results after review of most recent data on CRC mortality showed stopping boundary had been crossed in the annually screened group.  Through year 13, last complete year of FU. |
|  |  | % of CRC with Dukes’ D stage;  13 years of FU from entry -  biennial FOBT vs. UC^17^ | 0.68 (0.47-0.97) |  | 13 years of FU from entry (rate ratio)-  biennial FOBT vs. UC^17^ | 0.96 (0.74-1.23) |  |
| Bowel | Gothenburg | % of CRC that were Dukes' stage D at diagnosis; all cohorts (DOB 1918-1931);  FU until 31/12/2001^16^ | 0.99 (0.81-1.20) | Screening for all cohorts was not completed until May 1995 (Cohort 1). | CRC mortality; all cohorts;  FU until 31/12/2001 (mean 15.5 years) (rate ratio)^16^ | 0.84 (0.71-0.99) | Only timepoint available |
| Bowel | NORCCAP | % of CRC diagnosed at advanced stage in people aged 55-64 years;  FU until 31/12/2006^20^ | 0.88 (0.76-1.02) | Single screen up to Dec 2001. Timepoint provides sufficient FU after intervention and is earlier than mortality. | CRC mortality in people aged 55-64 years;  FU until 31/12/2015 (median 15 years) (hazard ratio)^22^ | 0.82 (0.66-1.02) | Planned end points were cumulative incidence and mortality of colorectal cancer after 5, 10, and 15 years. Original NORCCAP included 55-64 year olds. Chosen to fit UKFSST (15 years). |
| Bowel | NordICC | % of CRC with Dukes' stage C or D at diagnosis;  median 10-year FU^23^ | 0.97 (0.82-1.14) | Only timepoint available | CRC mortality;  10-year risk (median 10.0 years) (Kaplan-Meier estimate)^23^ | 0.90 (0.64-1.16) | Only timepoint available |
| Bowel | Nottingham | % of CRC with Dukes' stage C or D at diagnosis;  FU until 30/06/1995^25^ | 0.88 (0.80-0.97) | 3-6 screening rounds (biennial), so screening is ongoing for minimum up to 4 years since entry (Feb 1995). Mean FU of alternative timepoint is before screening has ended. | CRC mortality (verified cause of death);  FU until 30/06/1995 (median 7.8 years) (rate ratio)^25^ | 0.85 (0.74-0.98) | Hardcastle 1989^24^ reported that both groups would be followed up for at least 7 years. |
| Bowel | PLCO (bowel) | % of CRC diagnosed at stage III-IV;  13 years of FU or until 31/12/2009^33^ | 0.90 (0.81-1.00) | Only timepoint available | CRC mortality;  13 years of FU from entry or until 31/12/2009 (rate ratio)^33^ | 0.74 (0.63-0.87) | Main analysis timepoint: 13 years of FU or until 31/12/2009. |
| Bowel | SCORE | % of distal + proximal CRC diagnosed at stage III or IV;  FU until 31/12/2007^35^ | 0.90 (0.75-1.07) | Only timepoint available | CRC mortality;  FU until 31/12/2008 (5 centres) or 31/12/2007 (1 centre) (median 11.4 years) (rate ratio)^35^ | 0.78 (0.56-1.08) | A statistically significant reduction in mortality was expected to be detected after 11 years of FU. |
| Bowel | Telemark | % of CRC with Dukes’ stage C or D;  FU until 1993 (relative risk)^37^ | 1.00 (0.18-5.46) | 1-2 rounds  (single screen or 2-year interval); screening would have ended after 2 years of FU. Alternative timepoint provides sufficient FU and is earlier than mortality. | CRC mortality;  FU until 1995 (relative risk)^39^ | 0.33 (0.03-3.18) | No SAP or power calculation.  “Specified and all-cause mortality from 1983 up to and including 1995 were end points in this study…”  Chose 1995 to not include the exit screen. |
| Breast | CNBSS-1 | % of node-positive, invasive breast cancers diagnosed through 7 years^45^ | 1.27 (0.97-1.66) | Same timepoint as chosen mortality outcome.  Node-positivity was chosen over size to be consistent with what has been used in this analysis for other breast cancer trials. | Breast cancer mortality;  7 years of FU (relative risk)^45^ | 1.36 (0.84-2.21) | 5 years from entry; however, because insufficient deaths from breast cancer had occurred by 5 years to attain the planned power, FU was extended for 2 years, by which time there were enough breast cancer deaths to reach the planned power. |
| Breast | CNBSS-2 | % of node-positive, invasive breast cancers diagnosed through first 7 years^44^ | 0.94 (0.74-1.20) | As CNBSS-1 | Breast cancer mortality;  7 years of FU (relative risk)^44^ | 0.97 (0.62-1.52) | As CNBSS-1 |
| Breast | Edinburgh | % of breast cancers diagnosed at stage III-IV (Cohort 1);  7 years of FU from entry^56^ | 0.46 (0.35-0.61) | Only timepoint available. | Breast cancer mortality (Cohort 1);  7 years of FU from entry (mean 6.8 years) (rate ratio)^56^ | 0.84 (0.60-1.16) | 7 years of FU used for power calculations. |
| Breast | Gothenburg | % of node-positive, invasive breast cancers diagnosed during the screening phase (mean 6 years)^59^ | 0.89 (0.71-1.10) | Same cancers included as in mortality outcome | Breast cancer mortality in cases diagnosed in screening period (blinded review of cause of death); FU until 31/12/1996 (up to 14 years) (relative risk)^58^ | 0.78 (0.57-1.06) | Primary outcome: Deaths of cases diagnosed in screening period up to 31/12/1996. |
| Breast | HIP NY | % of node-positive, invasive breast cancers diagnosed during first 5 years of from entry^68^ | 0.80 (0.65-0.99) | Screening ended at 3 years of FU from entry (4 rounds, annually; June 1970). Screening still ongoing for alternative timepoint. | Breast cancer mortality in 10 years from entry (rate ratio)^63^ | 0.78 (0.63-0.96) | Minimum of 5 years FU after the screening program has been completed. Everything else sounds post hoc. |
| Breast | Malmo | % of invasive breast cancers diagnosed at stage II-IV;  FU until 31/12/1986^78^ | 0.67 (0.58-0.76) | Intervention is ongoing when mortality is measured. Same FU as mortality used for the late-stage outcome. | Breast cancer mortality;  FU until 31/12/1986 (mean 8.8 years) (rate ratio)^78^ | 0.96 (0.68-1.35) | Predetermined end of trial was 31/12/1986; no interim analyses have been performed. |
| Breast | Mumbai | % of breast cancers diagnosed at stage III-IV;  FU until May 2018 (March 2019)^82^ | 0.83 (0.72-0.96) | Only timepoint available | Breast cancer mortality;  FU until May 2018 (March 2019) (median 18 years) (rate ratio)^82^ | 0.85 (0.70-1.02) | Study was planned for a period of 16 years to include 4 screening rounds followed by 4 monitoring rounds. |
| Breast | Russia/WHO | % of node-positive breast cancers diagnosed until 1994^85^ | 0.97 (0.86-1.09) | First intervention received between 1985-1989 and a min of 2 sessions (up to 4) were provided in 3-year intervals. Participants that received their first session in 1989 are still expected to receive their 2^nd^ session in 1992, so after our alternative timepoint. | Breast cancer mortality in cases diagnosed until 31/12/1994;  FU until 31/12/1997 (relative risk)^85^ | 1.07 (0.86-1.34) | 5 years and 10 years FU.  Registration of new breast cancer cases will be continued up to 1994, with follow-up until 1999. Only timepoint available (at least in English language). |
| Breast | Shanghai | % of node-positive (N1-N3) breast cancers diagnosed until 31/12/2000^87^ | 0.95 (0.84-1.08) | Only timepoint available | Breast cancer mortality;  FU until 31/12/2000 (mean 10 years) (rate ratio)^87^ | 1.04 (0.82-1.33) | Total trial duration of 10 years. |
| Breast | Stockholm | % of invasive breast cancers diagnosed at stage II-IV;  FU until 1986^92^ | 0.93 (0.78-1.12) | Intervention ended in 1985, followed by exit screen 1985-1986. Alternative timepoints do not have sufficient FU after the intervention has ended, therefore selecting the timepoint that includes the exit screen. | Breast cancer mortality in cases diagnosed in screening period (including exit screen); FU until 31/12/1994 (rate ratio)^92^ | 0.74 (0.50-1.10) | SAP or protocol not reported.  FU until 31/12/1994 seems to be the main analysis time point for mortality. |
| Breast | Trivandrum | % of breast cancers diagnosed at stage III-IV;  FU until 31/12/2019^93^ | 0.90 (0.74-1.10) | Screening did not end until 2017. Alternative timepoint too early. | Breast cancer mortality;  FU until 31/12/2019 (mean 12 years) (rate ratio)^93^ | 1.03 (0.75-1.41) | Only timepoint available |
| Breast | Two-County | % of invasive breast cancers diagnosed at stage II+ in women aged 40-74 years;  FU until 31/12/1984^100^ | 0.61 (0.55-0.67) | Intervention is ongoing when mortality is measured. Same FU as mortality used for the late-stage outcome. | Breast cancer mortality in women aged 40-74 years; FU until 31/12/1984 (mean 6 years) (rate ratio)^100^ | 0.72 (0.54-0.98) | First mortality outcome reported. |
| Breast | UK Age | % of node-positive, invasive breast cancers diagnosed during the intervention period (before first NHSBSP screen)^110^ | 0.89 (0.79-1.00) | Screening did not end until 2006 / 7. Alternative timepoint too early. | Breast cancer mortality in cases diagnosed during the intervention period (before first NHSBSP screen);  FU until 31/12/2011 (median 17.7 years) (rate ratio)^113^ | 0.88 (0.74-1.04) | Primary endpoint was mortality from breast cancers diagnosed during the intervention phase of the trial. Power calculations revised to 14 years of FU; originally 10 years of FU. |
| Cervical | Mumbai | % of invasive cervical cancer diagnosed at  stage ≥IIB after 12 years (31/12/2011)^115^ | 0.86 (0.72-1.04) | Only timepoint available | Cervical cancer mortality;  FU until 31/12/2011 (mean 8.0 years; up to 12 years) (rate ratio)^115^ | 0.69 (0.50-0.94) | 16 years since enrolment.  However, the observed mortality rate in the control group was higher than assumed, which enabled us to detect a statistically significant reduction in mortality earlier than planned, at the end of 12 years. |
| Cervical | Osmanabad | % of cervical cancers diagnosed at stage II+;  FU until 31/12/2007 - VIA vs. UC^117^ | 0.79 (0.65-0.95) | Screening has just ended in Nov 2003. Not sufficient time after end of intervention for alternative timepoint. | Cervical cancer mortality;  FU until 31/12/2007 (mean 8 years) (rate ratio) - VIA vs. UC^117^ | 0.81 (0.57-1.16) | Within 15 years of enrolment. Only timepoint available. |
|  |  | FU until 31/12/2007 - cytology vs. UC^117^ | 0.55 (0.43-0.69) |  | FU until 31/12/2007 (mean 8 years) (rate ratio) - cytology vs. UC^117^ | 0.83 (0.58-1.20) |  |
|  |  | FU until 31/12/2007 - HPV vs. UC^117^ | 0.44 (0.33-0.59) |  | FU until 31/12/2007 (mean 8 years) (rate ratio) - HPV vs. UC^117^ | 0.49 (0.32-0.74) |  |
| Cervical | Tamil Nadu | % of invasive cervical cancer diagnosed at stage II+;  FU until 31/12/2006^119^ | 1.01 (0.86-1.20) | Screening has just ended in 30/04/2003. Not sufficient time after end of intervention for alternative timepoint. | Cervical cancer mortality;  FU until 31/12/2006 (up to 7 years) (rate ratio)^119^ | 0.59 (0.44-0.79) | The observed mortality rate in the control population during the study was much higher than assumed, which enabled us to observe a significant effect on cancer rate and mortality 7 years from the beginning of the study, rather than waiting for 10 years, as predicted in the power calculations.  Only timepoint available. |
| Liver | Qidong | % of liver cancers diagnosed at stage III (Chinese HCC staging system);  FU until 31/12/1995^120^ | 0.48 (0.35-0.67) | Only timepoint available | Liver cancer mortality;  FU until 31/12/1995 (mean 62-63 months) (rate ratio)^120^ | 1.02 (0.81-1.29) | Only timepoint available |
| Liver | Shanghai | % of liver cancer diagnosed at stage III (Chinese HCC staging system); FU until 31 December 1997^121^ | 0.41 (0.27-0.61) | Only timepoint available | Liver cancer mortality;  FU until 31/12/1997 (mean 4.1-4.3 years) (rate ratio)^121^ | 0.63 (0.41-0.98) | Only timepoint available |
| Lung | Czech Study | % of lung cancers diagnosed at stage III (clinical-diagnostic staging system with occult, I, II and III stages);  0-6 years after study entry^123^ | 0.87 (0.67-1.15) | Screening was completed at 6y from entry. Screening is ongoing at the alternative timepoints (and just ended for selected). | Lung cancer mortality;  15 years of FU from entry (relative risk)^125^ | 1.14 (0.96-1.36) | No reference to SAP or power calculations.  Chose timepoint consistent with PLCO.  Used all cancers as cancers diagnosed in first 3 or 6 years seem not their primary analysis. |
| Lung | DANTE | % of lung cancers diagnosed at stage II-IV;  FU until 15 May 2013^128^ | 0.69 (0.54-0.89) | Last recruited was in Feb 2006 and there were 5 rounds of screening (annually). Screening is still ongoing for some participants for the alternative timepoint. | Lung cancer mortality;  FU until 15/05/2013 (median 8.35 years) (rate ratio)^128^ | 1.00 (0.69-1.44) | Power calculation of 5 years of FU.  The only other available timepoint is until 25/01/2008 (median 35.7 and 31.5 months). |
| Lung | DLCST | % of lung cancers diagnosed at stage III-IV;  FU until 7 April 2015^130^ | 0.59 (0.46-0.77) | Intervention ended in March 2010, not sufficient FU after the end of intervention for alternative timepoint. | Lung cancer mortality;  FU until 07/04/2015 (median 9.8 years) (rate ratio)^130^ | 1.03 (0.66-1.61) | FU time from power calculations was 10 years from randomisation. |
| Lung | ITALUNG | % of lung cancers diagnosed at stage III-IV;  FU until 31 December 2013^131^ | 0.81 (0.60-1.11) | Alternative timepoint after mortality. | Lung cancer mortality;  FU until 31/12/2014 (median 9.3 years) (rate ratio)^131^ | 0.70 (0.47-1.03) | SAP: 10 years after randomisation |
| Lung | Johns Hopkins | % of lung cancers diagnosed at stage II-IV;  FU for up to 9 years from entry^134^ | 0.91 (0.77-1.06) | Alternative timepoint only provides information on prevalence screen. | Lung cancer mortality;  FU for up to 9 years from entry (mean 7.2 years) (rate ratio)^134^ | 0.83 (0.67-1.04) | No SAP or power calculation;  chose longest FU time available; otherwise, the late-stage outcome would have been measured after mortality, now it is at the same timepoint. |
| Lung | LSS | % of lung cancers diagnosed at stage III-IV;  1 year FU from entry^139^ | 0.89 (0.48-1.64) | Only timepoint available | Lung cancer mortality;  FU until 31/12/2005 (median 5.2 years) (rate ratio)^137^ | 1.24 (0.74-2.08) | Only timepoint available |
| Lung | LUSI | % of lung cancers diagnosed at stage II-IV;  FU for 7 years after randomisation^141^ | 0.39 (0.29-0.54) | Halfway point between 4y since entry (last screen) and 10y since entry (median mortality FU). | Lung cancer mortality;  FU until 30/04/2018 (within 11 years of randomisation; median 8.9 years) (relative risk)^141^ | 0.72 (0.45-1.16) | From trial registration: Primary outcome measure is mortality from lung cancer at 5 and 10 years. |
| Lung | Mayo Lung Project | % of lung cancers diagnosed at stage III-IV;  FU until 01/07/1983 (6-year screening period plus 1-5.5 years post-screen)^147^ | 0.76 (0.64-0.90) | Intervention did not end until July 1982. All alternative timepoints are earlier than that. | Lung cancer mortality;  FU until 01/07/1983 (6-year screening period plus 1-5.5 years post-screen) (relative risk)^147^ | 1.06 (0.82-1.36) | Mayo Lung Project ended on 01 July 1983. |
| Lung | Memorial Sloan Kettering | % of lung cancers diagnosed at stage II-IV;  FU for up to 9 years from entry^134^ | 0.98 (0.82-1.18) | Alternative timepoint does not have sufficient FU after intervention has ended (Oct 1982). | Lung cancer mortality;  FU for up to 9 years from entry (mean 7.2 years) (rate ratio)^134^ | 0.95 (0.73-1.25) | No SAP or power calculation available.  Chose timepoint with longest follow-up; same follow-up time as the late-stage outcome. |
| Lung | MILD | % of lung cancers diagnosed at stage II-IV;  FU until June 2018^157^ | 0.64 (0.50-0.81) | Screening period is ~6 years since entry. Alternative timepoint does not have sufficient FU after intervention has ended (for most participants <6y) | Lung cancer mortality;  FU until June 2018 (mean 9.7 years) (rate ratio) LDCT (combined) vs. UC ^157^ | 0.70 (0.45-1.09) | 10 years of FU.  Chose longest follow-up available; same analysis date as the chosen late-stage outcome. |
| Lung | NELSON | % of lung cancers diagnosed at stage III-IV in men;  FU for 10 years or 31/12/2015^159^ | 0.63 (0.55-0.72) | Only timepoint available. | Lung cancer mortality in men;  FU for 10 years from entry or 31/12/2015 (mean 9.5 years) (rate ratio)^159^ | 0.76 (0.61-0.94) | 10 years of FU in men. |
| Lung | NLST | % of lung cancers diagnosed at stage III-IV;  FU until 31/12/2009 (median 6.5 years)^160^ | 0.70 (0.64-0.77) | Not sufficient FU after end of screening for alternative timepoints. | Lung cancer mortality;  FU until 31/12/2009 (median 6.5 years) (rate ratio)^162^ | 0.84 (0.75-0.95) | SAP: 15/01/2009 for LC mortality (time lags associated with the end point verification process). LC mortality results subsequently reported using all events through 31/12/2009 (same cut-off date as for all-cause mortality). |
| Lung | PLCO (lung) | % non-small cell lung cancers diagnosed at stage III-IV;  FU for 13 years or 31/12/2009^163^ | 0.92 (0.87-0.98) | Only timepoint available | Lung cancer mortality;  FU for 13 years or 31/12/2009 (median 11.9 years) (rate ratio)^163^ | 0.99 (0.91-1.07) | 13 years or 31/12/2009, whichever came first. |
| Lung | UKLS | % of lung cancers diagnosed stage III-IV;  FU for 4 years since study entry^164^ | 0.33 (0.19-0.57) | Single screen soon after randomisation (Oct 2011-Feb 2013). Halfway timepoint from randomisation to mortality timepoint (median 7.3y). | Lung cancer mortality;  FU until 29/02/2020 (median 7.3 years) (rate ratio)^164^ | 0.65 (0.41-1.02) | SAP: For the initial FU report, the FU period is up to 29 February 2020 for mortality and up to 31 December 2019 for incidence of lung cancer. |
| Naso-pharyngeal | China NPC | % of NPC diagnosed at stage III-IV;  FU until 31/12/2014^165^ | 0.70 (0.58-0.85) | Only timepoint available | NPC mortality;  FU until 31/12/2014 (median 6.0 years) (rate ratio)^165^ | 0.81 (0.45-1.45) | Only timepoint available |
| Oral | Trivandrum | % of oral cancers diagnosed at stage III-IV;  FU until 31/12/2004^168^ | 0.76 (0.64-0.91) | Intervention is ongoing (last: 2006-2009) when mortality is measured. Same FU as mortality. | Oral cancer mortality;  FU until 31/12/2004 (mean 4.4-4.9 years; up to 9 years) (rate ratio)^168^ | 0.79 (0.51-1.22) | 12 year of enrolment (10 years of FU?).  First time they have reported mortality. Before exit screen (which was performed 2006-2009). |
| Ovarian | PLCO (ovarian) | % of ovarian cancers (including primary peritoneal and fallopian tube cancer) diagnosed at stage III-IV;  FU for 13 years from entry or until 28/02/2010^170^ | 0.99 (0.89-1.10) | Not sufficient FU after end of screening for alternative timepoint. | Ovarian cancer (including primary peritoneal and fallopian tube cancer) mortality;  FU for 13 years from entry or until 28/02/2010 (median 12.4 years) (rate ratio)^170^ | 1.18 (0.91-1.54) | Up to 13 years from randomisation or 28 February 2010, whichever came first. |
| Ovarian | UK Pilot | % of ovarian cancers diagnosed at stage III-IV;  FU until December 1997^173^ | 0.76 (0.53-1.10) | Only timepoint available | Ovarian cancer mortality;  FU until January 1998 (up to 8 years) (relative risk)^173^ | 0.50 (0.19-1.28) | Only timepoint available |
| Ovarian | UKCTOCS | % of primary ovarian cancer diagnosed at stage III-IV; FU until 31/12/2014 (mean 10.8 years) - MMS vs. UC^174^ | 0.77 (0.69-0.87) | Alternative timepoint after mortality. | Ovarian cancer mortality;  FU until 31/12/2014 (median 11.1 years) (rate ratio) - MMS vs. UC^174^ | 0.85 (0.70-1.03) | Original protocol:  7 years after randomisation.  Extended FU until 31/12/2014; FU extended again to 30/06/2020.  Same analysis date as for the late-stage outcome. |
|  |  | % of primary ovarian cancer diagnosed at stage III-IV; FU until 31/12/2014 (mean 10.8 years) - TVS vs. UC^174^ | 0.91 (0.82-1.10) |  | Ovarian cancer mortality;  FU until 31/12/2014 (median 11.1 years) (rate ratio) - TVS vs. UC^174^ | 0.89 (0.73-1.07) |  |
| Prostate | CAP | % of prostate cancer with Gleason score 8-10 at diagnosis;  <6 years of FU^176^ | 0.63 (0.57-0.70) | Halfway point between 18 months (screening period) to 10 years (median mortality) | Definite, probable, or intervention-related prostate cancer mortality;  FU until 31/03/2016 (median 10 years) (rate ratio)^176^ | 0.96 (0.86-1.08) | Defined in trial protocol/SAP as primary outcome and used for power calculation. |
| Prostate | ERSPC | % of prostate cancer with Gleason score 8-10 in men aged 55-69 years; whole trial excluding France and Portugal;  FU until 31/12/2008 (median 11.0 years)^195^ | 0.52 (0.47-0.57) | Intervention is ongoing when mortality is measured. Same FU as mortality used for the late-stage outcome. | Prostate cancer mortality in men aged 55-69 years; whole trial excluding France and Portugal;  FU until 31/12/2008 (mean 10.5 years) (rate ratio)^195^ | 0.79 (0.68-0.91) | FU through 2008 main analysis time point. |
| Prostate | ERSPC Pilot 1 | % of prostate cancer with Gleason score ≥3+4;  FU for median 19 years from entry (IQR 12-24)^200^ | 0.66 (0.43-1.01) | Only timepoint available | Prostate cancer mortality;  FU for median 19 years from entry (IQR 12-24) (relative risk)^200^ | 0.48 (0.17-1.36) | Only timepoint available |
| Prostate | Norrkoping | % of prostate cancers diagnosed at advanced stage (T3-T4, N1 or MX/M1); cancers diagnosed up to 31 December 1999^202^ | 0.59 (0.46-0.76) | Only timepoint available | Prostate cancer mortality in cancers diagnosed up to 31/12/1999;  FU until 31/12/2008 (20 years) (relative risk)^202^ | 1.16 (0.78-1.73) | 20-year FU used in power calculations. |
| Prostate | PLCO (prostate) | % of prostate cancers with Gleason score 8-10; FU through 10 years^203^ | 0.73 (0.63-0.85) | Intervention period ended at 5 years since randomisation. The alternative timepoint provides sufficient FU after the end of the intervention and is before the mortality endpoint. | Prostate cancer mortality;  FU for 13 years from entry or until 31/12/2009 (mean 11.1 years) (rate ratio)^204^ | 1.09 (0.87-1.36) | Reported as the planned follow-up in the “Design of the PLCO trial” publication. |
| Multiple* | D’Aquapendente | % of cancers diagnosed at stage IV;  FU for 24 months from entry^210^ | 0.62 (0.18-2.16) | Only timepoint available | Cancer-specific mortality;  24 months FU from entry (relative risk)^210^ | 0.49 (0.09-2.64) | Only timepoint available |
| Multiple* | MVTEP | % of cancers diagnosed at “advanced” stage;  24 months FU from entry ^211^ | 0.77 (0.33-1.79) | Only timepoint available | Cancer-specific mortality;  24 months FU (relative risk)^211^ | 0.40 (0.08-2.04) | Only timepoint available |
| Multiple* | SOMIT | % of cancers with N1 and/or M1;24 months FU from entry (relative risk)^212^ | 0.45 (0.21-0.96) | Only timepoint available | Cancer-specific mortality;  24 months FU from entry (relative risk)^212^ | 0.52 (0.10-2.75) | Only timepoint available |

**Abbreviations.** CI, confidence interval; CRC, colorectal cancer; Dec, December; DOB, date of birth; FOBT, faecal occult blood test; Feb, February; FU, follow-up; HCC, hepatocellular cancer; HPV, human papillomavirus; IQR, interquartile range; LC, lung cancer; LDCT, low-dose computed tomography; M1, distant metastasis present; MMS, multimodal screening; MX, metastasis cannot be measured; N1, cancer has spread to nearby lymph nodes; N1, N2, N3, number of nearby lymph nodes that have cancer; NHSBSP, NHS Breast Screening Programme; NPC, nasopharyngeal cancer; Oct, October; SAP, statistical analysis plan; T3, tumour size or area: cancer has broken through the capsule (covering) of the prostate gland; T4, tumour size or area: cancer has spread into other body organs nearby; TVS, transvaginal ultrasound; UC, usual care; VIA, visual inspection with acetic acid; WHO, Word Health Organization.

**Trial name abbreviations.** See Table S12 legend.

* Different target cancers screened for in each trial.

**REFERENCE LIST**

1. Chiu SY-H, Malila N, Yen AM-F, et al. Predicting the effectiveness of the Finnish population-based colorectal cancer screening programme. *Journal of medical screening* 2017;24(4):182-88. doi: <https://dx.doi.org/10.1177/0969141316684524>

2. Koskenvuo L, Malila N, Pitkaniemi J, et al. Sex differences in faecal occult blood test screening for colorectal cancer. *The British journal of surgery* 2019;106(4):436-47. doi: <https://dx.doi.org/10.1002/bjs.11011>

3. Paimela H, Malila N, Palva T, et al. Early detection of colorectal cancer with faecal occult blood test screening. *The British journal of surgery* 2010;97(10):1567-71. doi: <https://dx.doi.org/10.1002/bjs.7150>

4. Pitkaniemi J, Seppa K, Hakama M, et al. Effectiveness of screening for colorectal cancer with a faecal occult-blood test, in Finland. *BMJ open gastroenterology* 2015;2(1):e000034. doi: <https://dx.doi.org/10.1136/bmjgast-2015-000034>

5. Bech K, Kronborg O, Fenger C. Adenomas and hyperplastic polyps in screening studies. *World journal of surgery* 1991;15(1):7-13.

6. Jorgensen OD, Kronborg O, Fenger C. A randomised study of screening for colorectal cancer using faecal occult blood testing: results after 13 years and seven biennial screening rounds. *Gut* 2002;50(1):29-32.

7. Kronborg O, Fenger C, Sondergaard O. Initial mass screening for colorectal cancer with fecal occult blood test. A prospective randomized study at Funen in Denmark. *Scandinavian Journal of Gastroenterology* 1987;22(6):677-86.

8. Kronborg O, Fenger C, Olsen J, et al. Repeated screening for colorectal cancer with fecal occult blood test. A prospective randomized study at Funen, Denmark. *Scandinavian journal of gastroenterology* 1989;24(5):599-606.

9. Kronborg O, Fenger C, Worm J, et al. Causes of death during the first 5 years of a randomized trial of mass screening for colorectal cancer with fecal occult blood test. *Scandinavian journal of gastroenterology* 1992;27(1):47-52.

10. Kronborg O, Fenger C, Olsen J, et al. Randomised study of screening for colorectal cancer with faecal-occult-blood test. *Lancet (London, England)* 1996;348(9040):1467-71.

11. Kronborg O, Jorgensen OD, Fenger C, et al. Randomized study of biennial screening with a faecal occult blood test: results after nine screening rounds. *Scandinavian journal of gastroenterology* 2004;39(9):846-51.

12. Shaukat A, Kaalby L, Baatrup G, et al. Effects of Screening Compliance on Long-term Reductions in All-Cause and Colorectal Cancer Mortality. *Clinical Gastroenterology and Hepatology* 2021;19(5):967-75.e2. doi: <https://dx.doi.org/10.1016/j.cgh.2020.06.019>

13. Kewenter J, Bjork S, Haglind E, et al. Screening and rescreening for colorectal cancer. A controlled trial of fecal occult blood testing in 27,700 subjects. *Cancer* 1988;62(3):645-51.

14. Kewenter J, Brevinge H, Engaras B, et al. Results of screening, rescreening, and follow-up in a prospective randomized study for detection of colorectal cancer by fecal occult blood testing. *Scandinavian Journal of Gastroenterology* 1994;29(5):468-73.

15. Kewenter J, Brevinge H, Engaras B, et al. Follow-up after screening for colorectal neoplasms with fecal occult blood testing in a controlled trial. *Diseases of the Colon and Rectum* 1994;37(2):115-19.

16. Lindholm E, Brevinge H, Haglind E. Survival benefit in a randomized clinical trial of faecal occult blood screening for colorectal cancer. *The British journal of surgery* 2008;95(8):1029-36. doi: <https://dx.doi.org/10.1002/bjs.6136>

17. Mandel JS, Bond JH, Church TR, et al. Reducing mortality from colorectal cancer by screening for fecal occult blood. Minnesota Colon Cancer Control Study. *The New England journal of medicine* 1993;328(19):1365-71.

18. Mandel JS, Church TR, Ederer F, et al. Colorectal cancer mortality: Effectiveness of biennial screening for fecal occult blood. *Journal of the National Cancer Institute* 1999;91(5):434-37. doi: <http://dx.doi.org/10.1093/jnci/91.5.434>

19. Shaukat A, Mongin SJ, Geisser MS, et al. Long-term mortality after screening for colorectal cancer. *The New England journal of medicine* 2013;369(12):1106-14. doi: <https://dx.doi.org/10.1056/NEJMoa1300720>

20. Hoff G, Grotmol T, Skovlund E, et al. Risk of colorectal cancer seven years after flexible sigmoidoscopy screening: randomised controlled trial. *BMJ (Clinical research ed)* 2009;338:b1846. doi: <https://dx.doi.org/10.1136/bmj.b1846>

21. Holme O, Loberg M, Kalager M, et al. Effect of flexible sigmoidoscopy screening on colorectal cancer incidence and mortality: a randomized clinical trial. *JAMA* 2014;312(6):606-15. doi: <https://dx.doi.org/10.1001/jama.2014.8266>

22. Holme O, Loberg M, Kalager M, et al. Long-Term Effectiveness of Sigmoidoscopy Screening on Colorectal Cancer Incidence and Mortality in Women and Men: A Randomized Trial. *Annals of internal medicine* 2018;168(11):775-82. doi: <https://dx.doi.org/10.7326/M17-1441>

23. Bretthauer M, Loberg M, Wieszczy P, et al. Effect of Colonoscopy Screening on Risks of Colorectal Cancer and Related Death. *New England Journal of Medicine* 2022;387(17):1547-56. doi: <https://dx.doi.org/10.1056/NEJMoa2208375>

24. Hardcastle JD, Thomas WM, Chamberlain J, et al. Randomised, controlled trial of faecal occult blood screening for colorectal cancer. Results for first 107,349 subjects. *Lancet (London, England)* 1989;1(8648):1160-4.

25. Hardcastle JD, Chamberlain JO, Robinson MH, et al. Randomised controlled trial of faecal-occult-blood screening for colorectal cancer. *Lancet (London, England)* 1996;348(9040):1472-7.

26. Mapp TJ, Hardcastle JD, Moss SM, et al. Survival of patients with colorectal cancer diagnosed in a randomized controlled trial of faecal occult blood screening. *The British journal of surgery* 1999;86(10):1286-91.

27. Scholefield JH, Moss S, Sufi F, et al. Effect of faecal occult blood screening on mortality from colorectal cancer: results from a randomised controlled trial. *Gut* 2002;50(6):840-4.

28. Scholefield JH, Moss SM, Mangham CM, et al. Nottingham trial of faecal occult blood testing for colorectal cancer: A 20-year follow-up. *Gut* 2012;61(7):1036-40. doi: <https://dx.doi.org/10.1136/gutjnl-2011-300774>

29. Whynes DK, Mangham CM, Balfour TW, et al. Analysis of deaths occurring within the Nottingham trial of faecal occult blood screening for colorectal cancer. *Gut* 2010;59(8):1088-93. doi: <https://dx.doi.org/10.1136/gut.2009.192971>

30. Miller EA, Pinsky PF, Schoen RE, et al. Effect of flexible sigmoidoscopy screening on colorectal cancer incidence and mortality: long-term follow-up of the randomised US PLCO cancer screening trial. *The lancet Gastroenterology & hepatology* 2019;4(2):101-10. doi: <https://dx.doi.org/10.1016/S2468-1253(18)30358-3>

31. Pinsky PF, Miller EA, Zhu CS, et al. Overall mortality in men and women in the randomized Prostate, Lung, Colorectal, and Ovarian Cancer Screening Trial. *Journal of medical screening* 2019;26(3):127-34. doi: <https://dx.doi.org/10.1177/0969141319839097>

32. Prorok PC, Wright P, Riley TR, et al. Overall and Multiphasic Findings of the Prostate, Lung, Colorectal and Ovarian (PLCO) Randomized Cancer Screening Trial. *Reviews on recent clinical trials* 2018;13(4):257-73. doi: <https://dx.doi.org/10.2174/1574887113666180409153059>

33. Schoen RE, Pinsky PF, Weissfeld JL, et al. Colorectal-cancer incidence and mortality with screening flexible sigmoidoscopy. *The New England journal of medicine* 2012;366(25):2345-57. doi: <https://dx.doi.org/10.1056/NEJMoa1114635>

34. Juul FE, Cross AJ, Schoen RE, et al. 15-Year Benefits of Sigmoidoscopy Screening on Colorectal Cancer Incidence and Mortality. *Annals of Internal Medicine* 2022;175(11):1525-33. doi: <https://dx.doi.org/10.7326/M22-0835>

35. Segnan N, Armaroli P, Bonelli L, et al. Once-only sigmoidoscopy in colorectal cancer screening: Follow-up findings of the italian randomized controlled trial - SCORE. *Journal of the National Cancer Institute* 2011;103(17):1310-22. doi: <https://dx.doi.org/10.1093/jnci/djr284>

36. Senore C, Riggi E, Armaroli P, et al. Long-Term Follow-up of the Italian Flexible Sigmoidoscopy Screening Trial. *Annals of internal medicine* 2022;175(1):36-45. doi: <https://dx.doi.org/10.7326/M21-0977>

37. Hoff G, Sauar J, Vatn MH, et al. Polypectomy of adenomas in the prevention of colorectal cancer: 10 years' follow-up of the Telemark Polyp Study I. A prospective, controlled population study. *Scandinavian journal of gastroenterology* 1996;31(10):1006-10.

38. Hoff G, Thiis-Evensen E, Grotmol T, et al. Do undesirable effects of screening affect all-cause mortality in flexible sigmoidoscopy programmes? Experience from the Telemark Polyp Study 1983-1996. *European journal of cancer prevention : the official journal of the European Cancer Prevention Organisation (ECP)* 2001;10(2):131-7.

39. Thiis-Evensen E, Hoff GS, Sauar J, et al. Population-based surveillance by colonoscopy: effect on the incidence of colorectal cancer. Telemark Polyp Study I. *Scandinavian journal of gastroenterology* 1999;34(4):414-20.

40. Thiis-Evensen E, Kalager M, Bretthauer M, et al. Long-term effectiveness of endoscopic screening on incidence and mortality of colorectal cancer: A randomized trial. *United European Gastroenterology Journal* 2013;1(3):162-68. doi: <https://dx.doi.org/10.1177/2050640613483290>

41. Atkin WS, Edwards R, Kralj-Hans I, et al. Once-only flexible sigmoidoscopy screening in prevention of colorectal cancer: a multicentre randomised controlled trial. *Lancet (London, England)* 2010;375(9726):1624-33. doi: <https://dx.doi.org/10.1016/S0140-6736(10)60551-X>

42. Atkin W, Wooldrage K, Parkin DM, et al. Long term effects of once-only flexible sigmoidoscopy screening after 17 years of follow-up: the UK Flexible Sigmoidoscopy Screening randomised controlled trial. *Lancet (London, England)* 2017;389(10076):1299-311. doi: <https://dx.doi.org/10.1016/S0140-6736(17)30396-3>

43. Cross AJ, Robbins EC, Saunders BP, et al. Higher Adenoma Detection Rates at Screening Associated With Lower Long-Term Colorectal Cancer Incidence and Mortality. *Clinical gastroenterology and hepatology : the official clinical practice journal of the American Gastroenterological Association* 2022;20(2):e148-e67. doi: <https://dx.doi.org/10.1016/j.cgh.2020.09.020>

44. Miller AB, Baines CJ, To T, et al. Canadian National Breast Screening Study: 2. Breast cancer detection and death rates among women aged 50 to 59 years. *CMAJ : Canadian Medical Association journal = journal de l'Association medicale canadienne* 1992;147(10):1477-88.

45. Miller AB, Baines CJ, To T, et al. Canadian National Breast Screening Study: 1. Breast cancer detection and death rates among women aged 40 to 49 years. *CMAJ : Canadian Medical Association journal = journal de l'Association medicale canadienne* 1992;147(10):1459-76.

46. Miller AB, To T, Baines CJ, et al. The Canadian National Breast Screening Study: update on breast cancer mortality. *Journal of the National Cancer Institute Monographs* 1997(22):37-41.

47. Miller AB, To T, Baines CJ, et al. Canadian National Breast Screening Study-2: 13-year results of a randomized trial in women aged 50-59 years. *Journal of the National Cancer Institute* 2000;92(18):1490-9.

48. Miller AB, To T, Baines CJ, et al. The Canadian National Breast Screening Study-1: breast cancer mortality after 11 to 16 years of follow-up. A randomized screening trial of mammography in women age 40 to 49 years. *Annals of internal medicine* 2002;137(5 Part 1):305-12.

49. Miller AB, Wall C, Baines CJ, et al. Twenty five year follow-up for breast cancer incidence and mortality of the Canadian National Breast Screening Study: randomised screening trial. *BMJ (Clinical research ed)* 2014;348:g366. doi: <https://dx.doi.org/10.1136/bmj.g366>

50. Narod SA, Wall C, Baines C, et al. Impact of screening mammography on mortality from breast cancer before age 60 in women 40 to 49 years of age. *Current Oncology* 2014;21(5):217-21. doi: <https://dx.doi.org/10.3747/co.21.2067>

51. Shen Y, Yang Y, Inoue LYT, et al. Role of detection method in predicting breast cancer survival: analysis of randomized screening trials. *Journal of the National Cancer Institute* 2005;97(16):1195-203.

52. Alexander FE, Anderson TJ, Brown HK, et al. The Edinburgh randomised trial of breast cancer screening: results after 10 years of follow-up. *British journal of cancer* 1994;70(3):542-8.

53. Alexander FE, Anderson TJ, Brown HK, et al. 14 years of follow-up from the Edinburgh randomised trial of breast-cancer screening. *Lancet (London, England)* 1999;353(9168):1903-8.

54. Anderson TJ, Lamb J, Donnan P, et al. Comparative pathology of breast cancer in a randomised trial of screening. *Br J Cancer* 1991;64(1):108-13. doi: 10.1038/bjc.1991.251

55. Anderson TJ, Alexander FE, Lamb J, et al. Pathology characteristics that optimize outcome prediction of a breast screening trial. *British journal of cancer* 2000;83(4):487-92.

56. Roberts MM, Alexander FE, Anderson TJ, et al. Edinburgh trial of screening for breast cancer: mortality at seven years. *Lancet (London, England)* 1990;335(8684):241-6.

57. Bjurstam N, Bjorneld L, Duffy SW, et al. The Gothenburg breast screening trial: first results on mortality, incidence, and mode of detection for women ages 39-49 years at randomization. *Cancer* 1997;80(11):2091-9.

58. Bjurstam N, Bjorneld L, Warwick J, et al. The Gothenburg Breast Screening Trial. *Cancer* 2003;97(10):2387-96.

59. Bjurstam NG, Bjorneld LM, Duffy SW. Updated results of the Gothenburg Trial of Mammographic Screening. *Cancer* 2016;122(12):1832-5. doi: <https://dx.doi.org/10.1002/cncr.29975>

60. Larsson LG, Andersson I, Bjurstam N, et al. Updated overview of the Swedish Randomized Trials on Breast Cancer Screening with Mammography: age group 40-49 at randomization. *Journal of the National Cancer Institute Monographs* 1997(22):57-61.

61. Nystrom L, Rutqvist LE, Wall S, et al. Breast cancer screening with mammography: overview of Swedish randomised trials. *Lancet (London, England)* 1993;341(8851):973-8.

62. Nystrom L, Andersson I, Bjurstam N, et al. Long-term effects of mammography screening: Updated overview of the Swedish randomised trials. *Lancet* 2002;359(9310):909-19. doi: <https://dx.doi.org/10.1016/S0140-6736%2802%2908020-0>

63. Aron JL, Prorok PC. An analysis of the mortality effect in a breast cancer screening study. *International journal of epidemiology* 1986;15(1):36-43.

64. Habbema JD, van Oortmarssen GJ, van Putten DJ, et al. Age-specific reduction in breast cancer mortality by screening: an analysis of the results of the Health Insurance Plan of Greater New York study. *Journal of the National Cancer Institute* 1986;77(2):317-20.

65. Shapiro S, Strax P, Venet L. Evaluation of periodic breast cancer screening with mammography. Methodology and early observations. *JAMA* 1966;195(9):731-8.

66. Shapiro S. Evaluation of two contrasting types of screening programs. *Preventive Medicine* 1973;2(2):266-77. doi: <https://dx.doi.org/10.1016/0091-7435%2873%2990070-4>

67. Shapiro S, Goldberg JD, Hutchison GB. Lead time in breast cancer detection and implications for periodicity of screening. *American Journal of Epidemiology* 1974;100(5):357-66.

68. Shapiro S. Evidence on screening for breast cancer from a randomized trial. *Cancer* 1977;39(6 ,sup):2772-82.

69. Shapiro S. Efficacy of breast cancer screening. *UICC Technical Report Series* 1978;VOL 40:133-57.

70. Shapiro S, Venet W, Strax P, et al. Ten- to fourteen-year effect of screening on breast cancer mortality. *Journal of the National Cancer Institute* 1982;69(2):349-55.

71. Shapiro S, Venet W, Strax P, et al. Selection, follow-up, and analysis in the Health Insurance Plan Study: a randomized trial with breast cancer screening. *National Cancer Institute monograph* 1985;67:65-74.

72. Shapiro S. The status of breast cancer screening: a quarter of a century of research. *World journal of surgery* 1989;13(1):9-18.

73. Strax P, Venet L, Shapiro S, et al. Mammography and clinical examination in mass screening for cancer of the breast. *Cancer* 1967;20(12):2184-88. doi: <https://dx.doi.org/10.1002/1097-0142%28196712%2920:12%3C2184::AID-CNCR2820201217%3E3.0.CO;2-3>

74. Strax P. Earlier detection of breast cancer. *Cancer Cytology* 1971;11(2):8-11.

75. Strax P. Mass screening for control of breast cancer. *Cancer* 1984;53(3 Suppl):665-70.

76. Andersson I, Janzon L, Sigfusson BF. Mammographic breast cancer screening--a randomized trial in Malmo, Sweden. *Maturitas* 1985;7(1):21-9.

77. Andersson I, Sigfusson BF. Screening for breast cancer in Malmo: a randomized trial. *Recent results in cancer research Fortschritte der Krebsforschung Progres dans les recherches sur le cancer* 1987;105:62-6.

78. Andersson I, Aspegren K, Janzon L, et al. Mammographic screening and mortality from breast cancer: the Malmo mammographic screening trial. *BMJ (Clinical research ed)* 1988;297(6654):943-8.

79. Autier P, Hery C, Haukka J, et al. Advanced breast cancer and breast cancer mortality in randomized controlled trials on mammography screening. *Journal of Clinical Oncology* 2009;27(35):5919-23. doi: <https://dx.doi.org/10.1200/JCO.2009.22.7041>

80. Nystrom L, Bjurstam N, Jonsson H, et al. Reduced breast cancer mortality after 20+years of follow-up in the Swedish randomized controlled mammography trials in Malmo, Stockholm, and Goteborg. *Journal of Medical Screening* 2017;24(1):34-42. doi: 10.1177/0969141316648987

81. Zackrisson S, Andersson I, Janzon L, et al. Rate of over-diagnosis of breast cancer 15 years after end of Malmo mammographic screening trial: follow-up study. *BMJ (Clinical research ed)* 2006;332(7543):689-92.

82. Mittra I, Mishra GA, Dikshit RP, et al. Effect of screening by clinical breast examination on breast cancer incidence and mortality after 20 years: prospective, cluster randomised controlled trial in Mumbai. *BMJ (Clinical research ed)* 2021;372:n256. doi: <https://dx.doi.org/10.1136/bmj.n256>

83. Semiglazov VF, Moiseenko VM. Breast self-examination for the early detection of breast cancer: a USSR/WHO controlled trial in Leningrad. *Bulletin of the World Health Organization* 1987;65(3):391-6.

84. Semiglazov VF, Moiseyenko VM, Bavli JL, et al. The role of breast self-examination in early breast cancer detection (results of the 5-years USSR/WHO randomized study in Leningrad). *European journal of epidemiology* 1992;8(4):498-502.

85. Semiglazov V, Moiseyenko V, Manikhas A, et al. Role of breast self-examination in early detection of breast cancer: Russia/WHO prospective randomized trial in St. Petersburg. *Cancer Strategy* 1999;1:145-51.

86. Thomas DB, Gao DL, Self SG, et al. Randomized trial of breast self-examination in Shanghai: methodology and preliminary results. *Journal of the National Cancer Institute* 1997;89(5):355-65.

87. Thomas DB, Gao DL, Ray RM, et al. Randomized trial of breast self-examination in Shanghai: final results. *Journal of the National Cancer Institute* 2002;94(19):1445-57.

88. Frisell J, Glas U, Hellstrom L, et al. Randomized mammographic screening for breast cancer in Stockholm. Design, first round results and comparisons. *Breast cancer research and treatment* 1986;8(1):45-54.

89. Frisell J, Eklund G, Hellstrom L, et al. The Stockholm breast cancer screening trial--5-year results and stage at discovery. *Breast cancer research and treatment* 1989;13(1):79-87.

90. Frisell J, Eklund G, Hellstrom L, et al. Randomized study of mammography screening--preliminary report on mortality in the Stockholm trial. *Breast cancer research and treatment* 1991;18(1):49-56.

91. Frisell J, Lidbrink E. The Stockholm Mammographic Screening Trial: Risks and benefits in age group 40-49 years. *Journal of the National Cancer Institute Monographs* 1997(22):49-51.

92. Frisell J, Lidbrink E, Hellstrom L, et al. Followup after 11 years--update of mortality results in the Stockholm mammographic screening trial. *Breast cancer research and treatment* 1997;45(3):263-70.

93. Ramadas K, Basu P, Mathew BS, et al. Effectiveness of triennial screening with clinical breast examination: 14-years follow-up outcomes of randomized clinical trial in Trivandrum, India. *Cancer* 2023;129(2):272-82. doi: <https://dx.doi.org/10.1002/cncr.34526>

94. Sankaranarayanan R, Ramadas K, Thara S, et al. Clinical breast examination: preliminary results from a cluster randomized controlled trial in India. *Journal of the National Cancer Institute* 2011;103(19):1476-80. doi: <https://dx.doi.org/10.1093/jnci/djr304>

95. Chen HH, Tabar L, Fagerberg G, et al. Effect of breast cancer screening after age 65. *Journal of medical screening* 1995;2(1):10-4.

96. Duffy SW, Tabar L, Fagerberg G, et al. Breast screening, prognostic factors and survival--results from the Swedish two county study. *British journal of cancer* 1991;64(6):1133-8.

97. Fagerberg G, Baldetorp L, Grontoft O. Effects of repeated mammographic screening on breast cancer stage distribution. Results from a randomised study of 92,934 women in a Swedish county. *Acta Radiologica Oncology* 1985;24(6):465-73. doi: <http://dx.doi.org/10.3109/02841868509134418>

98. Tabar L, Akerlund E, Gad A. Five-year experience with single-view mammography randomized controlled screening in Sweden. *Recent results in cancer research Fortschritte der Krebsforschung Progres dans les recherches sur le cancer* 1984;90:105-13.

99. Tabar L, Gad A, Holmberg L, et al. Significant reduction in advanced breast cancer. Results of the first seven years of mammography screening in Kopparberg, Sweden. *Diagnostic imaging in clinical medicine* 1985;54(3-4):158-64.

100. Tabar L, Fagerberg CJ, Gad A, et al. Reduction in mortality from breast cancer after mass screening with mammography. Randomised trial from the Breast Cancer Screening Working Group of the Swedish National Board of Health and Welfare. *Lancet (London, England)* 1985;1(8433):829-32.

101. Tabar L, Fagerberg G, Duffy SW, et al. The Swedish two county trial of mammographic screening for breast cancer: recent results and calculation of benefit. *Journal of epidemiology and community health* 1989;43(2):107-14.

102. Tabar L, Fagerberg G, Duffy SW, et al. Update of the Swedish two-county program of mammographic screening for breast cancer. *Radiologic clinics of North America* 1992;30(1):187-210.

103. Tabar L, Fagerberg G, Chen HH, et al. Efficacy of breast cancer screening by age. New results from the Swedish Two-County Trial. *Cancer* 1995;75(10):2507-17.

104. Tabar L, Vitak B, Chen HH, et al. Update of the Swedish Two-County Trial of breast cancer screening: histologic grade-specific and age-specific results. *Swiss surgery = Schweizer Chirurgie = Chirurgie suisse = Chirurgia svizzera* 1999;5(5):199-204.

105. Tabar L, Vitak B, Chen HH, et al. The Swedish Two-County Trial twenty years later. Updated mortality results and new insights from long-term follow-up. *Radiologic clinics of North America* 2000;38(4):625-51.

106. Tabar L, Duffy SW, Yen MF, et al. All-cause mortality among breast cancer patients in a screening trial: support for breast cancer mortality as an end point. *Journal of medical screening* 2002;9(4):159-62.

107. Tabar L, Vitak B, Chen TH-H, et al. Swedish two-county trial: impact of mammographic screening on breast cancer mortality during 3 decades. *Radiology* 2011;260(3):658-63. doi: <https://dx.doi.org/10.1148/radiol.11110469>

108. Yen AM-F, Duffy SW, Chen TH-H, et al. Long-term incidence of breast cancer by trial arm in one county of the Swedish Two-County Trial of mammographic screening. *Cancer* 2012;118(23):5728-32. doi: <https://dx.doi.org/10.1002/cncr.27580>

109. Anderson TJ, Waller M, Ellis IO, et al. Influence of annual mammography from age 40 on breast cancer pathology. *Human pathology* 2004;35(10):1252-9.

110. Duffy S, Vulkan D, Cuckle H, et al. Annual mammographic screening to reduce breast cancer mortality in women from age 40 years: long-term follow-up of the UK Age RCT. *Health technology assessment (Winchester, England)* 2020;24(55):1-24. doi: <https://dx.doi.org/10.3310/hta24550>

111. Moss S, Waller M, Anderson TJ, et al. Randomised controlled trial of mammographic screening in women from age 40: predicted mortality based on surrogate outcome measures. *British journal of cancer* 2005;92(5):955-60.

112. Moss SM, Cuckle H, Evans A, et al. Effect of mammographic screening from age 40 years on breast cancer mortality at 10 years' follow-up: a randomised controlled trial. *Lancet (London, England)* 2006;368(9552):2053-60.

113. Moss SM, Wale C, Smith R, et al. Effect of mammographic screening from age 40 years on breast cancer mortality in the UK Age trial at 17 years' follow-up: a randomised controlled trial. *The Lancet Oncology* 2015;16(9):1123-32. doi: <https://dx.doi.org/10.1016/S1470-2045(15)00128-X>

114. Anttila A, Pokhrel A, Kotaniemi-Talonen L, et al. Cervical cancer patterns with automation-assisted and conventional cytological screening: a randomized study. *International journal of cancer* 2011;128(5):1204-12. doi: <https://dx.doi.org/10.1002/ijc.25677>

115. Shastri SS, Mittra I, Mishra GA, et al. Effect of VIA screening by primary health workers: randomized controlled study in Mumbai, India. *Journal of the National Cancer Institute* 2014;106(3):dju009. doi: <https://dx.doi.org/10.1093/jnci/dju009>

116. Sankaranarayanan R, Nene BM, Dinshaw KA, et al. A cluster randomized controlled trial of visual, cytology and human papillomavirus screening for cancer of the cervix in rural India. *International Journal of Cancer* 2005;116(4):617-23. doi: <https://dx.doi.org/10.1002/ijc.21050>

117. Sankaranarayanan R, Nene BM, Shastri SS, et al. HPV screening for cervical cancer in rural India. *The New England journal of medicine* 2009;360(14):1385-94. doi: <https://dx.doi.org/10.1056/NEJMoa0808516>

118. Sankaranarayanan R, Rajkumar R, Theresa R, et al. Initial results from a randomized trial of cervical visual screening in rural south India. *International journal of cancer* 2004;109(3):461-7.

119. Sankaranarayanan R, Esmy PO, Rajkumar R, et al. Effect of visual screening on cervical cancer incidence and mortality in Tamil Nadu, India: a cluster-randomised trial. *Lancet (London, England)* 2007;370(9585):398-406.

120. Chen JG, Parkin DM, Chen QG, et al. Screening for liver cancer: results of a randomised controlled trial in Qidong, China. *Journal of medical screening* 2003;10(4):204-9.

121. Zhang B-H, Yang B-H, Tang Z-Y. Randomized controlled trial of screening for hepatocellular carcinoma. *Journal of cancer research and clinical oncology* 2004;130(7):417-22.

122. Kubik A, Polak J. Lung cancer detection. Results of a randomized prospective study in Czechoslovakia. *Cancer* 1986;57(12):2427-37.

123. Kubik A, Parkin DM, Khlat M, et al. Lack of benefit from semi-annual screening for cancer of the lung: follow-up report of a randomized controlled trial on a population of high-risk males in Czechoslovakia. *International journal of cancer* 1990;45(1):26-33.

124. Kubik A, Haerting J. Survival and mortality in a randomized study of lung cancer detection. *Neoplasma* 1990;37(4):467-75.

125. Kubik AK, Parkin DM, Zatloukal P. Czech Study on Lung Cancer Screening: post-trial follow-up of lung cancer deaths up to year 15 since enrollment. *Cancer* 2000;89(11 Suppl):2363-8.

126. Infante M, Lutman FR, Cavuto S, et al. Lung cancer screening with spiral CT: baseline results of the randomized DANTE trial. *Lung cancer (Amsterdam, Netherlands)* 2008;59(3):355-63.

127. Infante M, Cavuto S, Lutman FR, et al. A randomized study of lung cancer screening with spiral computed tomography: three-year results from the DANTE trial. *American journal of respiratory and critical care medicine* 2009;180(5):445-53. doi: <https://dx.doi.org/10.1164/rccm.200901-0076OC>

128. Infante M, Cavuto S, Lutman FR, et al. Long-Term Follow-up Results of the DANTE Trial, a Randomized Study of Lung Cancer Screening with Spiral Computed Tomography. *American journal of respiratory and critical care medicine* 2015;191(10):1166-75. doi: <https://dx.doi.org/10.1164/rccm.201408-1475OC>

129. Saghir Z, Dirksen A, Ashraf H, et al. CT screening for lung cancer brings forward early disease. The randomised Danish Lung Cancer Screening Trial: status after five annual screening rounds with low-dose CT. *Thorax* 2012;67(4):296-301. doi: <https://dx.doi.org/10.1136/thoraxjnl-2011-200736>

130. Wille MMW, Dirksen A, Ashraf H, et al. Results of the Randomized Danish Lung Cancer Screening Trial with Focus on High-Risk Profiling. *American journal of respiratory and critical care medicine* 2016;193(5):542-51. doi: <https://dx.doi.org/10.1164/rccm.201505-1040OC>

131. Paci E, Puliti D, Lopes Pegna A, et al. Mortality, survival and incidence rates in the ITALUNG randomised lung cancer screening trial. *Thorax* 2017;72(9):825-31. doi: <https://dx.doi.org/10.1136/thoraxjnl-2016-209825>

132. Paci E, Puliti D, Carozzi FM, et al. Prognostic selection and long-term survival analysis to assess overdiagnosis risk in lung cancer screening randomized trials. *Journal of medical screening* 2021;28(1):39-47. doi: <https://dx.doi.org/10.1177/0969141320923030>

133. Puliti D, Mascalchi M, Carozzi FM, et al. Decreased cardiovascular mortality in the ITALUNG lung cancer screening trial: Analysis of underlying factors. *Lung cancer (Amsterdam, Netherlands)* 2019;138:72-78. doi: <https://dx.doi.org/10.1016/j.lungcan.2019.10.006>

134. Doria-Rose VP, Marcus PM, Szabo E, et al. Randomized controlled trials of the efficacy of lung cancer screening by sputum cytology revisited: a combined mortality analysis from the Johns Hopkins Lung Project and the Memorial Sloan-Kettering Lung Study. *Cancer* 2009;115(21):5007-17. doi: <https://dx.doi.org/10.1002/cncr.24545>

135. Frost JK, Ball WC, Jr., Levin ML, et al. Early lung cancer detection: results of the initial (prevalence) radiologic and cytologic screening in the Johns Hopkins study. *The American review of respiratory disease* 1984;130(4):549-54.

136. Levin ML, Tockman MS, Frost JK, et al. Lung cancer mortality in males screened by chest X-ray and cytologic sputum examination: a preliminary report. *Recent results in cancer research Fortschritte der Krebsforschung Progres dans les recherches sur le cancer* 1982;82:138-46.

137. Doroudi M, Pinsky PF, Marcus PM. Lung Cancer Mortality in the Lung Screening Study Feasibility Trial. *JNCI cancer spectrum* 2018;2(3):pky042. doi: <https://dx.doi.org/10.1093/jncics/pky042>

138. Gohagan J, Marcus P, Fagerstrom R, et al. Baseline findings of a randomized feasibility trial of lung cancer screening with spiral CT scan vs chest radiograph: the Lung Screening Study of the National Cancer Institute. *Chest* 2004;126(1):114-21.

139. Gohagan JK, Marcus PM, Fagerstrom RM, et al. Final results of the Lung Screening Study, a randomized feasibility study of spiral CT versus chest X-ray screening for lung cancer. *Lung cancer (Amsterdam, Netherlands)* 2005;47(1):9-15.

140. Becker N, Motsch E, Gross ML, et al. Randomized Study on Early Detection of Lung Cancer with MSCT in Germany: Results of the First 3 Years of Follow-up After Randomization. *Journal of thoracic oncology : official publication of the International Association for the Study of Lung Cancer* 2015;10(6):890-6. doi: <https://dx.doi.org/10.1097/JTO.0000000000000530>

141. Becker N, Motsch E, Trotter A, et al. Lung cancer mortality reduction by LDCT screening-Results from the randomized German LUSI trial. *International journal of cancer* 2020;146(6):1503-13. doi: <https://dx.doi.org/10.1002/ijc.32486>

142. Gonzalez Maldonado S, Motsch E, Trotter A, et al. Overdiagnosis in lung cancer screening: Estimates from the German Lung Cancer Screening Intervention Trial. *International journal of cancer* 2021;148(5):1097-105. doi: <https://dx.doi.org/10.1002/ijc.33295>

143. Baker SG, Kramer BS, Prorok PC. Early reporting for cancer screening trials. *Journal of medical screening* 2008;15(3):122-9. doi: <https://dx.doi.org/10.1258/jms.2008.007058>

144. Doria-Rose VP, Marcus PM. Death certificates provide an adequate source of cause of death information when evaluating lung cancer mortality: an example from the Mayo Lung Project. *Lung cancer (Amsterdam, Netherlands)* 2009;63(2):295-300. doi: <https://dx.doi.org/10.1016/j.lungcan.2008.05.019>

145. Fontana RS, Taylor WF. 978. *UICC Technical Report Series* 1978;VOL 40:233-53.

146. Fontana F. Screening for lung cancer. In: Miller AB, ed. Screening for cancer. New York: Academic Press 1985:377-95.

147. Fontana RS, Sanderson DR, Woolner LB, et al. Screening for lung cancer. A critique of the Mayo Lung Project. *Cancer* 1991;67(4 Suppl):1155-64.

148. Marcus PM, Bergstralh EJ, Fagerstrom RM, et al. Lung cancer mortality in the Mayo Lung Project: impact of extended follow-up. *Journal of the National Cancer Institute* 2000;92(16):1308-16.

149. Sanderson D, Fontana R. Results of Mayo lung project: an interim report. *Recent results in cancer research Fortschritte der Krebsforschung Progres dans les recherches sur le cancer* 1982;82:179-86.

150. Taylor WF, Fontana RS, Uhlenhopp MA, et al. Some results of screening for early lung cancer. *Cancer* 1981;47(5 Suppl):1114-20.

151. Flehinger BJ, Melamed MR, Zaman MB. Early lung cancer detection: Results of the initial (prevalence) radiologic and cytologic screening in he Memorial Sloan-Kettering Study. *American Review of Respiratory Disease* 1984;130(4):555-60.

152. Melamed MR, Flehinger BJ, Zaman MB, et al. Screening for early lung cancer. Results of the Memorial Sloan-Kettering study in New York. *Chest* 1984;86(1):44-53.

153. Melamed MR, Flehinger BJ, Zaman MB. Impact of early detection on the clinical course of lung cancer. *The Surgical clinics of North America* 1987;67(5):909-24.

154. Infante M, Sestini S, Galeone C, et al. Lung cancer screening with low-dose spiral computed tomography: evidence from a pooled analysis of two Italian randomized trials. *European journal of cancer prevention : the official journal of the European Cancer Prevention Organisation (ECP)* 2017;26(4):324-29. doi: <https://dx.doi.org/10.1097/CEJ.0000000000000264>

155. Pastorino U, Rossi M, Rosato V, et al. Annual or biennial CT screening versus observation in heavy smokers: 5-year results of the MILD trial. *European journal of cancer prevention : the official journal of the European Cancer Prevention Organisation (ECP)* 2012;21(3):308-15. doi: <https://dx.doi.org/10.1097/CEJ.0b013e328351e1b6>

156. Pastorino U, Sverzellati N, Sestini S, et al. Ten-year results of the Multicentric Italian Lung Detection trial demonstrate the safety and efficacy of biennial lung cancer screening. *European journal of cancer (Oxford, England : 1990)* 2019;118:142-48. doi: <https://dx.doi.org/10.1016/j.ejca.2019.06.009>

157. Pastorino U, Silva M, Sestini S, et al. Prolonged lung cancer screening reduced 10-year mortality in the MILD trial: new confirmation of lung cancer screening efficacy. *Annals of oncology : official journal of the European Society for Medical Oncology* 2019;30(7):1162-69. doi: <https://dx.doi.org/10.1093/annonc/mdz117>

158. Sverzellati N, Silva M, Calareso G, et al. Low-dose computed tomography for lung cancer screening: comparison of performance between annual and biennial screen. *European radiology* 2016;26(11):3821-29.

159. de Koning HJ, van der Aalst CM, de Jong PA, et al. Reduced Lung-Cancer Mortality with Volume CT Screening in a Randomized Trial. *The New England journal of medicine* 2020;382(6):503-13. doi: <https://dx.doi.org/10.1056/NEJMoa1911793>

160. National Lung Screening Trial Research T, Aberle DR, Adams AM, et al. Reduced lung-cancer mortality with low-dose computed tomographic screening. *The New England journal of medicine* 2011;365(5):395-409. doi: <https://dx.doi.org/10.1056/NEJMoa1102873>

161. National Lung Screening Trial Research T. Lung Cancer Incidence and Mortality with Extended Follow-up in the National Lung Screening Trial. *Journal of thoracic oncology : official publication of the International Association for the Study of Lung Cancer* 2019;14(10):1732-42. doi: <https://dx.doi.org/10.1016/j.jtho.2019.05.044>

162. Pinsky PF, Church TR, Izmirlian G, et al. The National Lung Screening Trial: results stratified by demographics, smoking history, and lung cancer histology. *Cancer* 2013;119(22):3976-83. doi: <https://dx.doi.org/10.1002/cncr.28326>

163. Oken MM, Hocking WG, Kvale PA, et al. Screening by chest radiograph and lung cancer mortality: the Prostate, Lung, Colorectal, and Ovarian (PLCO) randomized trial. *JAMA* 2011;306(17):1865-73. doi: <https://dx.doi.org/10.1001/jama.2011.1591>

164. Field JK, Vulkan D, Davies MPA, et al. Lung cancer mortality reduction by LDCT screening: UKLS randomised trial results and international meta-analysis. *The Lancet regional health Europe* 2021;10:100179. doi: <https://dx.doi.org/10.1016/j.lanepe.2021.100179>

165. Ji MF, Sheng W, Cheng WM, et al. Incidence and mortality of nasopharyngeal carcinoma: interim analysis of a cluster randomized controlled screening trial (PRO-NPC-001) in southern China. *Annals of oncology : official journal of the European Society for Medical Oncology* 2019;30(10):1630-37. doi: <https://dx.doi.org/10.1093/annonc/mdz231>

166. Ramadas K, Sankaranarayanan R, Jacob BJ, et al. Interim results from a cluster randomized controlled oral cancer screening trial in Kerala, India. *Oral oncology* 2003;39(6):580-8.

167. Sankaranarayanan R, Mathew B, Jacob BJ, et al. Early findings from a community-based, cluster-randomized, controlled oral cancer screening trial in Kerala, India. The Trivandrum Oral Cancer Screening Study Group. *Cancer* 2000;88(3):664-73.

168. Sankaranarayanan R, Ramadas K, Thomas G, et al. Effect of screening on oral cancer mortality in Kerala, India: a cluster-randomised controlled trial. *Lancet (London, England)* 2005;365(9475):1927-33.

169. Sankaranarayanan R, Ramadas K, Thara S, et al. Long term effect of visual screening on oral cancer incidence and mortality in a randomized trial in Kerala, India. *Oral oncology* 2013;49(4):314-21. doi: <https://dx.doi.org/10.1016/j.oraloncology.2012.11.004>

170. Buys SS, Partridge E, Black A, et al. Effect of screening on ovarian cancer mortality: the Prostate, Lung, Colorectal and Ovarian (PLCO) Cancer Screening Randomized Controlled Trial. *JAMA* 2011;305(22):2295-303. doi: <https://dx.doi.org/10.1001/jama.2011.766>

171. Pinsky PF, Yu K, Kramer BS, et al. Extended mortality results for ovarian cancer screening in the PLCO trial with median 15years follow-up. *Gynecologic oncology* 2016;143(2):270-75. doi: <https://dx.doi.org/10.1016/j.ygyno.2016.08.334>

172. Temkin SM, Miller EA, Samimi G, et al. Outcomes from ovarian cancer screening in the PLCO trial: Histologic heterogeneity impacts detection, overdiagnosis and survival. *European journal of cancer (Oxford, England : 1990)* 2017;87:182-88. doi: <https://dx.doi.org/10.1016/j.ejca.2017.10.015>

173. Jacobs IJ, Skates SJ, MacDonald N, et al. Screening for ovarian cancer: a pilot randomised controlled trial. *Lancet (London, England)* 1999;353(9160):1207-10.

174. Jacobs IJ, Menon U, Ryan A, et al. Ovarian cancer screening and mortality in the UK Collaborative Trial of Ovarian Cancer Screening (UKCTOCS): a randomised controlled trial. *Lancet (London, England)* 2016;387(10022):945-56. doi: <https://dx.doi.org/10.1016/S0140-6736(15)01224-6>

175. Menon U, Gentry-Maharaj A, Burnell M, et al. Ovarian cancer population screening and mortality after long-term follow-up in the UK Collaborative Trial of Ovarian Cancer Screening (UKCTOCS): a randomised controlled trial. *Lancet (London, England)* 2021;397(10290):2182-93. doi: <https://dx.doi.org/10.1016/S0140-6736(21)00731-5>

176. Martin RM, Donovan JL, Turner EL, et al. Effect of a Low-Intensity PSA-Based Screening Intervention on Prostate Cancer Mortality: The CAP Randomized Clinical Trial. *JAMA* 2018;319(9):883-95. doi: <https://dx.doi.org/10.1001/jama.2018.0154>

177. Aus G, Bergdahl S, Lodding P, et al. Prostate cancer screening decreases the absolute risk of being diagnosed with advanced prostate cancer--results from a prospective, population-based randomized controlled trial. *European urology* 2007;51(3):659-64.

178. Berenguer A, Lujan M, Paez A, et al. The Spanish contribution to the European Randomized Study of Screening for Prostate Cancer. *BJU international* 2003;92 Suppl 2:33-8.

179. Boevee SJ, Venderbos LDF, Tammela TLJ, et al. Change of tumour characteristics and treatment over time in both arms of the European Randomized study of Screening for Prostate Cancer. *European journal of cancer (Oxford, England : 1990)* 2010;46(17):3082-9. doi: <https://dx.doi.org/10.1016/j.ejca.2010.09.033>

180. Franlund M, Mansson M, Godtman RA, et al. Results from 22 years of Followup in the Goteborg Randomized Population-Based Prostate Cancer Screening Trial. *Journal of Urology* 2022;208(2):292-300. doi: <https://dx.doi.org/10.1097/JU.0000000000002696>

181. Hugosson J, Aus G, Lilja H, et al. Results of a randomized, population-based study of biennial screening using serum prostate-specific antigen measurement to detect prostate carcinoma. *Cancer* 2004;100(7):1397-405.

182. Hugosson J, Carlsson S, Aus G, et al. Mortality results from the Goteborg randomised population-based prostate-cancer screening trial. *The Lancet Oncology* 2010;11(8):725-32. doi: <https://dx.doi.org/10.1016/S1470-2045(10)70146-7>

183. Hugosson J, Godtman RA, Carlsson SV, et al. Eighteen-year follow-up of the Goteborg Randomized Population-based Prostate Cancer Screening Trial: effect of sociodemographic variables on participation, prostate cancer incidence and mortality. *Scandinavian journal of urology* 2018;52(1):27-37. doi: <https://dx.doi.org/10.1080/21681805.2017.1411392>

184. Hugosson J, Roobol MJ, Mansson M, et al. A 16-yr Follow-up of the European Randomized study of Screening for Prostate Cancer. *European urology* 2019;76(1):43-51. doi: <https://dx.doi.org/10.1016/j.eururo.2019.02.009>

185. Kilpelainen TP, Auvinen A, Maattanen L, et al. Results of the three rounds of the Finnish Prostate Cancer Screening Trial--the incidence of advanced cancer is decreased by screening. *International journal of cancer* 2010;127(7):1699-705. doi: <https://dx.doi.org/10.1002/ijc.25368>

186. Kilpelainen TP, Tammela TL, Malila N, et al. Prostate cancer mortality in the Finnish randomized screening trial. *Journal of the National Cancer Institute* 2013;105(10):719-25. doi: <https://dx.doi.org/10.1093/jnci/djt038>

187. Lujan M, Paez A, Angulo JC, et al. Prostate cancer incidence and mortality in the Spanish section of the European Randomized Study of Screening for Prostate Cancer (ERSPC). *Prostate cancer and prostatic diseases* 2014;17(2):187-91. doi: <https://dx.doi.org/10.1038/pcan.2014.7>

188. Lujan M, Paez A, Angulo JC, et al. Update of the results of the Spanish branch of the European Randomized Study on Screening for Prostate Cancer (ERSPC). *Actas urologicas espanolas* 2015;39(7):405-13. doi: <https://dx.doi.org/10.1016/j.acuro.2015.02.003>

189. Lujan Galan M, Paez Borda A, Llanes Gonzalez L, et al. Results of the spanish section of the European Randomized Study of Screening for Prostate Cancer (ERSPC). Update after 21 years of follow-up. *Resultados de la rama espanola del Estudio Randomizado Europeo de Screening del Cancer de Prostata (ERSPC) Actualizacion tras 21 anos de seguimiento* 2020;44(6):430-36. doi: <https://dx.doi.org/10.1016/j.acuro.2020.01.005>

190. Makinen T, Tammela TLJ, Hakama M, et al. Tumor characteristics in a population-based prostate cancer screening trial with prostate-specific antigen. *Clinical cancer research : an official journal of the American Association for Cancer Research* 2003;9(7):2435-9.

191. Pakarainen T, Nevalainen J, Talala K, et al. The Number of Screening Cycles Needed to Reduce Prostate Cancer Mortality in the Finnish Section of the European Randomized Study of Prostate Cancer (ERSPC). *Clinical cancer research : an official journal of the American Association for Cancer Research* 2019;25(2):839-43. doi: <https://dx.doi.org/10.1158/1078-0432.CCR-18-1807>

192. Postma R, van Leenders AGJLH, Roobol MJ, et al. Tumour features in the control and screening arm of a randomized trial of prostate cancer. *European urology* 2006;50(1):70-5.

193. Roobol MJ, Kranse R, Bangma CH, et al. Screening for prostate cancer: results of the Rotterdam section of the European randomized study of screening for prostate cancer. *European urology* 2013;64(4):530-9. doi: <https://dx.doi.org/10.1016/j.eururo.2013.05.030>

194. Schroder FH, Hugosson J, Roobol MJ, et al. Screening and prostate-cancer mortality in a randomized European study. *The New England journal of medicine* 2009;360(13):1320-8. doi: <https://dx.doi.org/10.1056/NEJMoa0810084>

195. Schroder FH, Hugosson J, Roobol MJ, et al. Prostate-cancer mortality at 11 years of follow-up. *The New England journal of medicine* 2012;366(11):981-90. doi: <https://dx.doi.org/10.1056/NEJMoa1113135>

196. Schroder FH, Hugosson J, Roobol MJ, et al. Screening and prostate cancer mortality: results of the European Randomised Study of Screening for Prostate Cancer (ERSPC) at 13 years of follow-up. *Lancet (London, England)* 2014;384(9959):2027-35. doi: <https://dx.doi.org/10.1016/S0140-6736(14)60525-0>

197. van der Cruijsen-Koeter IW, Vis AN, Roobol MJ, et al. Comparison of screen detected and clinically diagnosed prostate cancer in the European randomized study of screening for prostate cancer, section rotterdam. *The Journal of urology* 2005;174(1):121-5.

198. van Leeuwen PJ, Kranse R, Hakulinen T, et al. Disease-specific mortality may underestimate the total effect of prostate cancer screening. *Journal of medical screening* 2010;17(4):204-10. doi: <https://dx.doi.org/10.1258/jms.2010.010074>

199. Villers A, Bessaoud F, Tretarre B, et al. Contamination in control group led to no effect of PSA-based screening on prostate cancer mortality at 9 years follow-up: Results of the French section of European Randomized Study of Screening for Prostate Cancer (ERSPC). *Progres en urologie : journal de l'Association francaise d'urologie et de la Societe francaise d'urologie* 2020;30(5):252-60. doi: <https://dx.doi.org/10.1016/j.purol.2020.02.011>

200. Osses DF, Remmers S, Schroder FH, et al. Results of Prostate Cancer Screening in a Unique Cohort at 19yr of Follow-up. *European urology* 2019;75(3):374-77. doi: <https://dx.doi.org/10.1016/j.eururo.2018.10.053>

201. Sandblom G, Varenhorst E, Lofman O, et al. Clinical consequences of screening for prostate cancer: 15 years follow-up of a randomised controlled trial in Sweden. *European urology* 2004;46(6):717-24.

202. Sandblom G, Varenhorst E, Rosell J, et al. Randomised prostate cancer screening trial: 20 year follow-up. *BMJ (Clinical research ed)* 2011;342:d1539. doi: <https://dx.doi.org/10.1136/bmj.d1539>

203. Andriole GL, Crawford ED, Grubb RL, 3rd, et al. Mortality results from a randomized prostate-cancer screening trial. *The New England journal of medicine* 2009;360(13):1310-9. doi: <https://dx.doi.org/10.1056/NEJMoa0810696>

204. Andriole GL, Crawford ED, Grubb RL, 3rd, et al. Prostate cancer screening in the randomized Prostate, Lung, Colorectal, and Ovarian Cancer Screening Trial: mortality results after 13 years of follow-up. *Journal of the National Cancer Institute* 2012;104(2):125-32. doi: <https://dx.doi.org/10.1093/jnci/djr500>

205. Crawford ED, Grubb R, 3rd, Black A, et al. Comorbidity and mortality results from a randomized prostate cancer screening trial. *Journal of clinical oncology : official journal of the American Society of Clinical Oncology* 2011;29(4):355-61. doi: <https://dx.doi.org/10.1200/JCO.2010.30.5979>

206. Pinsky PF, Black A, Parnes HL, et al. Prostate cancer specific survival in the Prostate, Lung, Colorectal, and Ovarian (PLCO) Cancer Screening Trial. *Cancer epidemiology* 2012;36(6):e401-6. doi: <https://dx.doi.org/10.1016/j.canep.2012.08.008>

207. Pinsky PF, Prorok PC, Yu K, et al. Extended mortality results for prostate cancer screening in the PLCO trial with median follow-up of 15 years. *Cancer* 2017;123(4):592-99. doi: <https://dx.doi.org/10.1002/cncr.30474>

208. Pinsky PF, Miller E, Prorok P, et al. Extended follow-up for prostate cancer incidence and mortality among participants in the Prostate, Lung, Colorectal and Ovarian randomized cancer screening trial. *BJU international* 2019;123(5):854-60. doi: <https://dx.doi.org/10.1111/bju.14580>

209. Pinsky PF, Black A, Daugherty SE, et al. Metastatic prostate cancer at diagnosis and through progression in the Prostate, Lung, Colorectal, and Ovarian Cancer Screening Trial. *Cancer* 2019;125(17):2965-74. doi: <https://dx.doi.org/10.1002/cncr.32176>

210. Prandoni P, Bernardi E, Valle FD, et al. Extensive Computed Tomography versus Limited Screening for Detection of Occult Cancer in Unprovoked Venous Thromboembolism: A Multicenter, Controlled, Randomized Clinical Trial. *Seminars in thrombosis and hemostasis* 2016;42(8):884-90.

211. Robin P, Le Roux P-Y, Planquette B, et al. Limited screening with versus without (18)F-fluorodeoxyglucose PET/CT for occult malignancy in unprovoked venous thromboembolism: an open-label randomised controlled trial. *The Lancet Oncology* 2016;17(2):193-99. doi: <https://dx.doi.org/10.1016/S1470-2045(15)00480-5>

212. Piccioli A, Lensing AWA, Prins MH, et al. Extensive screening for occult malignant disease in idiopathic venous thromboembolism: a prospective randomized clinical trial. *Journal of thrombosis and haemostasis : JTH* 2004;2(6):884-9.

## **Section 4. Detailed meta-analytic results, including sensitivity and subgroup analyses**

## Table S16. The estimates of the screening effect on the absolute incidence of late-stage cancer, for three timepoints (if available).

Timepoints:

1. during or soon after the completion of the intervention phase,
2. at the time of the “primary” intermediate endpoint, and
3. at the timepoint at which the “main” mortality endpoint was reported.

Cervical cancer is excluded because temporal patterns are expected to differ when prevention of cancer is the main goal of a screening intervention.

| **Cancer type** | **Trial acronym/ name** | **“Main” mortality outcome** | **Timing of intermediate outcome** | | | | | |
| --- | --- | --- | --- | --- | --- | --- | --- | --- |
|  |  |  | **INTERVENTION PHASE (ongoing or completed)*** | **Estimated effect**  **RR (95% CI)** | **MAIN ANALYSIS TIMEPOINT**** | **Estimated effect**  **RR (95% CI)** | **AT MORTALITY TIMEPOINT** | **Estimated effect**  **RR (95% CI)** |
| Bowel | Finnish | CRC mortality; FU until 31/12/2012 | Incidence of non-localised (advanced) CRC; FU until 31/12/2007 | 1.36 (0.94-1.96) | Absolute incidence of CRC with N≥1; FU until 31/12/2011 | 1.07 (0.90-1.27) | NR | NR |
| Bowel | Funen | Death from CRC and complications from treatment; FU until August 1995 | Incidence of CRC with Dukes' C/D; FU until October 1988 | 0.93 (0.64-1.35) | Mortality timepoint | NA | Incidence of late-stage CRC (Dukes’ C, distant spread, no classification); FU until August 1995 | 0.84 (0.70-1.01) |
| Bowel | Gothenburg | CRC mortality (all cohorts: DOB 1918-1931); FU to 31/12/2001 | Incidence of Dukes' D CRC; all cohorts (DOB 1918-1931); up to October 1992 | 0.92 (0.61-1.38) | Mortality timepoint | NA | Incidence of Dukes' D CRC; all cohorts (DOB 1918-1931); FU to 31/12/2001 | 0.95 (0.76-1.18) |
| Bowel | Minnesota | CRC mortality; 13 years of FU;  annual FOBT vs. UC | NR | NR | Mortality timepoint | NA | Incidence of Dukes' D CRC; 13 years of FU; annual FOBT vs. UC | 0.50 (0.33-0.76) |
| Bowel | Minnesota | CRC mortality; 13 years of FU;  biennial FOBT vs. UC | NR | NR | Mortality timepoint | NA | Incidence of Dukes' D CRC; 13 years of FU; biennial FOBT vs. UC | 0.62 (0.42-0.92) |
| Bowel | NORCCAP | CRC mortality in people aged 55-64 years at entry; FU until 31/12/2015 | NR | NR | Incidence of Dukes’ C/distant spread CRC in people aged 55-64 years at entry; FU until 31/12/2006 | 0.90 (0.70-1.15) | NR | NR |
| Bowel | NordICC | CRC mortality; 10-year risk | NR | NR | Mortality timepoint | NA | Incidence Dukes' C/D CRC; median 10-year FU | 0.80 (0.65-1.00) |
| Bowel | Nottingham | CRC mortality (verified cause of death); FU until 30/06/1995 | Incidence of Dukes’ C/D CRC (from first 107,349 subjects); mean FU time of 3 years | 1.05 (0.75-1.48) | Mortality timepoint | NA | Incidence of Dukes’ C/D CRC; FU until 30/06/1995 | 0.91 (0.80-1.04) |
| Bowel | PLCO  (bowel) | CRC-specific mortality; 13 years since randomisation or 31/12/2009 | NR | NR | Mortality timepoint | NA | Incidence of stage III/IV CRC; 13 years since randomisation or 31/12/2009 | 0.71 (0.62-0.81) |
| Bowel | SCORE | CRC mortality; FU until 31/12/2008 (5 centres) or 31/12/2007 (1 centre) | NR | NR | Incidence of stage III-IV CRC; FU until 31/12/2007 | 0.73 (0.57-0.94) | NR | NR |
| Bowel | Telemark  Polyp I | CRC mortality; 1983-1995 | NR | NR | Absolute incidence of Dukes' C or D CRC; FU for 10 years (1983-1993) | 0.50 (0.05-5.48) | Incidence of Dukes' C or D CRC; FU for 13 years (1983-1995) | 0.25 (0.03-2.22) |
| Bowel | UKFSST | CRC mortality; cause of death verified by expert coder; FU until 31/12/2008 | NR | NR | Mortality timepoint | NA | Incidence of invasive CRC*** (all sites); FU until 31/12/2008 | 0.77 (0.70-0.84) |
| Breast | CNBSS-1 | Breast cancer mortality; 7 years of FU | Incidence of node-positive breast cancers; first 5 years after randomisation | 1.38 (1.02-1.86) | Mortality timepoint | NA | Absolute incidence of node-positive, invasive breast cancers; FU through 7 years | 1.55 (1.13-2.11) |
| Breast | CNBSS-2 | Breast cancer mortality; 7 years of FU | Incidence of node-positive breast cancers; first 5 years after randomisation | 1.03 (0.78-1.36) | Mortality timepoint | NA | Incidence of node-positive, invasive breast cancers; FU through 7 years | 1.09 (0.82-1.45) |
| Breast | Edinburgh | Breast cancer mortality; 7 years of FU (Cohort 1) | NR | NR | Mortality timepoint | NA | Incidence of stage III-IV breast cancers; 7 years of FU | 0.63 (0.46-0.87) |
| Breast | Gothenburg | Breast cancer mortality from cases diagnosed in screening period; blinded review of cause of death by independent endpoint committee; FU until 31/12/1996 | Incidence of node-positive breast cancers detected in screening period | 0.80 (0.61-1.05) | Intervention phase | NA | NR | NR |
| Breast | HIP NY | Breast cancer mortality; 10 completed years from entry | Incidence of node-positive breast cancers diagnosed in the first year after entry | 1.00 (0.59-1.69) | Incidence of node-positive breast cancers; 5 years of FU | 0.84 (0.65-1.10) | NR | NR |
| Breast | Malmo | Breast cancer mortality; FU until 31/12/1986 | Incidence of stage II-IV breast cancer; 5 years after study entry  *Estimates available up to 10 years since study entry* | 0.88 (NR) | Mortality timepoint | NA | Incidence of stage II-IV breast cancer; FU to 31/12/1986 | 0.83 (0.68-1.00) |
| Breast | Mumbai | Breast cancer mortality; FU until May 2018 | NR | NR | Mortality timepoint | NA | Incidence of stage III-IV breast cancer; FU until May 2018 | 0.81 (0.68-0.97) |
| Breast | Russia /  WHO | Breast cancer mortality of breast cancers diagnosed up to 31/12/1994; FU until 31/12/1997 | Incidence of breast cancers with N+ stage (N1-N2, node-positive) at diagnosis; 1985-1990 | 1.02 (0.77-1.35) | Incidence of breast cancers with N+ stage (N1-N2, node positive) at diagnosis; 1985-1994 | 1.20 (1.01-1.43) | NR | NR |
| Breast | Shanghai | Breast cancer mortality; FU until 31/12/2000 | NR | NR | Mortality timepoint | NA | Incidence of node-positive breast cancers (N1, N2, or N3) at diagnosis; FU until 31/12/2000 | 0.92 (0.79-1.07) |
| Breast | Stockholm | Breast cancer mortality in women aged 40-64 years; cancers diagnosed in screening period only; FU until 31/12/1994 | Incidence of stage II-IV breast cancers; 1981-1984 (CP corrected for size; SP excluding women born on 31^st^)  *Estimates from 1981 up to 1986* | 1.23 (0.94-1.61) | Incidence of stage II-IV breast cancers; 1981-1986 | 0.88 (0.68-1.12) | NR | NR |
| Breast | Trivandrum | Breast cancer mortality; FU until 31/12/2019 | NR | NR | Mortality timepoint | NA | Incidence of stage III-IV breast cancers; FU until 31/12/2019 | 1.18 (0.92-1.52) |
| Breast | Two-County | Breast cancer mortality (women aged 40-74 years); FU until 31/12/1984 | Incidence of stage II+ breast cancers (women aged 40-74 years); 3 years of FU  *Estimates available 1-8 years since randomisation* | 0.85 (0.71-1.01) | Mortality timepoint | NA | Incidence of stage II+ breast cancers (women aged 40-74 years); diagnosed up to 31/12/1984 | 0.79 (0.69-0.91) |
| Breast | UK Age | Breast cancer mortality; breast cancers diagnosed during the intervention phase; FU until 31/12/2011 | Incidence of node-positive breast cancers diagnosed up to 31/12/1999 | 0.89 (0.72-1.10) | Intervention phase | NA | NR | NR |
|  |  |  | Incidence of node-positive breast cancers diagnosed during the intervention period (before first NHSBSP screen) | 0.90 (0.78-1.05) |  |  |  |  |
| Liver | Qidong | Liver cancer mortality; FU until 31/12/1995 | NR | NR | Mortality timepoint | NA | Incidence of stage III^§^ liver cancer; FU until 31/12/1995 | 0.54 (0.37-0.81) |
| Liver | Shanghai | Liver cancer mortality; FU until 31/12/1997 | NR | NR | Mortality timepoint | NA | Incidence of stage III^§^ liver cancer; FU until 31/12/1997 | 0.53 (0.32-0.88) |
| Lung | Czech  study | Lung cancer mortality; 15 years of FU | Incidence of stage III^§§^ lung cancers (incident); diagnosed in 3-year period after baseline exam (including 3-year screen) | 1.24 (0.65-2.34) | Incidence of stage III^§§^ lung cancers (incident); 0-6 years after study entry | 1.15 (0.78-1.71) | NR | NR |
| Lung | DANTE | Lung cancer mortality; FU until 15/05/2013 | Incidence of stage II-IV lung cancers diagnosed up to 25/01/2008 | 1.14 (0.66-1.97) | Mortality timepoint | NA | Incidence of stage II-IV lung cancers diagnosed up to 15/05/2013 | 0.94 (0.64-1.38) |
| Lung | DLCST | Lung cancer mortality; FU until 07/04/2015 | Incidence of stage III-IV NSCLC; FU until 31/03/2010 | 1.90 (0.89-4.08) | Mortality timepoint | NA | Incidence of stage III-IV lung cancer; FU until 07/04/2015 | 1.12 (0.74-1.70) |
| Lung | ITALUNG | Lung cancer mortality; FU until 31/12/2014 | NR | NR | Incidence of stage III-IV lung cancers; diagnosed up to 31/12/2013 | 0.76 (0.48-1.19) | NR | NR |
| Lung | Johns  Hopkins | Lung cancer mortality; up to 9 years after study entry | NR | NR | Mortality timepoint | NA | Incidence of stage II-IV lung cancers; 9 years of FU after study entry | 0.86 (0.68-1.09) |
| Lung | LSS | Lung cancer mortality; median FU of 5.2 years (through 2005) | Incidence of stage III-IV lung cancer; baseline screen, year 1 screen and interval cancers | 1.78 (0.79-4.01) | Intervention phase | NA | NR | NR |
| Lung | LUSI | Lung cancer mortality; FU until 30/04/2018 | Incidence of stage II-IV lung cancer; 5 years after randomisation (active screening period) | 0.60 (0.35-1.05) | Incidence of stage II-IV lung cancer; 7 years after randomisation | 0.55 (0.34-0.88) | Incidence of stage II-IV lung cancer; FU until 30/04/2018 | 0.60 (0.40-0.90) |
| Lung | MLP | Lung cancer mortality; FU until 01/07/1983 | Incidence of stage III lung cancers; FU until 01/04/1980 | 0.86 (0.58-1.28) | Mortality timepoint | NA | Incidence of stage III-IV lung cancer; FU until 01/07/1983 | 0.98 (0.75-1.27) |
| Lung | MSK | Lung cancer mortality; FU until 9 years since study entry | Incidence of stage II-III lung cancer; whole screening period | 1.01 (0.75-1.36) | Mortality timepoint | NA | Incidence of stage II-IV lung cancers; FU until 9 years since study entry | 1.01 (0.77-1.33) |
| Lung | MILD | Lung cancer mortality; FU until June 2018 | NR | NR | Mortality timepoint | NA | Incidence of stage II-IV lung cancers; FU until June 2018 | 0.74 (0.50-1.10) |
| Lung | NELSON | Lung cancer mortality in men; at 10-year FU or 31/12/2015 | NR | NR | Mortality timepoint | NA | Incidence of stage III-IV lung cancers in men; at 10-year FU or 31/12/2015 | 0.71 (0.58-0.87) |
| Lung | NLST | Lung cancer mortality; FU until 31/12/2009 | Incidence of stage III-IV lung cancers; up to 1 year after T2 screening round | 0.90 (0.75-1.07) | Mortality timepoint | NA | Incidence of stage III-IV lung cancers; FU until 31/12/2009 | 0.79 (0.70-0.89) |
| Lung | PLCO  (Lung) | Lung-specific mortality; 13 years after randomisation or 31/12/2009 | NR | NR | Incidence of stage III-IV NSCLC; 7 years since randomisation | 0.94 (0.84-1.05) | Incidence of stage III-IV NSCLC; 13 years of FU or 31/12/2009 | 0.98 (0.89-1.07) |
| Lung | UKLS | Lung cancer mortality; FU until 29/02/2020 | Incidence of stage III-IV lung cancers; 1 year since study entry  *Estimates 1-6 years post randomisation* | 0.50 (0.19-1.33) | Incidence of stage III-IV lung cancers; 4 years since study entry | 0.44 (0.23-0.87) | Incidence of stage III-IV lung cancers; FU until 31/12/2019 | 0.43 (0.24-0.78) |
| Naso-pharyngeal | PRO-NPC-001 | NPC mortality; 2009-2014 | NR | NR | Mortality timepoint | NA | Incidence of stage III-IV NPC; FU until 31/12/2014 | 0.78 (0.57-1.07) |
| Oral | Trivandrum | Oral cancer mortality; FU until 31/12/2004 | Incidence of stage III-IV oral cancers; Rounds 1-2 (October 1995-June 2002) | 0.99 (0.69-1.40) | Mortality timepoint | NA | Incidence of stage III-IV oral cancers; FU until 31/12/2004 | 0.89 (0.68-1.16) |
| Ovarian | PLCO  (Ovarian) | Ovarian cancer (including primary peritoneal and fallopian tube cancer) mortality; FU for 13 years or 28/02/2010 | Incidence of stage III-IV ovarian cancer (including primary peritoneal and fallopian tube cancer); study years 0-5 (screening period) | 1.31 (0.97-1.78) | Mortality timepoint | NA | Incidence of stage III-IV ovarian cancer (including primary peritoneal and fallopian tube cancer); FU for 13 years or until 28/02/2010 | 1.20 (0.96-1.51) |
| Ovarian | UK Pilot | Ovarian cancer mortality; FU until January 1998 | NR | NR | Mortality timepoint | NA | Incidence of stage III-IV ovarian cancers; FU until 1997 | 0.61 (0.29-1.30) |
| Ovarian | UKCTOCS | Ovarian cancer mortality (primary analysis); FU until 31/12/2014;  MMS vs. UC | NR | NR | Mortality timepoint | NA | Incidence of stage III-IV primary ovarian cancers; FU until 31/12/2014; MMS vs. UC | 0.83 (0.70-0.99) |
| Ovarian | UKCTOCS | Ovarian cancer mortality (primary analysis); FU until 31/12/2014;  TVU vs. UC | NR | NR | Mortality timepoint | NA | Incidence of stage III-IV primary ovarian cancers; FU until 31/12/2014; TVU vs. UC | 0.91 (0.77-1.08) |
| Prostate | ERSPC | Prostate cancer mortality; whole trial excluding France; age 55-69 years; up to 31/12/2008 | NR | NR | Mortality timepoint | NA | Incidence of prostate cancers with Gleason score ≥8; whole trial (excluding France); age 55-69 years; up to 31/12/2008 | 0.82 (0.74-0.91) |
| Prostate | ERSPC  Pilot 1 | Prostate cancer mortality; FU for median 19 years (IQR 12-24) | NR | NR | Mortality timepoint | NA | Incidence of prostate cancers with Gleason score ≥3+4; FU for median 19 years (IQR 12-24) | 0.86 (0.50-1.48) |
| Prostate | Norrkoping | Prostate cancer mortality; cancers diagnosed up to 31/12/1999; FU until 31/12/2008 | NR | NR | Incidence of advanced tumours (T3-T4, N1 or MX/M1); cancers diagnosed up to 31/12/1999 | 0.87 (0.62-1.23) | NR | NR |
| Prostate | PLCO  (Prostate) | Prostate cancer mortality; 13 years of FU or until 31/12/2009 | NR | NR | Incidence of prostate cancer with Gleason score ≥8; through 10 years of FU | 0.85 (0.73-0.99) | Incidence of prostate cancer with Gleason score ≥8; 13 years of FU or until 31/12/2009 | 0.89 (0.77-1.01) |
| Prostate | UK CAP | Definite, probable, or intervention-related prostate cancer mortality; median FU of 10 years (31/03/2016) | Incidence of prostate cancer with Gleason score ≥8 at diagnosis; <3 years of FU  *Estimates from 1-14 years since randomisation (single screen)* | 1.26 (1.08-1.48) | Incidence of prostate cancer with Gleason score ≥8 at diagnosis; <6 years of FU | 1.00 (0.90-1.11) | Incidence of prostate cancer with Gleason score ≥8 at diagnosis; 10-year median FU (31/03/2016) | 0.91 (0.85-0.98) |
| Multiple^#^ | D'Aquapendente | Cancer-related mortality; 24 months of FU | NR | NR | Mortality timepoint | NA | Incidence of stage IV cancers; FU for 24 months | 0.74 (0.17-3.23) |
| Multiple^#^ | MVTEP | Cancer-related mortality during 24-month FU | NR | NR | Mortality timepoint | NA | Incidence of advanced-stage cancers; FU for 24 months | 0.71 (0.23-2.21) |
| Multiple^#^ | SOMIT | Cancer-related mortality; 24 months of FU | NR | NR | Mortality timepoint | NA | Incidence of cancers with loco-regional or distant metastasis (N1 and/or M1); FU for 24 months | 0.64 (0.22-1.90) |

**Abbreviations.** CI, confidence interval; CRC, colorectal cancer; DOB, date of birth; FOBT, faecal occult blood test; FU, follow-up; HPV, human Papillomavirus; IQR, interquartile range; M1, distant metastasis present; MMS, multimodal screening; MX, metastasis cannot be measured; N1, cancer has spread to nearby lymph nodes; N1, N2, N3, number of nearby lymph nodes that have cancer; NA, not applicable; NPC, nasopharyngeal cancer; NSCLC, non-small cell lung cancer; NR, not reported; RR, relative risk or rate ratio; T3, tumour size or area: cancer has broken through the capsule (covering) of the prostate gland; T4, tumour size or area: cancer has spread into other body organs nearby; TVU, transvaginal ultrasound; UC, usual care; VIA, visual inspection with acetic acid.

**Trial name abbreviations.** CAP, Cluster Randomized Trial of PSA Testing for Prostate Cancer; CNBSS, Canadian National Breast Screening Study; DANTE, Detection and Screening of Early Lung Cancer by Novel Imaging Technology and Molecular Essays Trial, DLCST, Danish Lung Cancer Screening Trial; ERSPC, European Randomized Study of Screening for Prostate Cancer; HIP NY, Health Insurance Plan of Greater New York; ITALUNG, Italian Lung Cancer Screening Trial; LSS, Lung Screening Study; LUSI, German Lung Cancer Screening Intervention Trial; MILD, Multicentric Italian Lung Detection; MVTEP, Standard Diagnostic Procedures With or Without Fludeoxyglucose F 18 Positron Emission Tomography in Finding Cancer in Patients With a Blood Clot in a Vein; NELSON, Nederlands–Leuvens Longkanker Screenings Onderzoek; NLST, National Lung Screening Trial; NORCCAP, Norwegian colorectal cancer prevention; NordICC, Nordic-European Initiative on Colorectal Cancer; PLCO, Prostate, Lung, Colorectal and Ovarian Cancer Screening Trial; SCORE, Screening for COlon Rectum trial; SOME, Screening for Occult Malignancy in Patients with Idiopathic Venous Thromboembolism; SOMIT, Subsequent diagnosis Of Malignancy in patients presenting with Idiopathic venous Thromboembolism; UKCTOS, UK Collaborative Trial of Ovarian Cancer Screening; UKFSST, UK Flexible Sigmoidoscopy Screening Trial; UKLS, UK Lung Cancer Screening Trial.

* Earlier than main analysis timepoint.

** Ideally midpoint (earlier than mortality and after intervention phase has ended).

*** No stage data reported. For screening tests that can prevent cancer, we used the incidence of invasive cancer as a potential late-stage surrogate outcome.

^#^ Different target cancers screened for in each trial.

^§^ Chinese hepatocellular cancer staging system.

^§§^ Clinical-diagnostic staging system with occult, I, II and III stages.

## Table S17. Meta-analysis results from the primary, sensitivity, and subgroup analyses for the association between the “primary” estimate of the absolute incidence of late-stage cancer and the “main” mortality outcome.

| **Primary analysis** | | | | | |
| --- | --- | --- | --- | --- | --- |
| **Cancer** | **N** | **Correlation (95% CI)** | **Slope (95% CI)** | **Intercept (95% CI)** | **R^2^ (95% CI)** |
| Bowel | 11 | 0.58 (0.27-0.93) | 0.42 (0.17-1.06) | -0.12 (-0.20--0.04) | 0.34 (0.07-0.86) |
| Breast | 13 | 0.79 (0.49-0.94) | 0.61 (0.37-0.87) | -0.05 (-0.10--0.01) | 0.62 (0.24-0.88) |
| Cervix | 4 | 0.77 (0.72-1.00) | 0.69 (0.55-2.84) | -0.16 (-0.24-0.12) | 0.59 (0.52-1.00) |
| Lung | 14 | 0.91 (0.84-0.96) | 0.70 (0.52-0.90) | 0.01 (-0.07-0.03) | 0.83 (0.71-0.92) |
| Multiple* | 3 | -0.40 (-1.00-1.00) | -0.75 (-inf-inf) | -1.03 (-1.74-0.94) | 0.16 (0.00-1.00) |
| Ovary | 3 | 0.97 (-1.00-1.00) | 0.98 (-inf-inf) | -0.01 (-0.69-0.17) | 0.94 (0.00-1.00) |
| Prostate | 5 | 0.42 (-1.00-1.00) | 0.59 (-3.83-9.04) | -0.03 (-0.58-1.56) | 0.18 (0.00-1.00) |
| All cancers | 57 | 0.69 (0.47-0.84)^a^ | 0.58 (0.36-0.82) | -0.04 (-0.09--0.01) | 0.47 (0.23-0.71) |

^a^ Correlation coefficient excluding trials where the late-stage outcome was defined as invasive cancer was: only UKFSST 0.69; only Finnish cervical screening trial 0.69; both UKFSST and Finnish cervical screening trial 0.69. Correlation coefficient in an unweighted analysis: 0.69.

| **Subgroup analysis: Pre-mortality estimates available** | | | | | |
| --- | --- | --- | --- | --- | --- |
| **Cancer** | **N** | **Correlation (95% CI)** | **Slope (95% CI)** | **Intercept (95% CI)** | **R^2^** |
| Bowel | 4 | 0.92 (0.66-1.00) | 0.91 (0.25-2.21) | -0.04 (-0.17-0.44) | 0.84 (0.43-1.00) |
| Breast | 3 | 0.99 (-1.00-1.00) | 0.95 (-inf-inf) | -0.11 (-0.47-0.07) | 0.97 (0.00-1.00) |
| Cervix | NA | NA | NA | NA | NA |
| Lung | 5 | 0.94 (0.42-1.00) | 0.66 (0.09-1.10) | 0.03 (-0.33-0.05) | 0.89 (0.36-1.00) |
| Multiple* | NA | NA | NA | NA | NA |
| Ovary | NA | NA | NA | NA | NA |
| Prostate | 3 | -0.96 (-1.00-1.00) | -0.87 (-inf-inf) | -0.04 (-0.04-0.45) | 0.91 (0.00-1.00) |
| All cancers | 15 | 0.74 (0.49-0.90) | 0.63 (0.43-0.92) | 0.00 (-0.06-0.03) | 0.54 (0.24-0.81) |

| **Sensitivity analysis: Late stage defined as stage III+** | | | | | |
| --- | --- | --- | --- | --- | --- |
| **Cancer** | **N** | **Correlation (95% CI)** | **Slope (95% CI)** | **Intercept (95% CI)** | **R^2^** |
| Bowel | NA | NA | NA | NA | NA |
| Breast | 5 | 0.28 (-1.00-1.00) | 0.13 (-0.50-0.53) | -0.11 (-0.25-0.00) | 0.08 (0.00-1.00) |
| Cervix | NA | NA | NA | NA | NA |
| Lung | 14 | 0.87 (0.75-0.95) | 0.65 (0.46-0.84) | 0.00 (-0.08-0.03) | 0.76 (0.56-0.90) |
| Multiple* | NA | NA | NA | NA | NA |
| Ovary | 3 | 0.97 (-1.00-1.00) | 0.98 (-inf-inf) | -0.01 (-0.69-0.17) | 0.94 (0.00-1.00) |
| Prostate | NA | NA | NA | NA | NA |
| All cancers | 30 | 0.67 (0.33-0.89) | 0.49 (0.20-0.75) | -0.02 (-0.09-0.02) | 0.45 (0.11-0.80) |

**Abbreviations.** CI, confidence interval; Inf, infinite; NA, not available (fewer than three trials available for analysis).

Note. “All cancers” includes all cancer types included in this meta-analysis. Liver, nasopharyngeal, and oral cancers are not reported separately because there were fewer than three trials for each.

* Different target cancers screened for in each trial.

## Table S18. Meta-analysis results from the primary, sensitivity, and subgroup analyses for the association between the “primary” estimate of the proportion of late-stage cancer (among all cancer diagnoses) and the “main” mortality outcome.

| **Primary analysis** | | | | | |
| --- | --- | --- | --- | --- | --- |
| **Cancer** | **N** | **Correlation (95% CI)** | **Slope (95% CI)** | **Intercept (95% CI)** | **R^2^** |
| Bowel | 10 | 0.21 (-0.25-0.48) | 0.18 (-0.74-0.59) | -0.15 (-0.26-0.05) | 0.05 (0.00-0.26) |
| Breast | 13 | 0.53 (0.16-0.87) | 0.38 (0.09-1.23) | -0.04 (-0.14-0.06) | 0.28 (0.03-0.75) |
| Cervix | 3 | 0.04 (-1.00-1.00) | 0.03 (-inf-inf) | -0.39 (-0.53-0.03) | 0.00 (0.00-1.00) |
| Lung | 14 | 0.67 (0.27-0.85) | 0.45 (0.19-0.68) | 0.02 (-0.09-0.14) | 0.44 (0.07-0.71) |
| Multiple* | 3 | -0.88 (-1.00-1.00) | -0.43 (-inf-inf) | -0.98 (-1.17--0.66) | 0.77 (0.00-1.00) |
| Ovary | 3 | 0.72 (-1.00-1.00) | 1.07 (-inf-inf) | 0.07 (-0.69-0.21) | 0.52 (0.00-1.00) |
| Prostate | 5 | 0.81 (-1.00-1.00) | 0.91 (-5.65-1.87) | 0.38 (-2.66-1.00) | 0.65 (0.01-1.00) |
| All cancers | 55 | 0.30 (0.00-0.54) | 0.21 (0.00-0.39) | -0.07 (-0.15--0.01) | 0.09 (0.00-0.29) |

| **Subgroup analysis: Pre-mortality estimates available** | | | | | |
| --- | --- | --- | --- | --- | --- |
| **Cancer** | **N** | **Correlation (95% CI)** | **Slope (95% CI)** | **Intercept (95% CI)** | **R^2^** |
| Bowel | 4 | -0.13 (-1.00-1.00) | -1.57 (-25.83-17.23) | -0.30 (-3.02-2.08) | 0.02 (0.00-1.00) |
| Breast | 3 | 0.79 (-1.00-1.00) | 1.46 (-inf-inf) | 0.05 (-0.32-0.40) | 0.62 (0.00-1.00) |
| Cervix | NA | NA | NA | NA | NA |
| Lung | 5 | 0.70 (-0.28-1.00) | 0.41 (-0.57-1.70) | 0.04 (-0.35-0.21) | 0.49 (0.04-1.00) |
| Multiple* | NA | NA | NA | NA | NA |
| Ovary | NA | NA | NA | NA | NA |
| Prostate | 3 | 0.52 (-1.00-1.00) | 0.54 (-inf-inf) | 0.23 (-1.46-0.36) | 0.27 (0.00-1.00) |
| All cancers | 15 | 0.30 (-0.19-0.63) | 0.18 (-0.18-0.44) | 0.00 (-0.13-0.08) | 0.09 (0.00-0.39) |

| **Sensitivity analysis: Late stage defined as stage III+** | | | | | |
| --- | --- | --- | --- | --- | --- |
| **Cancer** | **N** | **Correlation (95% CI)** | **Slope (95% CI)** | **Intercept (95% CI)** | **R^2^** |
| Bowel | NA | NA | NA | NA | NA |
| Breast | 5 | -0.16 (-1.00-1.00) | -0.06 (-0.48-0.31) | -0.15 (-0.24-0.08) | 0.03 (0.00-1.00) |
| Cervix | NA | NA | NA | NA | NA |
| Lung | 14 | 0.62 (0.19-0.82) | 0.40 (0.13-0.62) | 0.01 (-0.11-0.12) | 0.39 (0.04-0.68) |
| Multiple* | NA | NA | NA | NA | NA |
| Ovary | 3 | 0.72 (-1.00-1.00) | 1.07 (-inf-inf) | 0.07 (-0.69-0.21) | 0.52 (0.00-1.00) |
| Prostate | NA | NA | NA | NA | NA |
| All cancers | 30 | 0.32 (-0.08-0.61) | 0.21 (-0.05-0.42) | -0.05 (-0.16-0.02) | 0.10 (0.00-0.38) |

**Abbreviations.** CI, confidence interval; Inf, infinite; NA, not available (fewer than three trials available for analysis).

Note. “All cancers” includes all cancer types included in this meta-analysis. Liver, nasopharyngeal, and oral cancers are not reported separately because there were fewer than three trials for each.

* Different target cancers screened for in each trial.

## **Section 5. Selected aspects of limitations in the reported trial data**

## Table S19. Various aspects of the limitations in the reported data from 57 trials.

All questions relate to the selected references for each trial. Please consult Section 3 for the complete reference list.

Availability of cancer treatment information

Question: “Was the offered treatment and/or adherence to treatment more different between trial arms than might be expected (within the different cancer stages)?”

“Unavailable”: there was not enough information available to allow this judgment.

“Likely similar”: treatment options and adherence were similar between the trial arms.

“Likely dissimilar”: the offered treatment and/or adherence was different enough between trial arms to raise concerns.

Missing outcome data

Question: “Is there high missingness overall (≥10%) or a large difference in missingness between trial arms (≥5% absolute difference) for either the late-stage cancer incidence or cancer-specific mortality, or a large difference in data missingness between the late-stage cancer incidence and mortality outcomes (≥5% absolute difference)?”

“Unavailable”: there was not enough information to allow this judgment.

“No”: no large differences reported.

“Yes”: large differences reported on at least one of the sub-questions.

| **Cancer type** | **Trial acronym/ name** | **“Primary” late-stage outcome** | **“Main” mortality outcome** | **Availability of cancer treatment information** | **Missing outcome data** |
| --- | --- | --- | --- | --- | --- |
| Bowel | Finnish | CRC with N≥1 (left and right colon); FU until 31/12/2011^2^ | CRC mortality; FU until 31/12/2012 (median 4.5, range 0.0-8.3 years)^4^ | Unavailable | Yes |
| Bowel | Funen | Late-stage CRC (Dukes’ C, distant spread, no classification); FU until Aug 1995^10^ | CRC mortality (deaths from CRC or complications from treatment); FU until Aug 1995^10^ | Unavailable | No |
| Bowel | Gothenburg | Dukes’ D CRC; all cohorts; FU until 31/12/2001^16^ | CRC mortality; all cohorts; FU until 31/12/2001^16^ | Unavailable | No |
| Bowel | Minnesota | Dukes’ D CRC; 13 years of FU from entry^17^ | CRC mortality; 13 years of FU from entry^17^ | Likely similar | Unavailable |
| Bowel | NORCCAP | Dukes’ C or distant spread in people aged 55-64 years: 31/12/2006^20^ | CRC mortality in people aged 55-64 years; FU until 31/12/2015^22^ | Unavailable | Yes |
| Bowel | NordICC | Dukes’ C or D CRC; median 10-year FU from entry^23^ | CRC mortality; 10-year risk^23^ | Unavailable | Yes |
| Bowel | Nottingham | Dukes’ C or D CRC; FU until 30/06/1995^25^ | CRC mortality (verified cause of death); FU until 30/06/1995^25^ | Unavailable | No |
| Bowel | PLCO (bowel) | Stage III-IV CRC; 13 years of FU from entry or until 31/12/2009^33^ | CRC mortality; 13 years of FU from entry or until 31/12/2009^33^ | Likely similar | Unavailable |
| Bowel | SCORE | Stage III-IV CRC; FU until 31/12/2007^35^ | CRC mortality; FU until 31/12/2008 (5 centres) or 31/12/2007 (1 centre)^35^ | Unavailable | Unavailable |
| Bowel | Telemark | Dukes’ C or D CRC; FU until 1993^37^ | CRC mortality; FU until 1995^39^ | Unavailable | No |
| Bowel | UKFSST | CRC incidence (all sites);  FU until 31/12/2008^41^ | CRC mortality (death verified by expert coder); FU until 31/12/2008^41^ | Unavailable | No |
| Breast | CNBSS-1 | Node-positive BC: 7 years of FU from entry^45^ | BC mortality; 7 years of FU from entry^45^ | Unavailable | Yes |
| Breast | CNBSS-2 | Node-positive BC: 7 years of FU from entry^44^ | BC mortality; 7 years of FU from entry^44^ | Unavailable | Yes |
| Breast | Edinburgh | Stage III-IV BC (cohort 1); 7 years of FU from entry^56^ | BC mortality (cohort 1); 7 years of FU from entry^56^ | Likely similar | Unavailable |
| Breast | Gothenburg | Node-positive BC detected in screening period (including exit screen)^59^ | BC mortality in cases diagnosed in screening period (including exit screen) (blinded review of cause of death); FU until 31/12/1996^58^ | Unavailable | Yes |
| Breast | HIP NY | Node-positive BC; FU for 5 years from entry^68^ | BC mortality; FU for 10 years from entry^71^ | Unavailable | Yes |
| Breast | Malmo | Stage II-IV BC; FU until 31/12/1986^78^ | BC mortality; FU until 31/12/1986^78^ | Likely similar | Unavailable |
| Breast | Mumbai | Stage III-IV BC; FU until May 2018 (March 2019?)^82^ | BC mortality; FU until May 2018 (March 2019?)^82^ | Likely similar | Unavailable |
| Breast | Russia / WHO | Node-positive BC; FU until 31/12/1994^85^ | BC mortality in cases diagnosed until 31/12/1994; FU until 31/12/1997^85^ | Unavailable | Unavailable |
| Breast | Shanghai | Node-positive BC; FU until 31/12/2000^87^ | BC mortality; FU until 31/12/2000^87^ | Likely similar | No |
| Breast | Stockholm | Stage II-IV BC detected in screening period (including exit screen) (1981-1986)^92^ | BC mortality in cases diagnosed in screening period (including exit screen); FU until 31/12/1994^92^ | Likely similar | Unavailable |
| Breast | Trivandrum | Stage III-IV BC; FU until 31/12/2019^93^ | BC mortality; FU until 31/12/2019^93^ | Likely similar | Unavailable |
| Breast | Two-County | Stage II-IV BC; FU until 31/12/1984^100^ | BC mortality; FU until 31/12/1984^100^ | Unavailable | Unavailable |
| Breast | UK Age | Node-positive BC; diagnosed during the intervention period (before first NHSBSP screen)^110^ | BC mortality in cases diagnosed during the intervention period (before first NHSBSP screen); FU until 31/12/2011^113^ | Unavailable | Unavailable |
| Cervical | Finnish | Invasive cervical cancer; FU until 31/12/2007^114^ | Cervical cancer mortality; FU until 31/12/2007^114^ | Unavailable | Unavailable |
| Cervical | Mumbai | Stage ≥IIB invasive cervical cancer; FU until 31/12/2011^115^ | Cervical cancer mortality; FU until 31/12/2011^115^ | Likely dissimilar | Yes |
| Cervical | Osmanabad | Stage II+ invasive cervical cancer; FU until 31/12/2007^117^ | Cervical cancer mortality; FU until 31/12/2007^117^ | Unavailable | Unavailable |
| Cervical | Tamil Nadu | Stage II+ invasive cervical cancer; FU until 31/12/2006^119^ | Cervical cancer mortality; FU until 31/12/2006^119^ | Unavailable | Yes |
| Liver | Qidong | Stage III liver cancer (Chinese HCC staging system); FU until 31/12/1995^120^ | Liver cancer mortality; FU until 31/12/1995^120^ | Unavailable | Unavailable |
| Liver | Shanghai | Stage III liver cancer (Chinese HCC staging system); FU until 31/12/1997^121^ | Liver cancer mortality; FU until 31/12/1997^121^ | Likely similar | Unavailable |
| Lung | Czech Study | Stage III lung cancer (incident); FU for 6 years from entry^123^ | Lung cancer mortality; FU for 15 years from entry^125^ | Likely similar | Unavailable |
| Lung | DANTE | Stage II-IV lung cancer; FU until 15/05/2013^128^ | Lung cancer mortality; FU until 15/05/2013^128^ | Unavailable | No |
| Lung | DLCST | Stage III-IV lung cancer; FU until 07/04/2015^130^ | Lung cancer mortality; FU until 07/04/2015^130^ | Unavailable | No |
| Lung | ITALUNG | Stage III-IV lung cancer; FU until 31/12/2013^131^ | Lung cancer mortality; FU until 31/12/2014^131^ | Unavailable | Yes |
| Lung | Johns Hopkins | Stage II-IV lung cancer; FU for up to 9 years from entry^134^ | Lung cancer mortality; FU for up to 9 years from entry^134^ | Unavailable | Unavailable |
| Lung | LSS | Stage III-IV lung cancer; baseline screen, year 1 screen and interval cancers^139^ | Lung cancer mortality; FU until 31/12/2005^137^ | Unavailable | Yes |
| Lung | LUSI | Stage II-IV lung cancer: FU for 7 years from entry^141^ | Lung cancer mortality; FU until 30/04/2018^141^ | Unavailable | No |
| Lung | Mayo Lung Project | Stage III-IV lung cancer; FU until 01/07/1983^147^ | Lung cancer mortality; FU until 01/07/1983^147^ | Unavailable | Unavailable |
| Lung | Memorial Sloan Kettering | Stage II-IV lung cancer; FU for up to 9 years from entry^134^ | Lung cancer mortality; FU for up to 9 years from entry^134^ | Unavailable | Unavailable |
| Lung | MILD | Stage II-IV lung cancer; FU until June 2018^157^ | Lung cancer mortality; FU until June 2018^157^ | Unavailable | No |
| Lung | NELSON | Stage III-IV lung cancer in men; FU for 10 years from entry or until 31/12/2015^159^ | Lung cancer mortality in men; FU for 10 years from entry or 31/12/2015^159^ | Unavailable | No |
| Lung | NLST | Stage III-IV lung cancer; FU until 31/12/2009^160^ | Lung cancer mortality; FU until 31/12/2009^162^ | Unavailable | Unavailable |
| Lung | PLCO (lung) | Stage III-IV NSCLC: FU for 7 years from entry^163^ | Lung cancer mortality; FU for 13 years or 31/12/2009^163^ | Likely similar | Unavailable |
| Lung | UKLS | Stage III-IV lung cancer; FU for 4 years from entry^164^ | Lung cancer mortality; FU until 29/02/2020^164^ | Unavailable | Unavailable |
| Naso-pharyngeal | China NPC | Stage III-IV NPC; FU until 31/12/2014^165^ | NPC mortality; FU until 31/12/2014^165^ | Unavailable | Unavailable |
| Oral | Trivandrum | Stage III-IV oral cancer; FU until 31/12/2004^168^ | Oral cancer mortality; FU until 31/12/2004^168^ | Unavailable | Unavailable |
| Ovarian | PLCO (ovarian) | Stage III-IV ovarian cancer (including primary peritoneal and fallopian tube cancer);  FU for 13 years from entry or until 28/02/2010^170^ | Ovarian cancer (including primary peritoneal and fallopian tube cancer) mortality; FU for 13 years from entry or until 28/02/2010^170^ | Likely similar | Unavailable |
| Ovarian | UK Pilot | Stage III-IV ovarian cancer; FU until December 1997^173^ | Ovarian cancer mortality; FU until January 1998^173^ | Likely similar | No |
| Ovarian | UKCTOCS | Stage III-IV primary ovarian cancer; FU until 31/12/2014^174^ | Ovarian cancer mortality; FU until 31/12/2014^174^ | Unavailable | No |
| Prostate | CAP | PC with Gleason score 8-10; <6 years of FU since randomisation^176^ | Definite, probable, or intervention-related PC mortality; FU until 31/03/2016^176^ | Unavailable | Unavailable |
| Prostate | ERSPC | PC with Gleason score 8-10 in men aged 55-69 years; whole trial excluding France; FU until 31/12/2008^195^ | PC mortality in men aged 55-69 years; whole trial excluding France; FU until 31/12/2008^195^ | Unavailable | Unavailable |
| Prostate | ERSPC Pilot 1 | PC with Gleason score ≥3+4;  FU for median 19 years from entry^200^ | PC mortality; FU for median 19 years from entry^200^ | Unavailable | Yes |
| Prostate | Norrkoping | Advanced PC (T3-4, N1 or MX/M1); FU until 31/12/1999^202^ | PC mortality in cancers diagnosed up to 31/12/1999; FU until 31/12/2008^202^ | Unavailable | No |
| Prostate | PLCO (prostate) | PC with Gleason score 8-10; FU through 10 years^203^ | PC mortality; FU for 13 years from entry or until 31/12/2009^204^ | Likely similar | Unavailable |
| Multiple* | D’Aqua-pendente | Stage IV cancer; 24 months FU from entry^210^ | Cancer-specific mortality; 24 months FU from entry^210^ | Unavailable | No |
| Multiple* | MVTEP | ‘Advanced’ cancer; 24 months FU from entry^211^ | Cancer-specific mortality; 24 months FU from entry^211^ | Unavailable | No |
| Multiple* | SOMIT | Cancer with N1 and/or M1; 24 months FU from entry^212^ | Cancer-specific mortality; 24 months FU from entry^212^ | Unavailable | No |

**Abbreviations.** BC, breast cancer; CRC, colorectal cancer; FU, follow-up; HCC, hepatocellular carcinoma; M1, distant metastasis present; MX, metastasis cannot be measured; N1, cancer has spread to nearby lymph nodes; N1, N2, N3, number of nearby lymph nodes that have cancer; NHSBSP, National Health Service Breast Screening Programme; NPC, nasopharyngeal cancer; NSCLC; non-small cell lung cancer; PC, prostate cancer; T3, tumour size or area: cancer has broken through the capsule (covering) of the prostate gland; T4, tumour size or area: cancer has spread into other body organs nearby; WHO, Word Health Organization.

**Trial name abbreviations.** See Table S12 legend.

* Different target cancers screened for in each trial.
