# Supplementary material for: Late-Stage Outcomes as Surrogates for Mortality in Cancer Screening Trials: A Systematic Review and Meta-analysis
Source: Cancer Epidemiol Biomarkers Prev. 2025 Jul 22;34(10):1694–709. doi: 10.1158/1055-9965.EPI-25-0201 (PMC12491949; doi:10.1158/1055-9965.EPI-25-0201)
Supplement: Figure S2 — shows The effect of the timing of the reporting of late-stage incidence on the association with the mortality outcome, by cancer (if data were available for at least two time points). [file epi-25-0201_figure_s2_suppsf2.docx]

## **Figure S2**. The effect of the timing of the reporting of late-stage incidence on the association with the mortality outcome, by cancer (if data were available for at least two time points).

**Lung cancer**

**Breast cancer**

**Bowel cancer**

**Oral cancer**

Prostate cancer

**Ovarian cancer**

**Prostate cancer**
