# Supplementary material for: Late-Stage Outcomes as Surrogates for Mortality in Cancer Screening Trials: A Systematic Review and Meta-analysis
Source: Cancer Epidemiol Biomarkers Prev. 2025 Jul 22;34(10):1694–709. doi: 10.1158/1055-9965.EPI-25-0201 (PMC12491949; doi:10.1158/1055-9965.EPI-25-0201)
Supplement: Figure S3 — shows he association between the screening effect on the proportion of late-stage cancers (among all cancers diagnosed up to the time of the reporting) and the screening effect on mortality (trial-level surrogacy), by cancer type where at least three trials were available for analysis. [file epi-25-0201_figure_s3_suppsf3.docx]

## **Figure S3**. The association between the screening effect on the proportion of late-stage cancers (among all cancers diagnosed up to the time of the reporting) and the screening effect on mortality (trial-level surrogacy), by cancer type where at least three trials were available for analysis.
