# Supplementary material for: Late-Stage Outcomes as Surrogates for Mortality in Cancer Screening Trials: A Systematic Review and Meta-analysis
Source: Cancer Epidemiol Biomarkers Prev. 2025 Jul 22;34(10):1694–709. doi: 10.1158/1055-9965.EPI-25-0201 (PMC12491949; doi:10.1158/1055-9965.EPI-25-0201)
Supplement: Figure S4 — shows the Evaluation of the utility of regarding the 95% CI RR for late-stage incidence as a “forecast” of the final trial result for cancer-specific mortality, only including 15 trial arm comparisons with the late-stage outcome RR reported prior to the primary analysis for mortality but after the screening period had ended. [file epi-25-0201_figure_s4_suppsf4.docx]

## **Figure S4.** Evaluation of the utility of regarding the 95% CI RR for late-stage incidence as a “forecast” of the final trial result for cancer-specific mortality, only including 15 trial arm comparisons with the late-stage outcome RR reported prior to the primary analysis for mortality but after the screening period had ended.


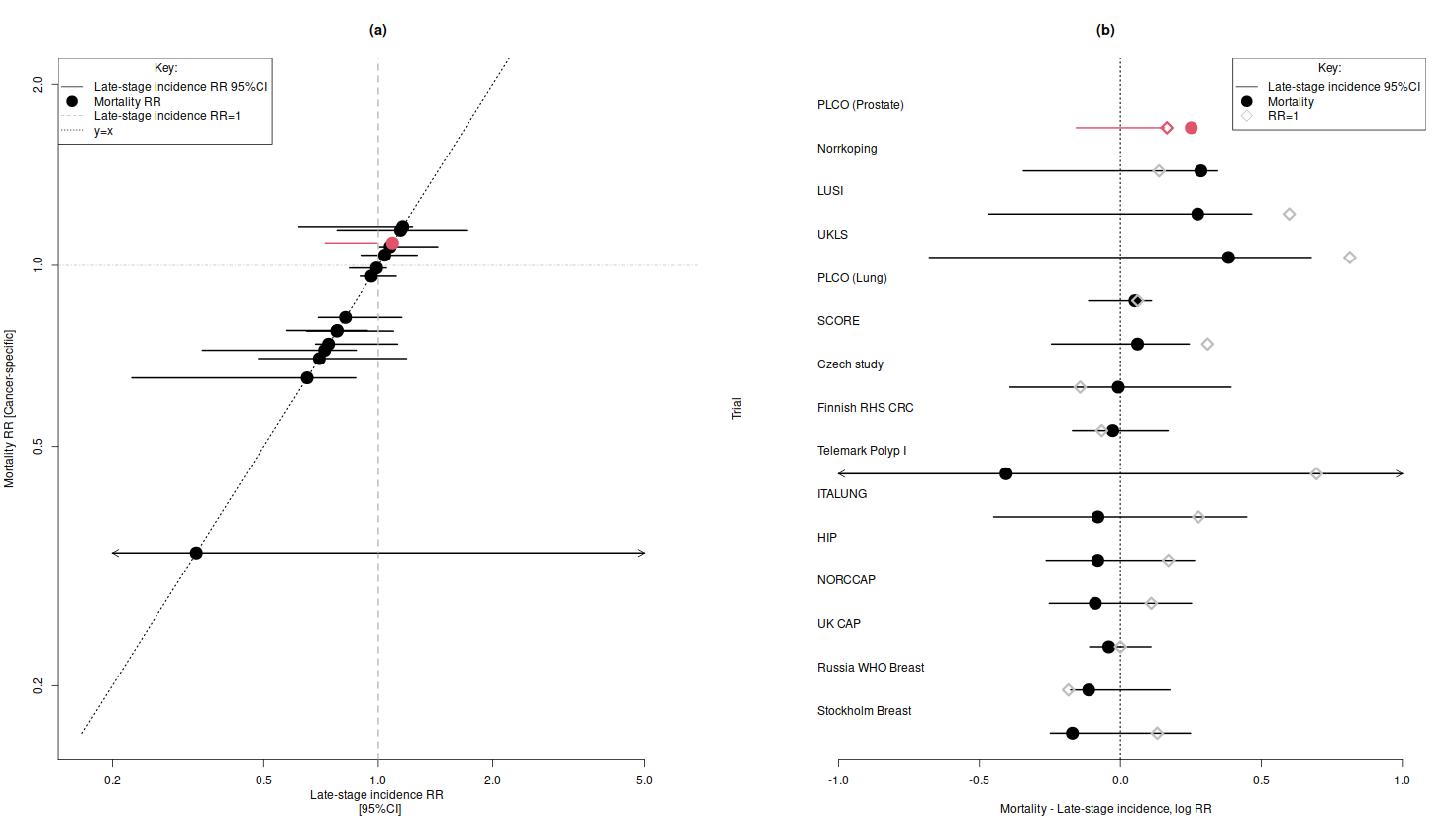
Panel (a) shows the observed RR for mortality (point estimates) vs. the “predicted” 95% CI for the RR based on the observed absolute incidence of late-stage cancer in the intervention vs. the control arm. Panel (b) transforms the data from (a) so that observed – predicted mortality is shown by trial, ordered by where on the prediction the observed mortality fell (mortality at top end of range at top, and bottom end of range at bottom of the plot). Estimates marked red are from trials where the observed mortality exceeds predicted range based on the late-stage outcome; observed mortality RR was included within the 95% CI range of the late-stage outcome for all the other trials shown in black. Wide confidence intervals are due to inclusion of smaller trials.
